# PABPC4 Inhibits SADS-CoV Replication by Degrading the Nucleocapsid Protein Through Selective Autophagy

**DOI:** 10.3390/vetsci12030257

**Published:** 2025-03-10

**Authors:** Chenchen Zhao, Yan Qin, Haixin Huang, Wei Chen, Yanqing Hu, Xinyu Zhang, Yuying Li, Tian Lan, Wenchao Sun

**Affiliations:** 1 Wenzhou Key Laboratory for Virology and Immunology, Institute of Virology, Wenzhou University, Wenzhou 325035, China; zhaochenchen0508@163.com (C.Z.); qinyan_qy@163.com (Y.Q.); huanghaixinn@163.com (H.H.); 23451039005@stu.wzu.edu.cn (W.C.); j648260369@gmail.com (X.Z.); 15858559633@163.com (Y.L.); 2 The Second Affiliated Hospital of Wenzhou Medical University, Wenzhou 325027, China; huyanqing0811@163.com

**Keywords:** PABPC4, SADS-CoV, MARCHF8, NDP52, selective autophagy

## Abstract

Swine acute diarrhea syndrome coronavirus (SADS-CoV) is a novel enteric coronavirus that causes severe clinical diarrhea and intestinal pathological injury in pigs. Selective autophagy is an important mechanism of host defense against virus invasion. However, the mechanism through which SADS-CoV-mediated selective autophagy mediates the innate immune response remains unknown. Here, we report that the host protein PABPC4 can inhibit SADS-CoV replication through targeting and degrading its N protein. Furthermore, we demonstrate that PABPC4 recruits MARCHF8 (an E3 ubiquitin ligase), which ubiquitinates the N protein and is degraded via NDP52/CALCOCO2 (a selective autophagy cargo receptor). Taken together, these findings reveal a new mechanism by which PABPC4 inhibits virus replication, and reveal a new target for antiviral drug development.

## Simple Summary

SADS-CoV is a newly emerging coronavirus affecting the swine industry worldwide. However, our understanding of its pathogenic pathways remains limited. In this study, the researchers aimed to determine the role of the host factor, poly(A)-binding protein 4 (PABPC4), in SADS-CoV infection. This study found that PABPC4 inhibits SADS-CoV replication by degrading the nucleocapsid protein through selective autophagy. This discovery could potentially contribute to developing novel antiviral strategies to prevent and control SADS-CoV infections, protecting the pig industry from significant economic losses.

## 1. Introduction

Swine acute diarrhea syndrome coronavirus (SADS-CoV) is an enveloped, single-stranded, positive-sense RNA virus belonging to the genus *Alphacoronavirus* subgroup of the *Coronaviridae* family. SADS-CoV was first discovered in Guangdong Province in China in 2017 [1] and spread rapidly in Fujian, Guangxi, and other areas in China [2,3,4,5]. SADS-CoV has caused the widespread death of piglets and obvious economic losses. The clinical symptoms of SADS-CoV-infected animals include severe acute diarrhea, vomiting, and weight loss, and the mortality rate in piglets ≤ 5 days of age can reach 90% [2]. Pigs are a major reservoir of this disease, but research has shown that SADS-CoV can also proliferate in multiple vertebrate-derived cells, including those from humans, mice, birds, bats, mink, canines, and monkeys [6]. This finding suggests that SADS-CoV has zoonotic potential. The SADS-CoV genome consists of open reading frame (ORF) 1a (ORF1a), ORF1b, S, ORF3, E, M, ORF6, N, and ORF7, and the whole length is 27 kb [7]. The N protein shows the greatest conservation among all structural proteins in CoVs [8]. Recently, an increasing number of studies have suggested that the N protein functions through various pathways, such as autophagy, apoptosis, and the evasion of the host innate immune response [8,9].

Autophagy is a cellular process that occurs widely in eukaryotic cells [10]. It is a lysosome-dependent degradation pathway and an important mechanism for cellular self-protection. Based on the presence of a selective degradation substrate, autophagy can be divided into selective autophagy and nonselective autophagy. Cargo receptor proteins confer selectivity in autophagy by binding to both the cargo and ATG8-family proteins [11] on the internal membrane vesicle membrane, thereby tethering the cargo to the nascent autophagosomal region [12]. The main cargo receptors include sequestosome 1 (SQSTM1/p62) [13], nuclear dot protein 52 kDa (NDP52/CALCOCO2) [14], optineurin (OPTN) [15], and neighbor of BRCA1 (NBR1) [16,17]. Autophagy has been found to be involved in many viral infection processes. For instance, during Porcine Epidemic Diarrhea (PEDV) infection, BST2 binds to the viral nucleocapsid protein and suppresses PEDV replication by selective autophagy [18]; and OPTN suppresses herpes simplex virus (HSV) replication by selectively targeting the HSV-1 tegument protein VP16 and the fusion glycoprotein Gb, thereby protecting the CNS from viral infection [19]. In contrast, some viruses exploit autophagy to stimulate their own proliferation. For example, in the H7N9 influenza A virus, PB1 promotes the K27 polyubiquitination of MAVS, recruits NBR1, and enhances MAVS autophagic degradation to aid viral replication [20]. Similarly, the deubiquitinase encoded in the N-terminal of EBV’s BPLF1 interacts with SQSTM1/p62 and removes its ubiquitin groups, thus inhibiting selective autophagy [21]. However, how virus-induced selective autophagy influences SADS-CoV replication has not been determined.

PABPC4 is a poly(A)-binding protein that is conserved and expressed extensively in eukaryotes [22]. Poly(A)-binding proteins, such as those involved in mRNA polyadenylation, nonsense-mediated decay (NMD), the stress response, and the control of mRNA translation initiation, play important roles in the regulation of mRNA homeostasis [23,24]. To date, research on poly(A)-binding proteins has focused mainly on mRNA homeostasis, while studies on the relationship between poly(A)-binding proteins and viral replication are rare. According to the existing literature, PABPC4 can interact with the nucleocapsid proteins of various coronaviruses and degrade nucleocapsid proteins through autophagy [25]. However, whether SADS-CoV and PABPC4 have the same relationship is unknown. In this study, we showed that PABPC4 inhibits viral replication in SADS-CoV-infected cells. Further experiments demonstrated that PABPC4 employs MARCHF8 to ubiquitinate the N protein. Subsequently, NDP52 transports this ubiquitinated N protein to the autophagosomes for selective degradation. Taken together, the results of this study provide novel insights into how cells inhibit viral replication through the selective autophagic degradation of the N protein.

## 2. Materials and Methods

### 2.1. Antibodies, Reagents, Plasmids, and siRNAs

Anti-PABPC4 (14960-1-AP), anti-GAPDH (60004-1-Ig), anti-MARCHF8 (14119-1-AP), and anti-NDP52 (12229-1-AP) were provided by Proteintech. Anti-HA (bsm-33003 m) and anti-FLAG (bsm-33346 M) antibodies were purchased from Bioss. The anti-SADS-CoV N proteins, which were previously generated through monoclonal antibody experiments, have been preserved in our laboratory. Chloroquine (C193834), MG132 (R166528), and 3MA (M486160) were purchased from Aladdin. The gene-encoding N protein from SADS-CoV was cloned and inserted into the pCAGGS-FLAG and pXJ40-HA vectors. The entire cDNA sequences of Sus scrofa (pig) (GenBank accession number 100515840) and *Chlorocebus sabaeus* (green monkey) (GenBank accession number 103225052) PABPC4 were cloned and inserted into pXJ40-HA and pCAGGS-FLAG vectors. The entire cDNA sequences of Sus scrofa (GenBank accession number 100157416) and *C. sabaeus* (GenBank accession number 103215808) MACHF8 were cloned and inserted into a pXJ40-HA vector. The entire cDNA sequences of Sus scrofa (GenBank accession number 100523089) and *C. sabaeus* (GenBank accession number 103243627) NDP52 were cloned and inserted into a pXJ40-HA vector. *C. sabaeus* PABPC4, MARCHF8, and NDP52 small interfering RNAs (siRNAs) were designed and purchased from Tsingke Biotech, together with a control siRNA (Table 1). The primers used were synthesized by Sangon Biotech. All recombinant plasmids were confirmed by Sanger sequencing.

### 2.2. Cell Culture and Viruses

Human embryonic kidney cells (HEK-293T cells, BFN60700110A, from ATCC), Vero-E6 cells (Vero, CBP60972, from ATCC), and ST cells (226—596823—812, from ATCC) were cultured in Dulbecco’s modified Eagle’s medium (DMEM, C11995500BT, HyClone) containing 10% fetal bovine serum (FBS, SA211.02, CELLMAX) at 37 °C in a 5% CO_2_ environment. The SADS-CoV strain was proliferated and previously stored in Wenzhou University laboratory. For SADS-CoV infection, VERO-E6 cells were added to 10 mg/mL trypsin in DMEM.

### 2.3. RNA Isolation and Quantitative PCR Analysis

TRIzol reagent (B511321, Sangon Biotech, Shanghai, China) was employed to extract total RNA from the cultured cells, which was then reverse transcribed into cDNA by reverse transcriptase (RR036A, Takara, Japan). RT-qPCR was carried out in triplicate. The relative mRNA expression was normalized to the GAPDH expression. Taq Pro Universal SYBR qPCR Master Mix (Q712, Vazyme, Nanjing, China) and an ABI QuantStudio 3 Real-time PCR system were used for all RT-qPCR assays.

### 2.4. Coimmunoprecipitation Assay

Cell samples were collected 24 h after the transfection of the plasmids with cell lysis buffer supplemented with a protease inhibitor. ProteinIso^®^ Protein A/G Resin (DP501, TransGen Biotech, Beijing, China) was used to isolate the cellular proteins. The corresponding antibody was added to the supernatant, which was subsequently eluted with PBS. Prior to Western blotting analysis, the mixture underwent denaturation at 100 °C for 5 min.

### 2.5. Western Blotting Analysis

The cells were washed twice with cold PBS and incubated on ice with lysis buffer (P0013; Beyotime Biotechnology, Shanghai, China) containing a phosphatase inhibitor cocktail (HY-K0022; MedChemExpress, New Jersey, USA) and protease inhibitor cocktail (CW2200; CWBIO, Taizhou, China). The lysates were denatured for 5 min in 5× SDS-PAGE loading buffer (P0015, Beyotime Biotechnology, Shanghai, China) and separated via SDS-PAGE (E304-01, Vazyme, Nanjing, China). The proteins were subsequently transferred to PVDF membranes. The membranes were blocked for 1 h at room temperature and then incubated with the corresponding primary antibody at room temperature for 2 h. Afterwards, the membranes were incubated for 1 h with the appropriate secondary antibody conjugated with HRP. Enhanced chemiluminescence (P10300, NCMbiotech, Suzhou, China) was used to detect the chemiluminescent bands. The protein bands were measured by ImageJ software version 1.8 (Universal Hood II, BIO-RAD Laboratories, Shanghai, China). The original images of all Western blot assays can be found in the Supplementary Materials.

### 2.6. Confocal Immunofluorescence Assay

The cell samples were fixed in 4% paraformaldehyde (P1110, Solarbio, Beijing, China) for 20 min and then permeabilized for 1 h. The primary antibody was incubated for 2 h at room temperature. Then, the cells were incubated with fluorochrome-conjugated secondary antibodies for 2 h at room temperature. Finally, DAPI (C1006, Beyotime, Shanghai, China) was used to stain the nuclei. The images were collected using an inverted microscope.

### 2.7. Cell Viability Assay

A confluent monolayer of cells seeded in 96-well plates was incubated for 12 h with or without the indicated inhibitors. Cell viability was measured by using a Cell Counting Kit-8 assay (Dojindo, Kumamoto Prefecture, Japan) according to the manufacturer’s instructions.

### 2.8. Statistical Analysis

The results presented are all from 3 independent experiments. The statistical analysis of the experimental data in this paper was carried out by one-way ANOVA in GraphPad Prism 8 software and a two-tailed Student’s *t*-test. “ns” denotes not significant; “*” stands for *p* < 0.05; “**” stands for *p* < 0.01; “***” stands for *p* < 0.001; and “****” stands for *p* < 0.0001.

## 3. Results

### 3.1. SADS-CoV Inhibits the Endogenous Expression of PABPC4

**Figure 1:**
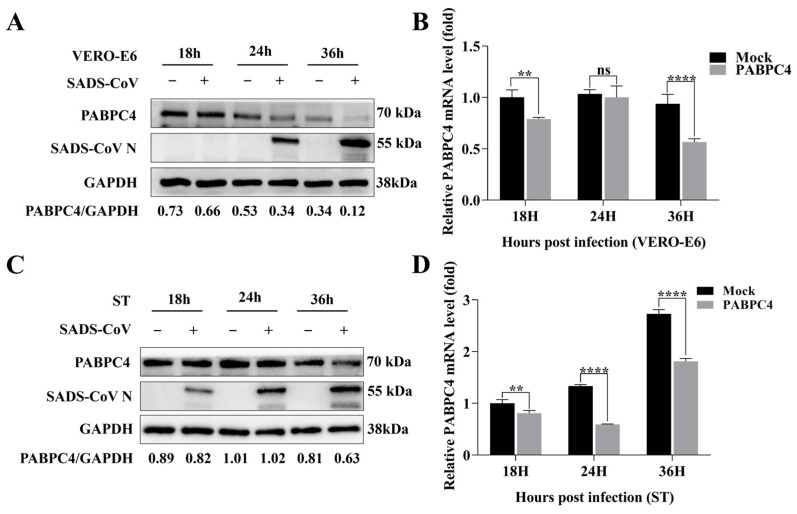
SADS-CoV inhibited the endogenous expression of PABPC4. (**A**,**B**) VERO-E6 cells were infected with SADS-CoV at a multiplicity of infection (MOI) of 1 or mock-infected. Then, the cells were harvested at various time points. The samples were subjected to Western blot analysis and RT-qPCR detection. (**C**,**D**) ST cells were infected or mock-infected with SADS CoV (MOI of 10) and then collected at different times. The samples were subjected to Western blot analysis and RT-qPCR detection. The results are expressed as the means ± SDs of the triplicate experiments. “ns” denotes not significant; Statistical significance was assessed by a two-tailed Student’s *t*-test: ** (*p* < 0.01), and **** (*p* < 0.0001).

To assess the effect of PABPC4 on the proliferation of SADS-CoV, VERO-E6 cells were infected with SADS-CoV at a multiplicity of infection (MOI) of 1 for quantitative real-time PCR (RT-qPCR) and Western blotting analysis. The results showed that, compared with that in uninfected cells, endogenous PABPC4 expression was downregulated at both the protein and mRNA levels during SADS-CoV infection in VERO-E6 cells (Figure 1A,B). Similar to the results obtained with SADS-CoV infection in the ST cells, PABPC4 expression was consistent with the VERO-E6 results (Figure 1C,D). Thus, the expression of PABPC4 was reduced by SADS-CoV infection.

### 3.2. PABPC4 Inhibits SADS-CoV Replication

**Figure 2:**
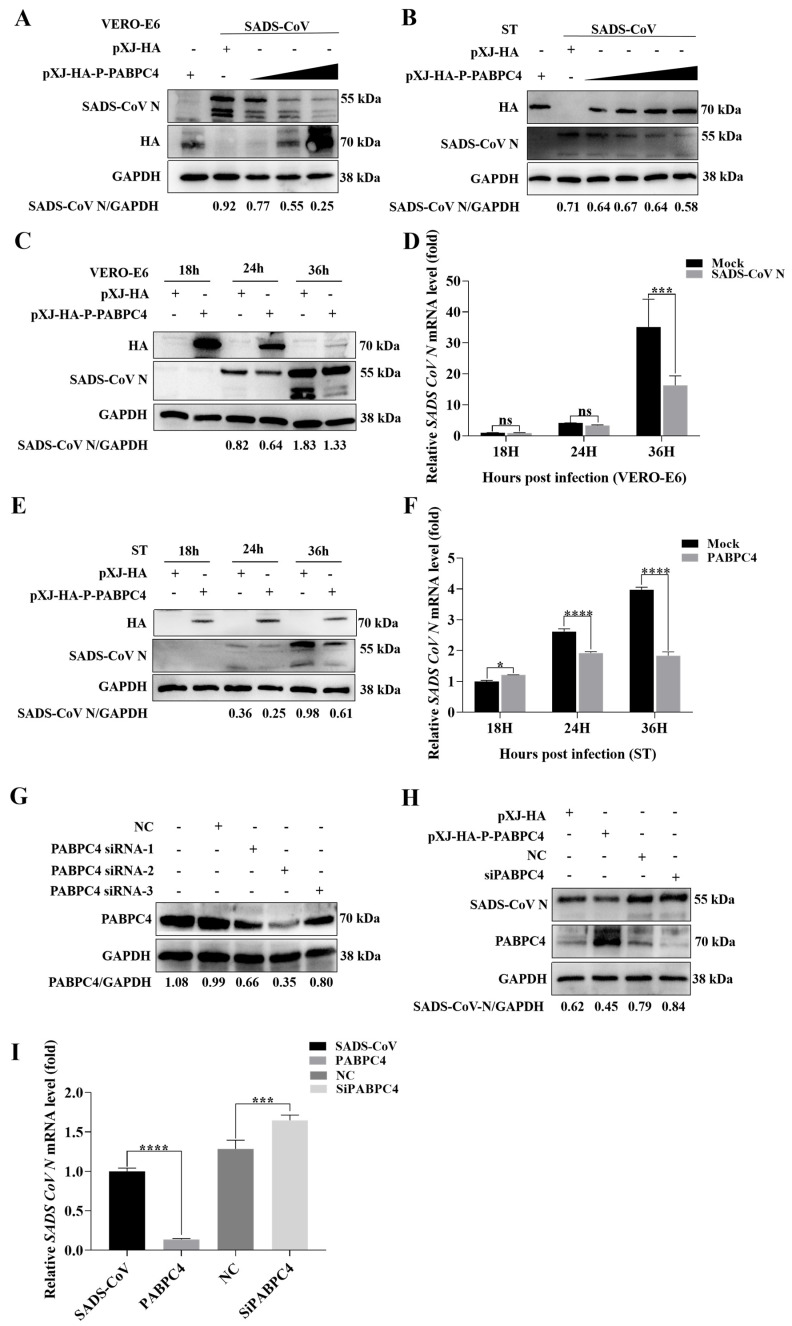
PABPC4 inhibits SADS-CoV replication. (**A**,**B**) VERO-E6 and ST cells were transfected with different concentrations of HA-PABPC4 for 12 h and infected with SADS-CoV at an MOI of 1 or 10. The cell lysates were collected for analysis via Western blotting. GAPDH was used as the sample loading control. (**C**,**D**) For 12 h, VERO-E6 cells were transfected with HA-PABPC4 or an empty vector. Subsequently, they were infected with SADS-CoV at an MOI of 1 and gathered at the indicated times. Analyses of the lysates were performed using Western blot and RT-qPCR. (**E**,**F**) ST cells were transfected for 12 h using HA-PABPC4 or an empty vector. Post-transfection, they were infected with SADS-CoV at an MOI of 1 and collected at specific time intervals. Analyses of the lysates were carried out via Western blot and RT-qPCR. (**G**) VERO-E6 cells were transfected with negative control siRNA or one of three different PABPC4 siRNAs for 24 h. Analyses of the lysates were carried out via Western blot and RT-qPCR. GAPDH served as the control for sample loading. (**H**,**I**) For 24 h, VERO-E6 cells were transfected with a mock, HA-PABPC4, negative-control siRNA, or siRNA-2. Then, they were infected with SADS-CoV at an MOI of 1 for 24 h. Analyses of the lysates were carried out via Western blot and RT-qPCR. GAPDH served as the control for sample loading. The results are expressed as the means ± SDs of the triplicate experiments. “ns” denotes not significant; Statistical significance was assessed by a two-tailed Student’s *t*-test: * (*p* < 0.05), *** (*p* < 0.001), and **** (*p* < 0.0001).

To explore the effect of PABPC4 on SADS-CoV replication, PABPC4 was overexpressed at different concentrations in VERO-E6 and ST cells infected with SADS-CoV, as confirmed by Western blotting analysis. As shown in Figure 2A,B, the replication of SADS-CoV was significantly affected by the concentration gradient of PABPC4. To further validate how PABPC4 affects SADS-CoV replication, the HA-PABPC4 or a mock plasmid were transfected VERO-E6 and ST cells. After 12 h, the cells were infected with SADS-CoV. The cells were collected at various time points and viral mRNA and protein expression levels were analyzed. As predicted, PABPC4 overexpression had lower expression levels of viral proteins and mRNAs than the control vector (Figure 2C–F). These results showed that PABPC4 can inhibit the replication of SADS-CoV.

To confirm these conclusions, we engineered siRNAs targeting PABPC4. Then, three siRNAs were screened, and siRNA-2 was ultimately selected for the subsequent experiments (Figure 2G). VERO-E6 cells were first transfected with siPABPC4 for 18 h and then inoculated with SADS-CoV. The analysis of viral mRNA and protein expression was carried out on the samples. Similarly, the SADS-CoV N protein and mRNA expression in cells transfected with siRNA-2 was significantly greater than that in cells transfected with negative control siRNA (NC) (Figure 2H,I). These results imply that PABPC4 contributes to the inhibition of SADS-CoV replication.

### 3.3. PABPC4 Targets and Degrades the SADS-CoV N Protein

**Figure 3:**
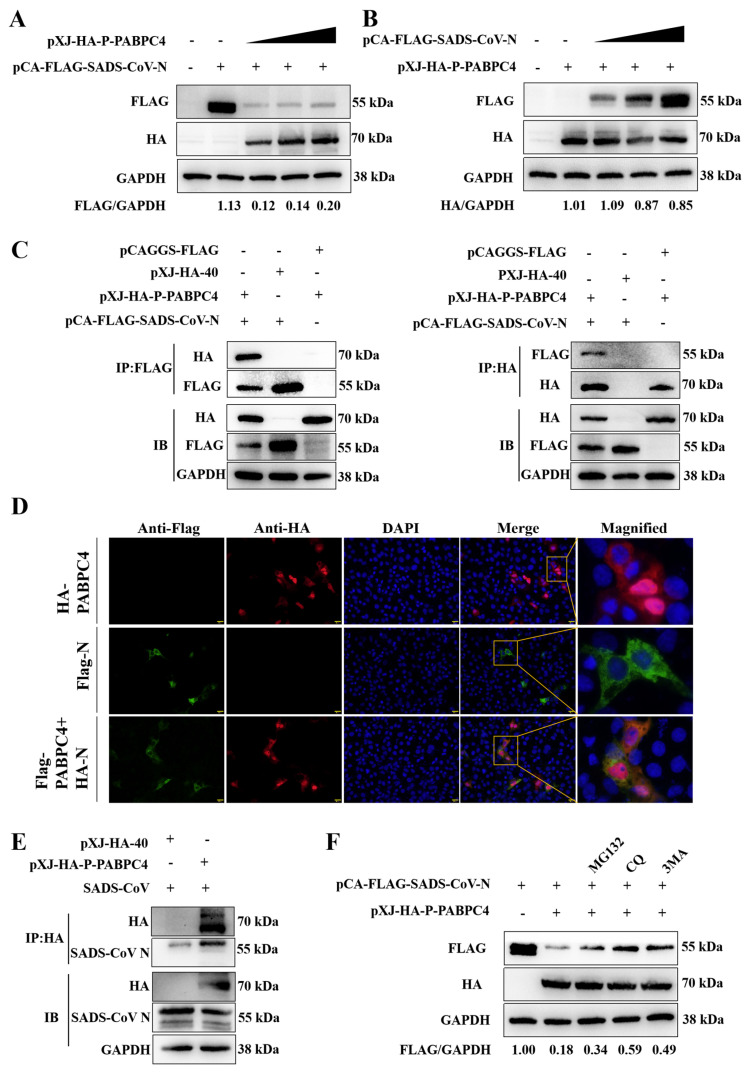
PABPC4 targets and degrades the SADS-CoV N protein. (**A**,**B**) HA-PABPC4 and Flag-N were transfected into HEK-293T cells for 24 h. After that, the collected cell lysates underwent Western blot analysis. A control group was established, with GAPDH serving as the sample normalization control. (**C**) HA-PABPC4, Flag-N, or empty vectors were transfected into HEK-293T cells for 24 h. The lysates were analyzed by co-IP. (**D**) HA-PABPC4, Flag-N, or an empty vector was transfected into the VERO-E6 cells for 24 h. The cells underwent fixation and were then used for immunofluorescence examination. Fluorescence signals were observed via confocal immunofluorescence microscopy. Scale bars: 20 µm. (**E**) Vero-E6 cells were transfected with HA-PABPC4 or empty vector for 24 h and then infected with SADS-CoV at an MOI of 1. The lysates were analyzed by co-IP. (**F**) HEK-293T cells were transfected with HA-PABPC4 and Flag-N for 18 h and then treated with MG132 (5 µm), CQ (10 µm), or 3MA (0.5 µm) for 12 h. Analyses of the lysates were carried out via Western blot.

To explore whether PABPC4 inhibits SADS-CoV replication by interacting with the N protein, the HA-PABPC4 plasmid and the Flag-N plasmid were transfected into HEK-293T cells or a control plasmid for 24 h. As shown in Figure 3A,B, the levels of N protein expression dropped in a dose-dependent way as a result of PABPC4. To confirm this, we use co-transfection HEK-293T cells with the HA-PABPC4 plasmid and Flag-N plasmid via co-IP. The findings indicated that PABPC4 and the SADS-CoV N protein could be efficiently co-immunoprecipitated together (Figure 3C). Subsequently, we utilized confocal immunofluorescence to examine the subcellular localization of PABPC4 and the SADS-CoV N protein. PABPC4 and SADS-CoV N protein were colocalized in the cytoplasm (Figure 3D). Then, we investigated whether the VERO-E6 cells infected with SADS-CoV interacted with PABPC4. The results showed that PABPC4 coimmunoprecipitated with the SADS-CoV N protein (Figure 3E). Next, we further investigated the degradation pathway of the N protein. The proteasome pathway and the lysosome pathway are the primary mechanisms involved in protein degradation. Therefore, we selected MG132 (proteasome inhibitor) and 3-methyladenine (3MA, autophagy inhibitor) and chloroquine (CQ, autophagy inhibitor) for the experiment. As shown in Figure 3F, the degradation of the N protein induced by PABPC4 was impeded by MG132, 3MA, and CQ. Compared with MG132, the degradation of blocking N by 3MA and CQ were more obvious. Interestingly, MG132 also partially restored N protein levels. This observation suggests N protein may be involved in a potential cross-over dialogue between the proteasome and autophagy degradation pathways. However, our study mainly focuses on the N protein autophagic degradation pathway, so the relationship between the two pathways has not been studied in depth. Therefore, we believe that PABPC4 targets the SADS-CoV N protein and degrades the N protein mainly through the autophagy pathway.

### 3.4. PABPC4 Degrades the SADS-CoV N Protein by Activating the PABPC4/MARCH8/NDP52 Autophagosome Pathway

**Figure 4:**
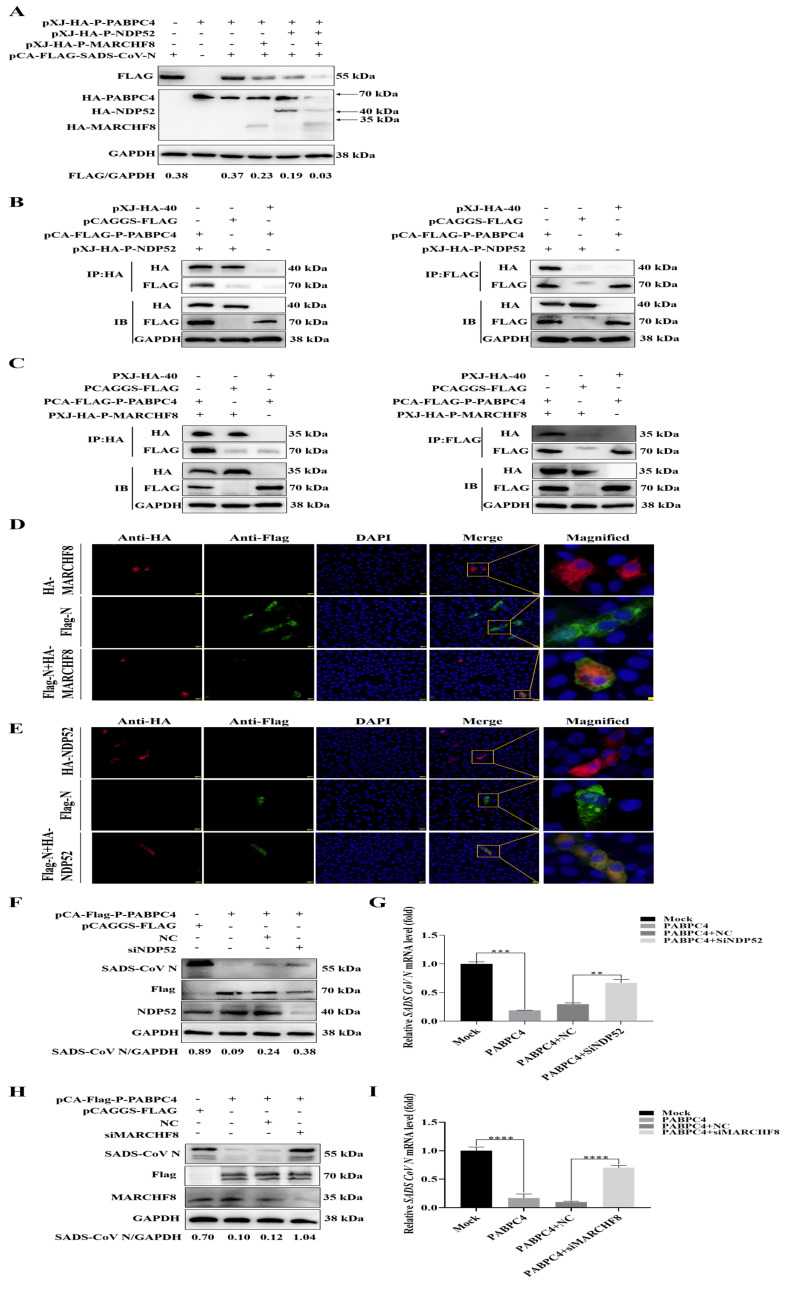
PABPC4 degrades the SADS-CoV N protein by activating the PABPC4/MARCH8/NDP52 autophagosome pathway. (**A**) Flag-N was co-transfected with HA-PABPC4, HA-MARCHF8, or HA-NDP52 into HEK-293T cells for 24 h. Analyses of the lysates were carried out via Western blot. GAPDH served as the control for sample loading. (**B**) Flag-PABPC4 was co-transfected with HA-NDP52 or empty vectors into HEK-293T cells for 24 h. Analyses of the lysates were carried out via co-IP. (**C**) Flag-PABPC4 was co-transfected with HA-MARCHF8 or empty vectors into HEK-293T cells for 24 h. Analyses of the lysates were carried out via co-IP. (**D**) HA-MARCHF8, Flag-N, or an empty vector was transfected into VERO-E6 cells for 24 h. The cells underwent fixation and were then used for immunofluorescence examination. Confocal immunofluorescence microscopy was employed to detect the fluorescence signals. Scale bars: 20 µm. (**E**) HA-NDP52, Flag-N, or an empty vector was transfected into VERO-E6 cells for 24 h. The cells underwent fixation and were then used for immunofluorescence examination. Confocal immunofluorescence microscopy was employed to detect the fluorescence signals. Scale bars: 20 µm. (**F**,**G**) VERO-E6 cells were cotransfected with Flag-PABPC4 and an empty vector, NC, or siRNA-NDP52 for 12 h and infected with SADS-CoV at an MOI of 1. Analyses of the lysates were carried out via Western blot and RT-qPCR. (**H**,**I**) VERO-E6 cells were cotransfected with Flag-PABPC4 and an empty vector, NC, or siRNA-MARCHF8 for 12 h and infected with SADS-CoV at an MOI of 1. Analyses of the lysates were carried out via Western blot and RT-qPCR. The results are expressed as the means ± SDs of the triplicate experiments. Statistical significance was assessed by a two-tailed Student’s *t*-test: ** (*p* < 0.01), *** (*p* < 0.001), and **** (*p* < 0.0001).

MARCHF8, an E3 ubiquitin ligase, can ubiquitinate viral factors so that they are recognized by NDP52 and delivered to the autolysosome for degradation [26,27]. To assess whether MARCHF8 and NDP52 participate in the PABPC4 degradation of the N protein, HA-PABPC4, HA-MARCHF8, HA-NDP52, or Flag-N plasmid were transfected into HEK-293T cells for 24 h. MARCHF8 and NDP52 promoted the degradation of the N protein (Figure 4A). Then, we used Co-IP to explore the relationship between PABPC4 and MARCHF8 or NDP52. As shown in Figure 4B,C, PABPC4 interacted with MARCHF8 and NDP52. To verify whether MARCHF8 and NDP52 affected the PABPC4-mediated degradation of the SADS-CoV N protein, we employed confocal immunofluorescence to examine the subcellular localization of the SADS-CoV N protein and MARCHF8 or NDP52. The results showed the colocalization of MARCHF8 or NDP52 with the SADS-CoV N protein in the cytoplasm (Figure 4D,E). Then, we engineered siRNAs targeting MARCHF8 or NDP52. For 24 h, siRNA (NC, siMARCHF8, siNDP52) or plasmids encoding HA-PABPC4 or FLAG-SADS-CoV-N were co-transfected into the VERO-E6 cells. After that, SADS-CoV was used to infect the cells for 24 h. Western blotting revealed that the cells transfected with siMARCHF8 or siNDP52 expressed more SADS-CoV N protein than the cells transfected with the NC (Figure 4F–I). Taken together, these results demonstrate that PABPC4 degraded the SADS-CoV N protein by activating the PABPC4/MARCH8/NDP52 autophagosome pathway.

## 4. Discussion

Increasing evidence has demonstrated the important role of autophagy in viral infection and antiviral immunity, with viruses using diverse strategies to antagonize cellular autophagy for enhanced viral replication. Porcine coronaviruses also inhibit or use autophagy pathways to enhance viral infection through different pathways. For instance, porcine transmissible gastroenteritis virus (TGEV) induces autophagy to promote cell survival. However, TGEV-induced autophagy restricts TGEV replication, as evidenced by enhanced TGEV replication upon the silencing of the ATG5, ATG7, or LC3 genes [28,29]. PEDV-infected cells induce autophagy to support viral replication, while BST2 can inhibit PEDV replication through the selective autophagy pathway [16]. Porcine delta coronavirus (PDCoV) can enhance viral replication by inducing autophagy through the p38/MAPK pathway [30]. Recent studies have indicated that SADS-CoV promotes its proliferation by inducing autophagy through the ITGA3-Akt-mTOR pathway and the IRE1-JNK-Beclin1 signaling pathway [31,32].

In this study, we first found that the endogenous expression of PABPC4 was significantly inhibited by infection with SADS-CoV. Moreover, the experimental results showed that the replication of SADS-CoV proteins was influenced by the overexpression of PABPC4 in a dose-dependent and time-dependent manner, which was consistent with the results of PEDV, SARS-CoV, and MERS studies. It was demonstrated in a previous study that SP1 binds to the PABPC4 promoter to directly upregulate PABPC4 expression. Coronavirus can downregulate the transcription factor SP1, thereby inhibiting PABPC4 expression and reducing the synthesis of PABPC4 mRNA [28]. The use of engineered siRNAs targeting PABPC4 showed that the replication of SADS-CoV in cells transfected with siRNA-PABPC4 was significantly greater than that in cells transfected with the NC. The experimental results showed that PABPC4 inhibited the replication of SADS-CoV. Subsequently, we investigated which viral protein interacted with PABPC4. The PABPC4 plasmid and the N plasmid were introduced into HEK-293T cells. The results showed that the N protein interacted with PABPC4. Co-IP and IF also confirmed these results. We then found that PABPC4 inhibited N protein expression through the autophagy pathway. Through Western blotting and Co-IP experiments, we found that MARCHF8 and NDP52 interacted with PABPC4 and increased the degradation of the N protein. VERO-E6 cells were transfected with siRNA-MARCHF8 or siRNA-NDP52 and then infected with SADS-CoV. These findings showed that MARCHF8 and NDP52 promoted the PABPC4 degradation of the N protein.

**Figure 5:**
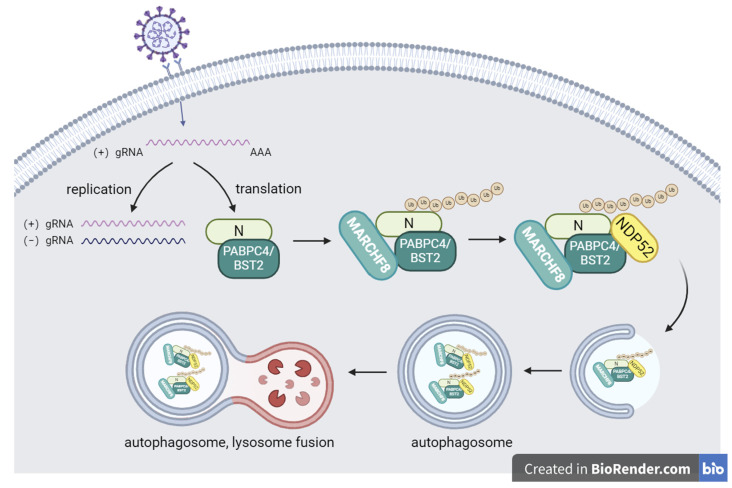
The antiviral mechanism of PABPC4 involves the inhibition of SADS-CoV replication. During SADS-CoV infection, PABPC4 interacts with the SADS-CoV N protein and directs the E3 ubiquitin ligase MARCHF8 towards the N protein for ubiquitination. The cargo receptor NDP52 recognizes the ubiquitinated N protein and conveys it to autolysosomes to enable degradation, leading to the suppression of SADS-CoV infection.

To date, there have been few studies on the mechanism by which SADS-CoV escapes host innate immunity and promotes self-replication and transmission. Our study shows that PABPC4 can interact with the SADS-CoV N protein and inhibit SADS-CoV replication (Figure 5). This finding may lead us to study how to inhibit the replication of SADS-CoV in the host. These discoveries might assist in comprehending the host antiviral response and the creation of new antiviral therapies to prevent and manage SADS-CoV infection.

## 5. Conclusions

This study reveals that PABPC4, as a broad-spectrum anti-coronavirus factor, can inhibit viruses by degrading viral proteins. Our results confirm that PABPC4 inhibits SADS-CoV replication by degrading the N protein through MARCHF8/NDP52. A better understanding of PABPC4’s function during SADS-CoV infection will be key to developing more effective antiviral therapies.

## Figures and Tables

**Table 1:** The sequences of the siRNAs.

Name	Sequence (5′–3′)
si-PABPC4 sense	GAAACAUUCUAUCCUGCAA (dT)(dT)
si-PABPC4 antisense	UUGCAGGAUAGAAUGUUUC (dT)(dT)
si-MARCHF8 sense	GGAAAGAGACUCAAGGCCUA (dT)(dT)
si-MARCHF8 antisense	UAGGCCUUGAGUCUCUUCC (dT)(dT)
si-NDP52 sense	GCAGUUAGAGCACCUGAAA (dT)(dT)
si-NDP52 antisense	UUUCAGGUGCUCUAACUGC (dT)(dT)

## Data Availability

The original contributions presented in this study are included in the article/Supplementary Material. Further inquiries can be directed to the corresponding authors.

## Supplementary Materials

The following supporting information can be downloaded at: https://www.mdpi.com/article/10.3390/vetsci12030257/s1. The original images of all Western blot assays.
