# Supplementary figures and images for: PABPC4 Inhibits SADS-CoV Replication by Degrading the Nucleocapsid Protein Through Selective Autophagy (part 2 of 2)
Source: Vet Sci. 2025 Mar 10;12(3):257. doi: 10.3390/vetsci12030257 (PMC11946123; doi:10.3390/vetsci12030257)

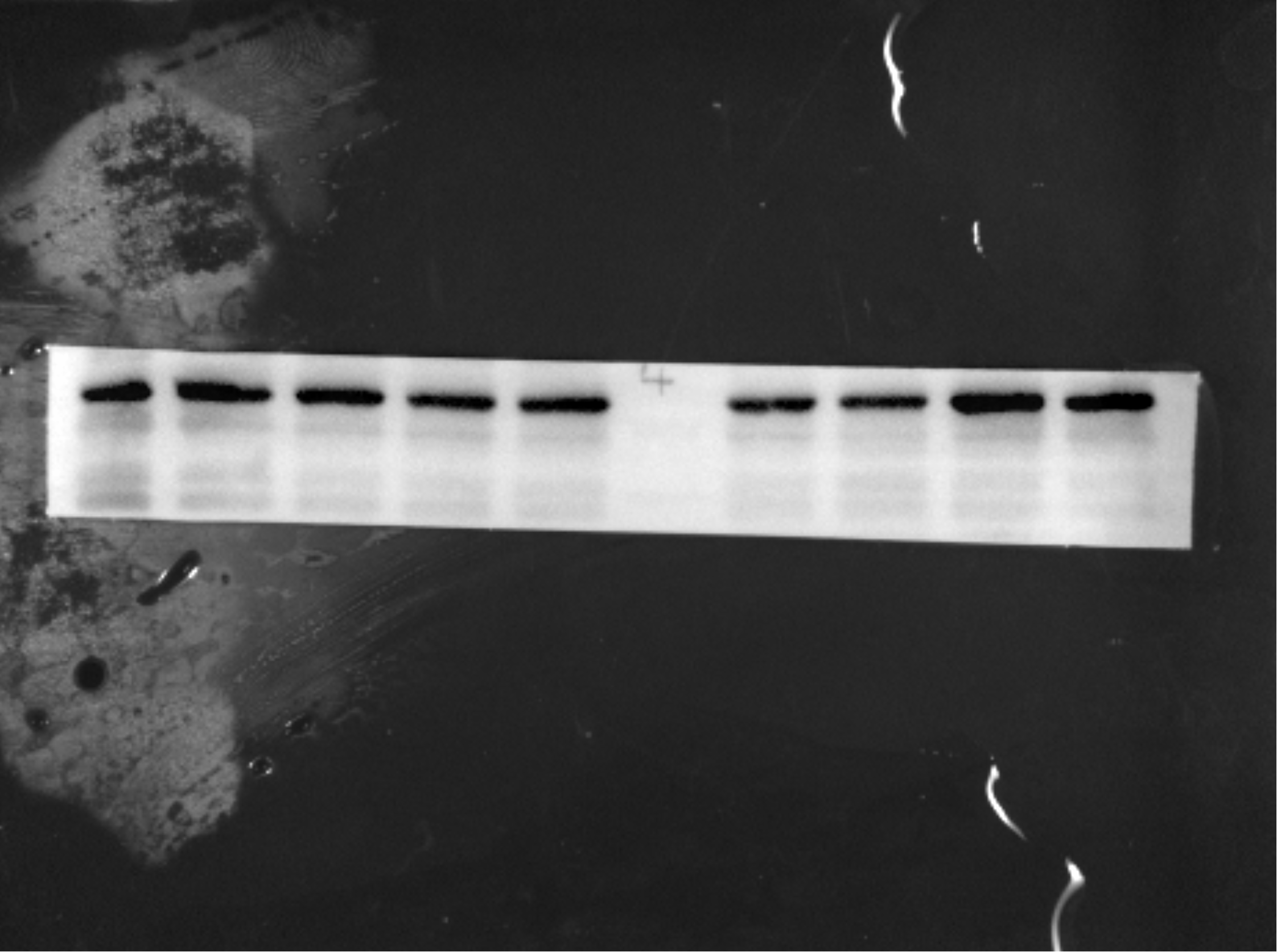

Supplement: Supplementary file 1 [file vetsci-12-00257-s001.zip › PABPC4 original blot images/Fig.4/A/gapdh/merge.tif]

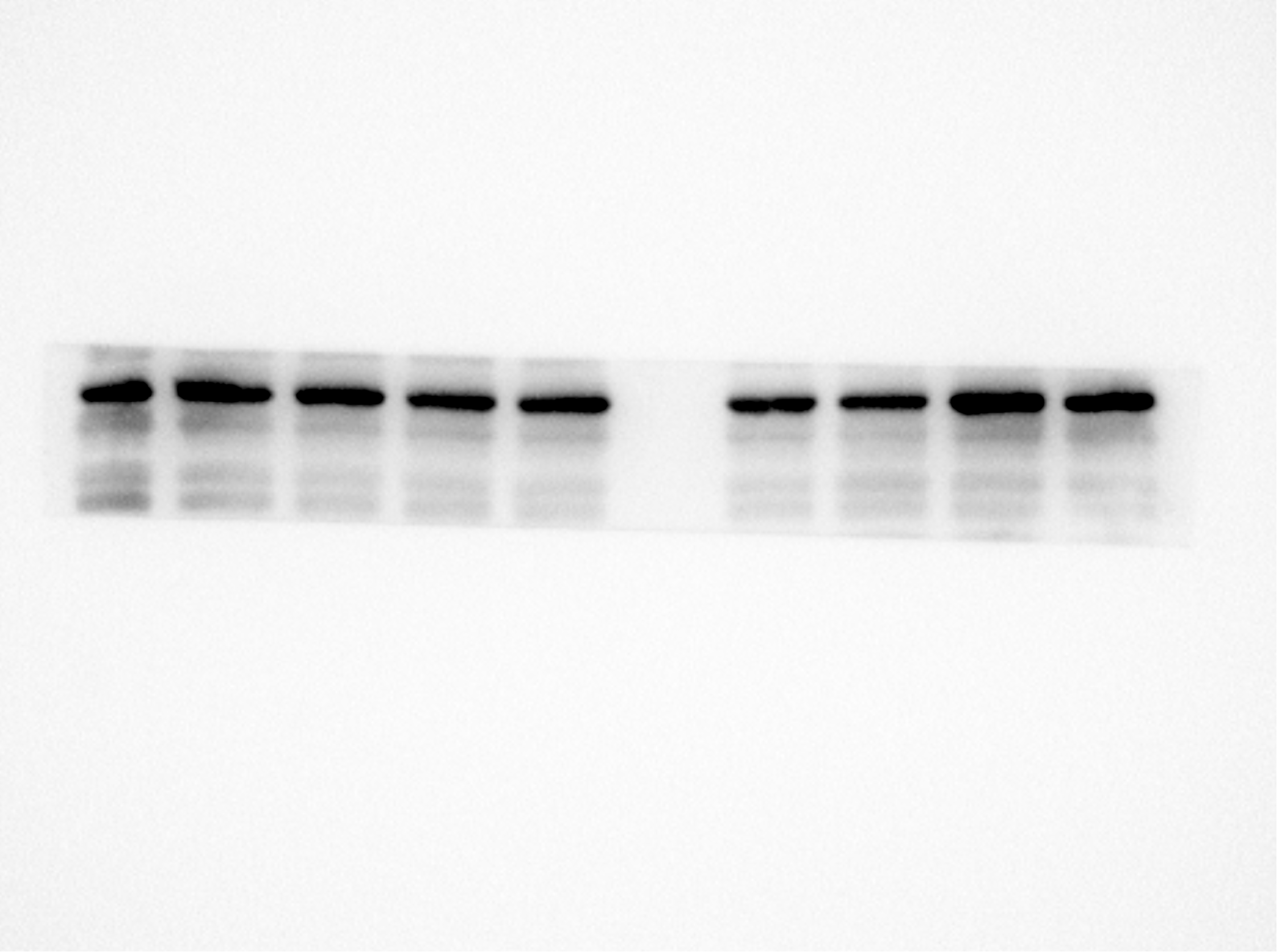

Supplement: Supplementary file 1 [file vetsci-12-00257-s001.zip › PABPC4 original blot images/Fig.4/A/gapdh/shiyantu.tif]

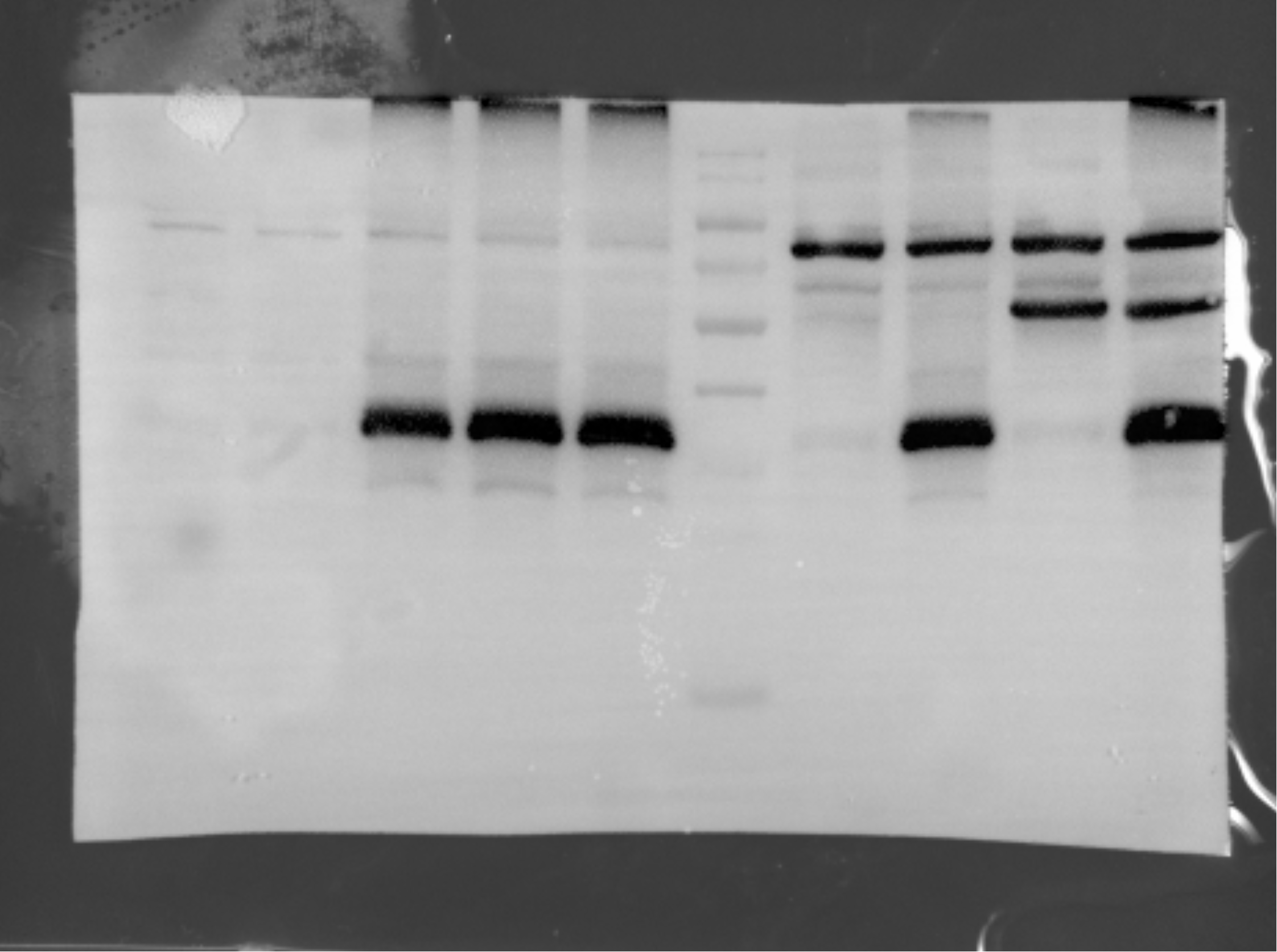

Supplement: Supplementary file 1 [file vetsci-12-00257-s001.zip › PABPC4 original blot images/Fig.4/A/HA/merge.tif]

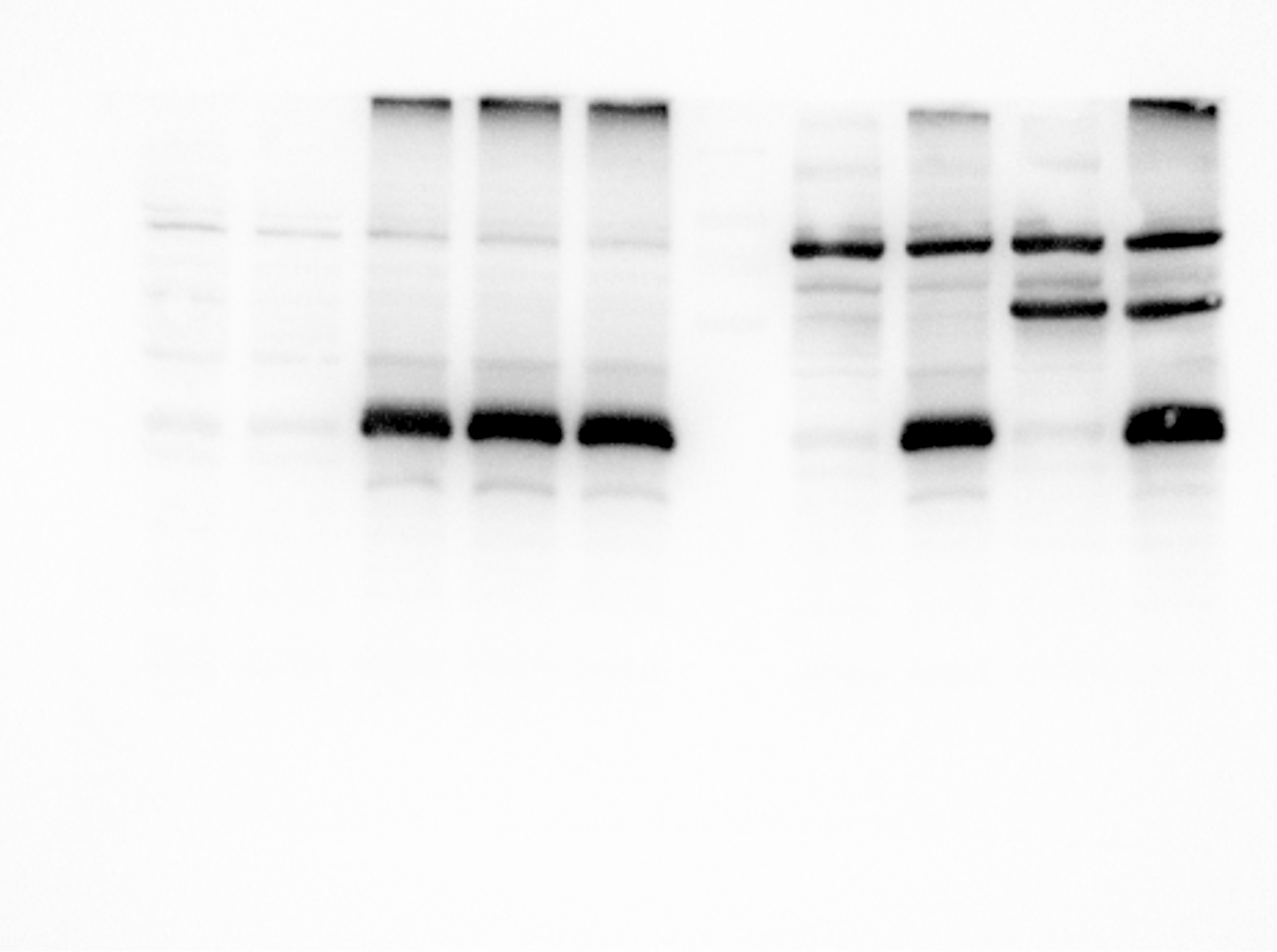

Supplement: Supplementary file 1 [file vetsci-12-00257-s001.zip › PABPC4 original blot images/Fig.4/A/HA/shiyantu.tif]

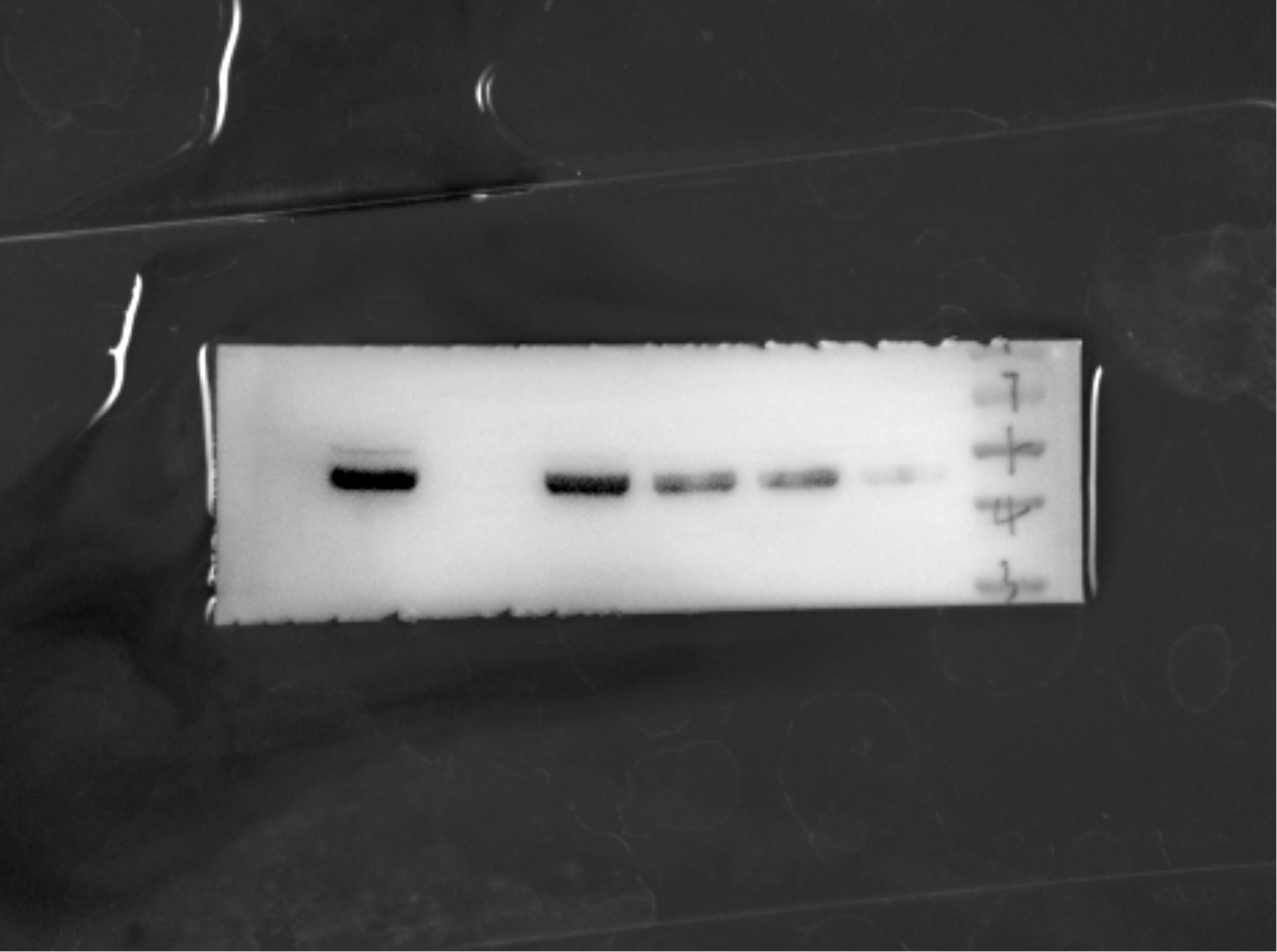

Supplement: Supplementary file 1 [file vetsci-12-00257-s001.zip › PABPC4 original blot images/Fig.4/A/补/flag/h.tif]

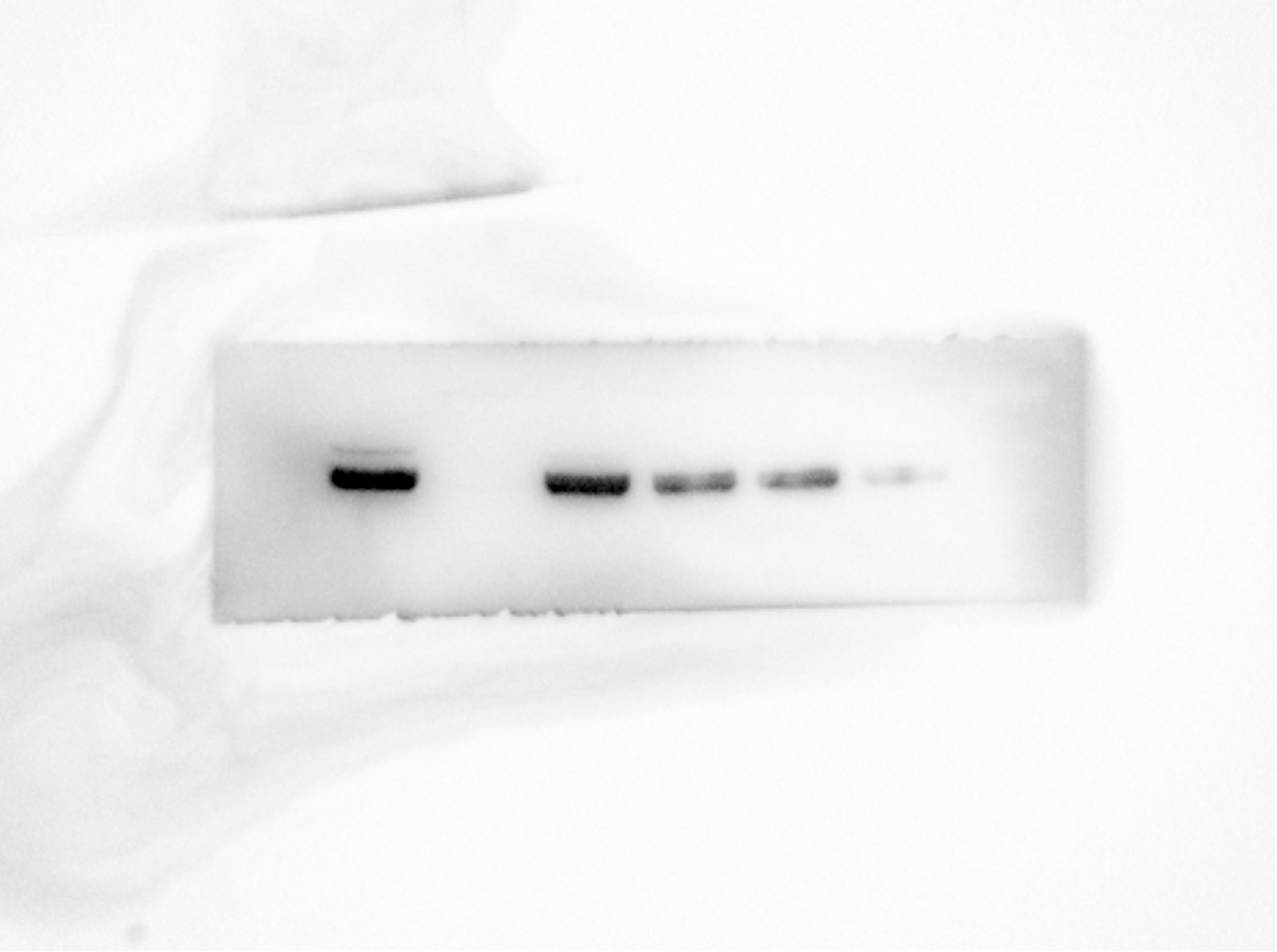

Supplement: Supplementary file 1 [file vetsci-12-00257-s001.zip › PABPC4 original blot images/Fig.4/A/补/flag/s.tif]

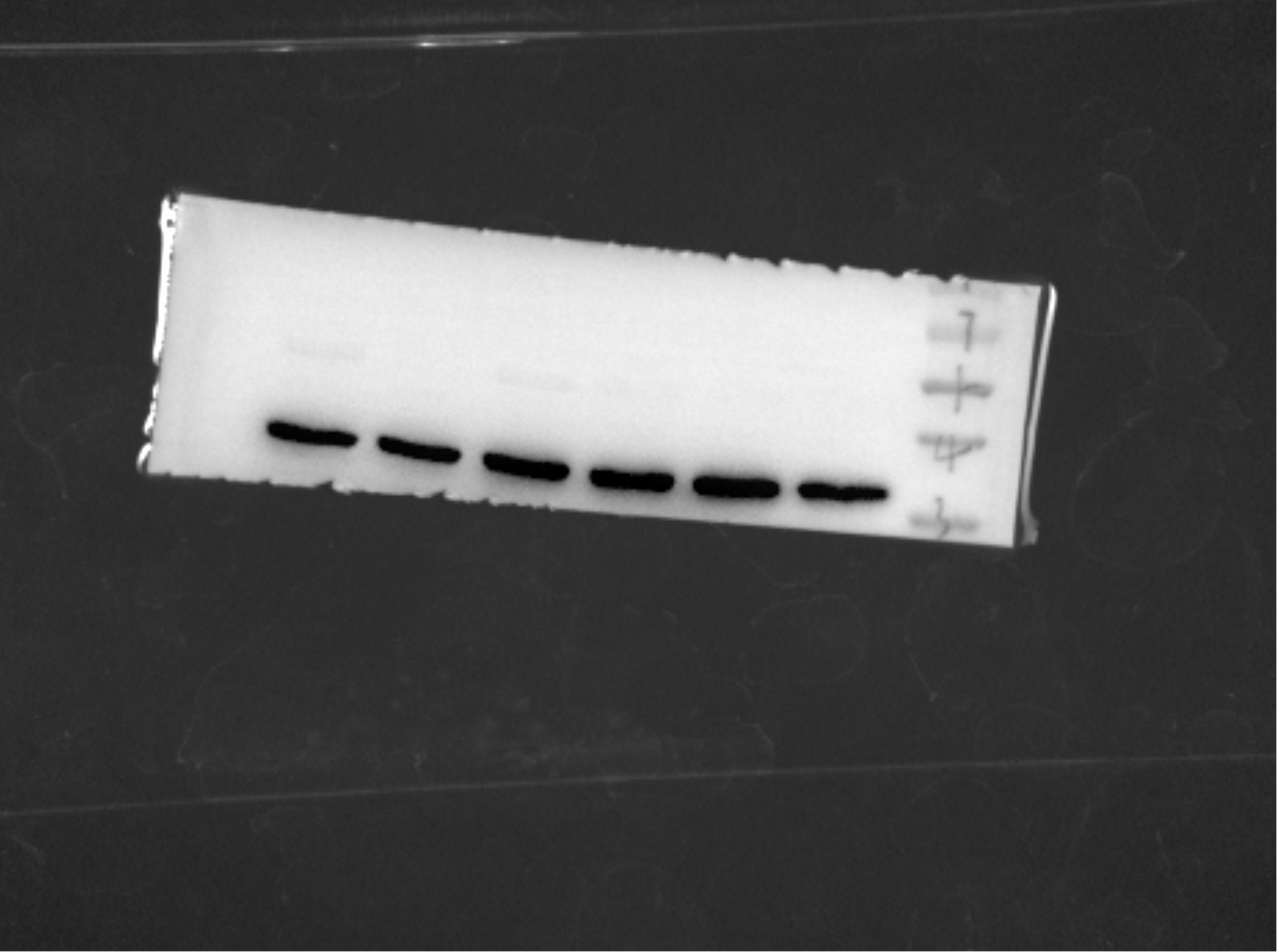

Supplement: Supplementary file 1 [file vetsci-12-00257-s001.zip › PABPC4 original blot images/Fig.4/A/补/gapdh/h.tif]

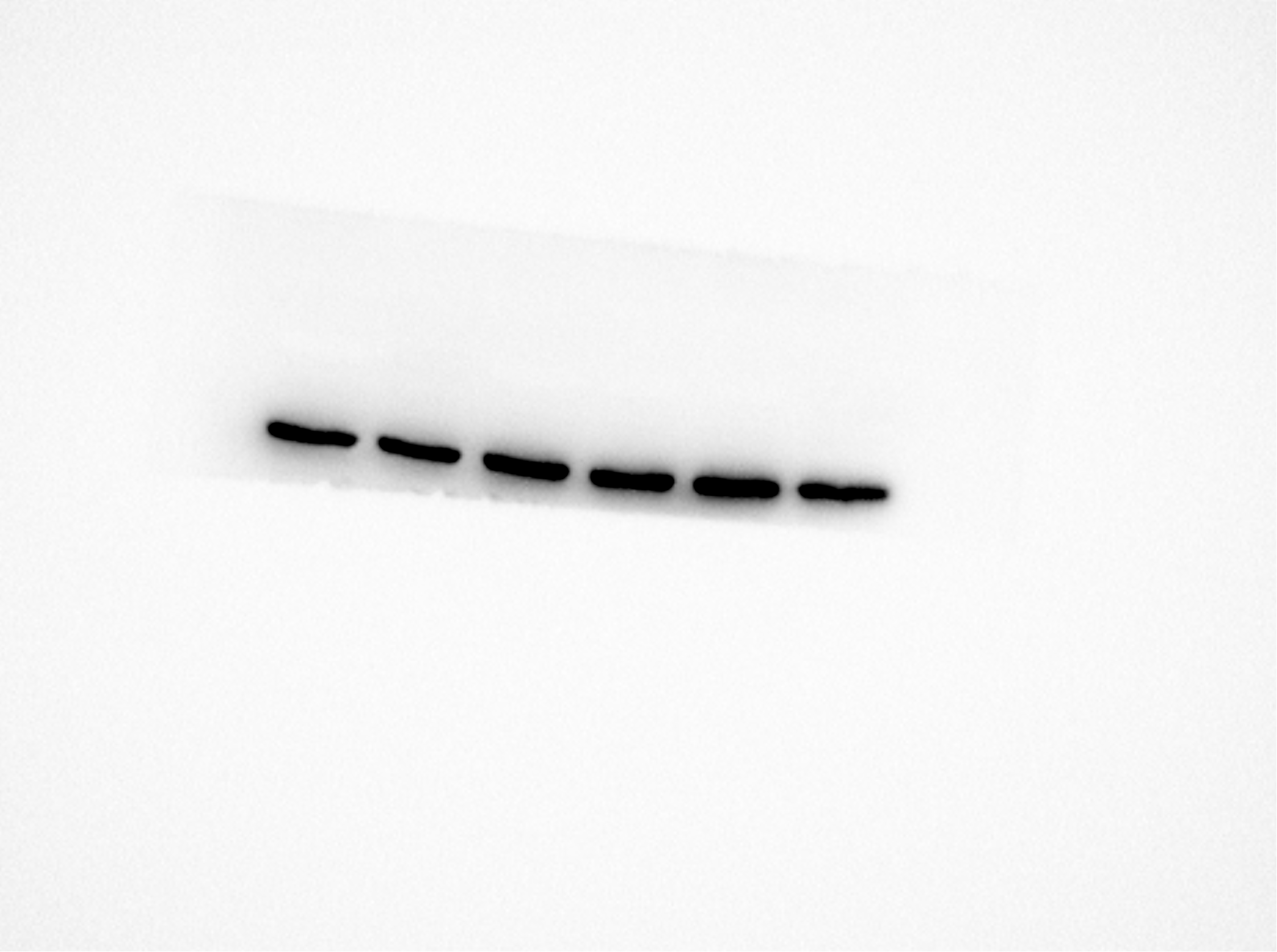

Supplement: Supplementary file 1 [file vetsci-12-00257-s001.zip › PABPC4 original blot images/Fig.4/A/补/gapdh/s.tif]

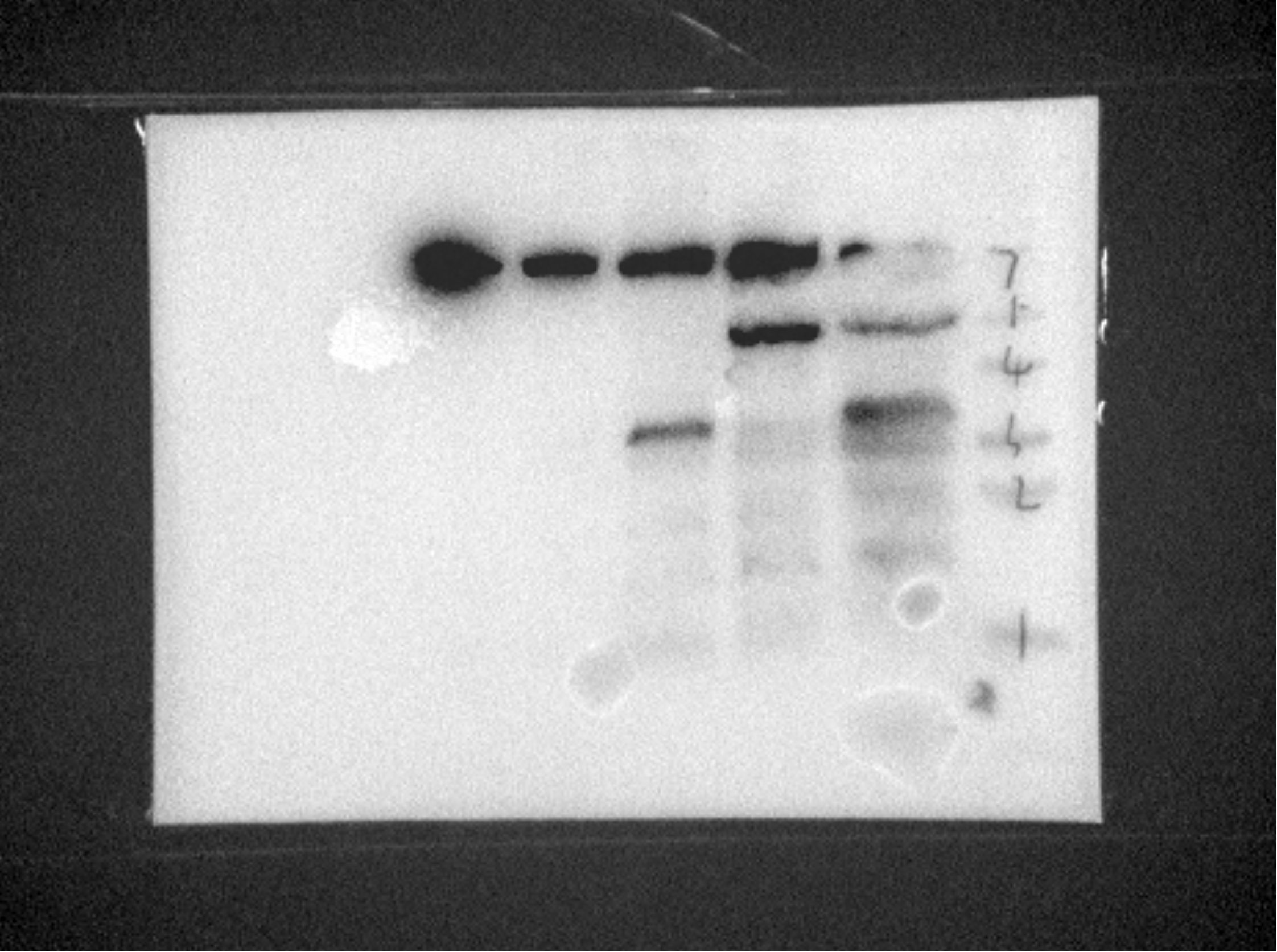

Supplement: Supplementary file 1 [file vetsci-12-00257-s001.zip › PABPC4 original blot images/Fig.4/A/补/HA/H.tif]

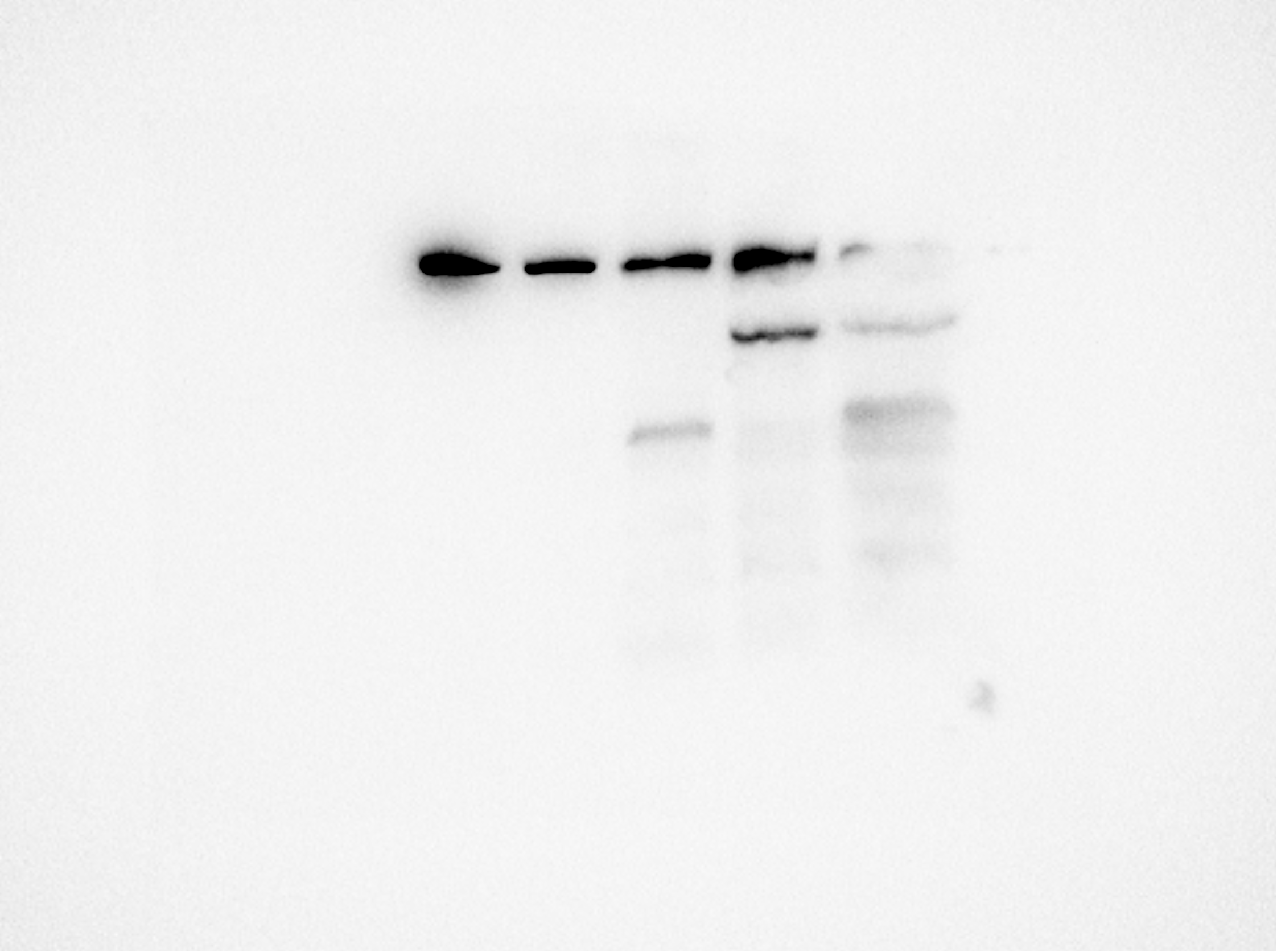

Supplement: Supplementary file 1 [file vetsci-12-00257-s001.zip › PABPC4 original blot images/Fig.4/A/补/HA/S.tif]

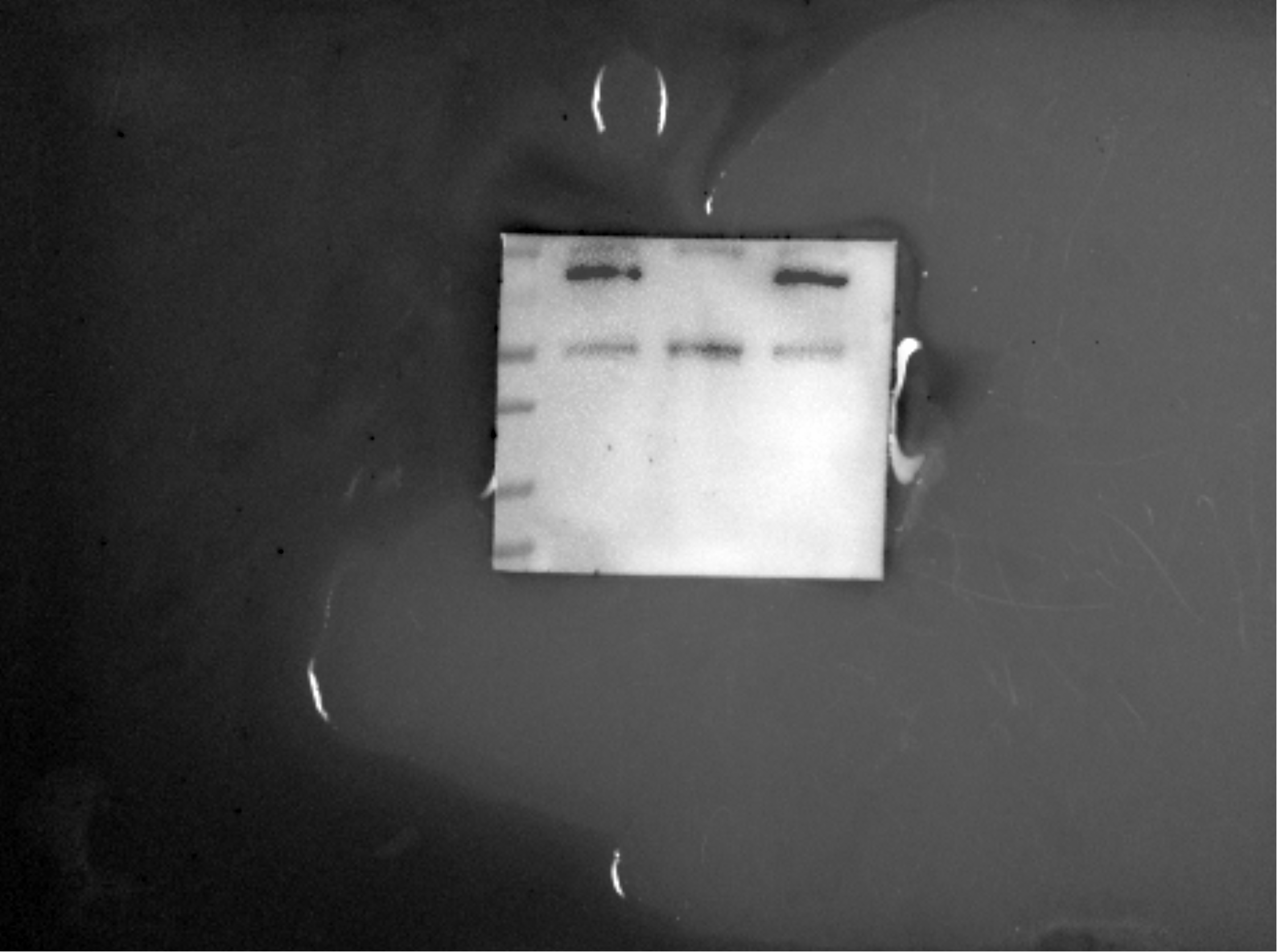

Supplement: Supplementary file 1 [file vetsci-12-00257-s001.zip › PABPC4 original blot images/Fig.4/B/IB/FLAG-FLAG/h.tif]

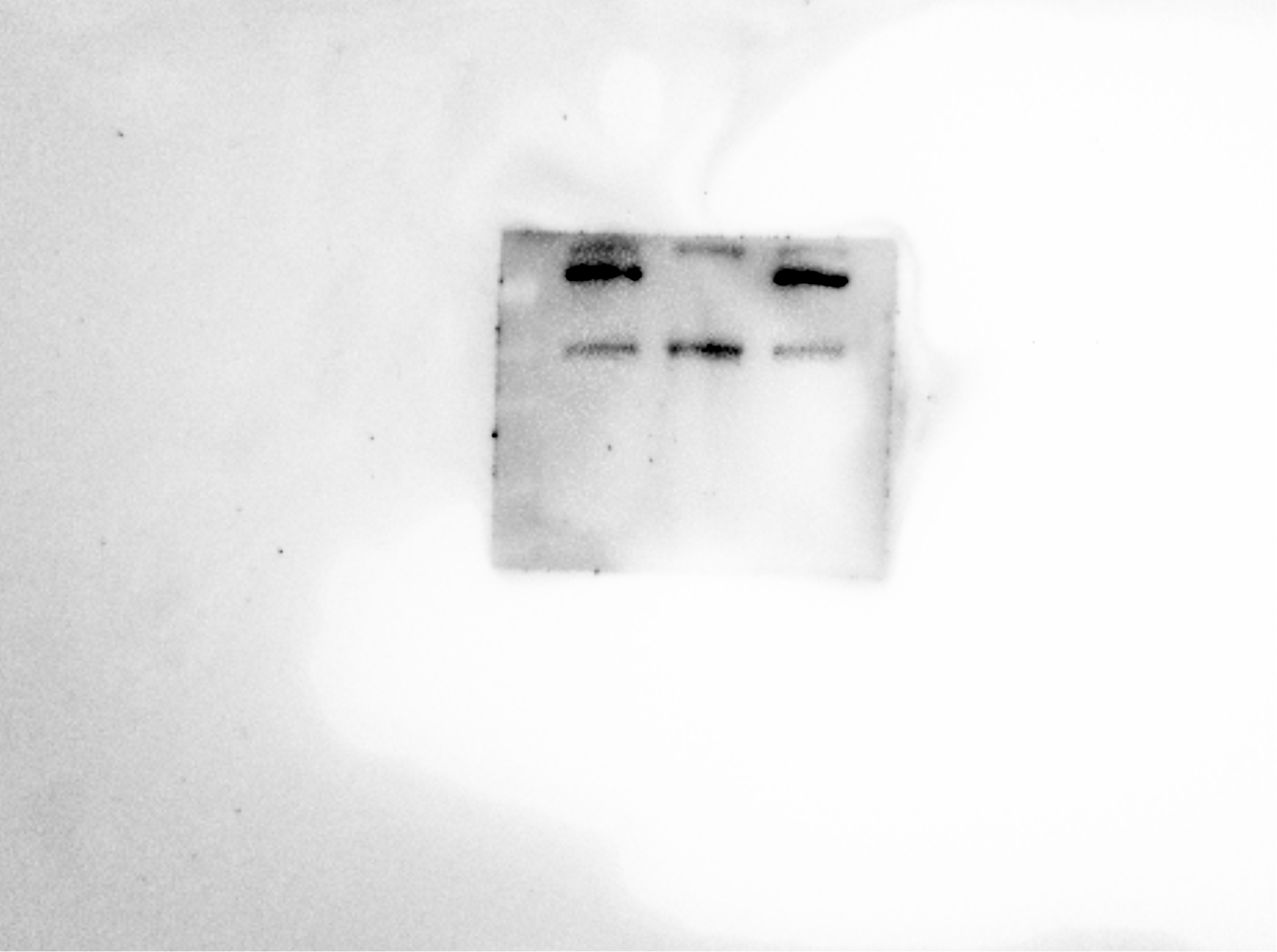

Supplement: Supplementary file 1 [file vetsci-12-00257-s001.zip › PABPC4 original blot images/Fig.4/B/IB/FLAG-FLAG/s.tif]

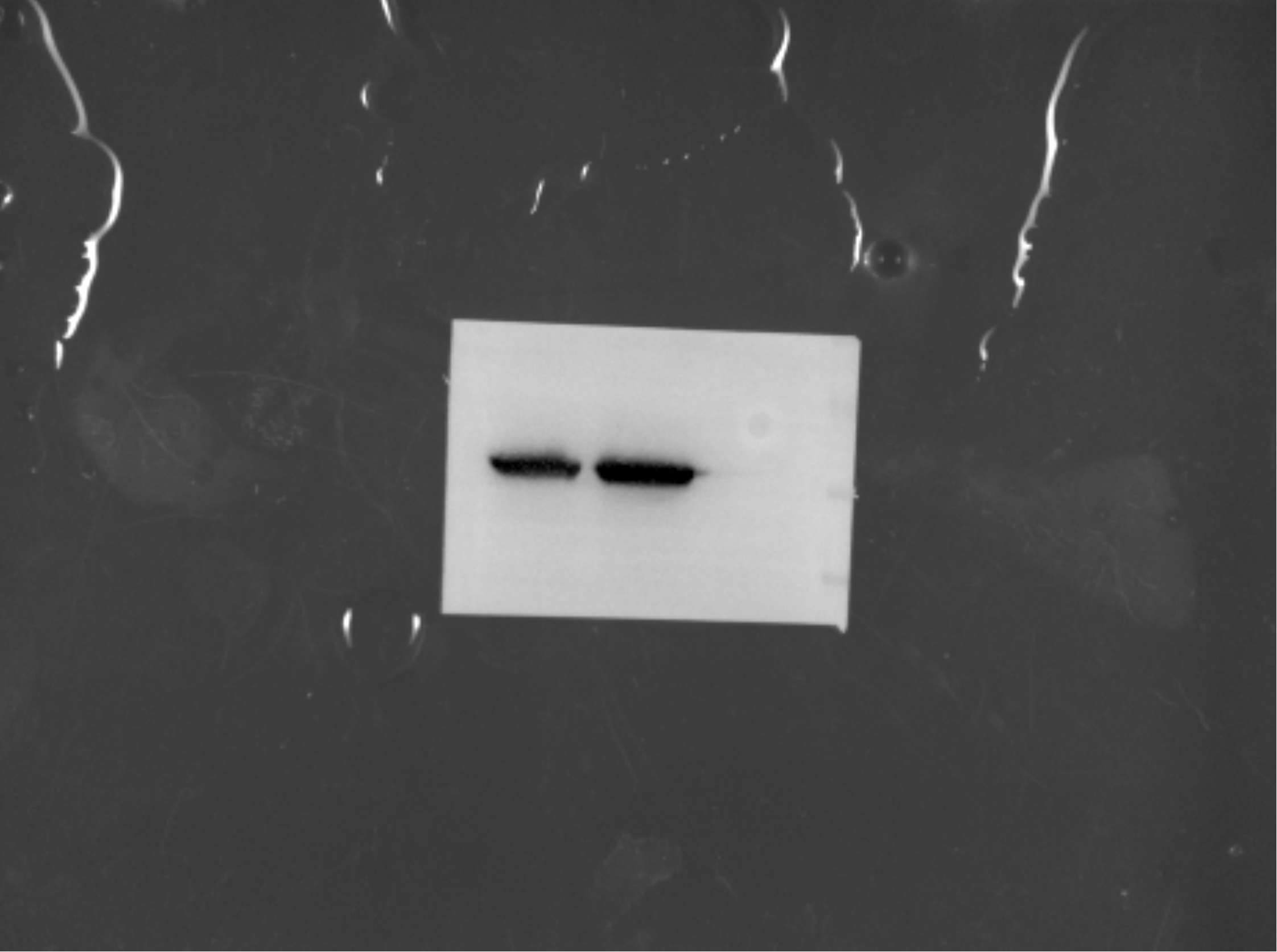

Supplement: Supplementary file 1 [file vetsci-12-00257-s001.zip › PABPC4 original blot images/Fig.4/B/IB/FLAG-HA/H.tif]

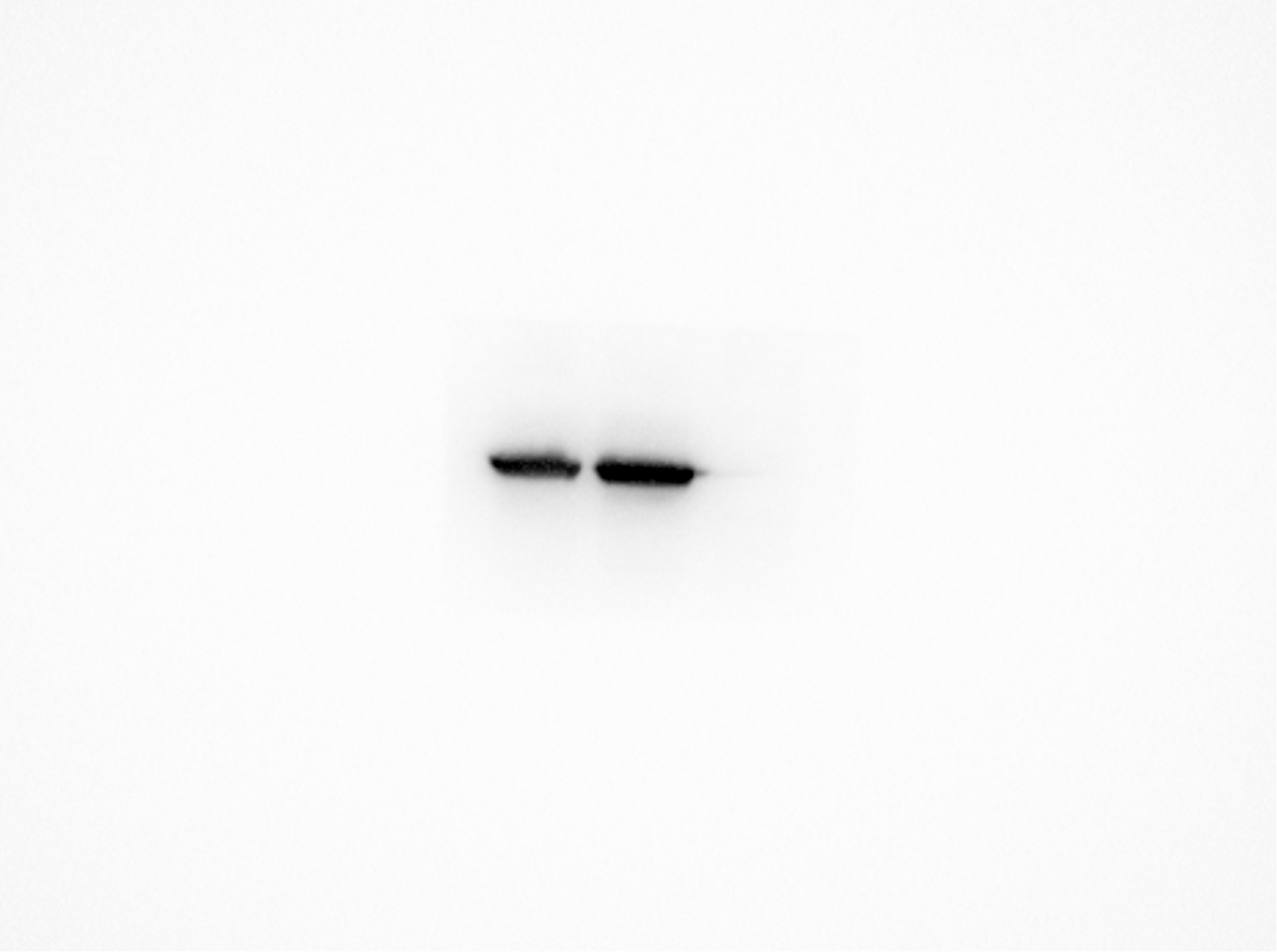

Supplement: Supplementary file 1 [file vetsci-12-00257-s001.zip › PABPC4 original blot images/Fig.4/B/IB/FLAG-HA/S.tif]

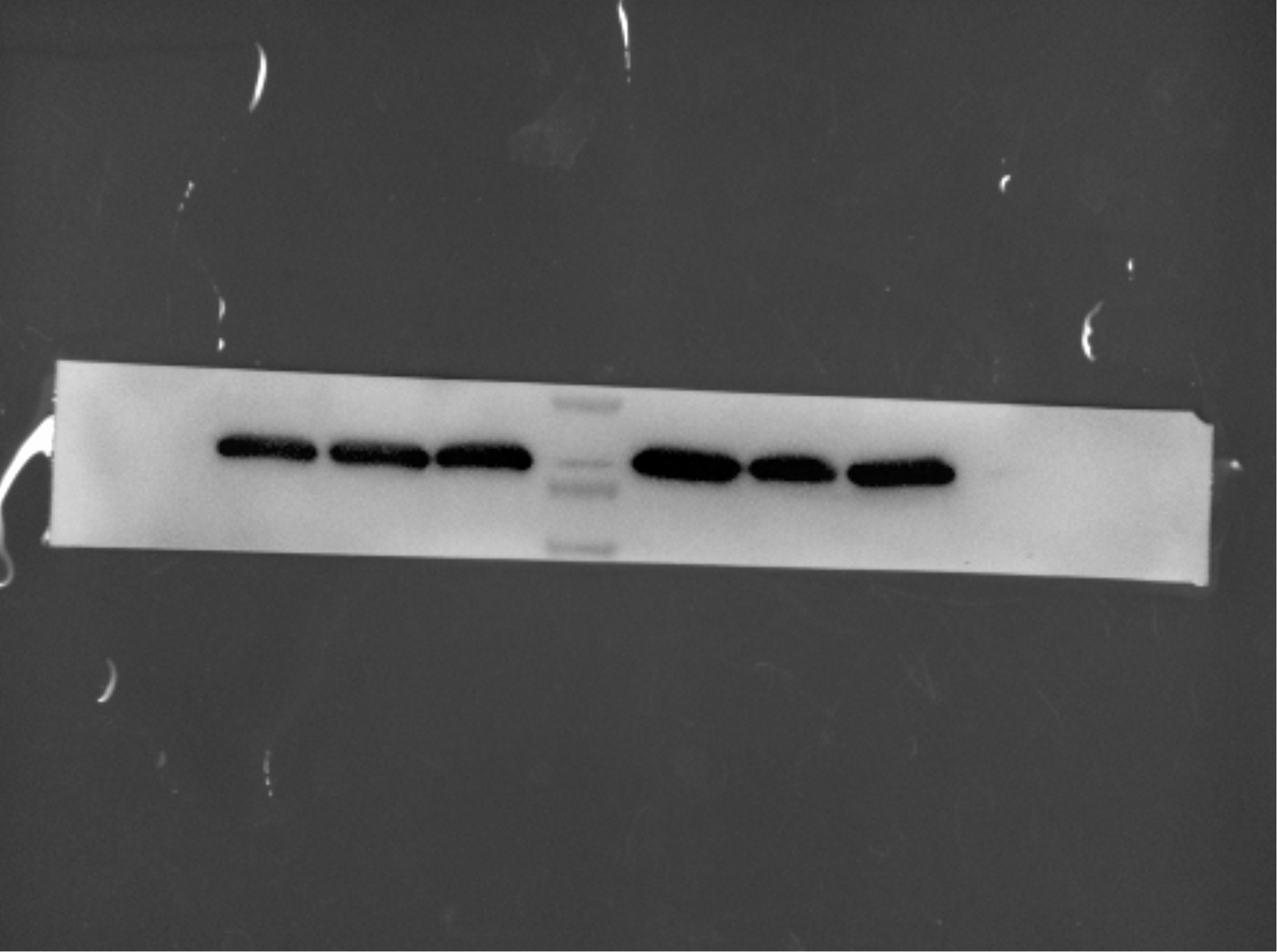

Supplement: Supplementary file 1 [file vetsci-12-00257-s001.zip › PABPC4 original blot images/Fig.4/B/IB/gapdh/merge.tif]

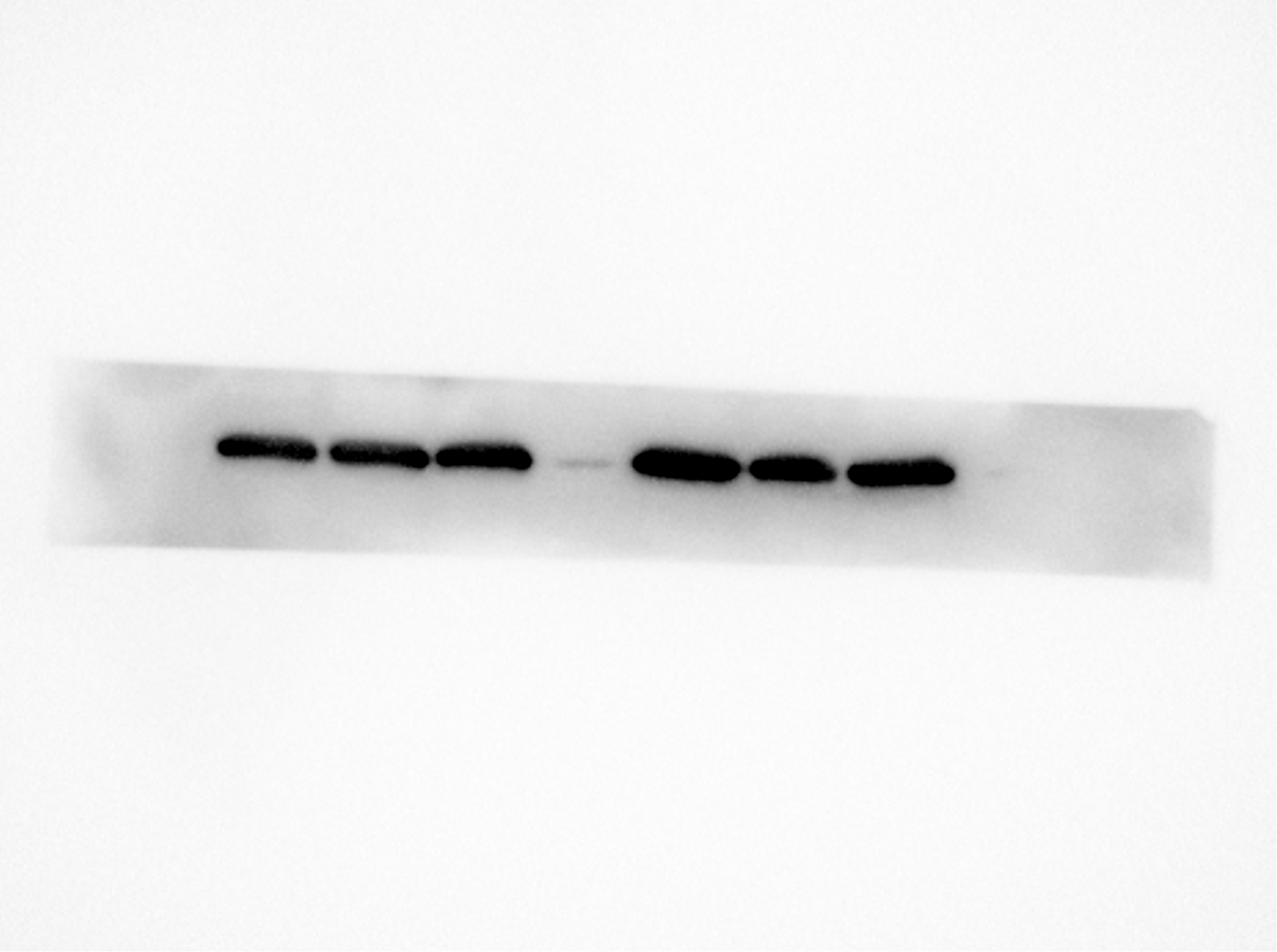

Supplement: Supplementary file 1 [file vetsci-12-00257-s001.zip › PABPC4 original blot images/Fig.4/B/IB/gapdh/s.tif]

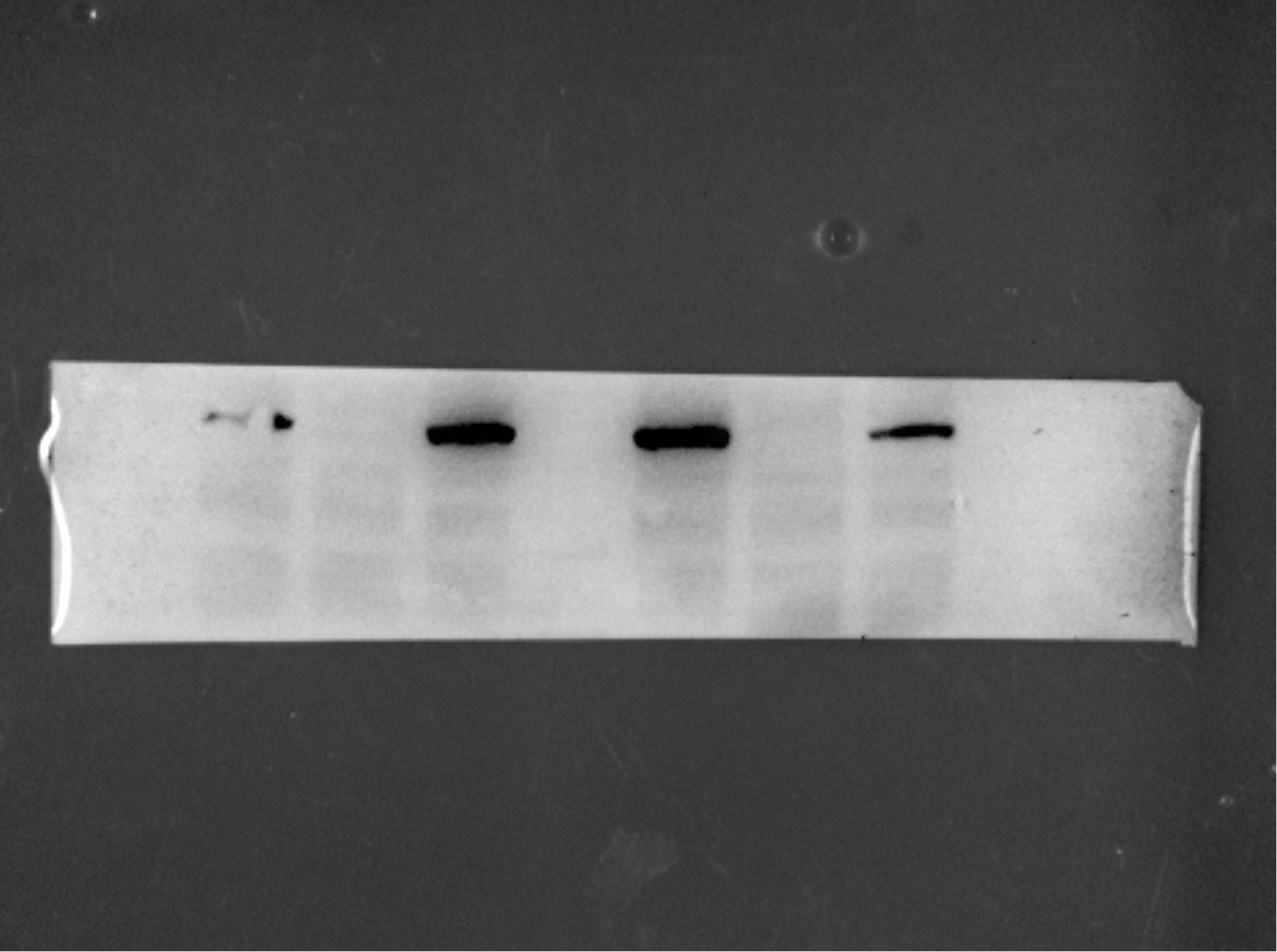

Supplement: Supplementary file 1 [file vetsci-12-00257-s001.zip › PABPC4 original blot images/Fig.4/B/IB/HA-FLAG/H.tif]

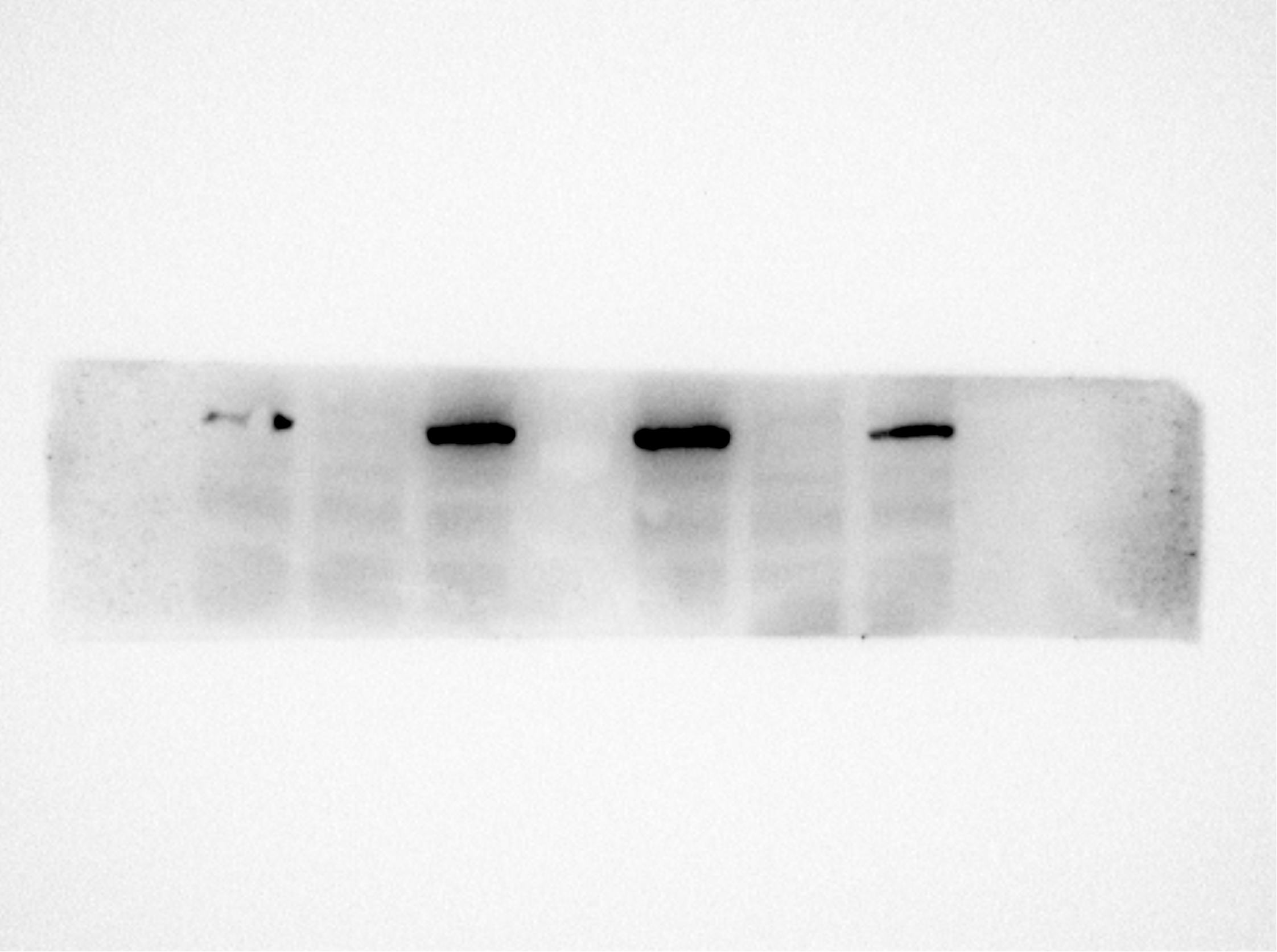

Supplement: Supplementary file 1 [file vetsci-12-00257-s001.zip › PABPC4 original blot images/Fig.4/B/IB/HA-FLAG/S.tif]

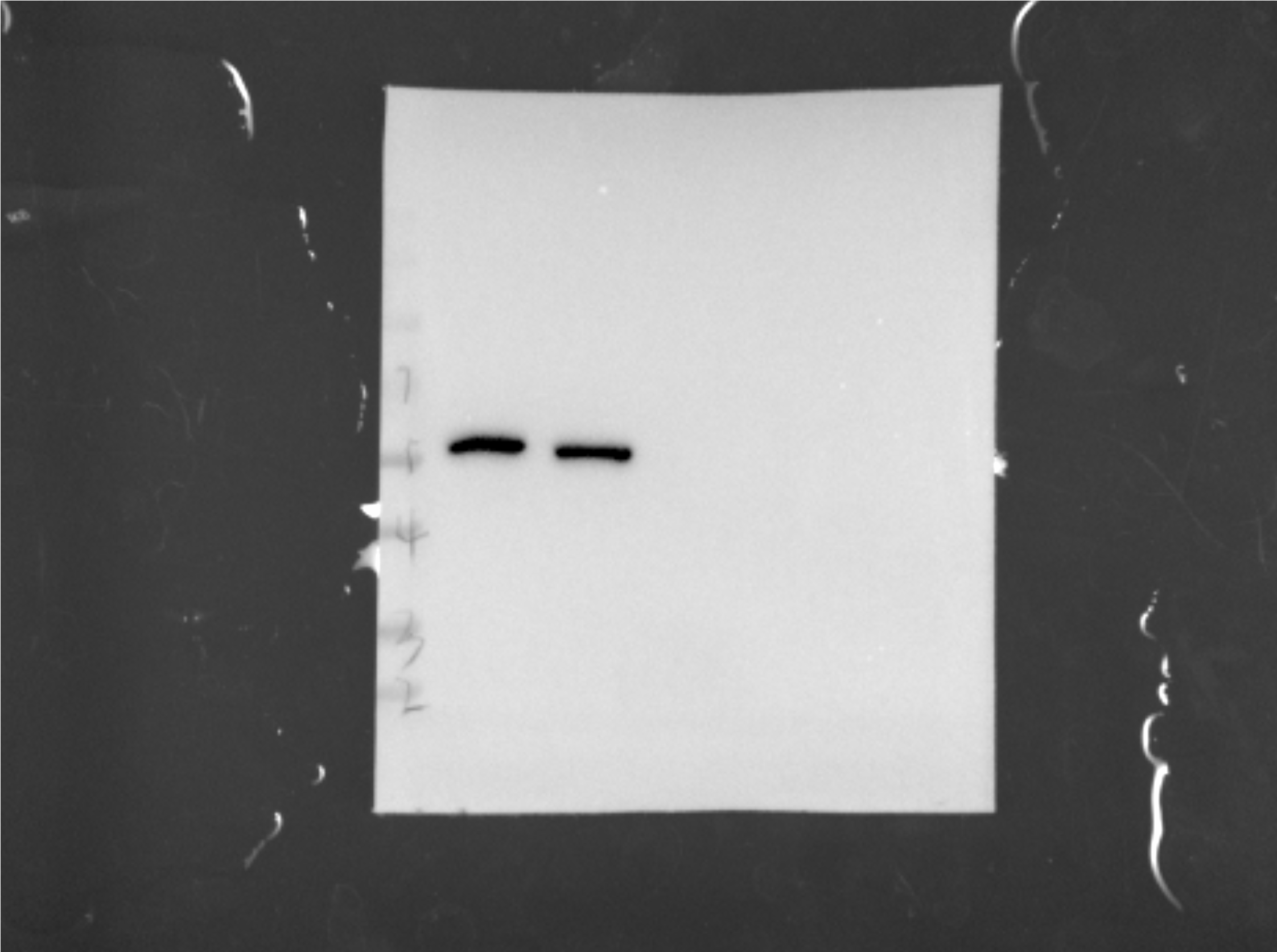

Supplement: Supplementary file 1 [file vetsci-12-00257-s001.zip › PABPC4 original blot images/Fig.4/B/IB/HA-HA/H.tif]

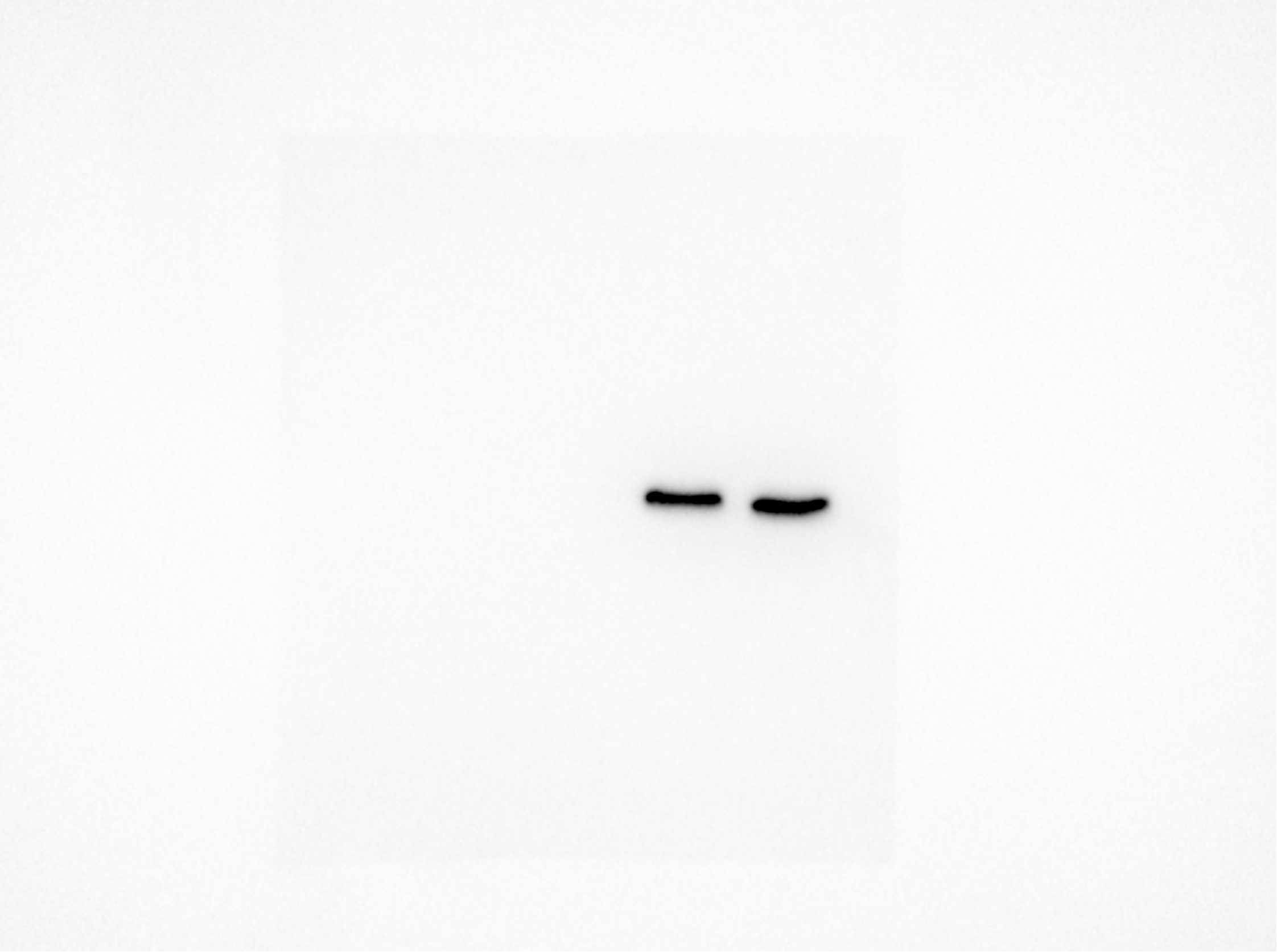

Supplement: Supplementary file 1 [file vetsci-12-00257-s001.zip › PABPC4 original blot images/Fig.4/B/IB/HA-HA/S.tif]

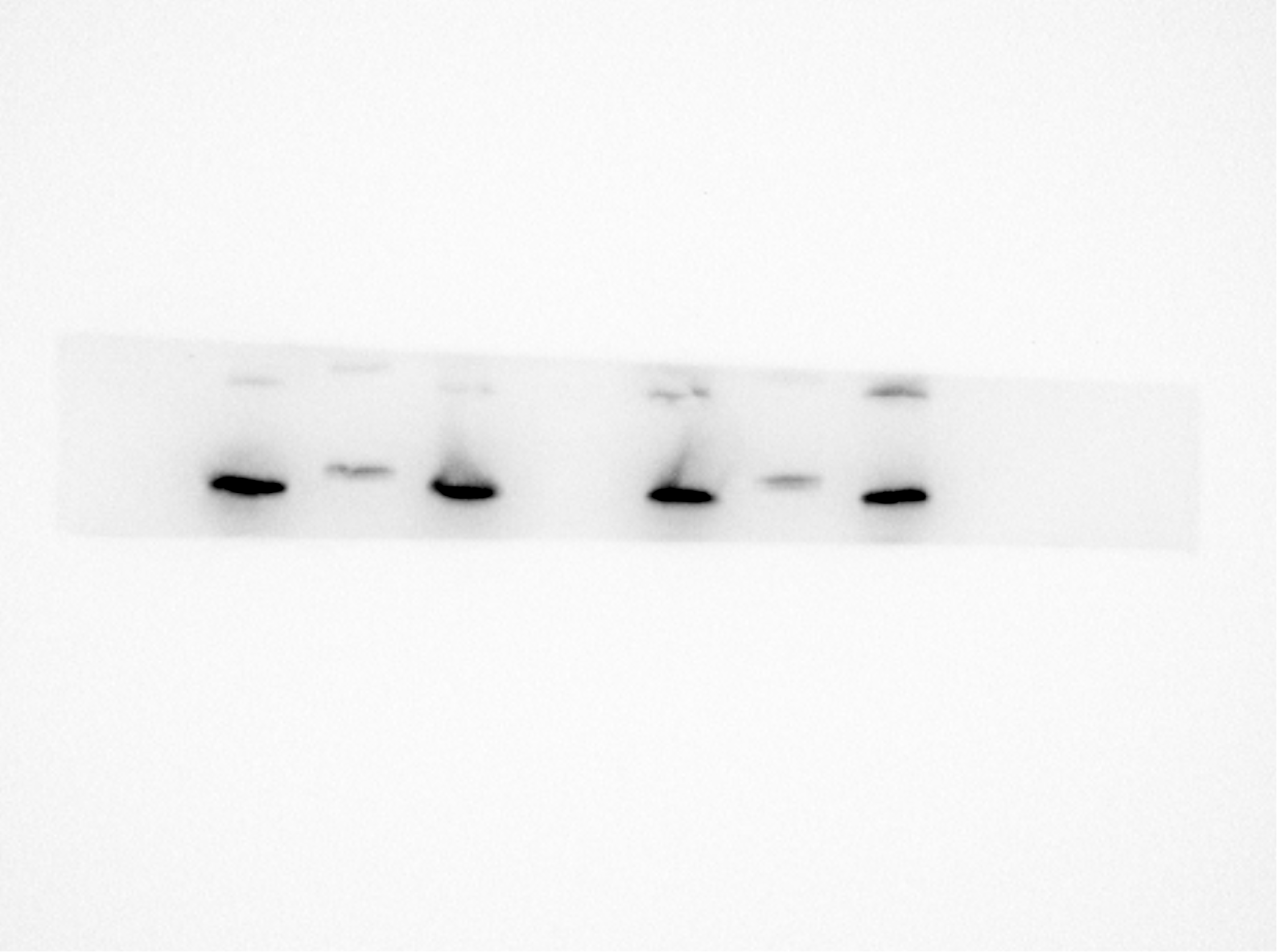

Supplement: Supplementary file 1 [file vetsci-12-00257-s001.zip › PABPC4 original blot images/Fig.4/B/ip-flag/flag/Administrator 2023-08-22_21h02m27s.tif]

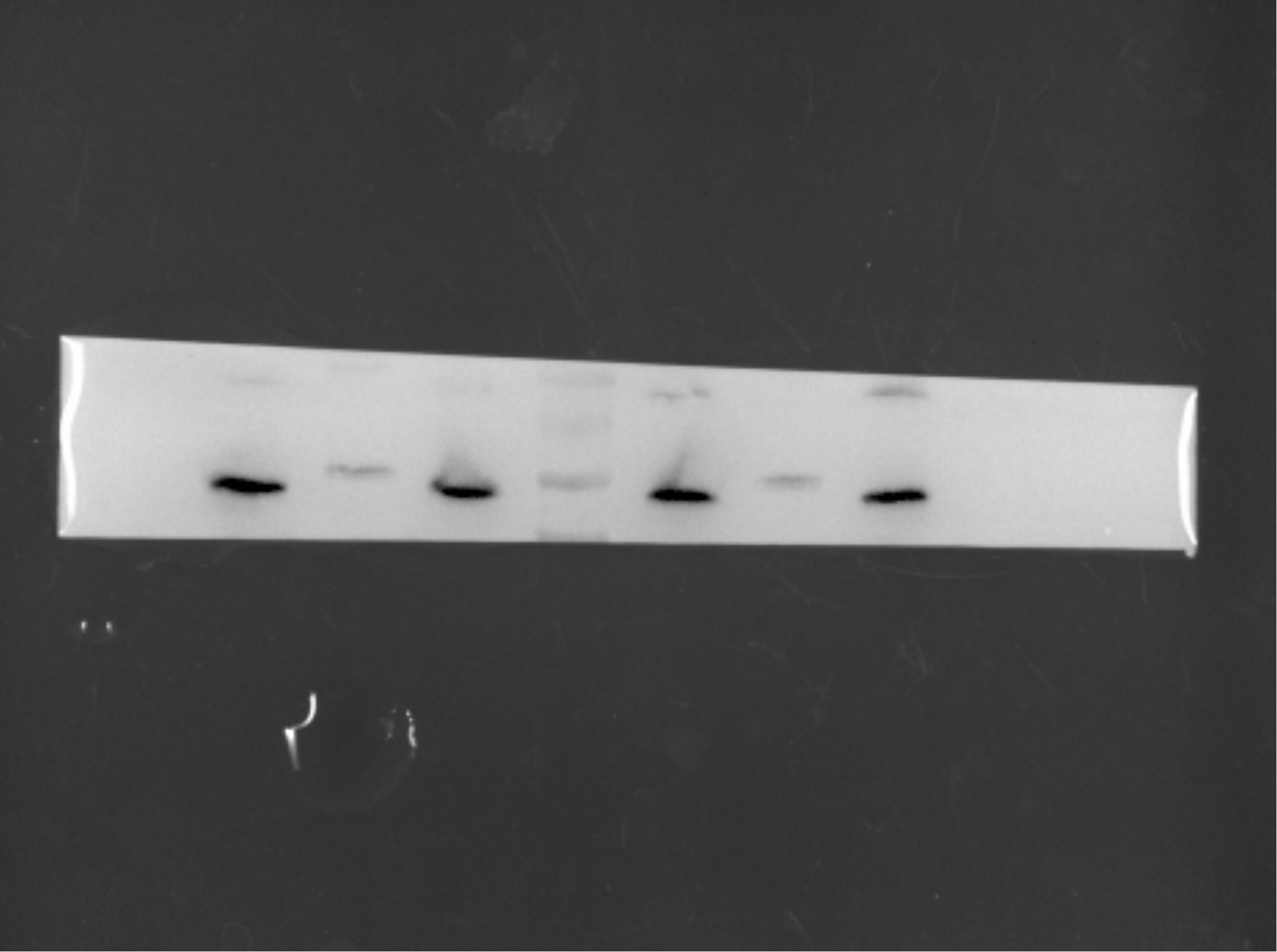

Supplement: Supplementary file 1 [file vetsci-12-00257-s001.zip › PABPC4 original blot images/Fig.4/B/ip-flag/flag/Administrator 2023-08-22_21h03m31s+Administrator 2023-08-22_21h02m27s.tif]

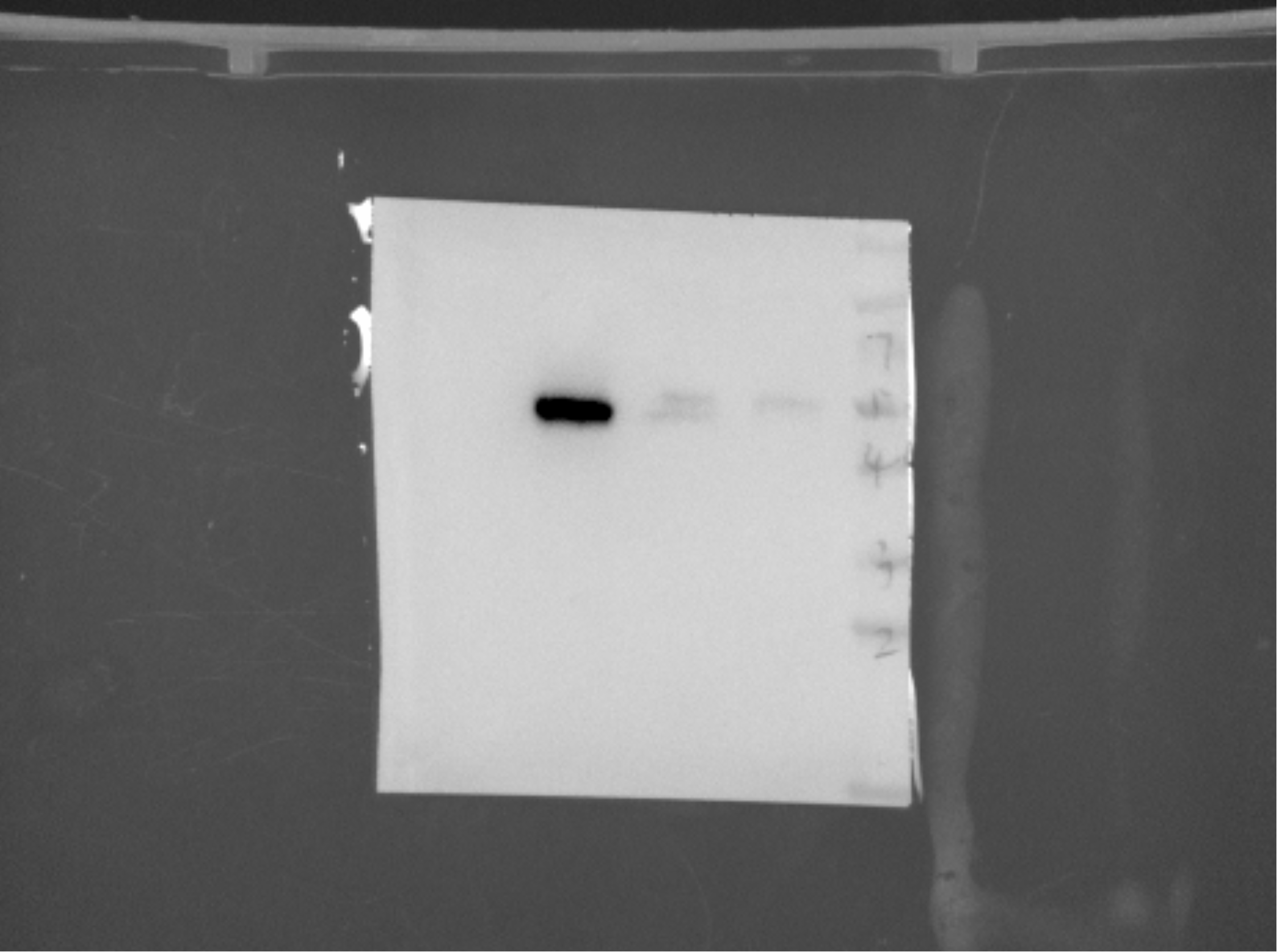

Supplement: Supplementary file 1 [file vetsci-12-00257-s001.zip › PABPC4 original blot images/Fig.4/B/ip-flag/ha/h.tif]

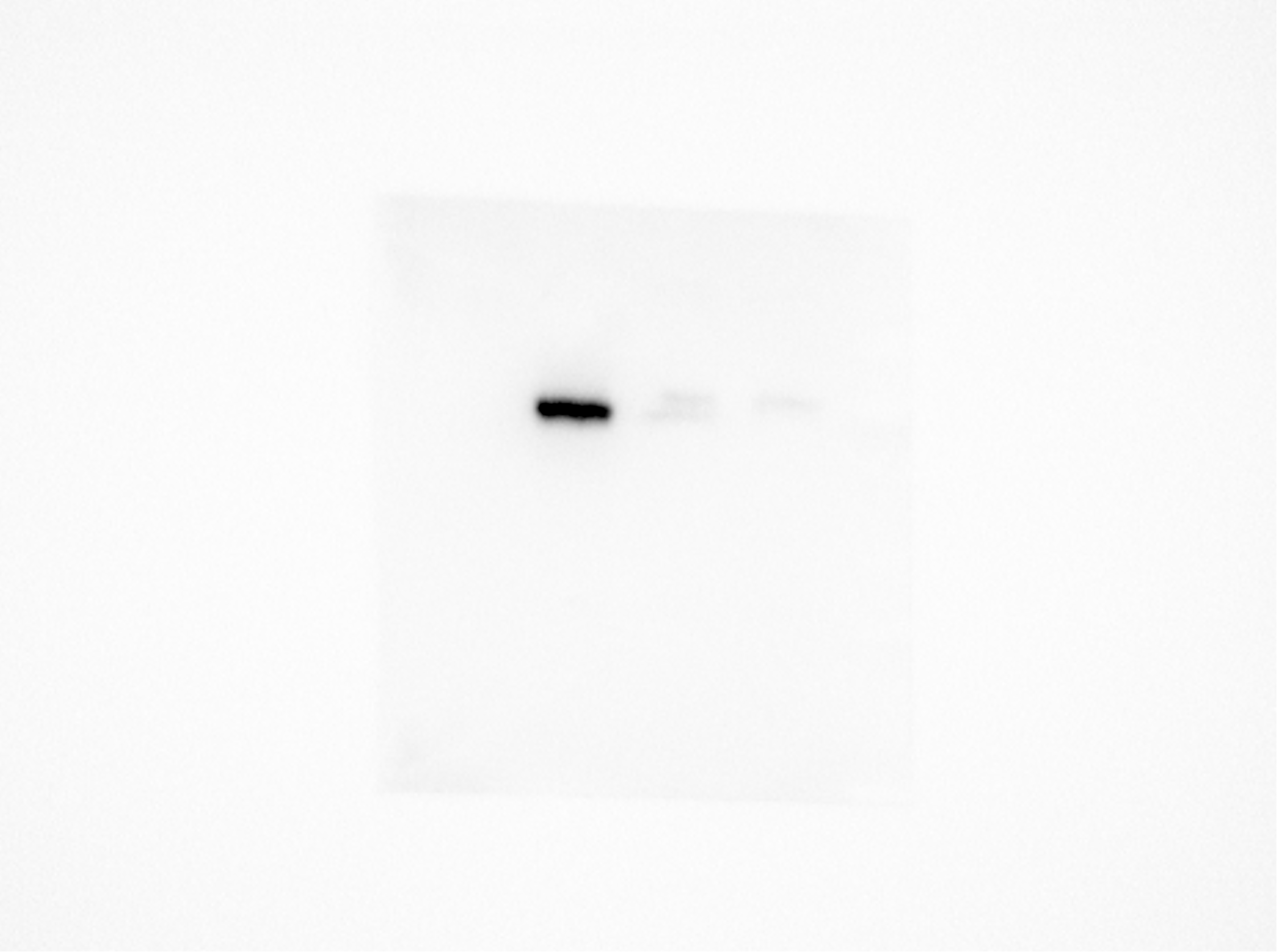

Supplement: Supplementary file 1 [file vetsci-12-00257-s001.zip › PABPC4 original blot images/Fig.4/B/ip-flag/ha/s.tif]

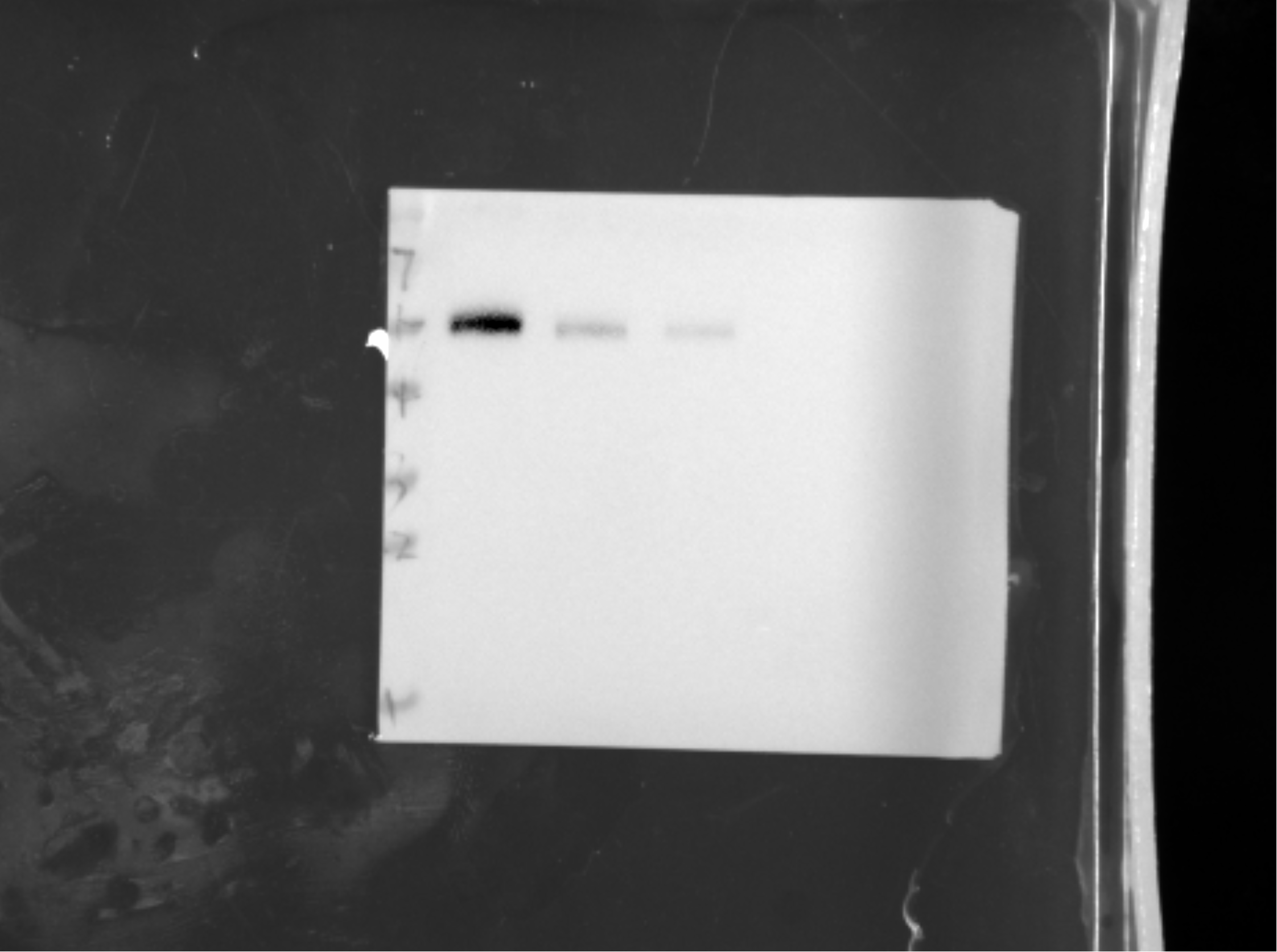

Supplement: Supplementary file 1 [file vetsci-12-00257-s001.zip › PABPC4 original blot images/Fig.4/B/ip-ha/flag/h.tif]

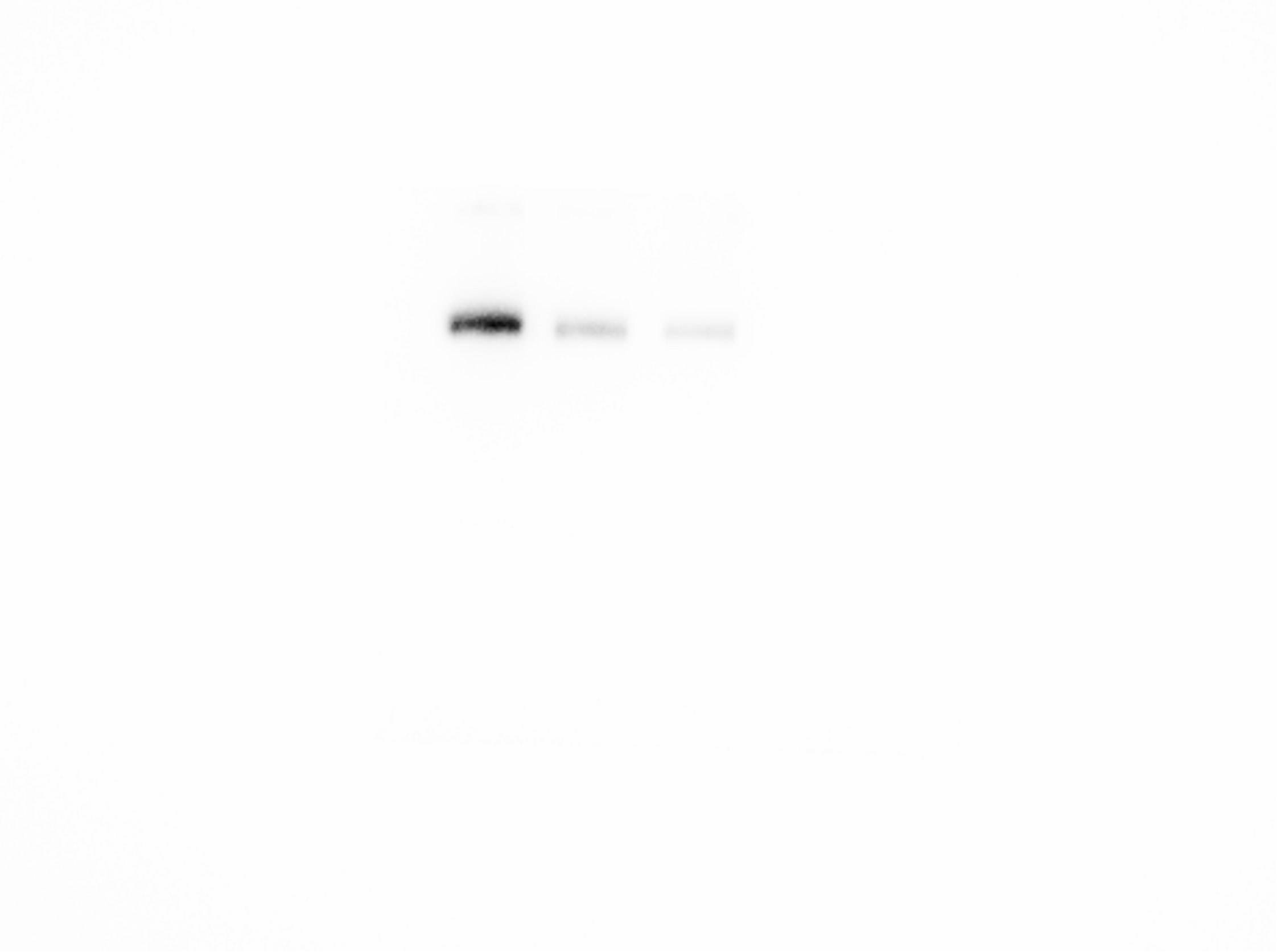

Supplement: Supplementary file 1 [file vetsci-12-00257-s001.zip › PABPC4 original blot images/Fig.4/B/ip-ha/flag/s.tif]

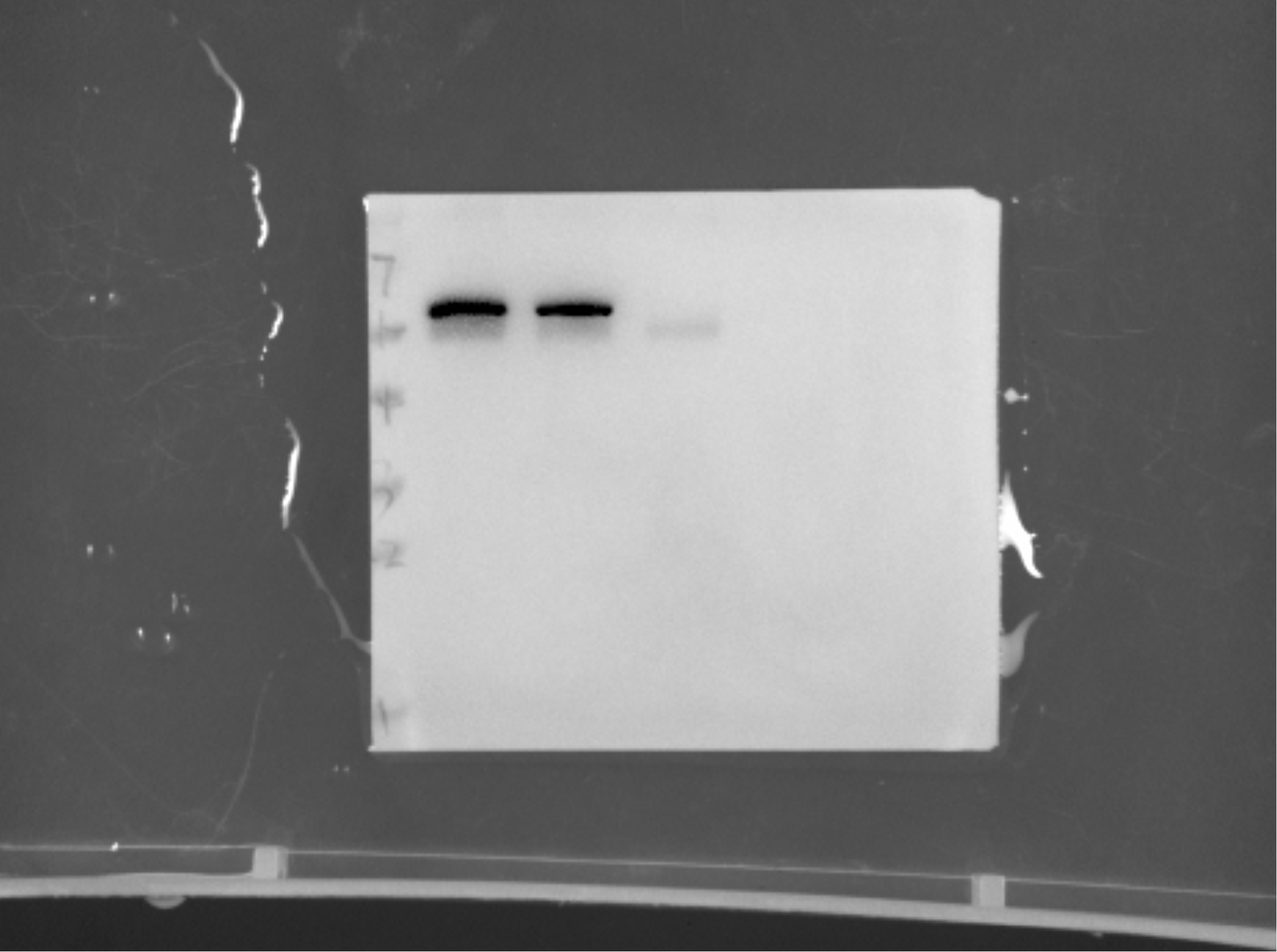

Supplement: Supplementary file 1 [file vetsci-12-00257-s001.zip › PABPC4 original blot images/Fig.4/B/ip-ha/ha/h.tif]

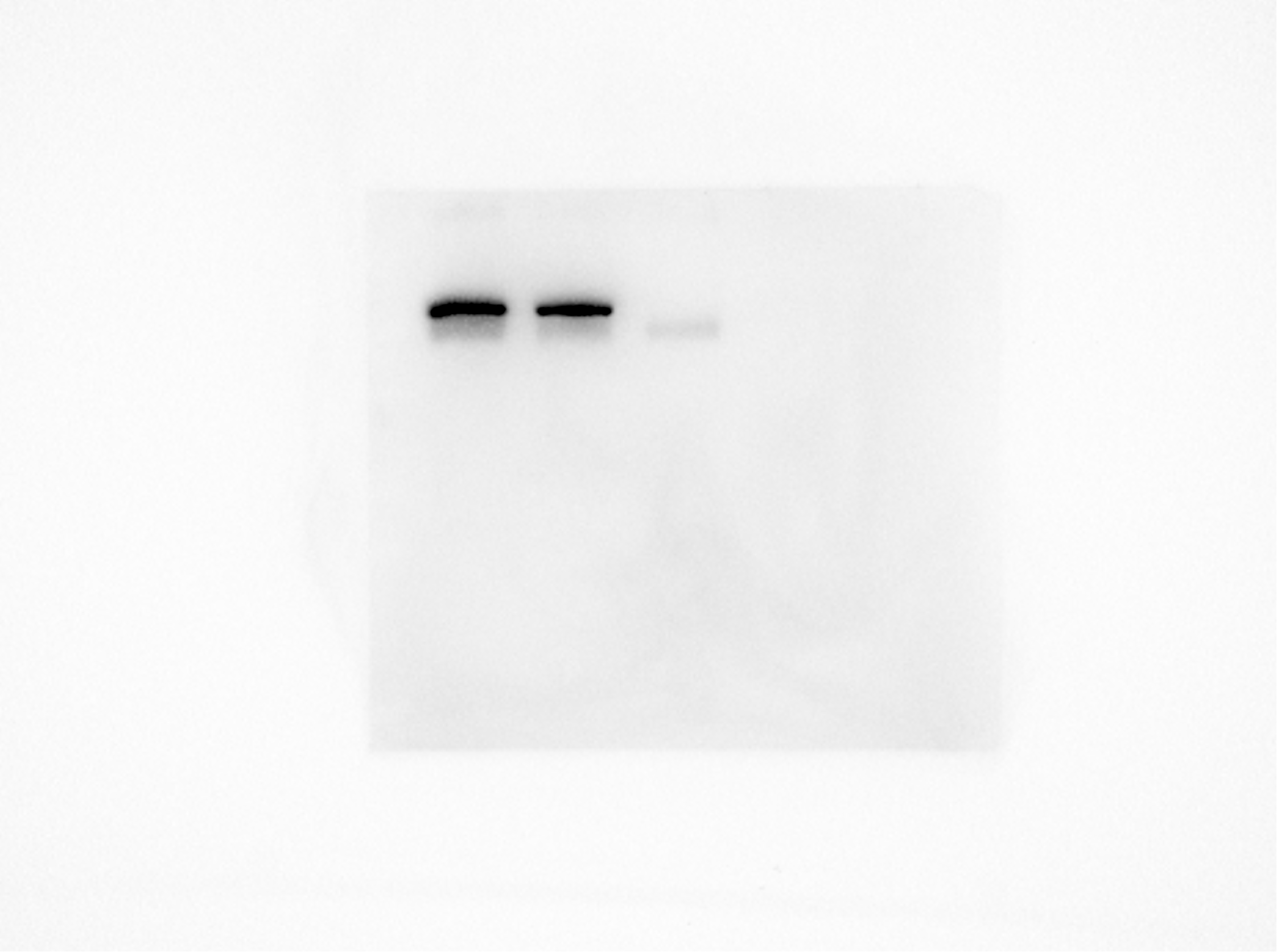

Supplement: Supplementary file 1 [file vetsci-12-00257-s001.zip › PABPC4 original blot images/Fig.4/B/ip-ha/ha/s.tif]

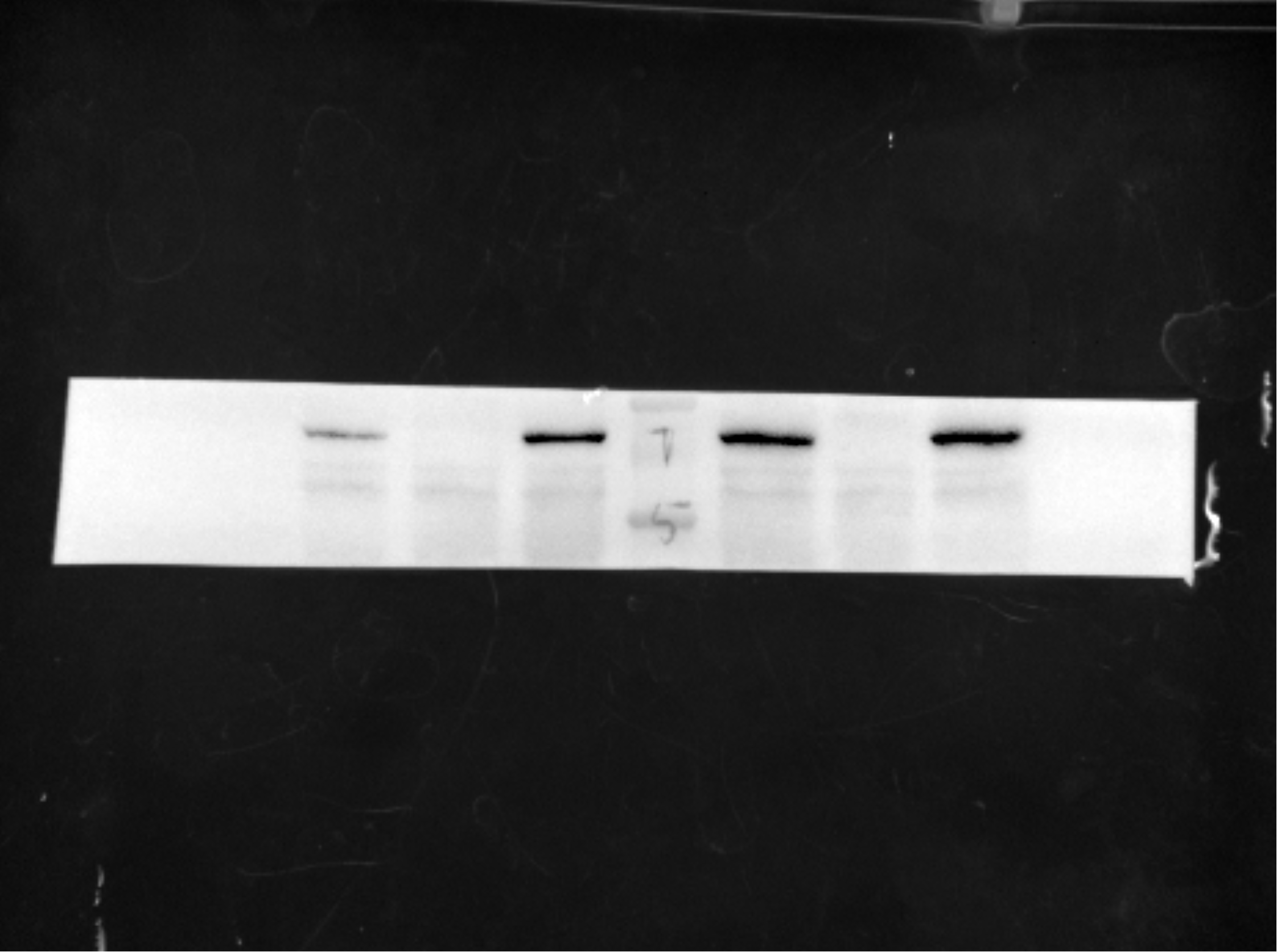

Supplement: Supplementary file 1 [file vetsci-12-00257-s001.zip › PABPC4 original blot images/Fig.4/C/IB/flag/h.tif]

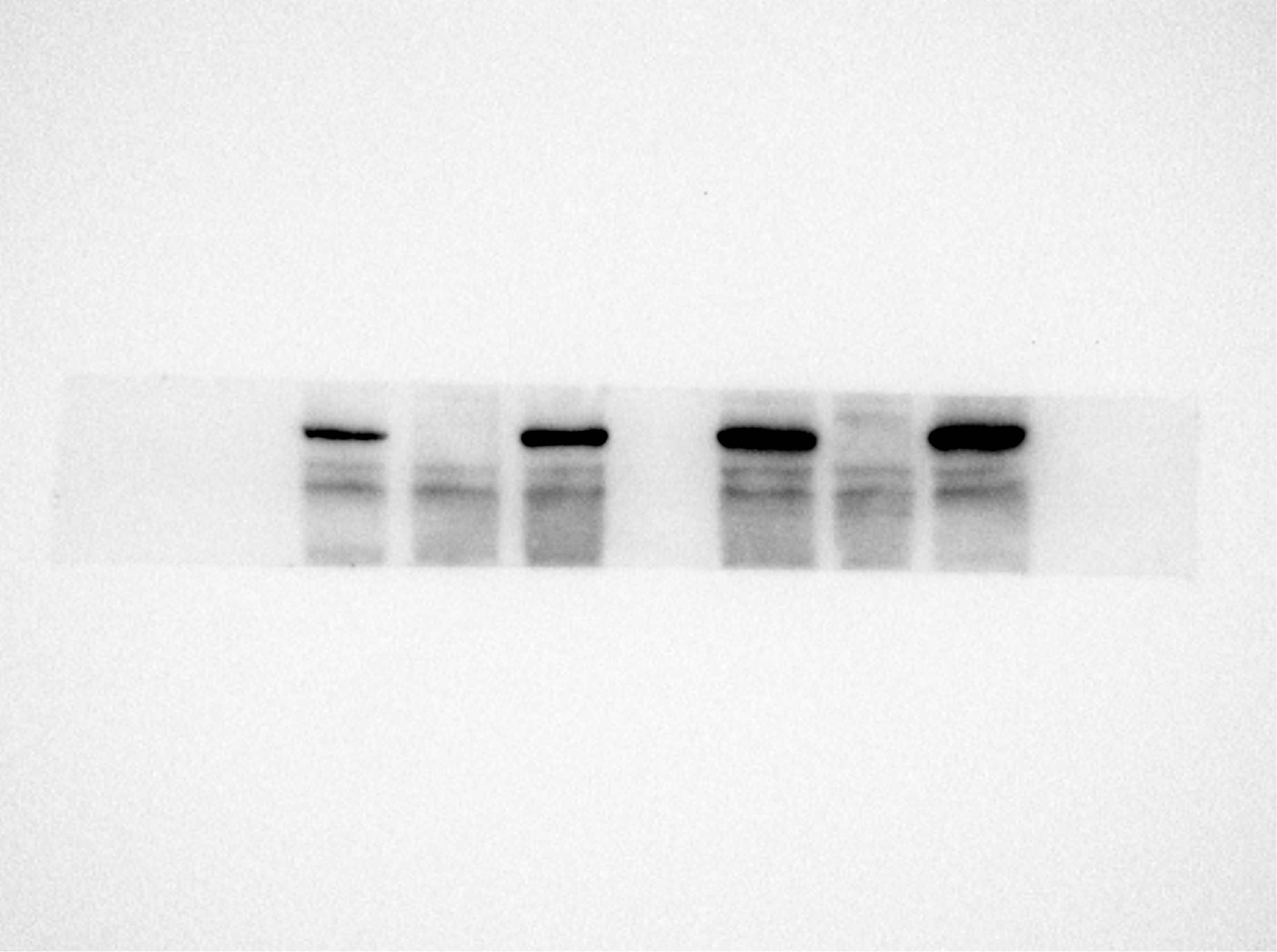

Supplement: Supplementary file 1 [file vetsci-12-00257-s001.zip › PABPC4 original blot images/Fig.4/C/IB/flag/s.tif]

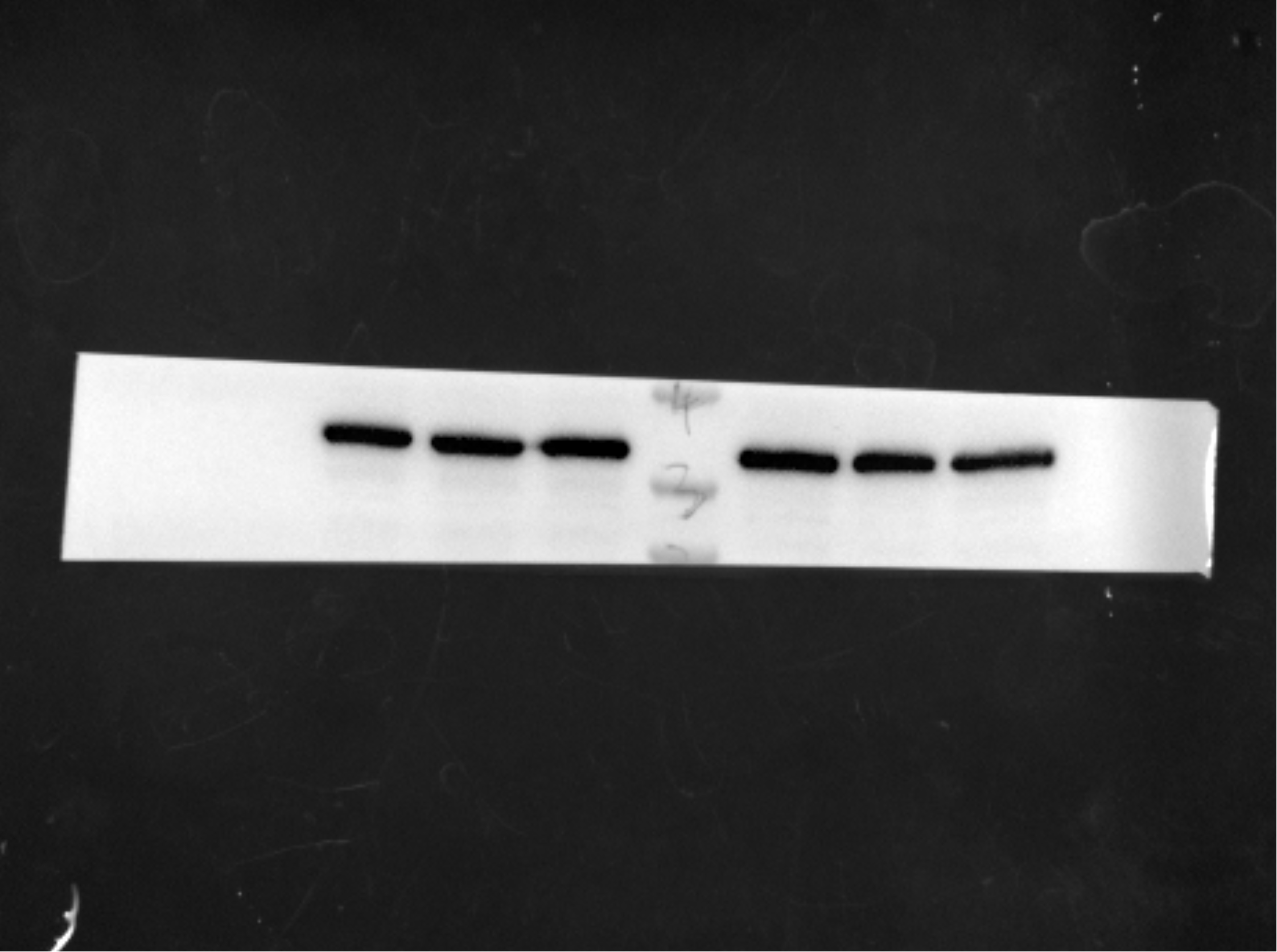

Supplement: Supplementary file 1 [file vetsci-12-00257-s001.zip › PABPC4 original blot images/Fig.4/C/IB/gapdh/h.tif]

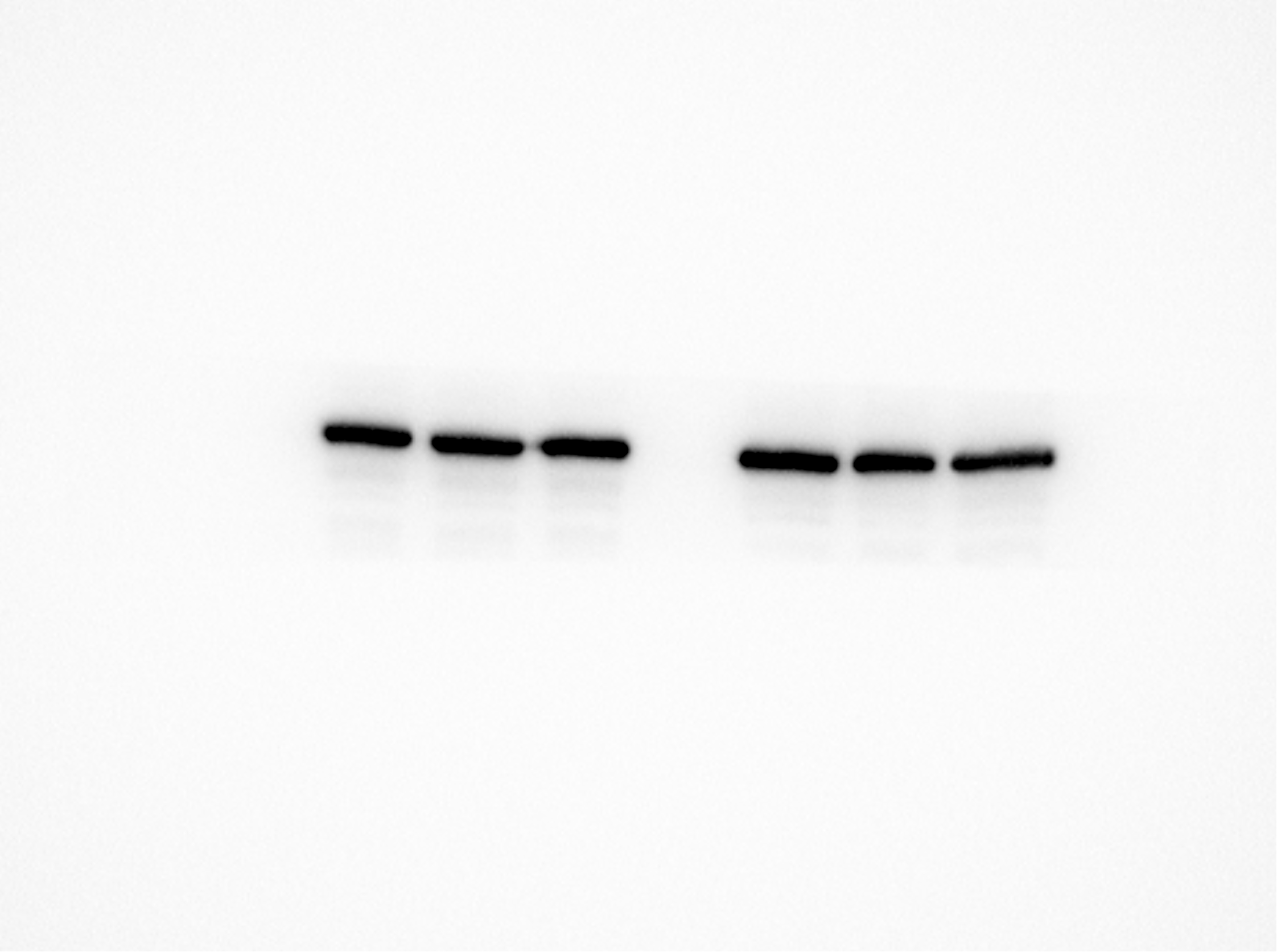

Supplement: Supplementary file 1 [file vetsci-12-00257-s001.zip › PABPC4 original blot images/Fig.4/C/IB/gapdh/s.tif]

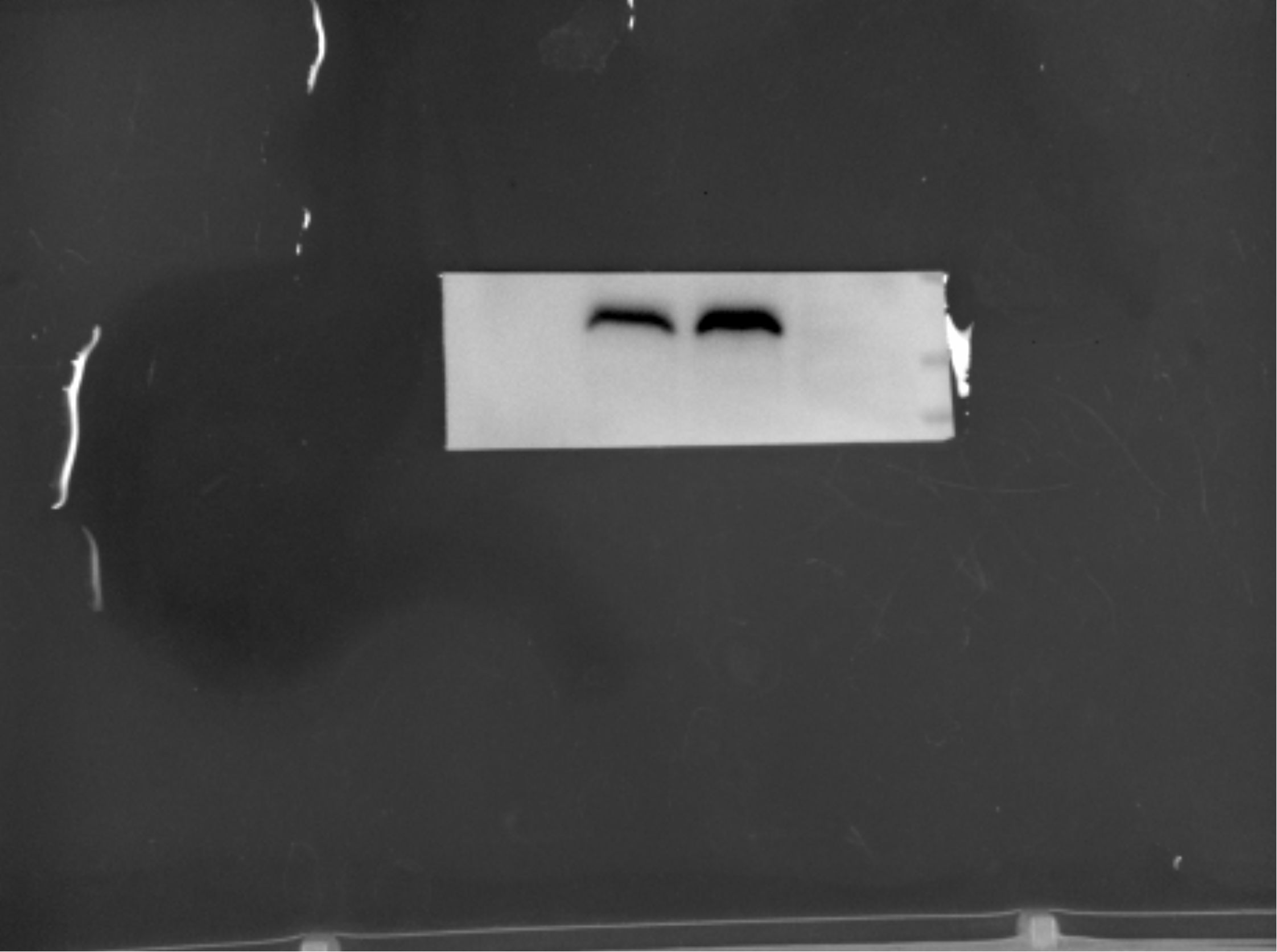

Supplement: Supplementary file 1 [file vetsci-12-00257-s001.zip › PABPC4 original blot images/Fig.4/C/IB/ha/1/merge.tif]

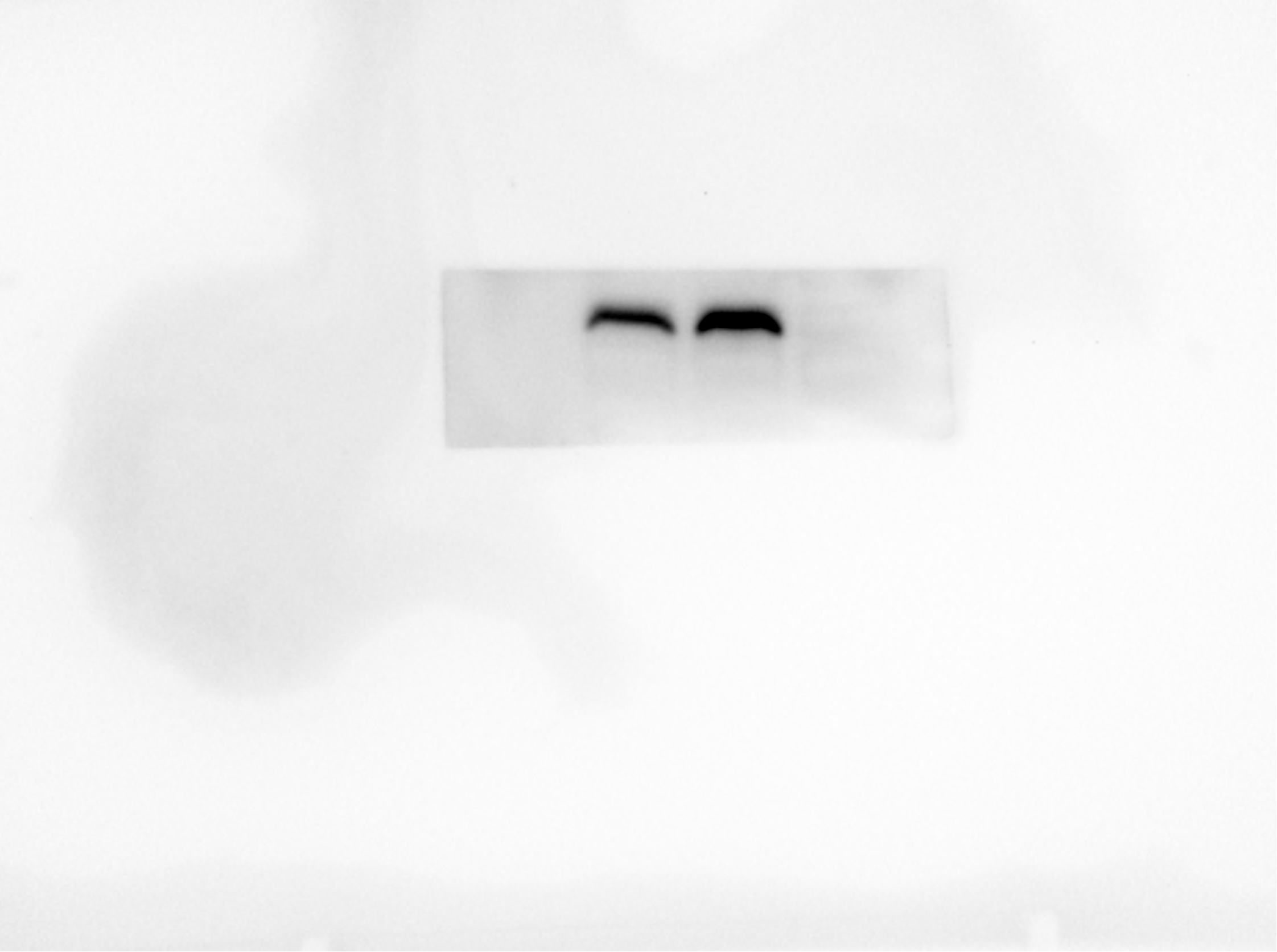

Supplement: Supplementary file 1 [file vetsci-12-00257-s001.zip › PABPC4 original blot images/Fig.4/C/IB/ha/1/s.tif]

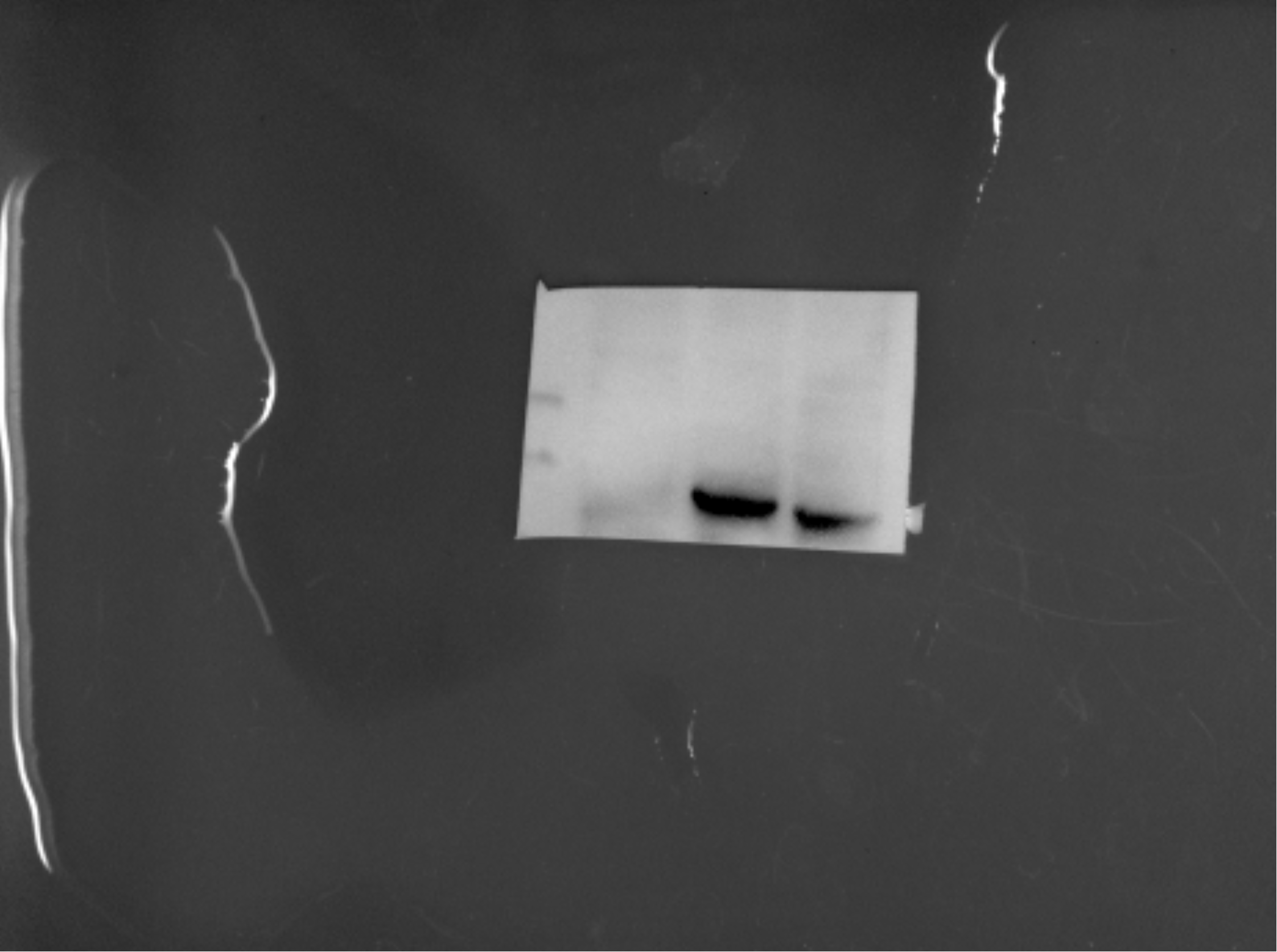

Supplement: Supplementary file 1 [file vetsci-12-00257-s001.zip › PABPC4 original blot images/Fig.4/C/IB/ha/2/merge.tif]

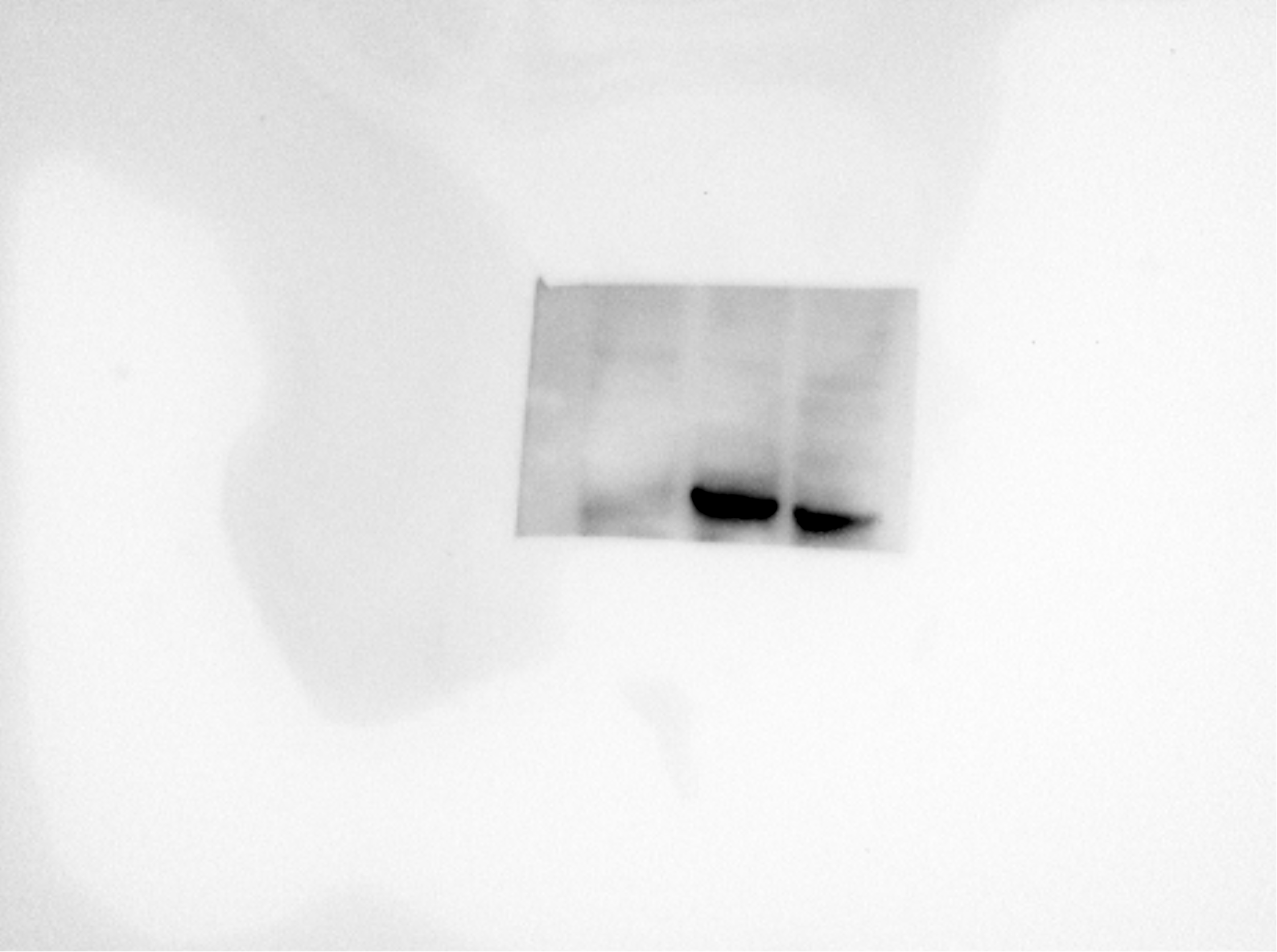

Supplement: Supplementary file 1 [file vetsci-12-00257-s001.zip › PABPC4 original blot images/Fig.4/C/IB/ha/2/s.tif]

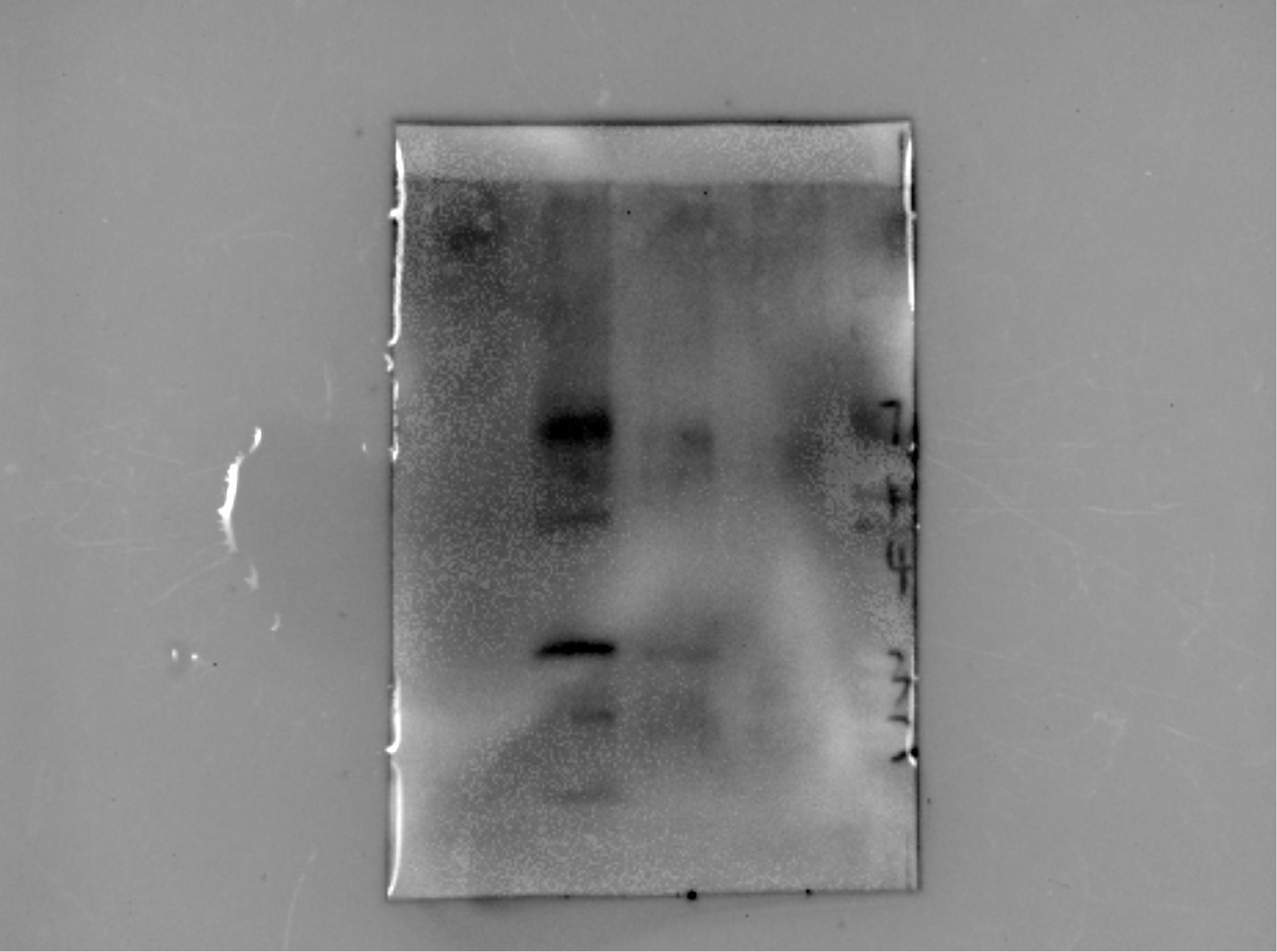

Supplement: Supplementary file 1 [file vetsci-12-00257-s001.zip › PABPC4 original blot images/Fig.4/C/IP/flag/HA/MERGE.tif]

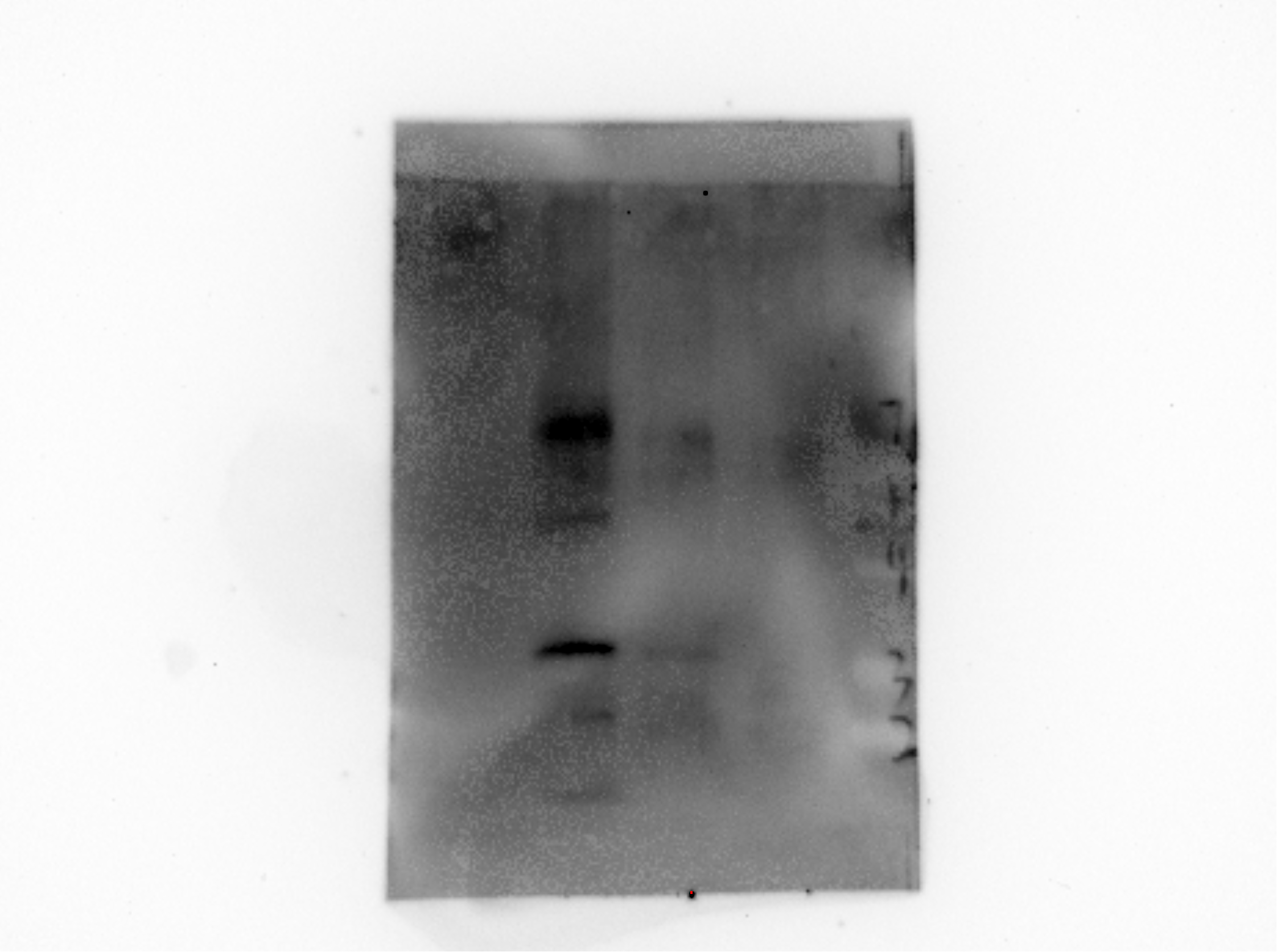

Supplement: Supplementary file 1 [file vetsci-12-00257-s001.zip › PABPC4 original blot images/Fig.4/C/IP/flag/HA/S.tif]

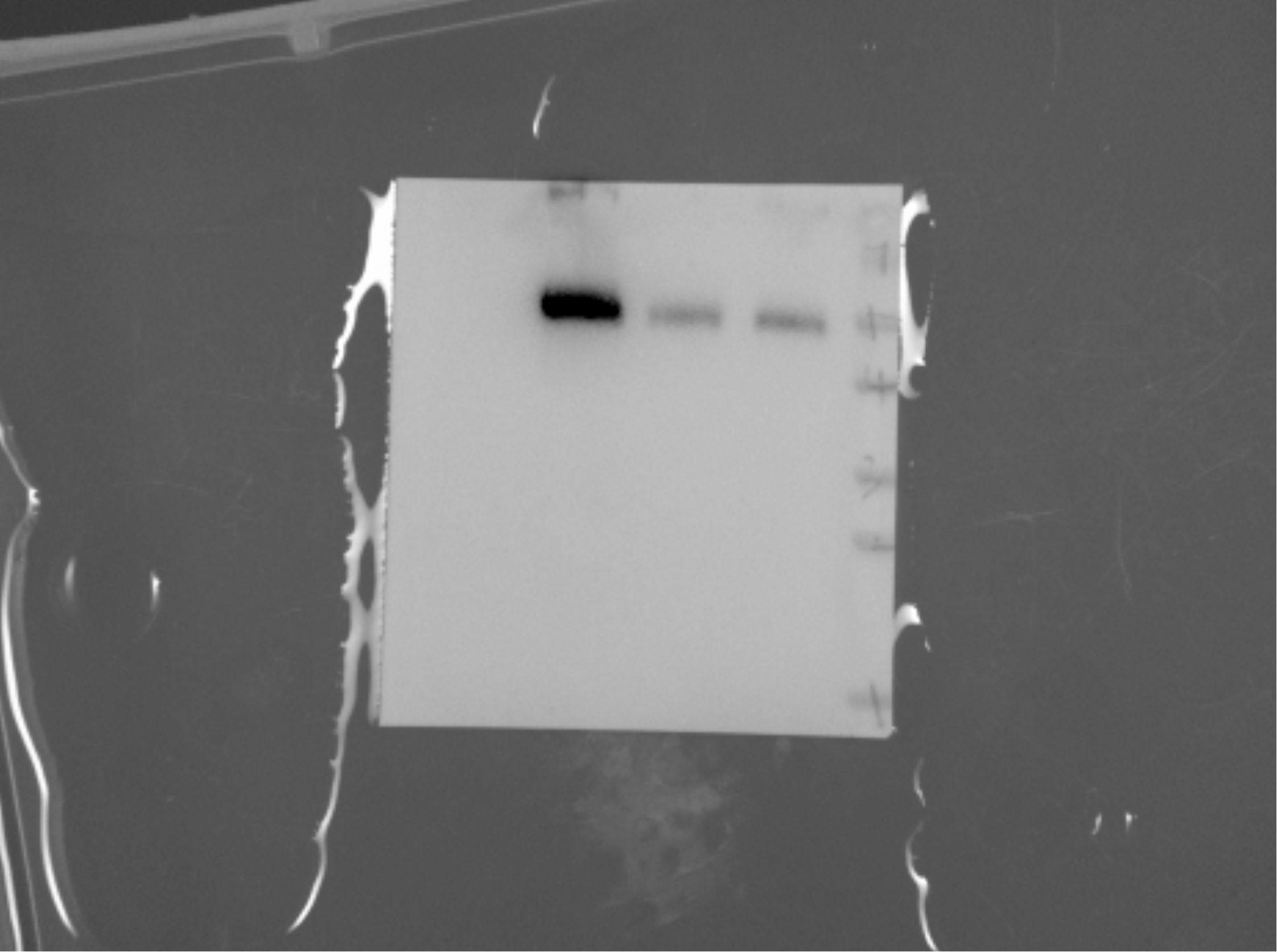

Supplement: Supplementary file 1 [file vetsci-12-00257-s001.zip › PABPC4 original blot images/Fig.4/C/IP/HA/flag/h.tif]

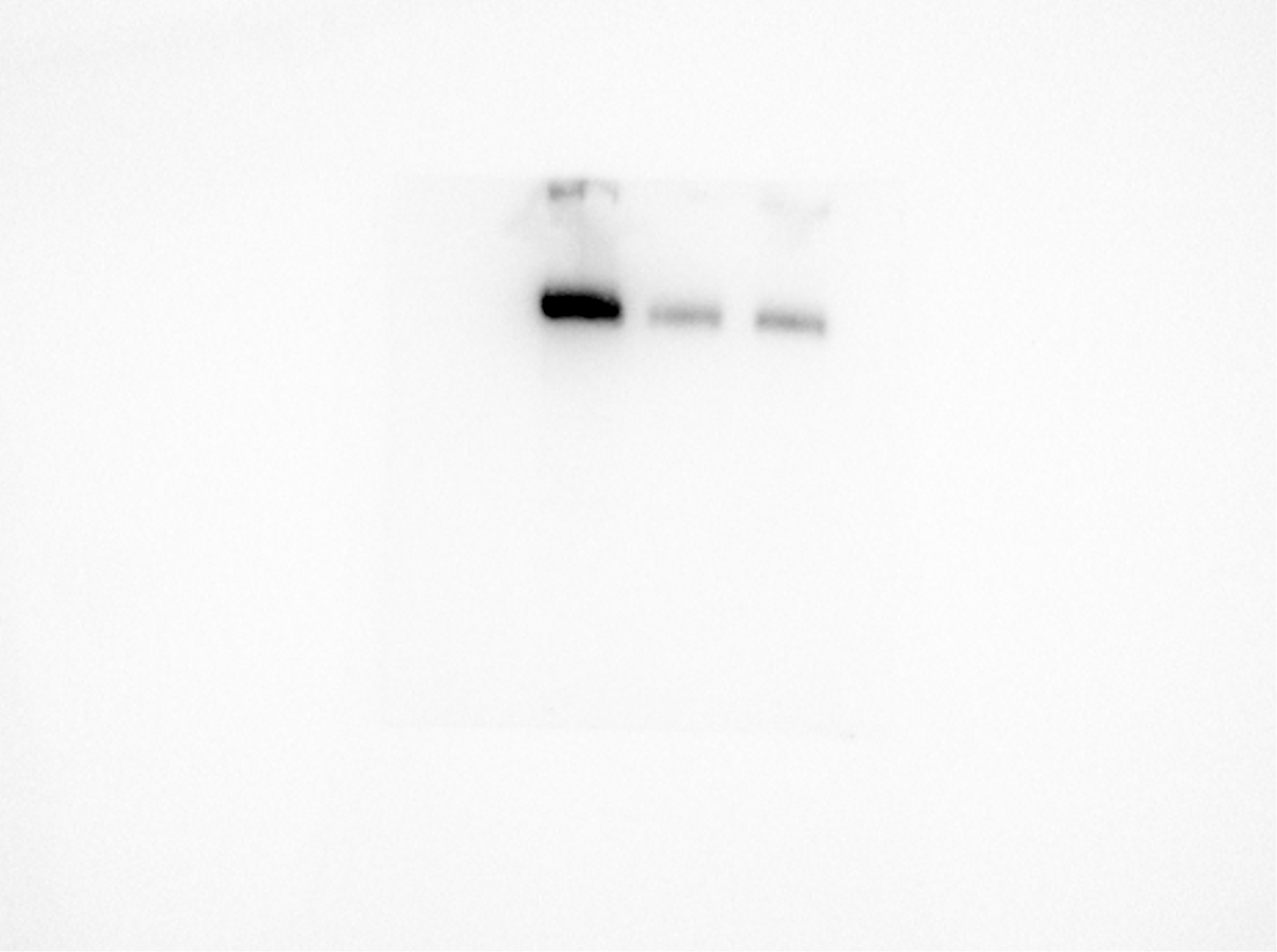

Supplement: Supplementary file 1 [file vetsci-12-00257-s001.zip › PABPC4 original blot images/Fig.4/C/IP/HA/flag/s.tif]

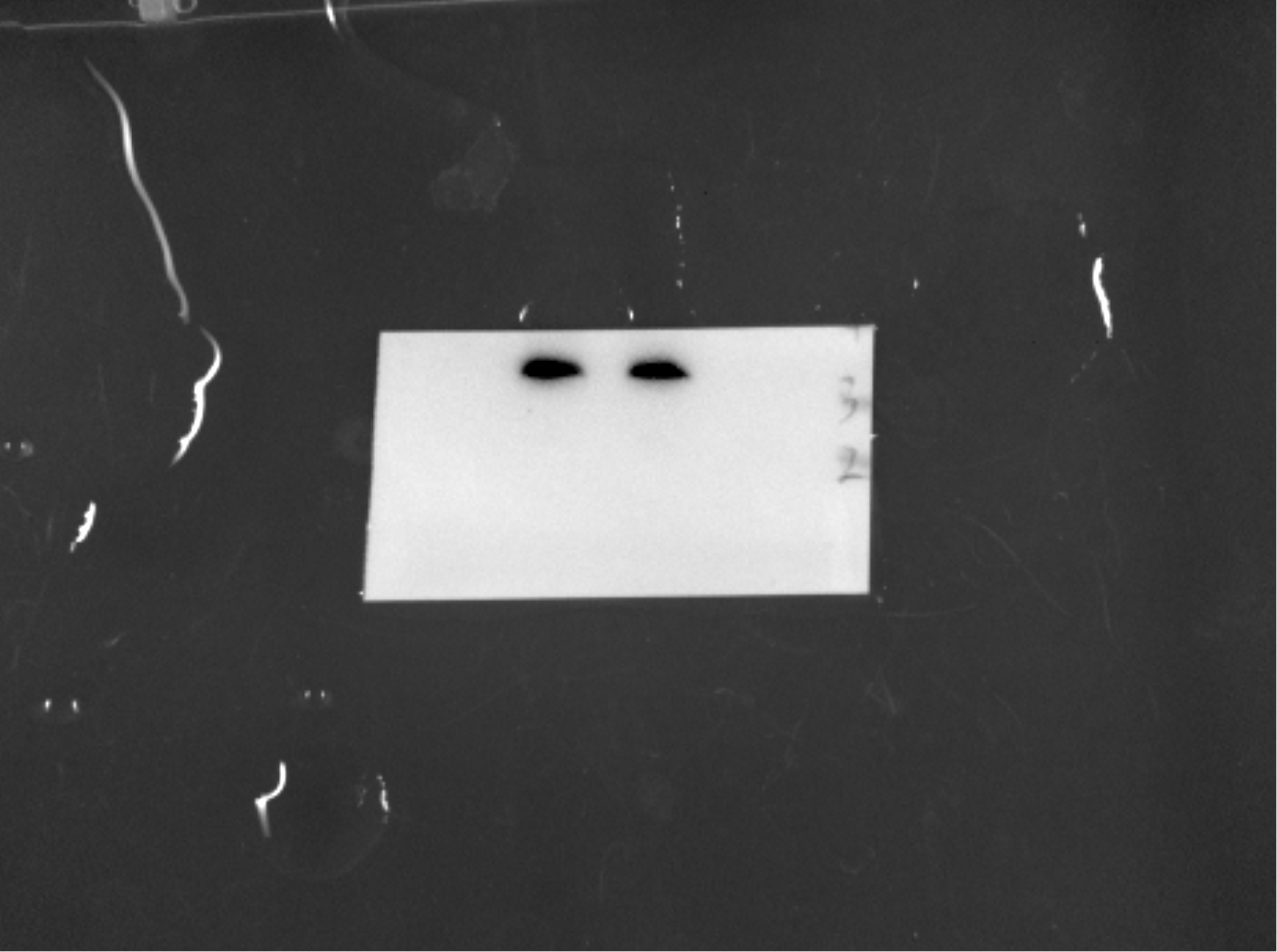

Supplement: Supplementary file 1 [file vetsci-12-00257-s001.zip › PABPC4 original blot images/Fig.4/C/IP/HA/ha-ha/h.tif]

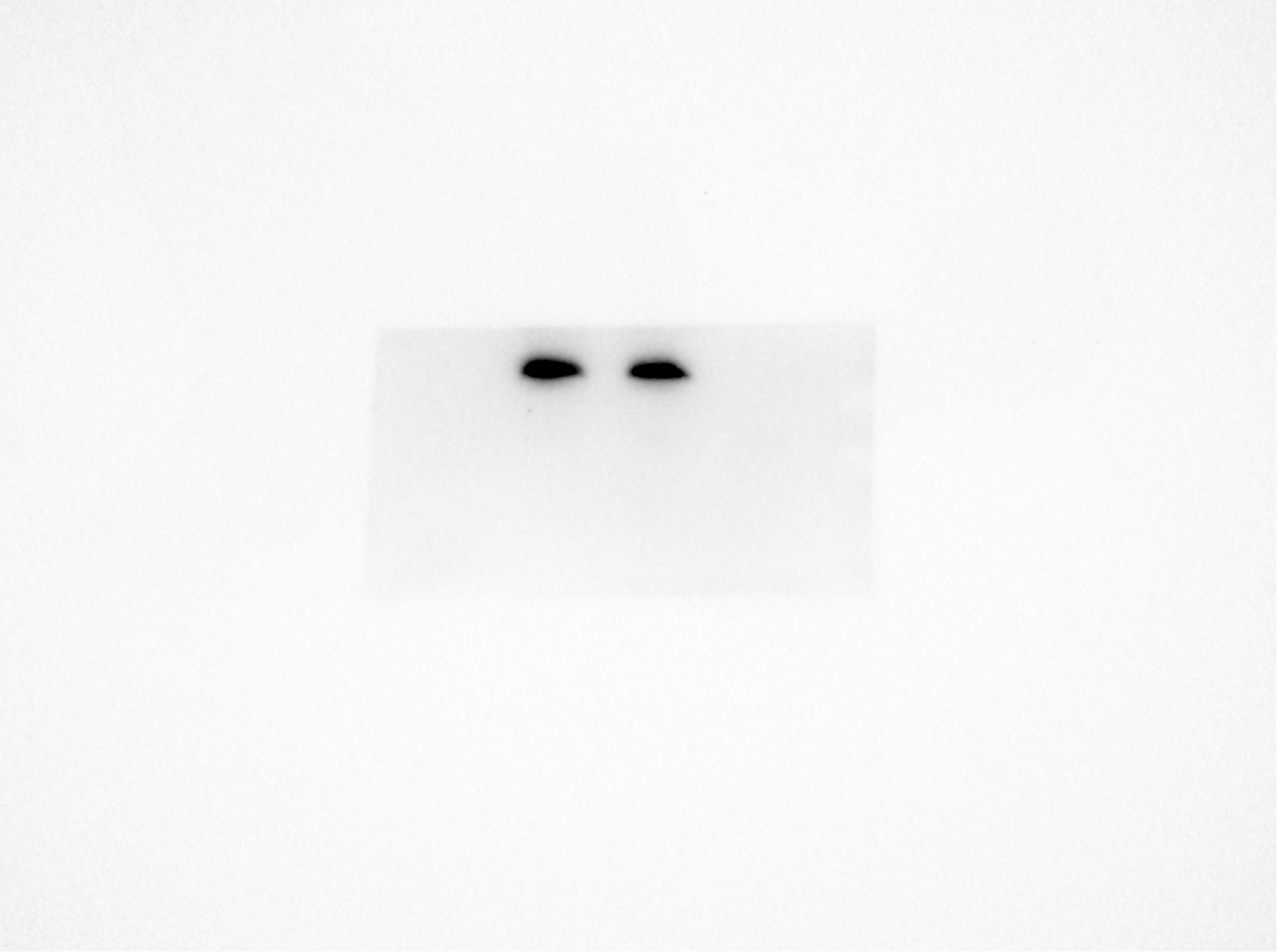

Supplement: Supplementary file 1 [file vetsci-12-00257-s001.zip › PABPC4 original blot images/Fig.4/C/IP/HA/ha-ha/s.tif]

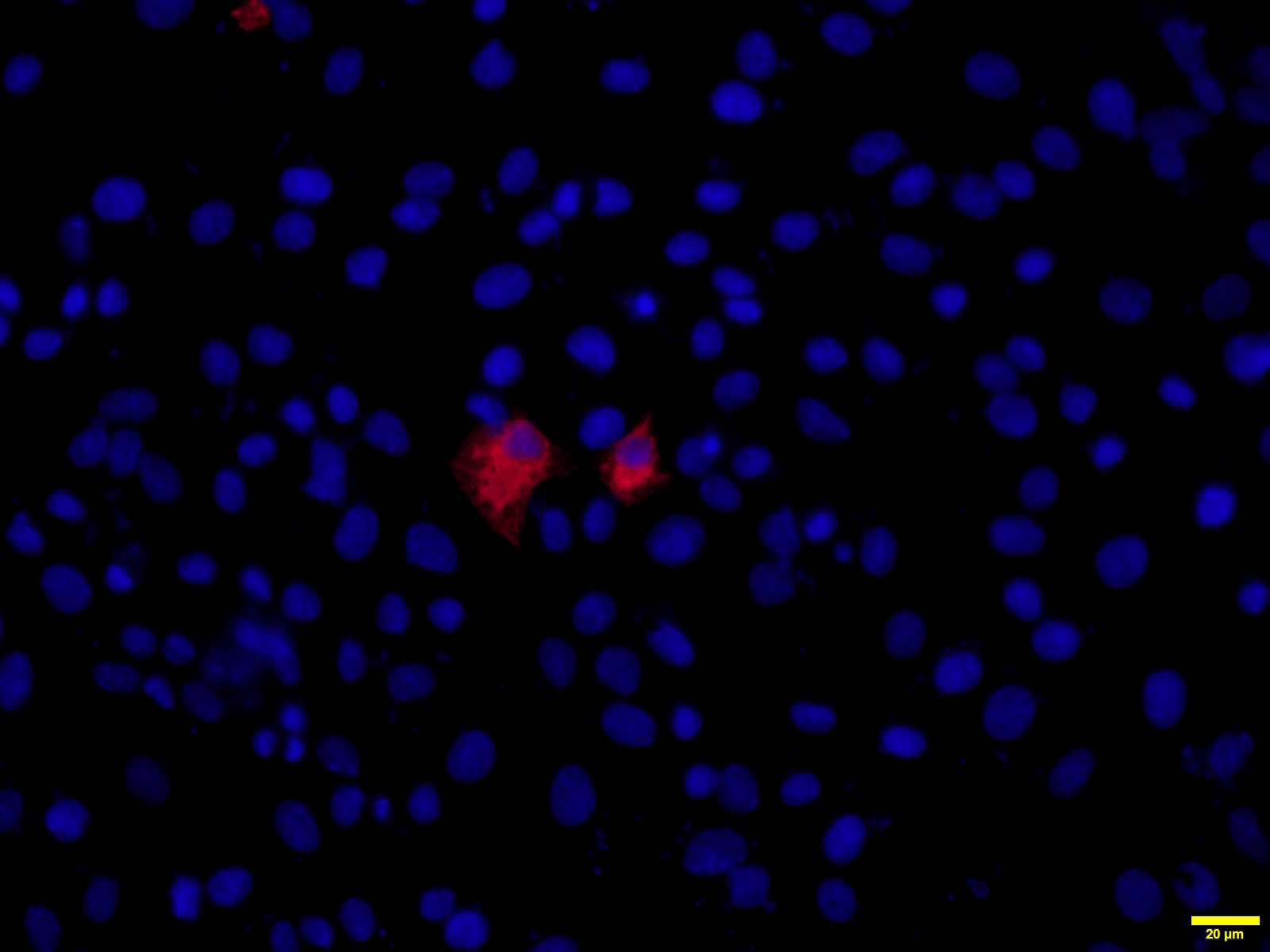

Supplement: Supplementary file 1 [file vetsci-12-00257-s001.zip › PABPC4 original blot images/Fig.4/D/MARCHF8/图像_02.jpg]

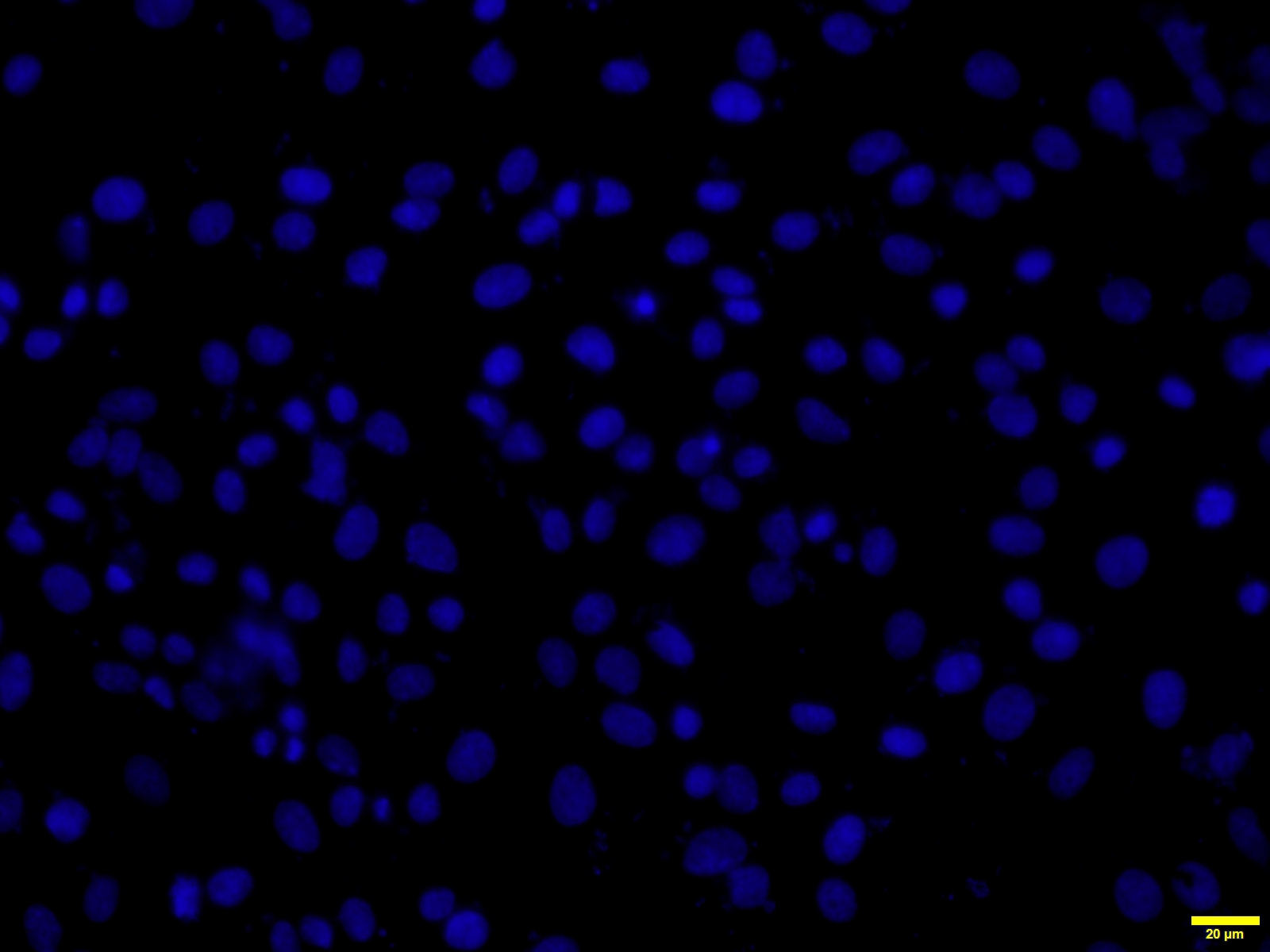

Supplement: Supplementary file 1 [file vetsci-12-00257-s001.zip › PABPC4 original blot images/Fig.4/D/MARCHF8/图像_12748.jpg]

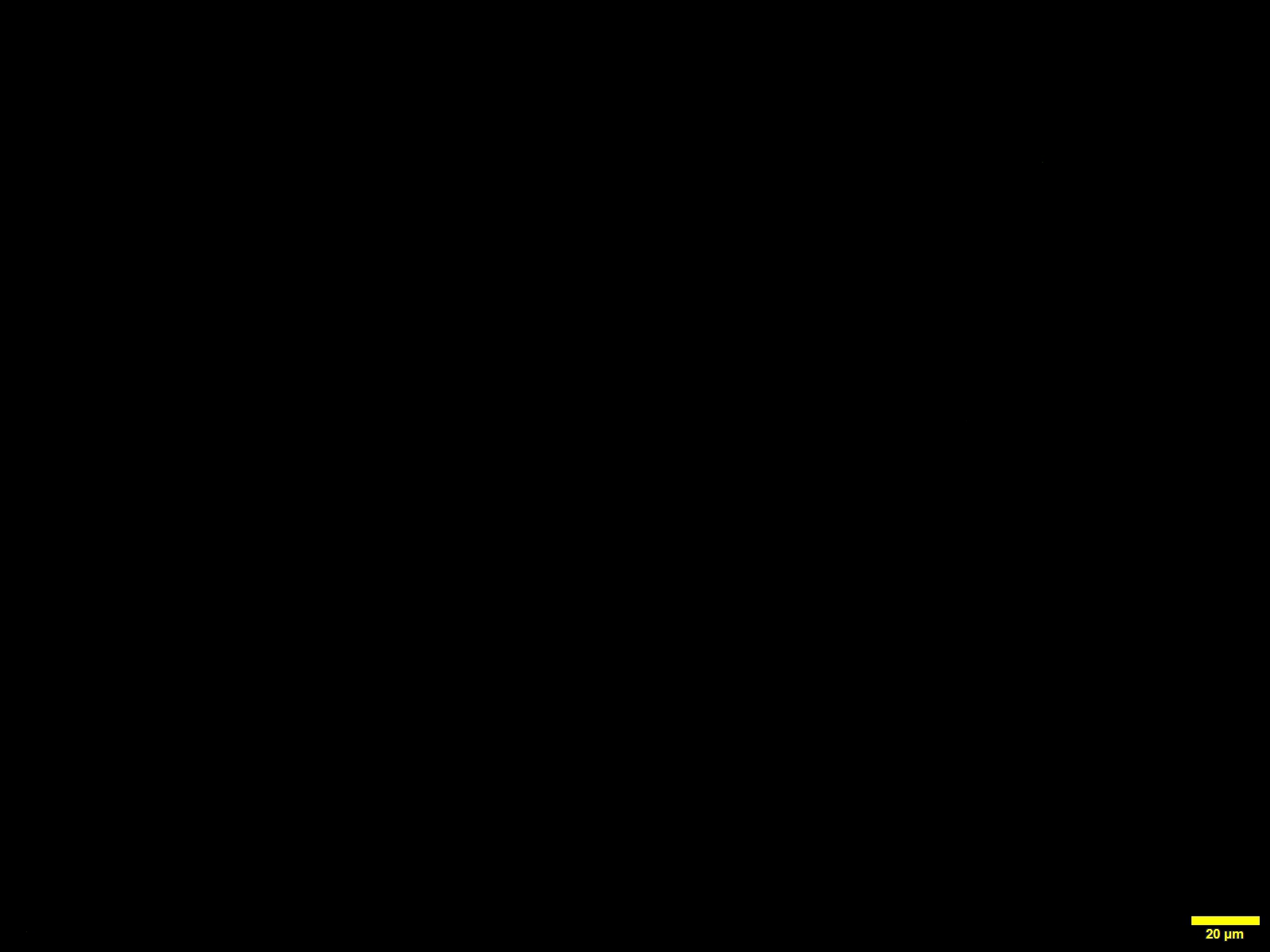

Supplement: Supplementary file 1 [file vetsci-12-00257-s001.zip › PABPC4 original blot images/Fig.4/D/MARCHF8/图像_12749.jpg]

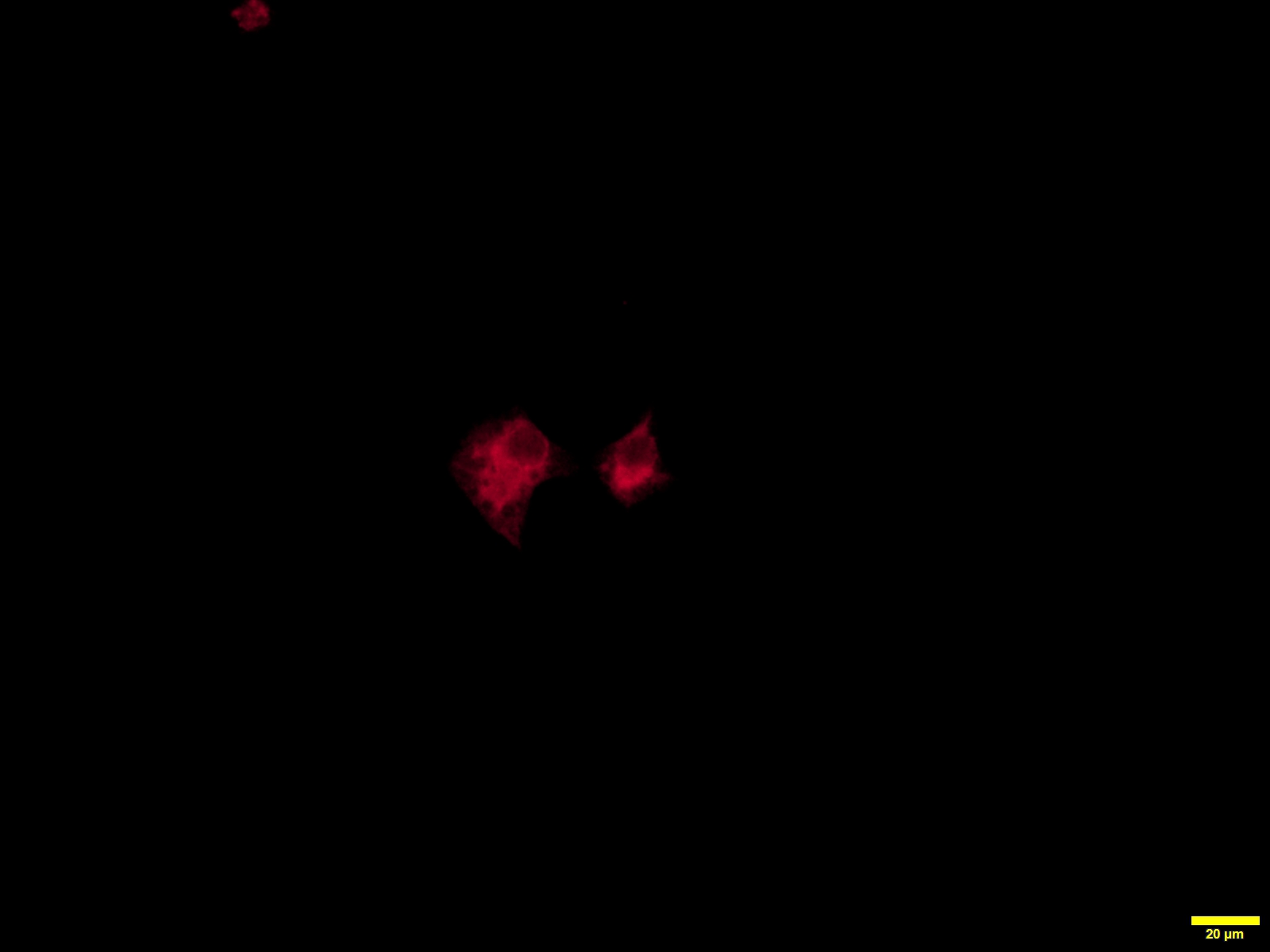

Supplement: Supplementary file 1 [file vetsci-12-00257-s001.zip › PABPC4 original blot images/Fig.4/D/MARCHF8/图像_12750.jpg]

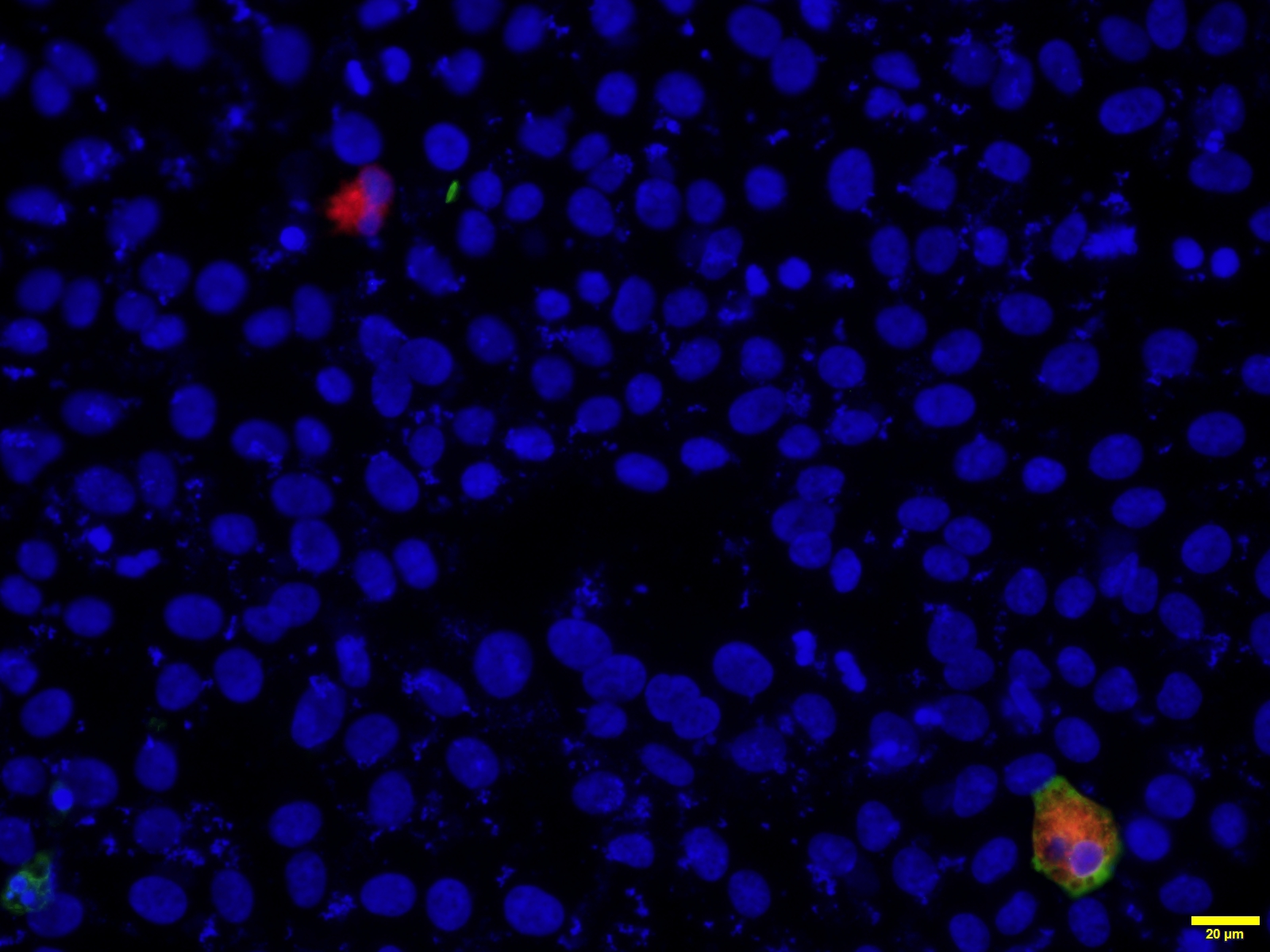

Supplement: Supplementary file 1 [file vetsci-12-00257-s001.zip › PABPC4 original blot images/Fig.4/D/MARCHF8+N/图像_04.jpg]

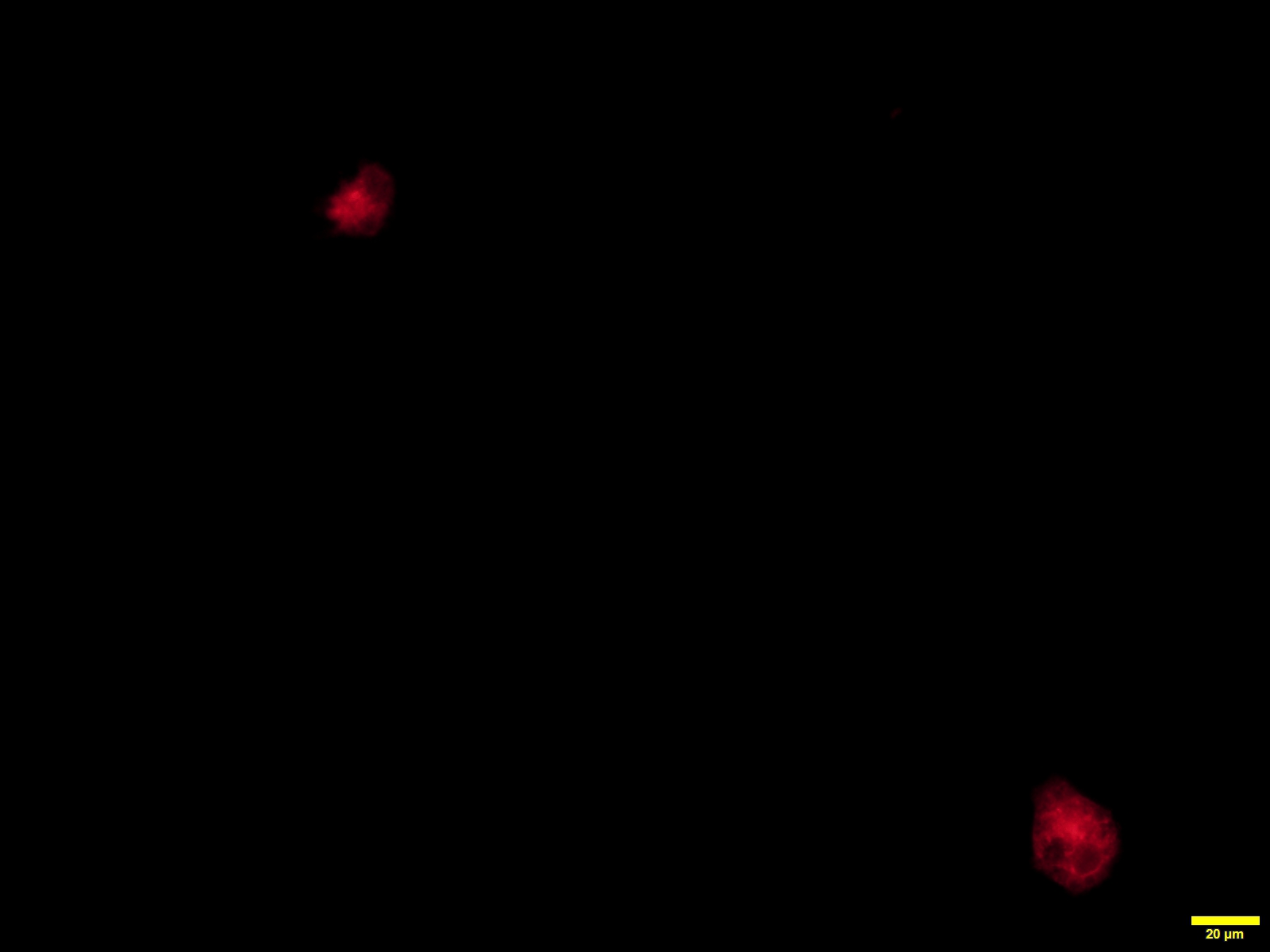

Supplement: Supplementary file 1 [file vetsci-12-00257-s001.zip › PABPC4 original blot images/Fig.4/D/MARCHF8+N/图像_12660.jpg]

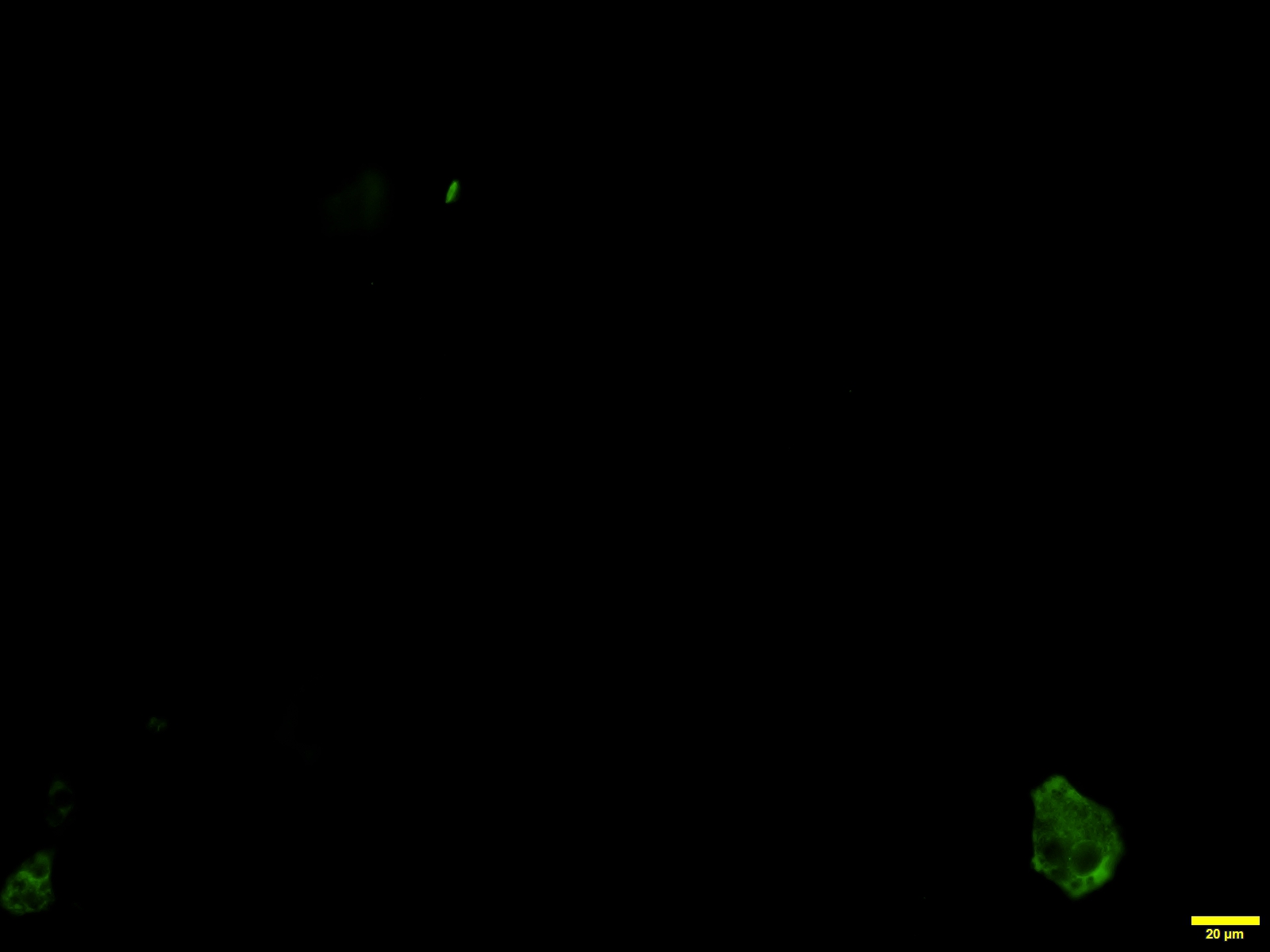

Supplement: Supplementary file 1 [file vetsci-12-00257-s001.zip › PABPC4 original blot images/Fig.4/D/MARCHF8+N/图像_12661.jpg]

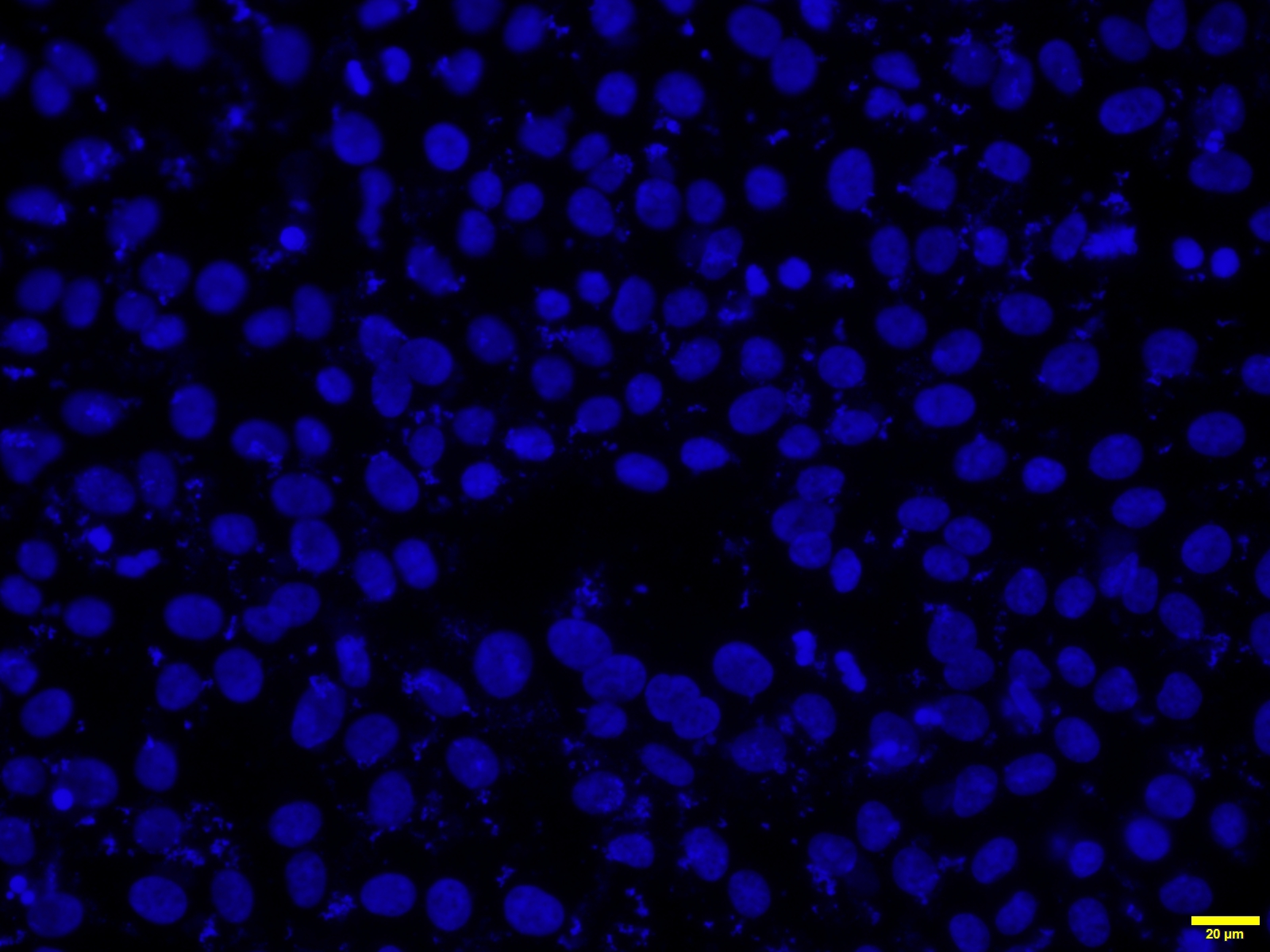

Supplement: Supplementary file 1 [file vetsci-12-00257-s001.zip › PABPC4 original blot images/Fig.4/D/MARCHF8+N/图像_12662.jpg]

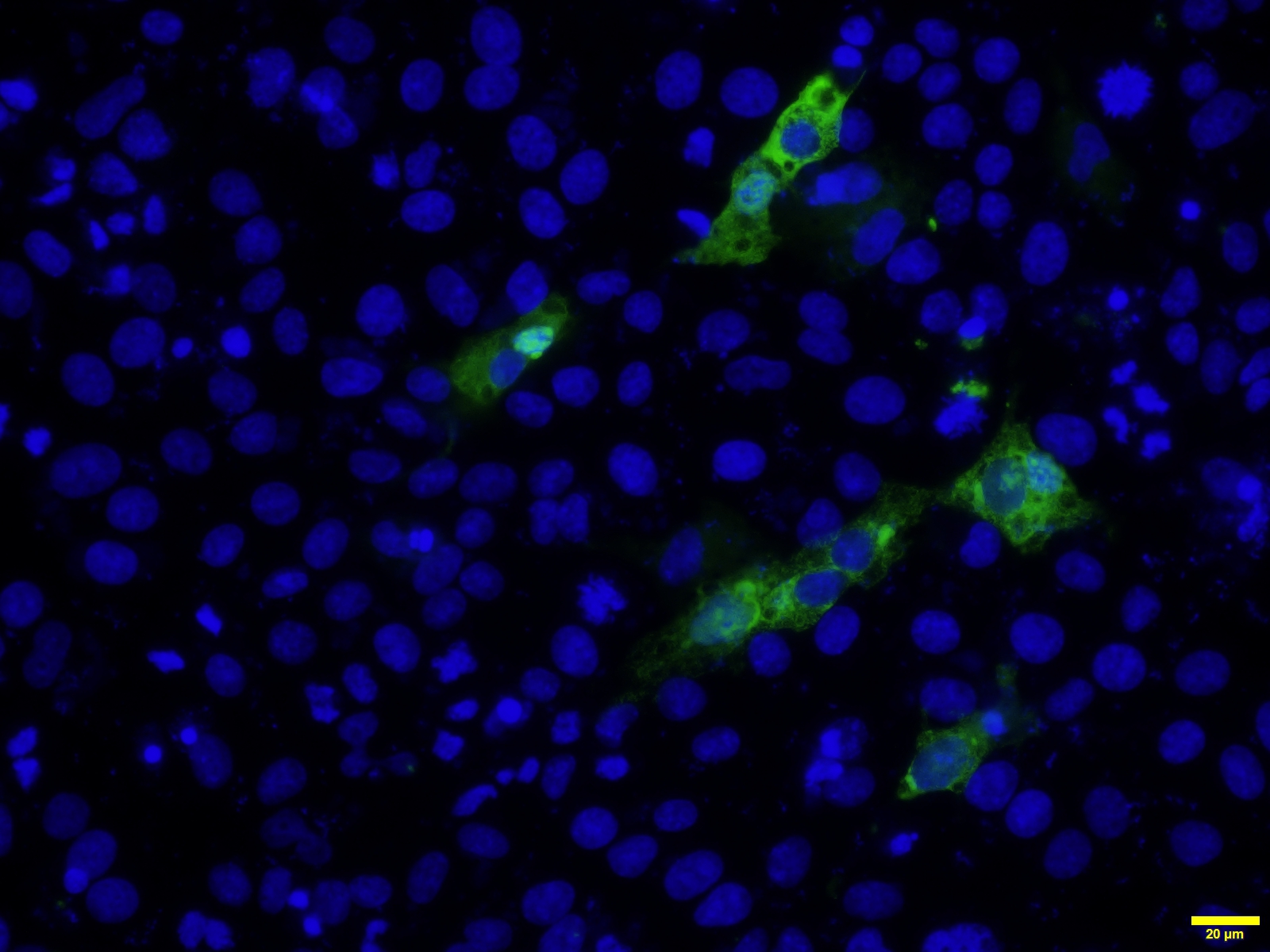

Supplement: Supplementary file 1 [file vetsci-12-00257-s001.zip › PABPC4 original blot images/Fig.4/D/N/图像_02.jpg]

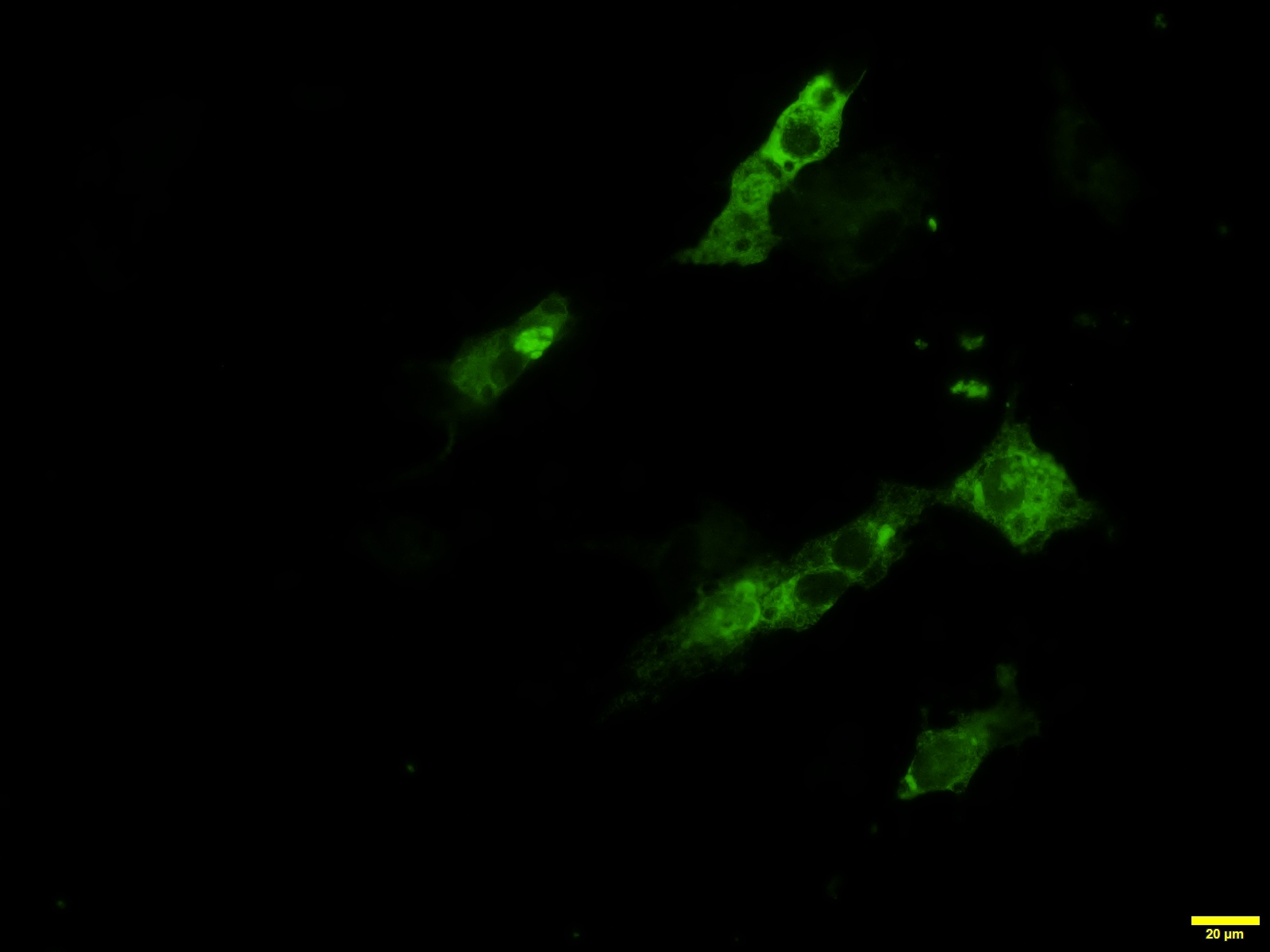

Supplement: Supplementary file 1 [file vetsci-12-00257-s001.zip › PABPC4 original blot images/Fig.4/D/N/图像_12604.jpg]

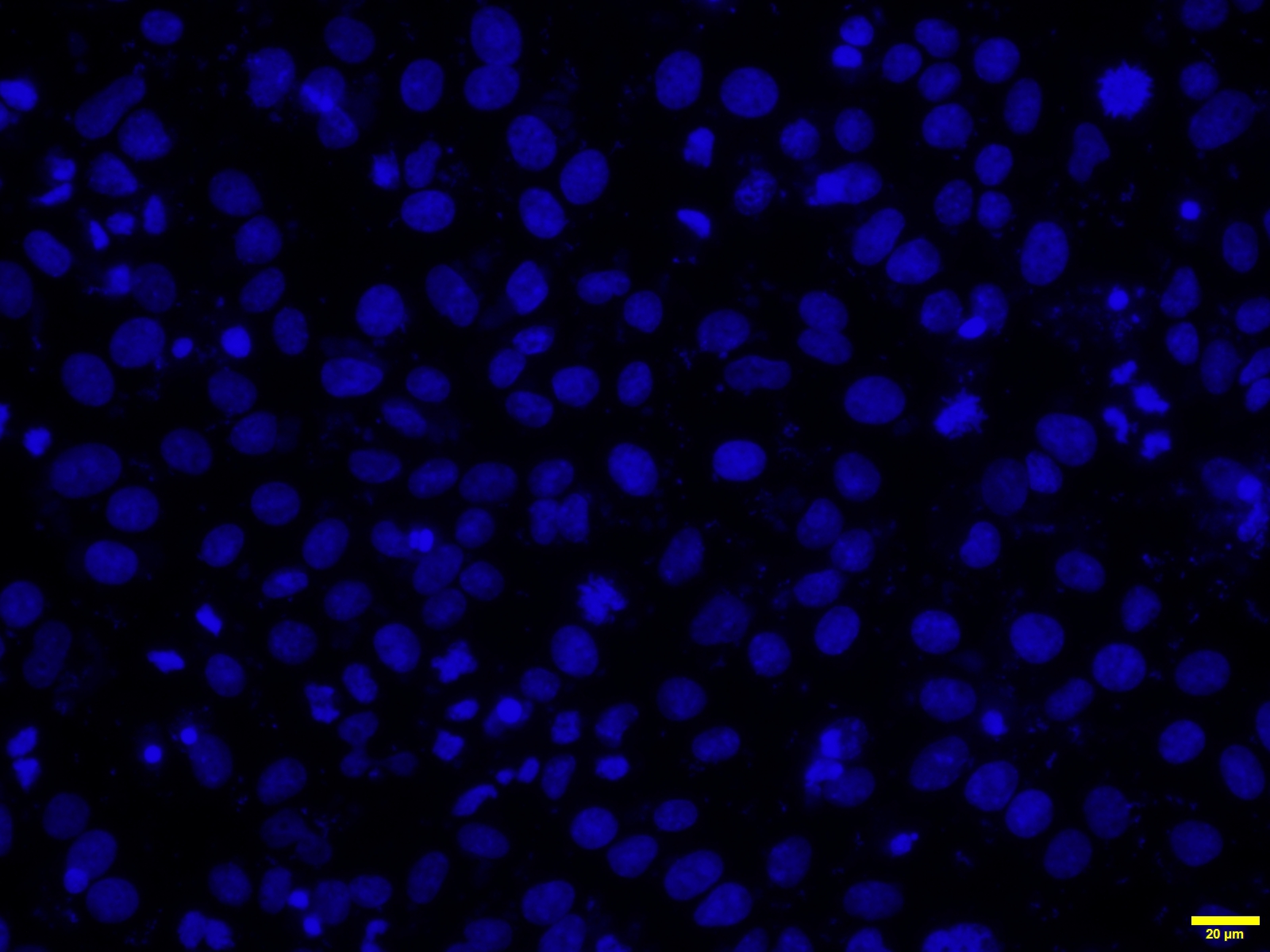

Supplement: Supplementary file 1 [file vetsci-12-00257-s001.zip › PABPC4 original blot images/Fig.4/D/N/图像_12605.jpg]

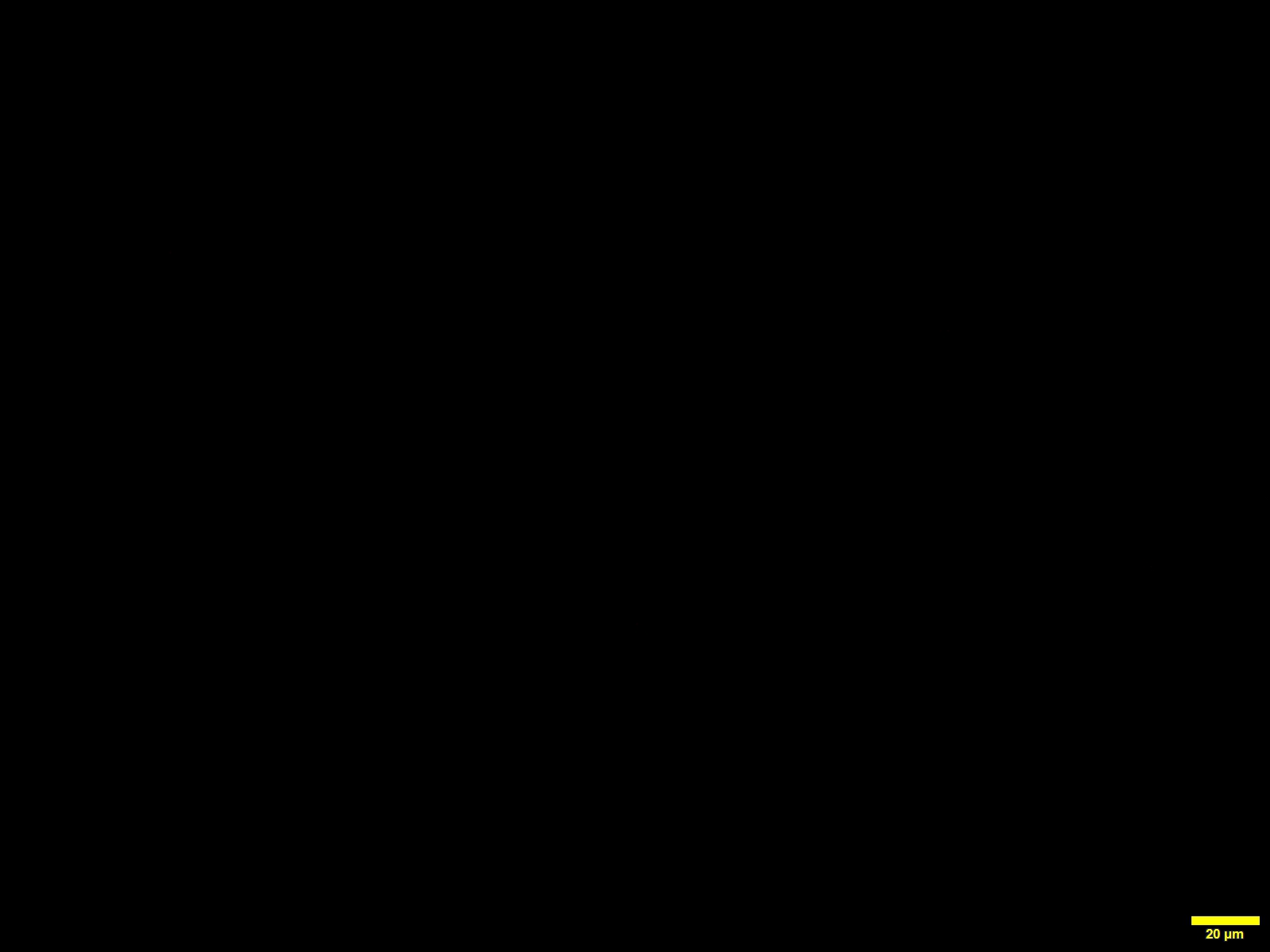

Supplement: Supplementary file 1 [file vetsci-12-00257-s001.zip › PABPC4 original blot images/Fig.4/D/N/图像_12606.jpg]

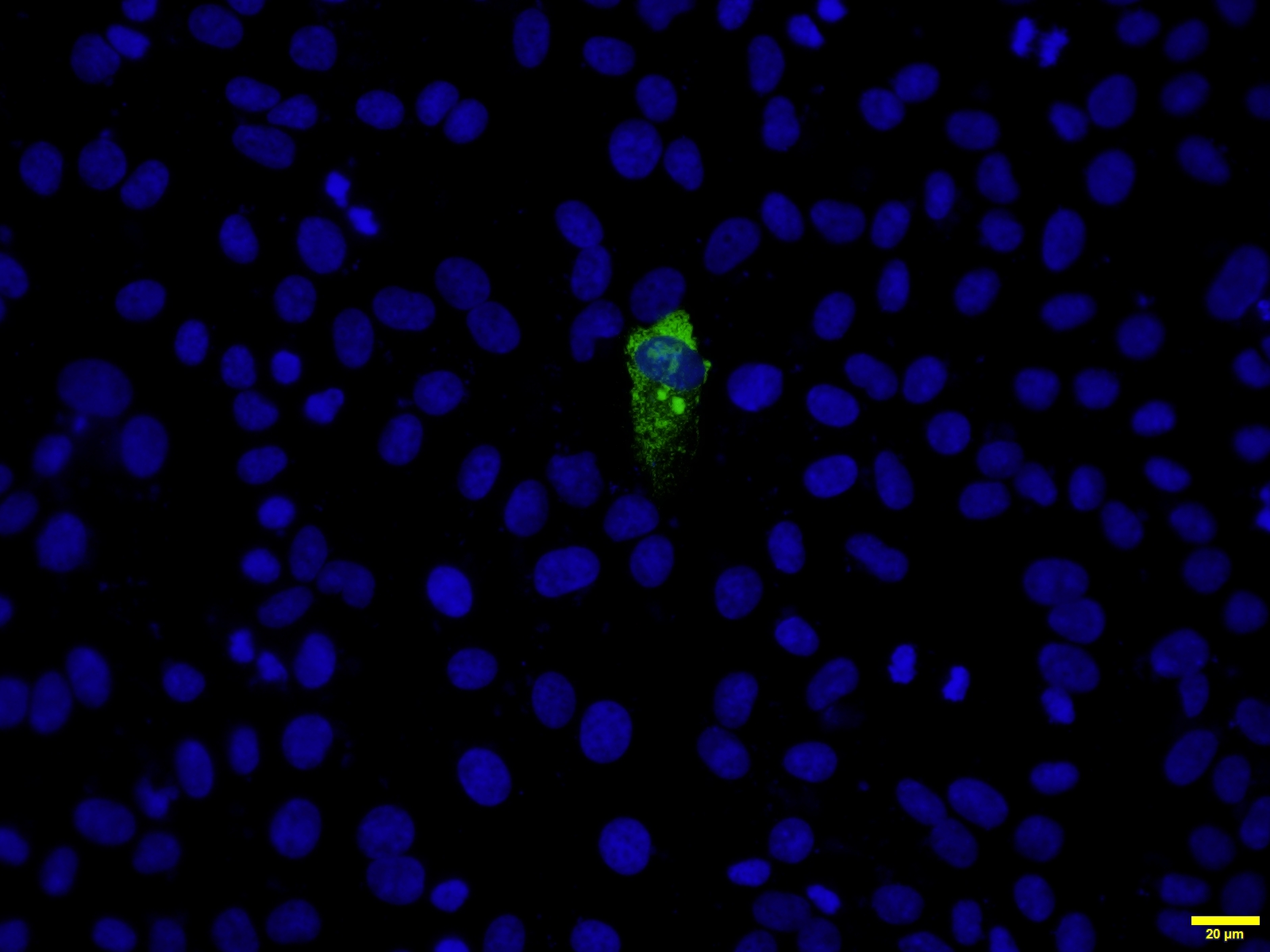

Supplement: Supplementary file 1 [file vetsci-12-00257-s001.zip › PABPC4 original blot images/Fig.4/E/N/图像_01.jpg]

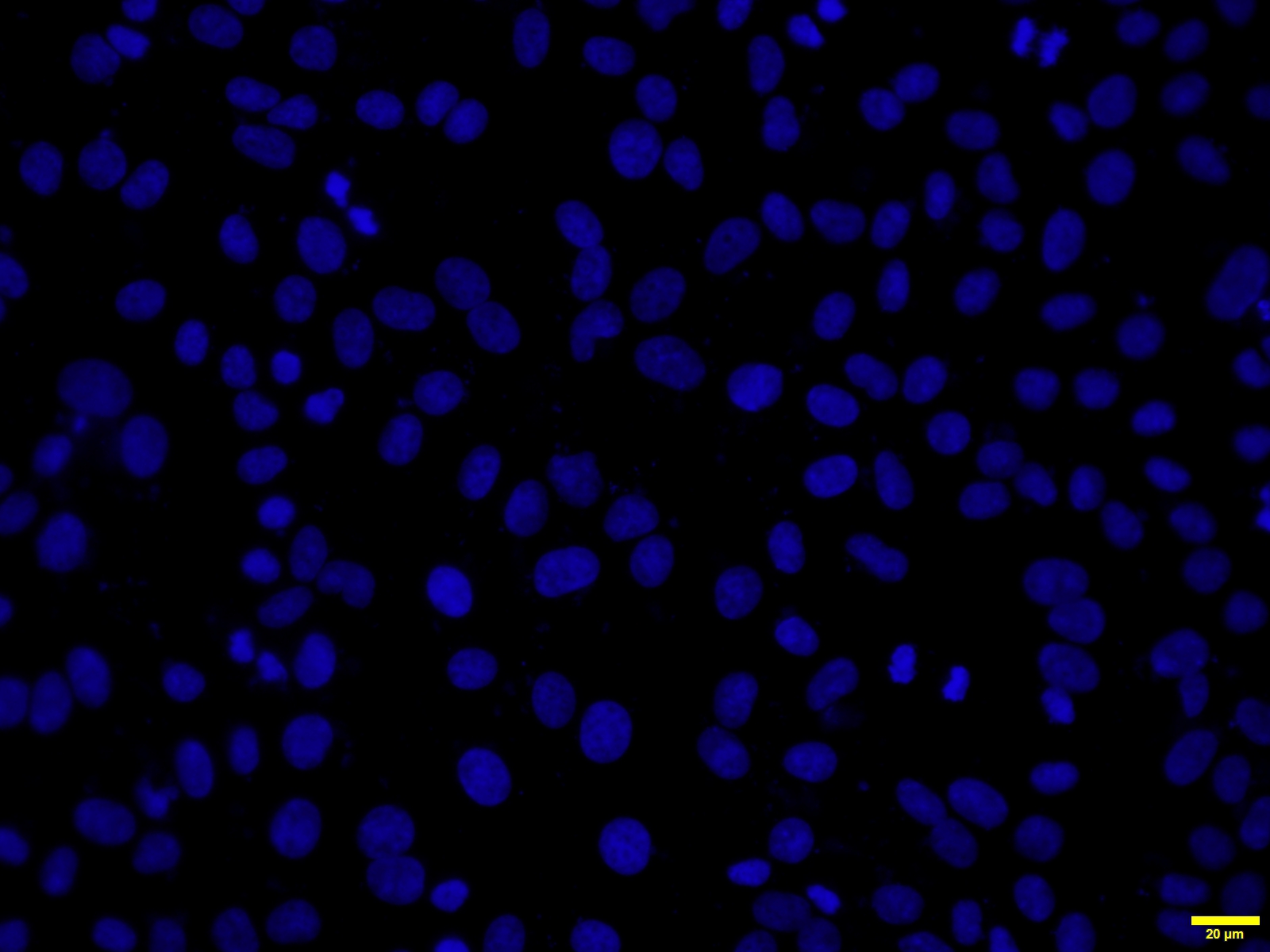

Supplement: Supplementary file 1 [file vetsci-12-00257-s001.zip › PABPC4 original blot images/Fig.4/E/N/图像_12861.jpg]

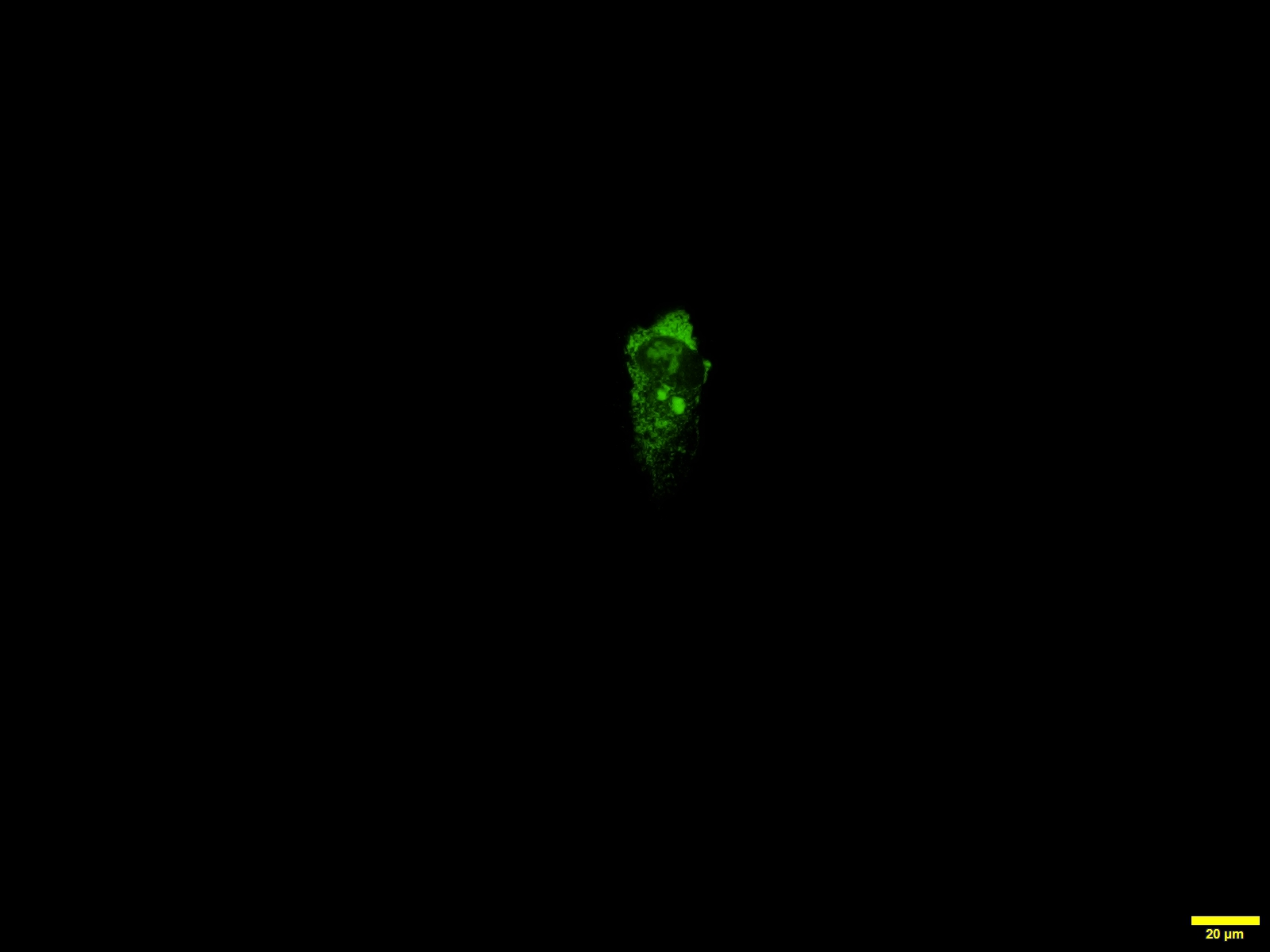

Supplement: Supplementary file 1 [file vetsci-12-00257-s001.zip › PABPC4 original blot images/Fig.4/E/N/图像_12862.jpg]

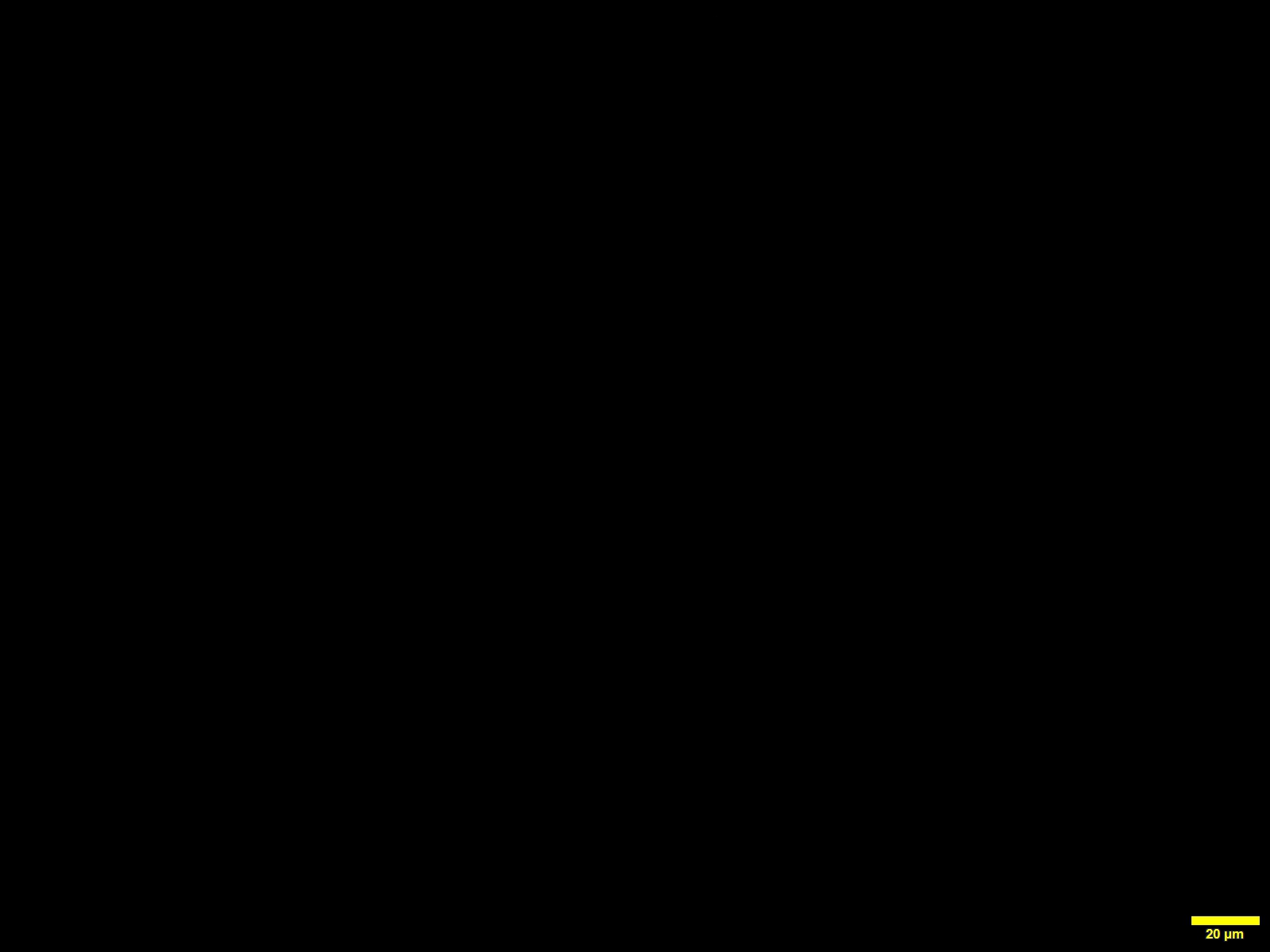

Supplement: Supplementary file 1 [file vetsci-12-00257-s001.zip › PABPC4 original blot images/Fig.4/E/N/图像_12863.jpg]

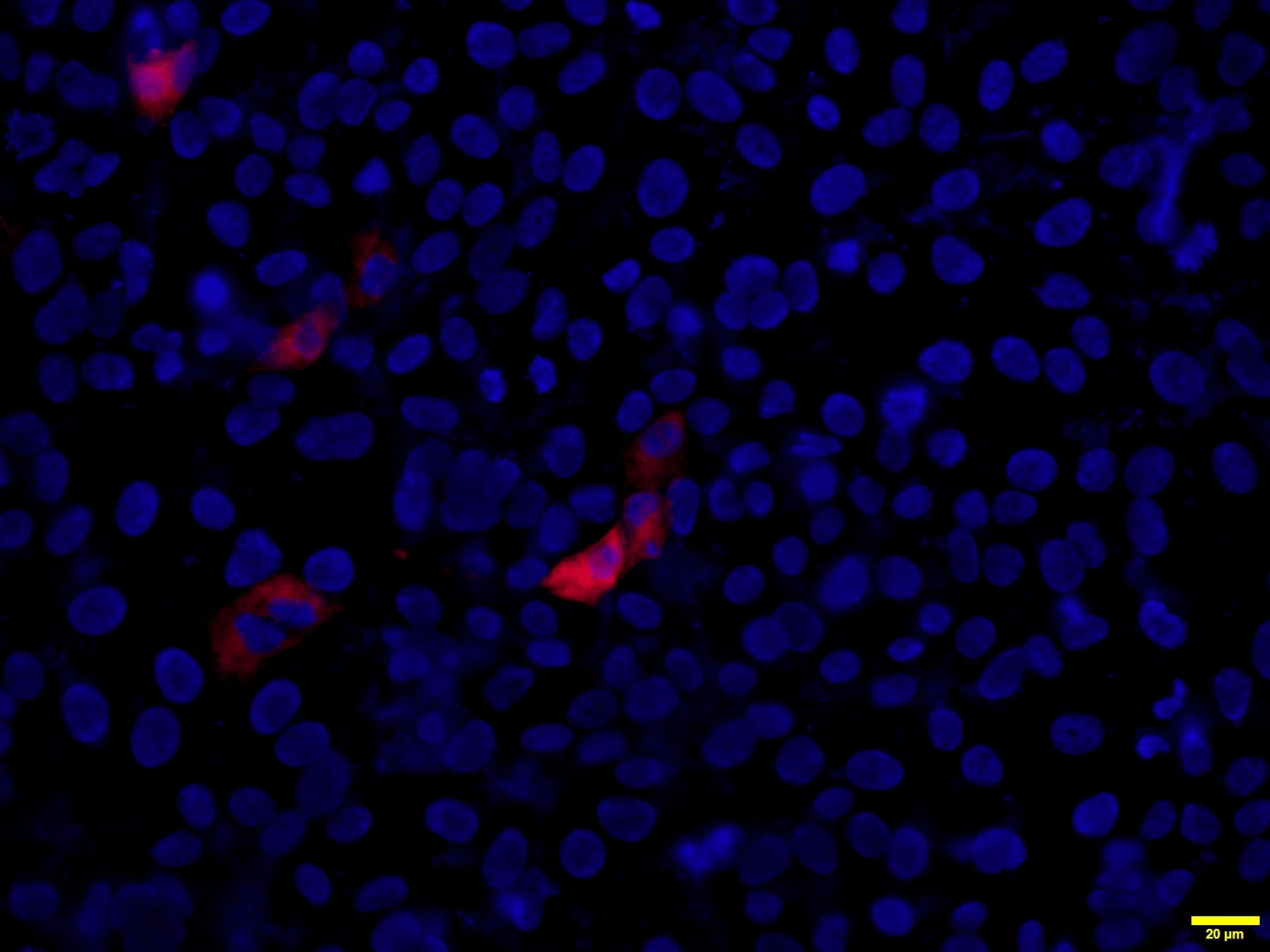

Supplement: Supplementary file 1 [file vetsci-12-00257-s001.zip › PABPC4 original blot images/Fig.4/E/NDP52/图像_03.jpg]

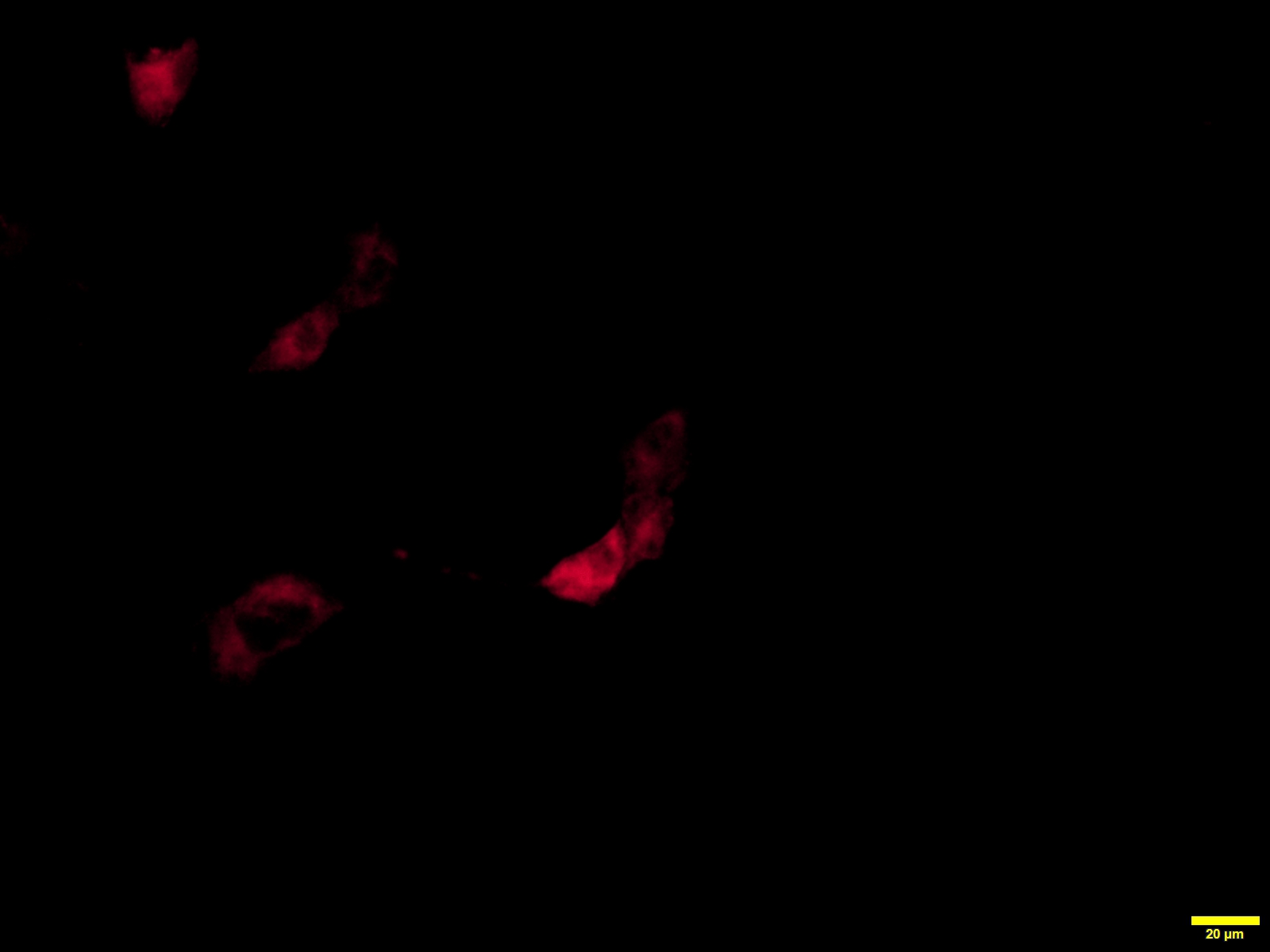

Supplement: Supplementary file 1 [file vetsci-12-00257-s001.zip › PABPC4 original blot images/Fig.4/E/NDP52/图像_12854.jpg]

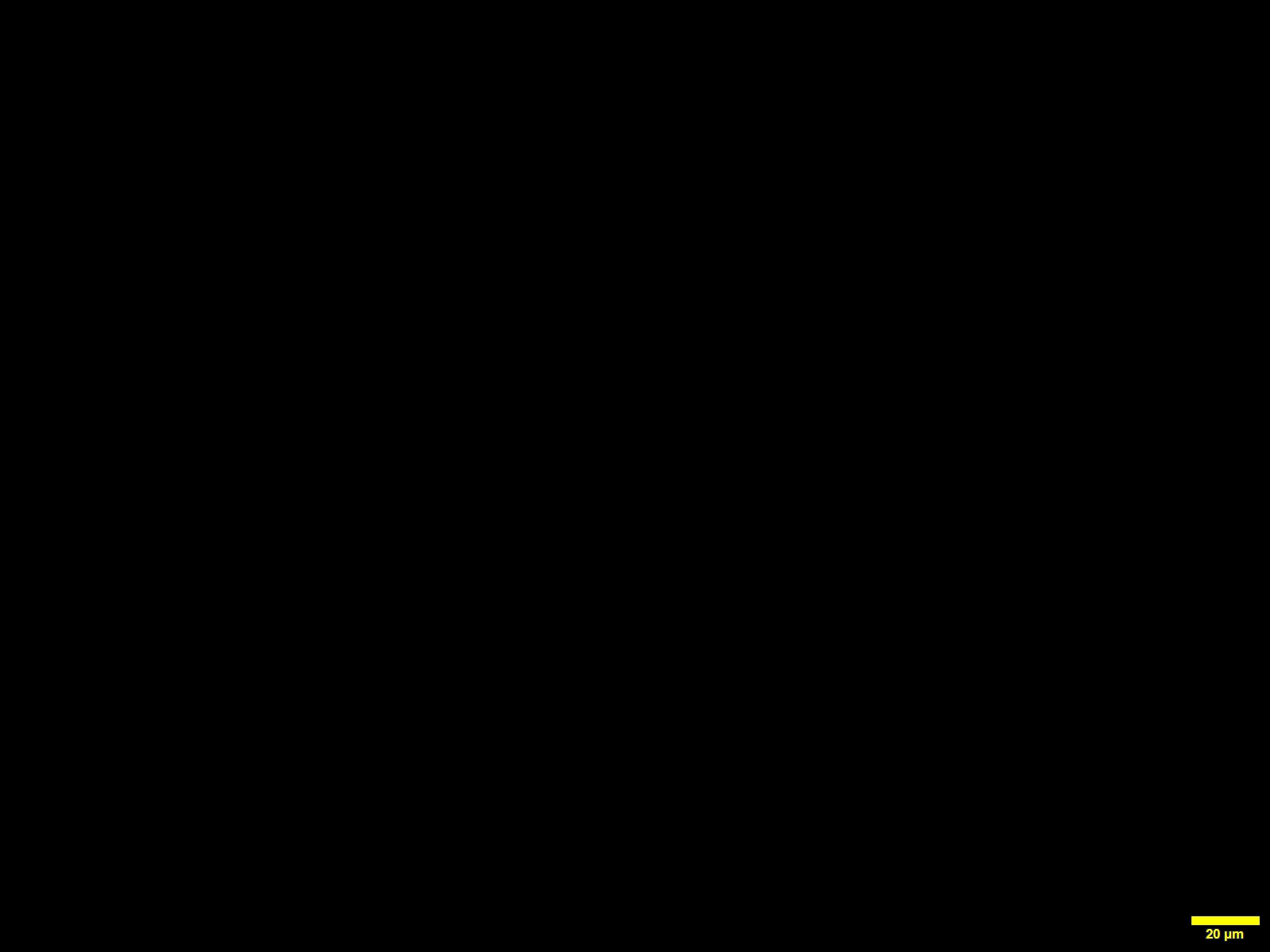

Supplement: Supplementary file 1 [file vetsci-12-00257-s001.zip › PABPC4 original blot images/Fig.4/E/NDP52/图像_12855.jpg]

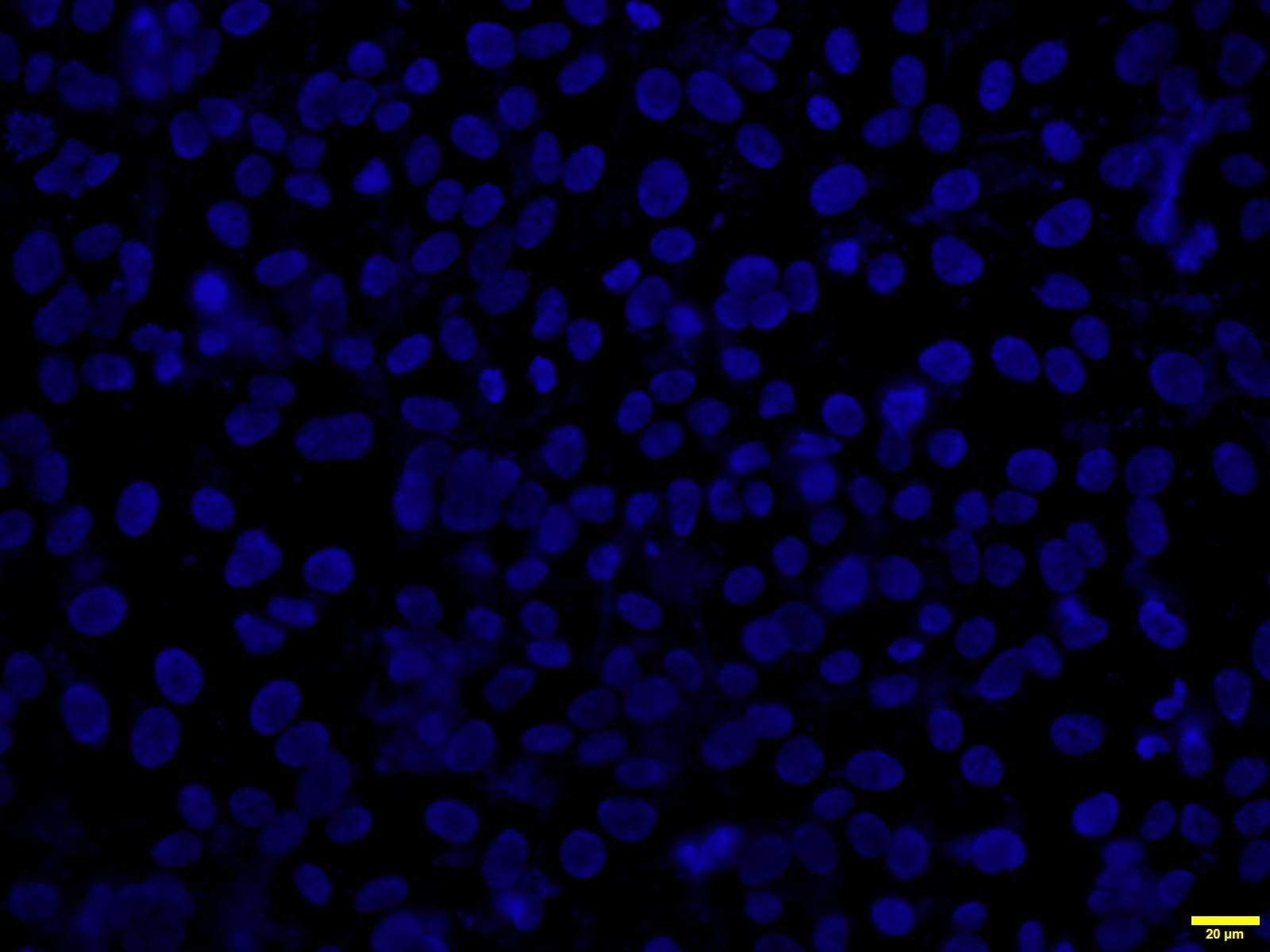

Supplement: Supplementary file 1 [file vetsci-12-00257-s001.zip › PABPC4 original blot images/Fig.4/E/NDP52/图像_12856.jpg]

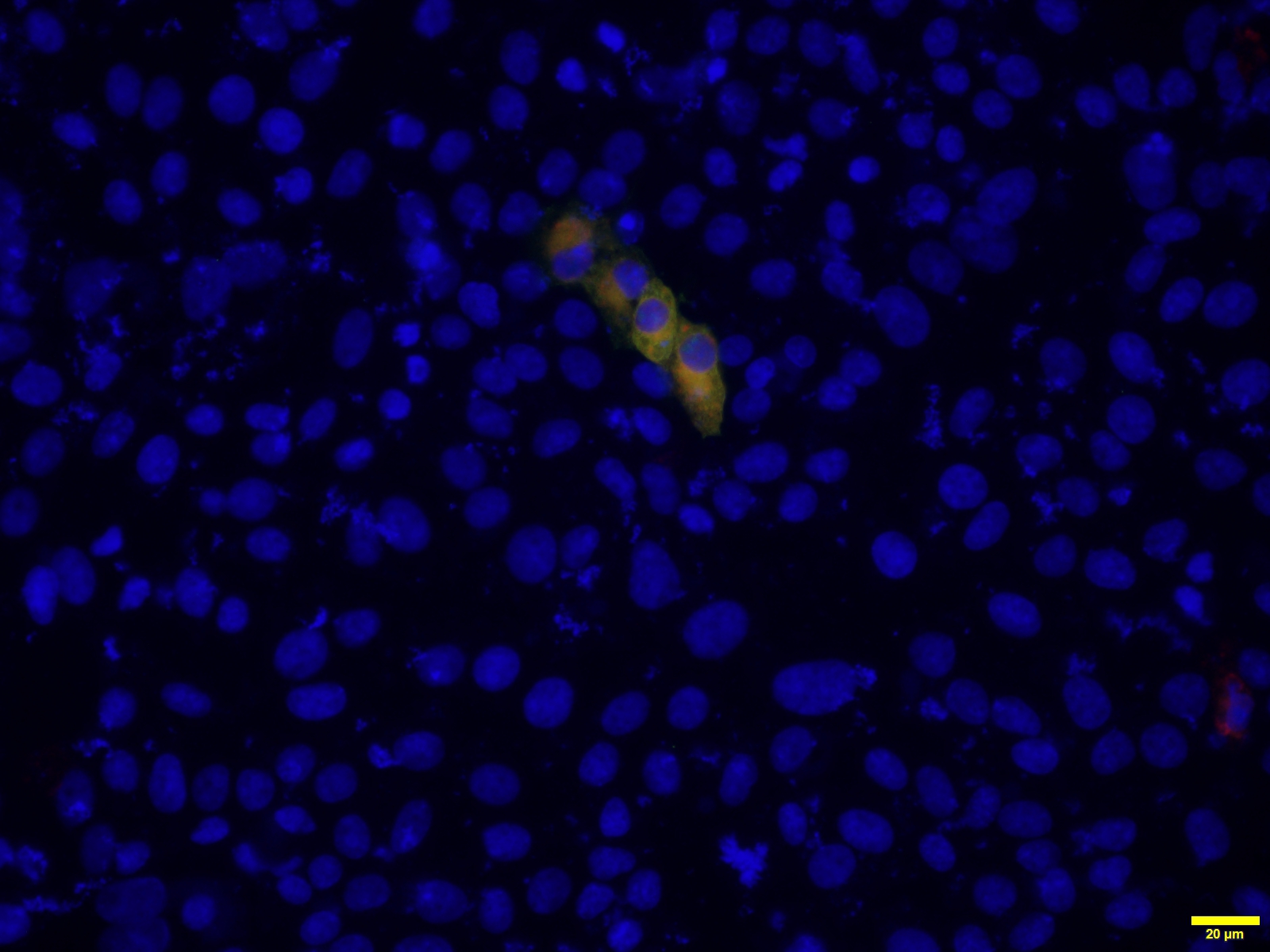

Supplement: Supplementary file 1 [file vetsci-12-00257-s001.zip › PABPC4 original blot images/Fig.4/E/NDP52+N/图像_03.jpg]

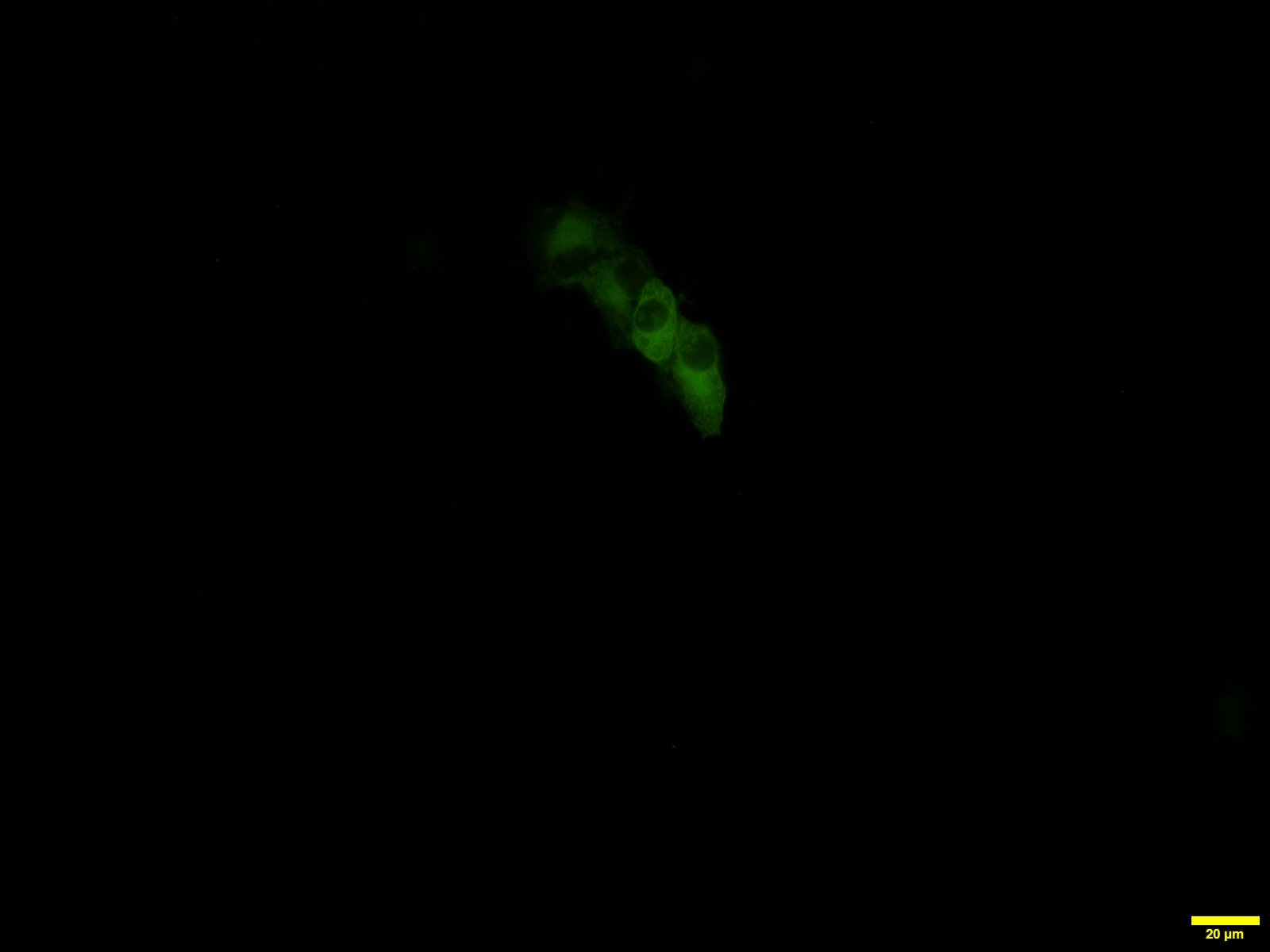

Supplement: Supplementary file 1 [file vetsci-12-00257-s001.zip › PABPC4 original blot images/Fig.4/E/NDP52+N/图像_12715.jpg]

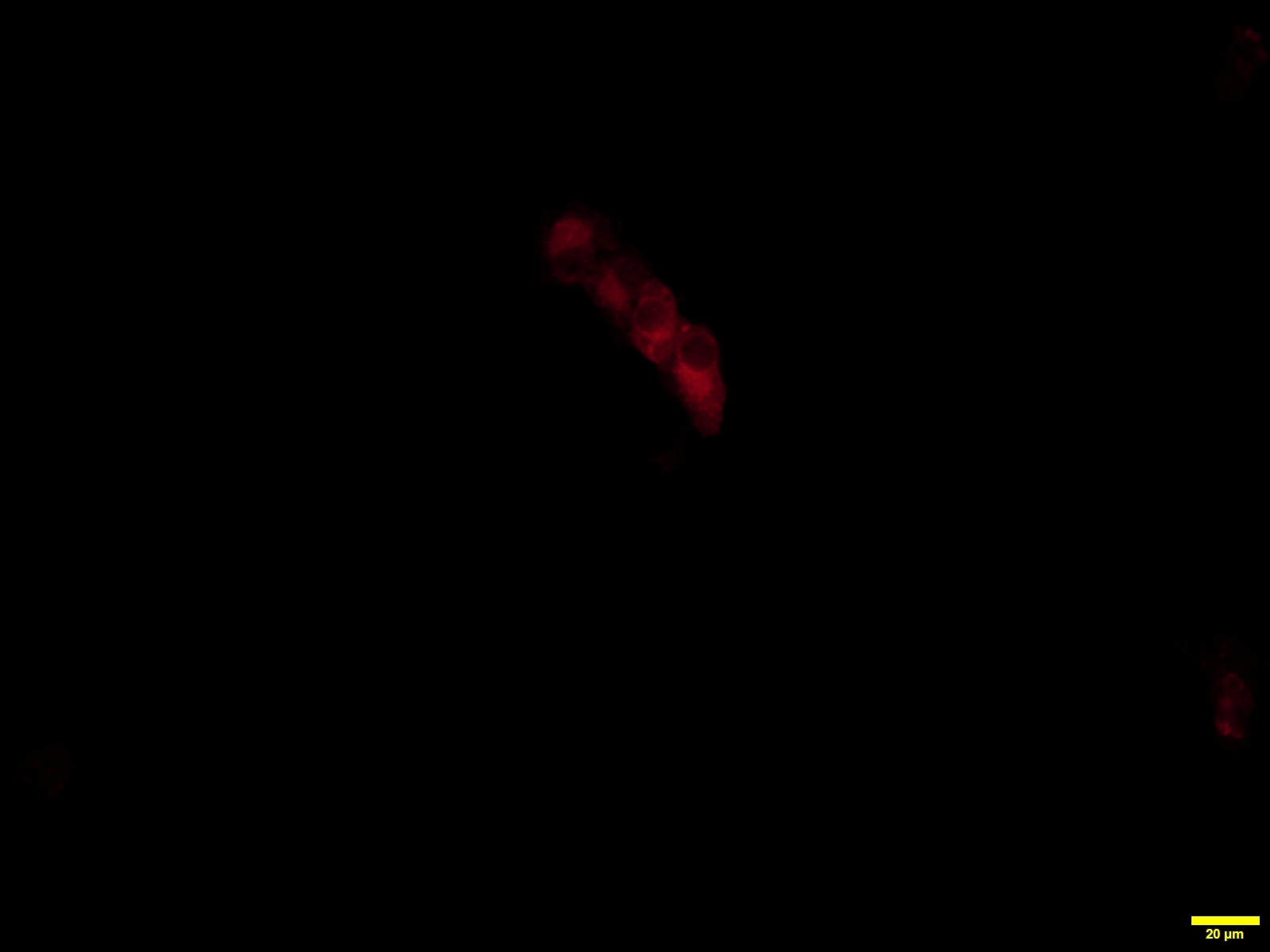

Supplement: Supplementary file 1 [file vetsci-12-00257-s001.zip › PABPC4 original blot images/Fig.4/E/NDP52+N/图像_12716.jpg]

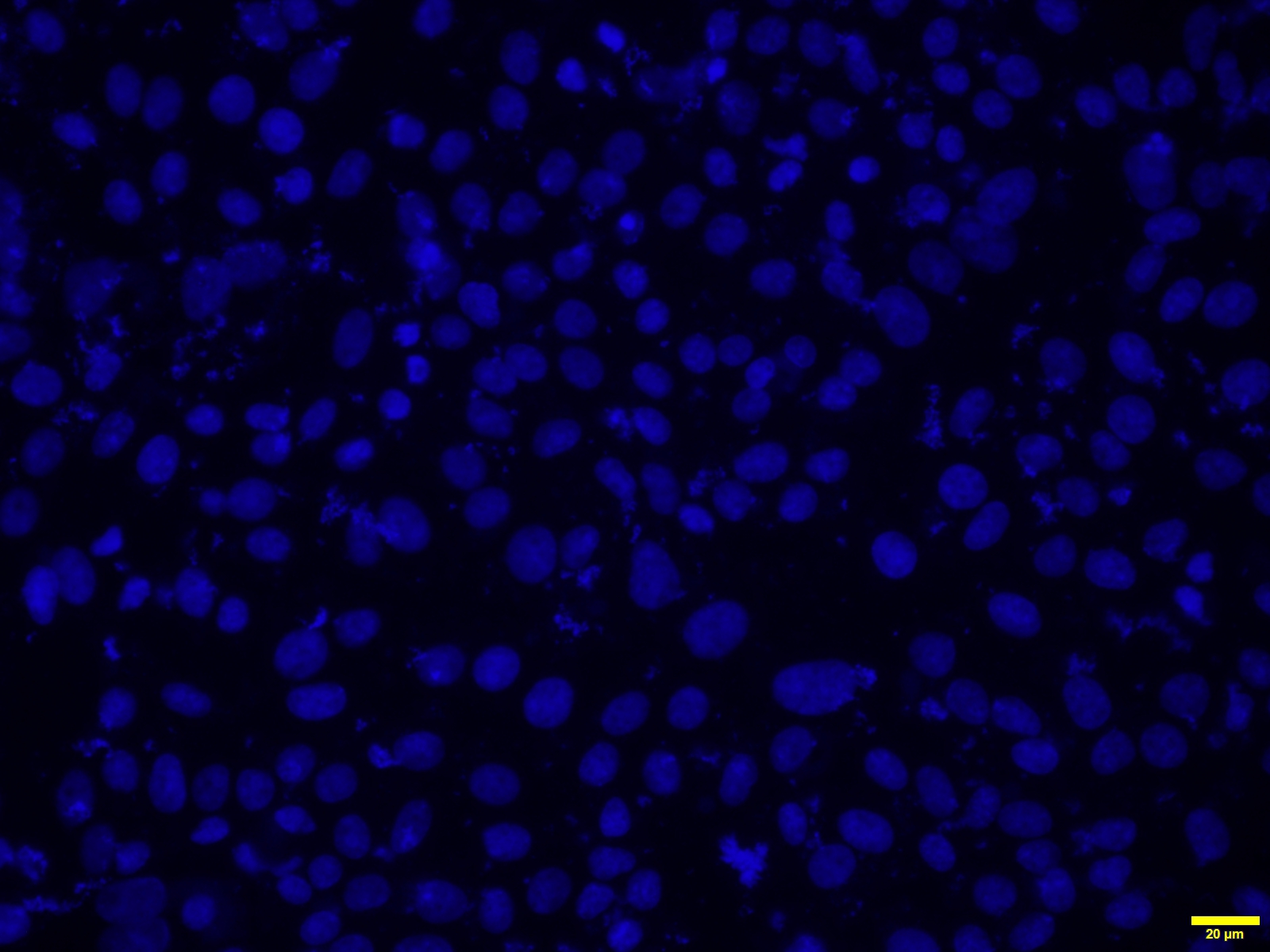

Supplement: Supplementary file 1 [file vetsci-12-00257-s001.zip › PABPC4 original blot images/Fig.4/E/NDP52+N/图像_12717.jpg]

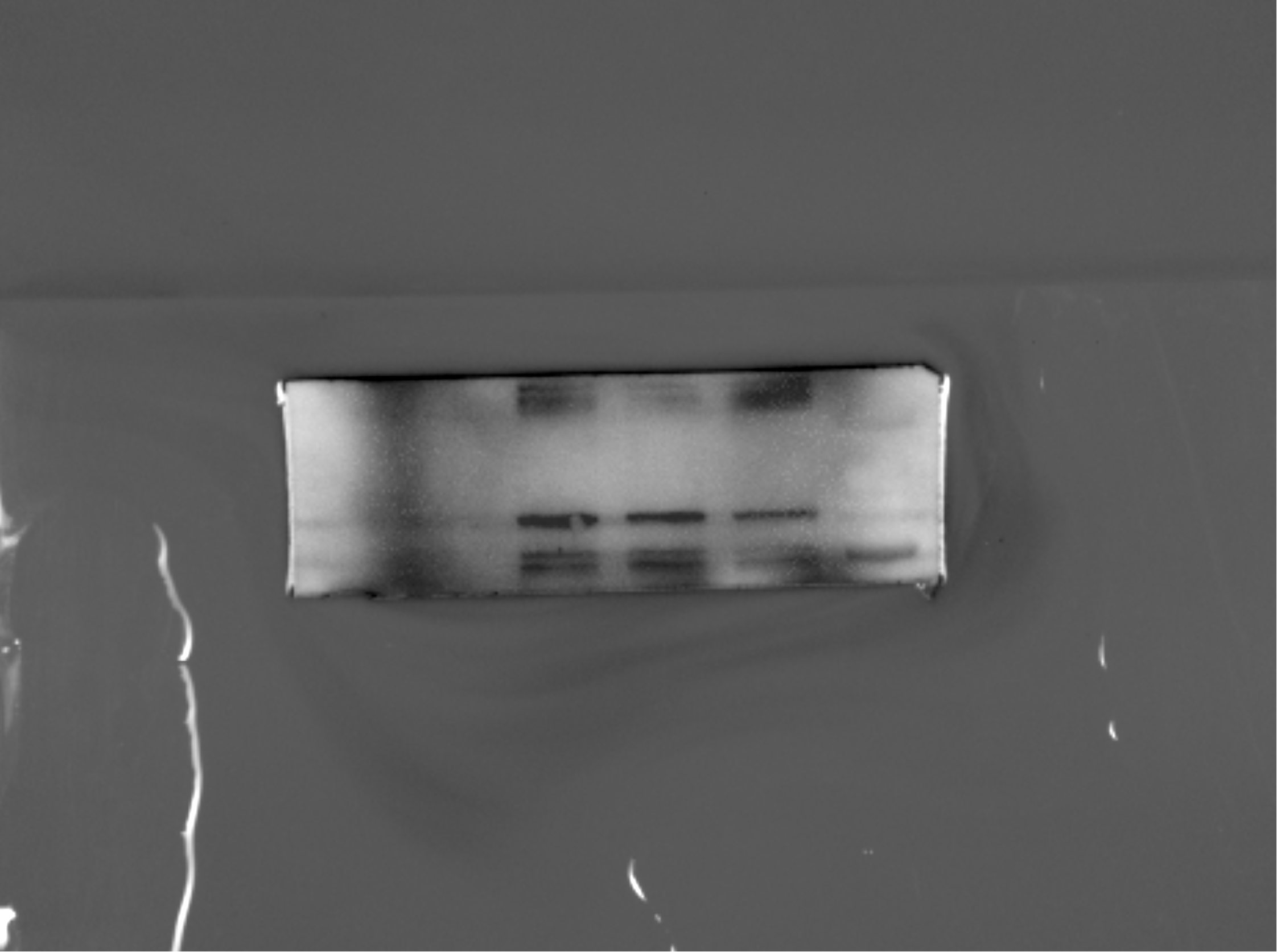

Supplement: Supplementary file 1 [file vetsci-12-00257-s001.zip › PABPC4 original blot images/Fig.4/F+G/FLAG/h.tif]

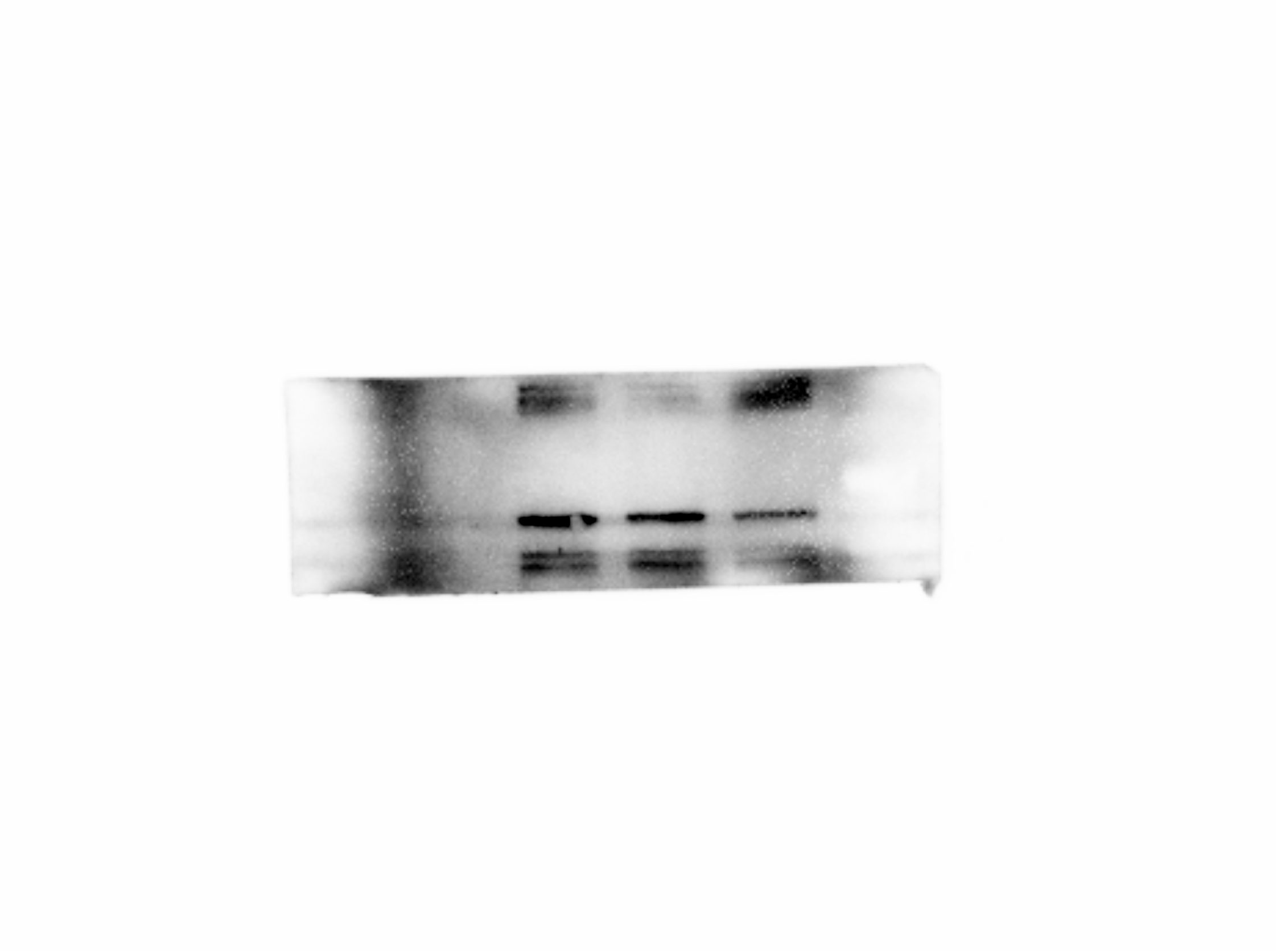

Supplement: Supplementary file 1 [file vetsci-12-00257-s001.zip › PABPC4 original blot images/Fig.4/F+G/FLAG/s.tif]
