# Supplementary figures and images for: PABPC4 Inhibits SADS-CoV Replication by Degrading the Nucleocapsid Protein Through Selective Autophagy (part 1 of 2)
Source: Vet Sci. 2025 Mar 10;12(3):257. doi: 10.3390/vetsci12030257 (PMC11946123; doi:10.3390/vetsci12030257)

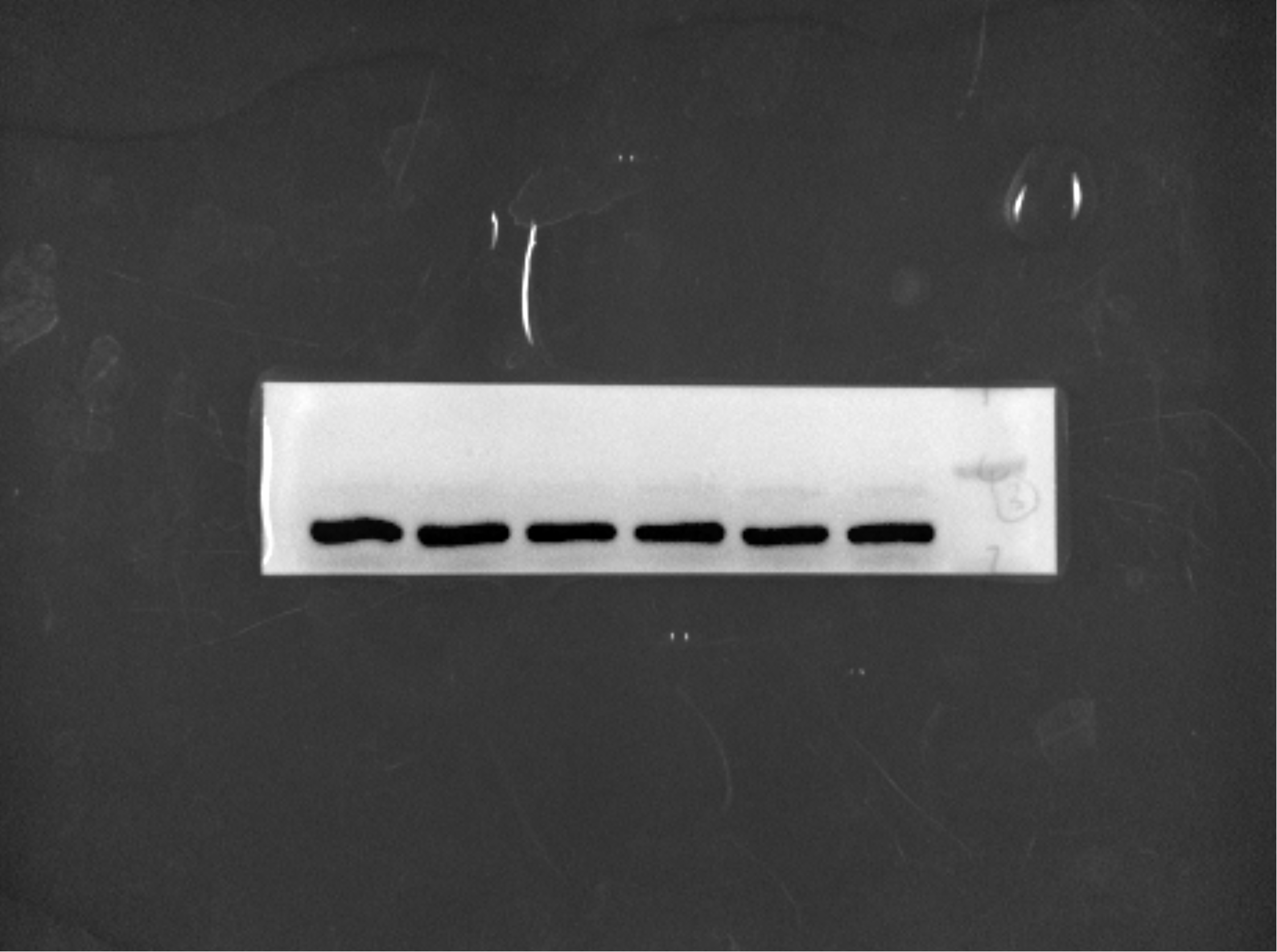

Supplement: Supplementary file 1 [file vetsci-12-00257-s001.zip › PABPC4 original blot images/Fig.1/A+B/gapdh/h.tif]

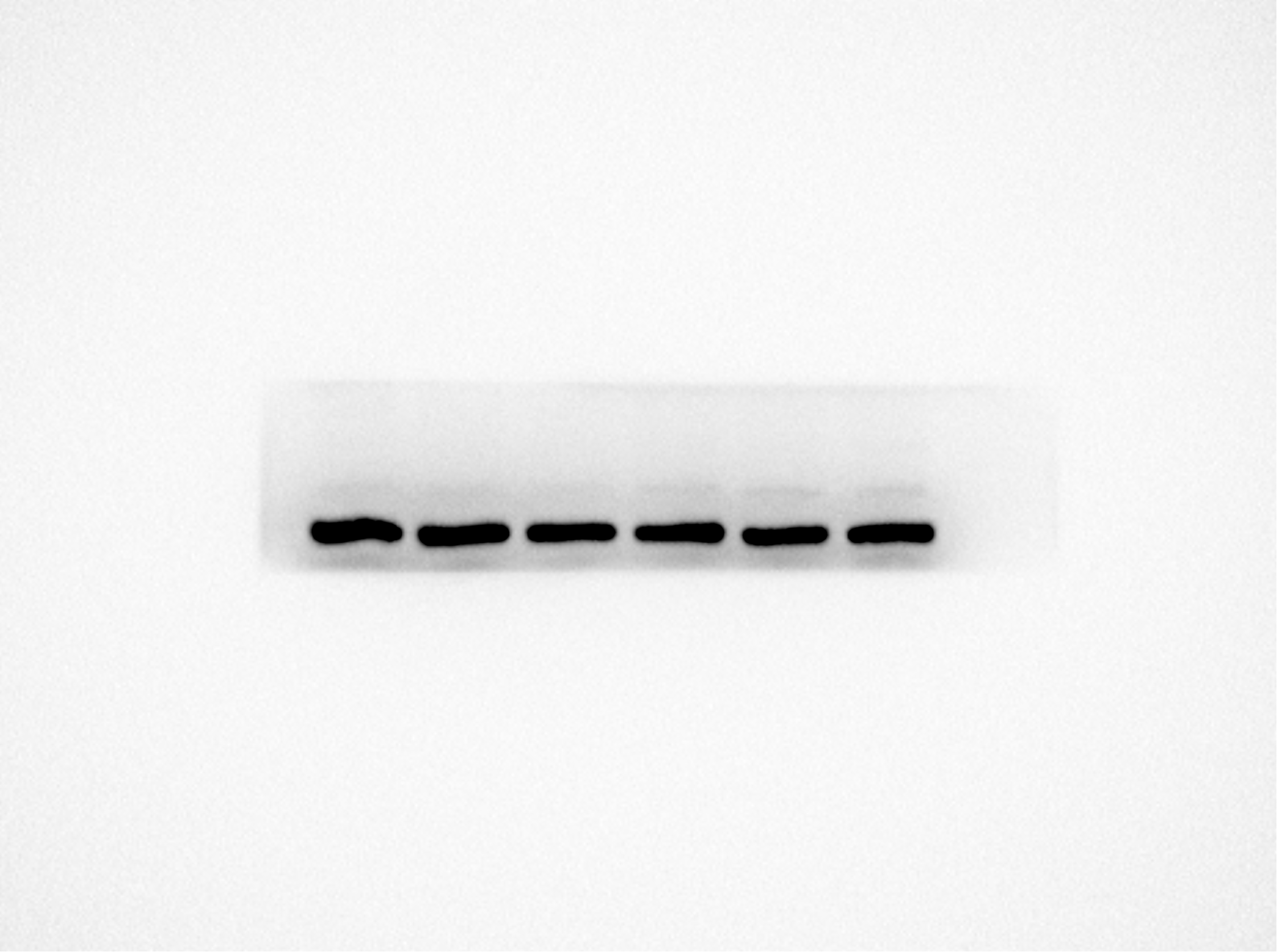

Supplement: Supplementary file 1 [file vetsci-12-00257-s001.zip › PABPC4 original blot images/Fig.1/A+B/gapdh/s.tif]

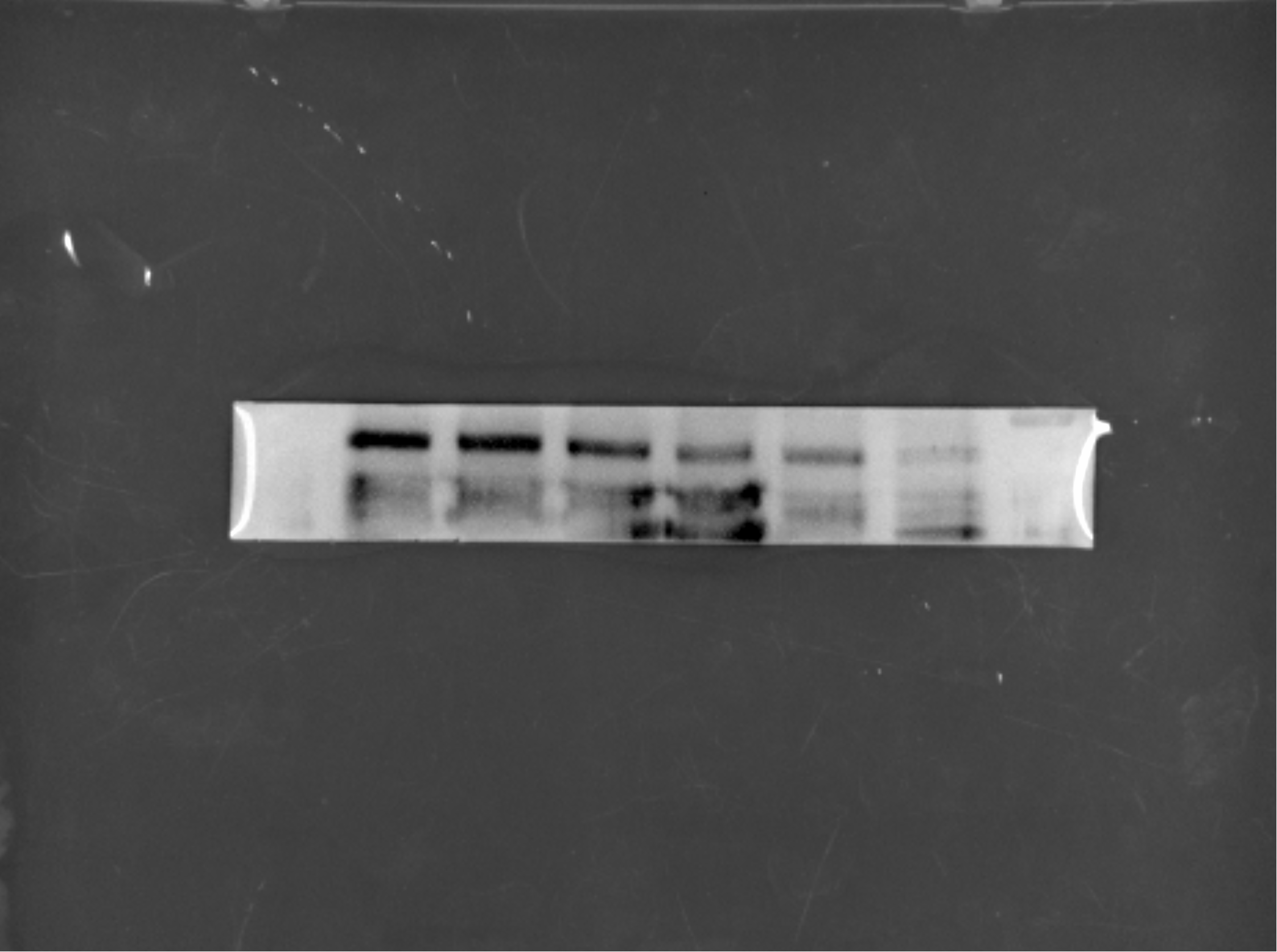

Supplement: Supplementary file 1 [file vetsci-12-00257-s001.zip › PABPC4 original blot images/Fig.1/A+B/PABPC4/h.tif]

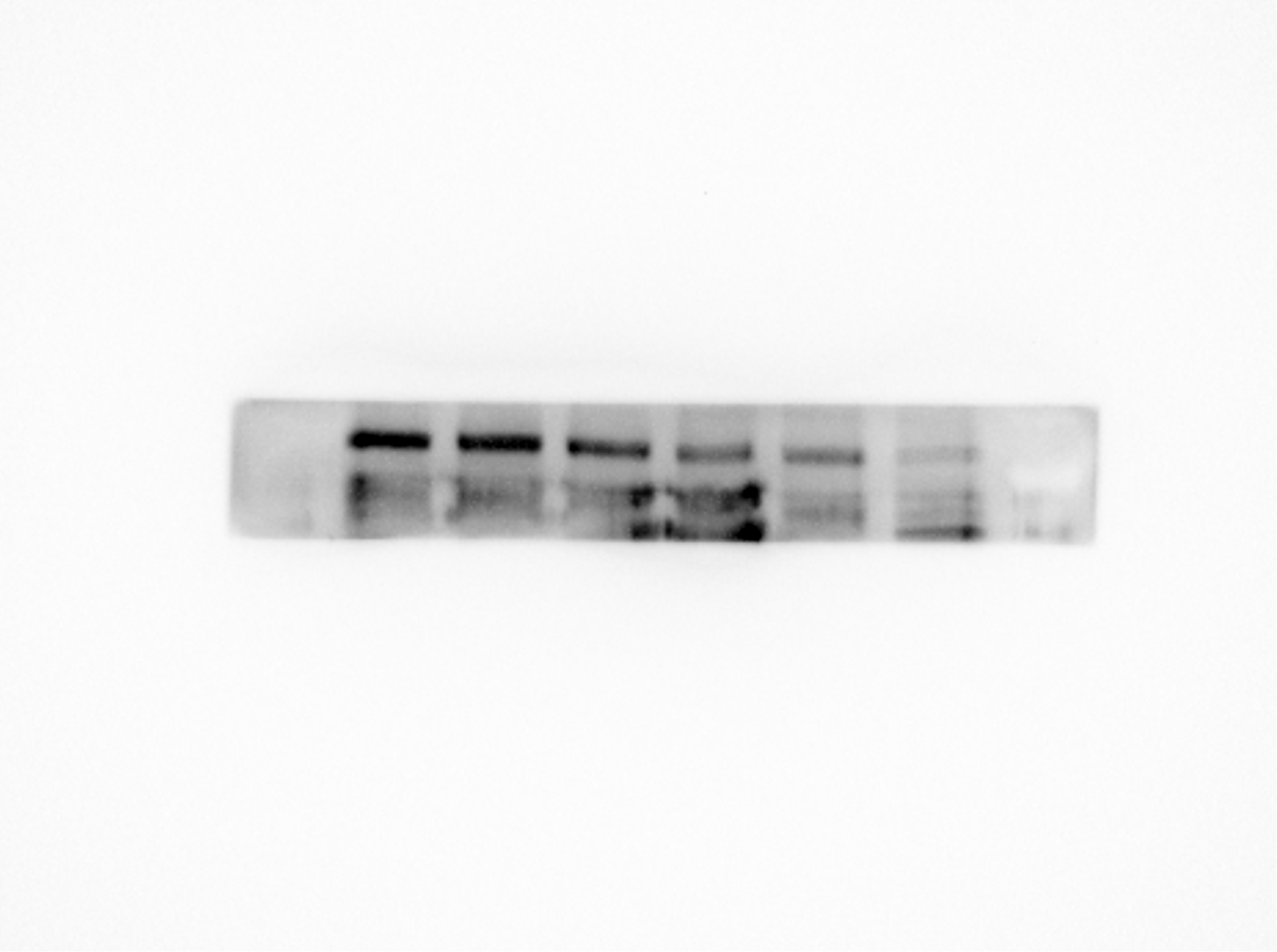

Supplement: Supplementary file 1 [file vetsci-12-00257-s001.zip › PABPC4 original blot images/Fig.1/A+B/PABPC4/s.tif]

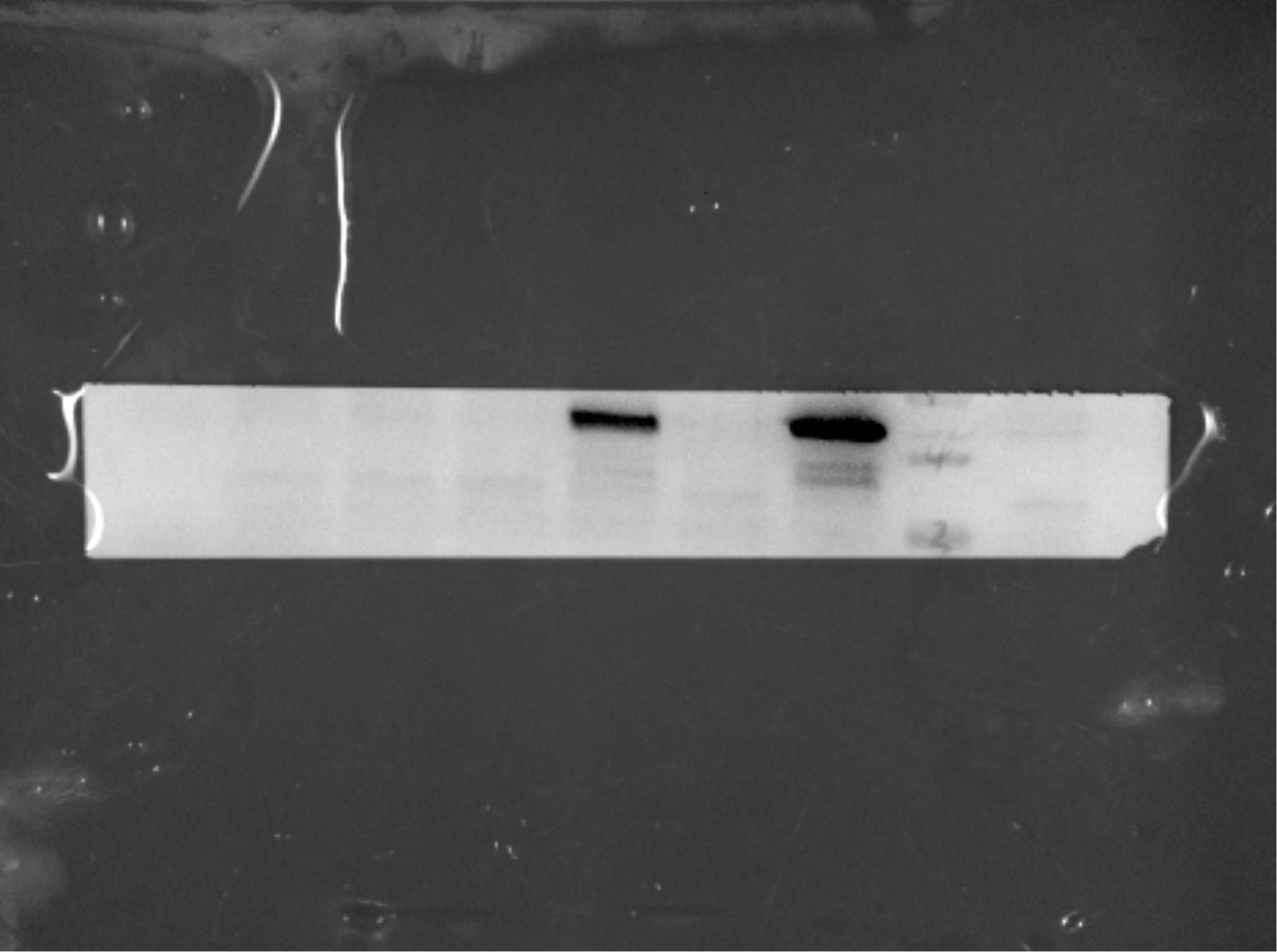

Supplement: Supplementary file 1 [file vetsci-12-00257-s001.zip › PABPC4 original blot images/Fig.1/A+B/SADS-CoV-N/H.tif]

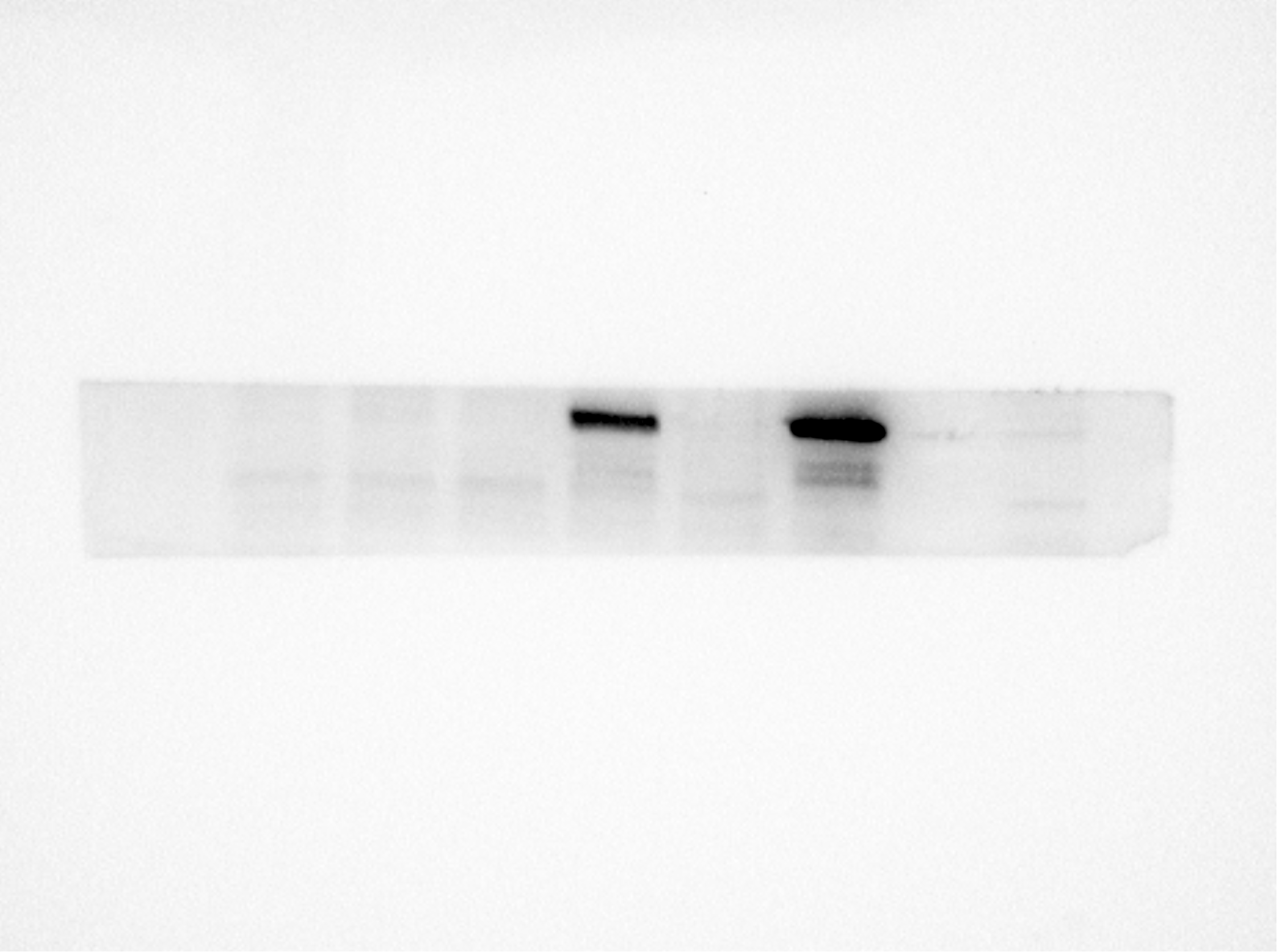

Supplement: Supplementary file 1 [file vetsci-12-00257-s001.zip › PABPC4 original blot images/Fig.1/A+B/SADS-CoV-N/S.tif]

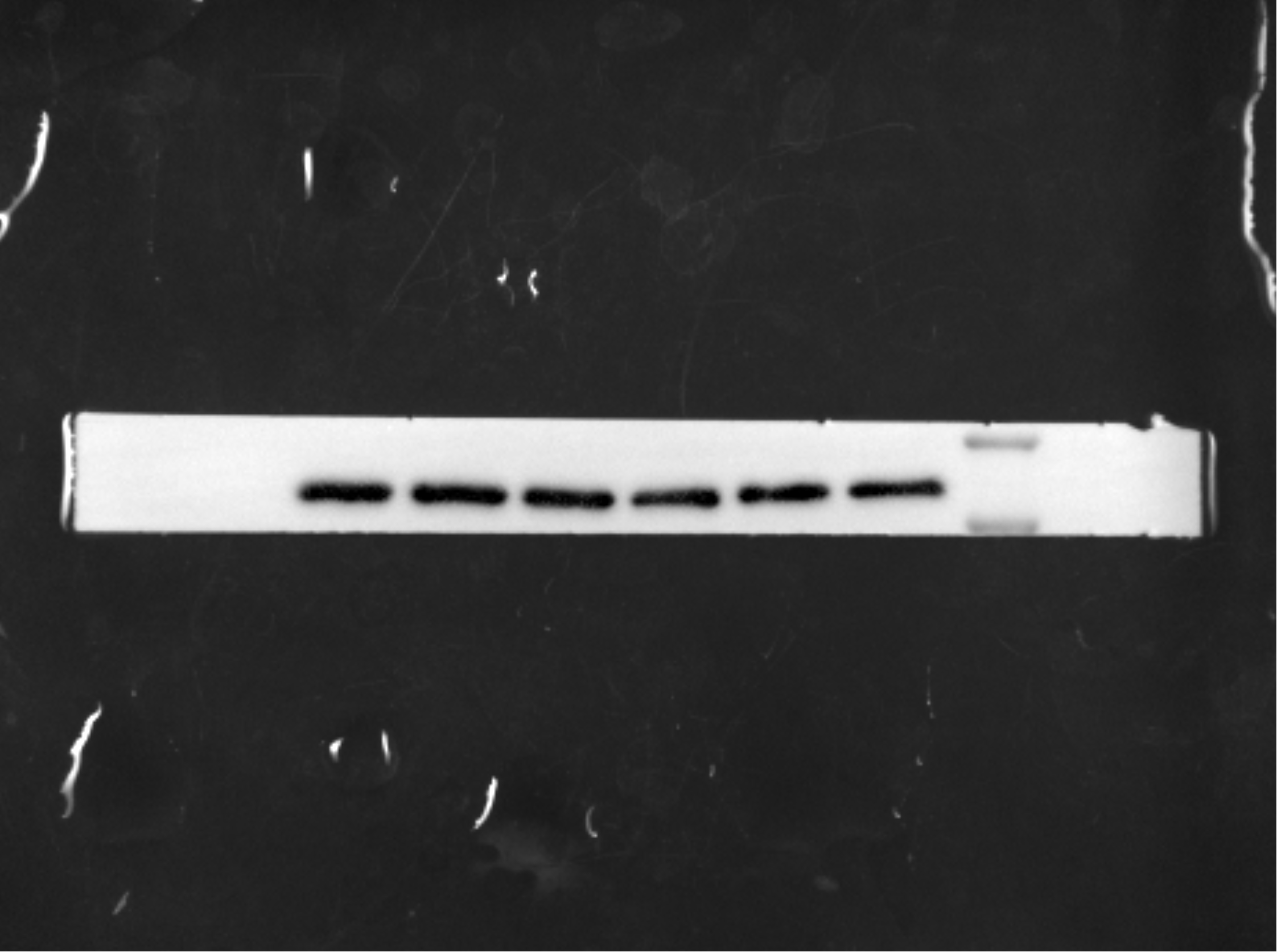

Supplement: Supplementary file 1 [file vetsci-12-00257-s001.zip › PABPC4 original blot images/Fig.1/C+D/gapdh/merge.tif]

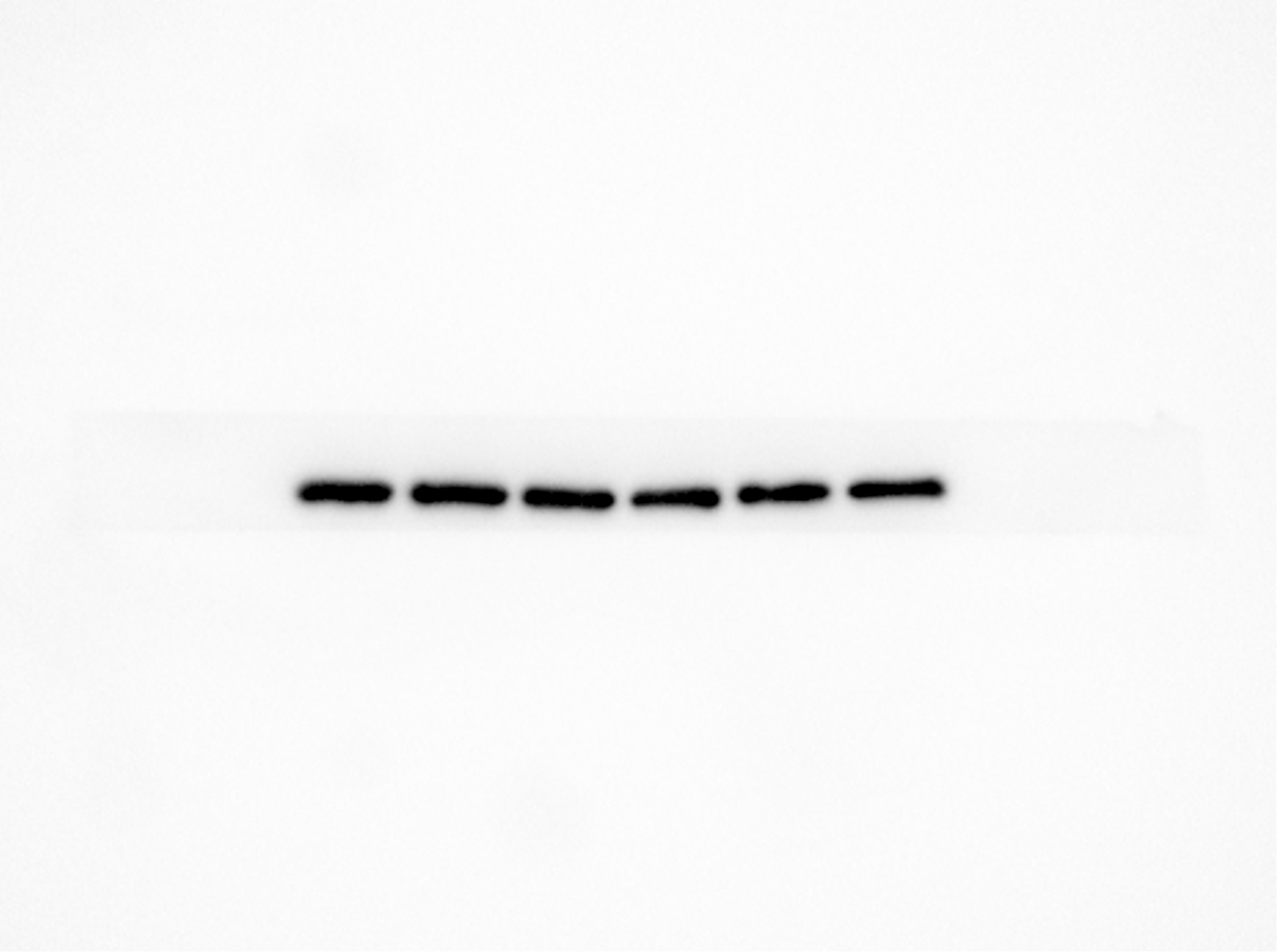

Supplement: Supplementary file 1 [file vetsci-12-00257-s001.zip › PABPC4 original blot images/Fig.1/C+D/gapdh/s.tif]

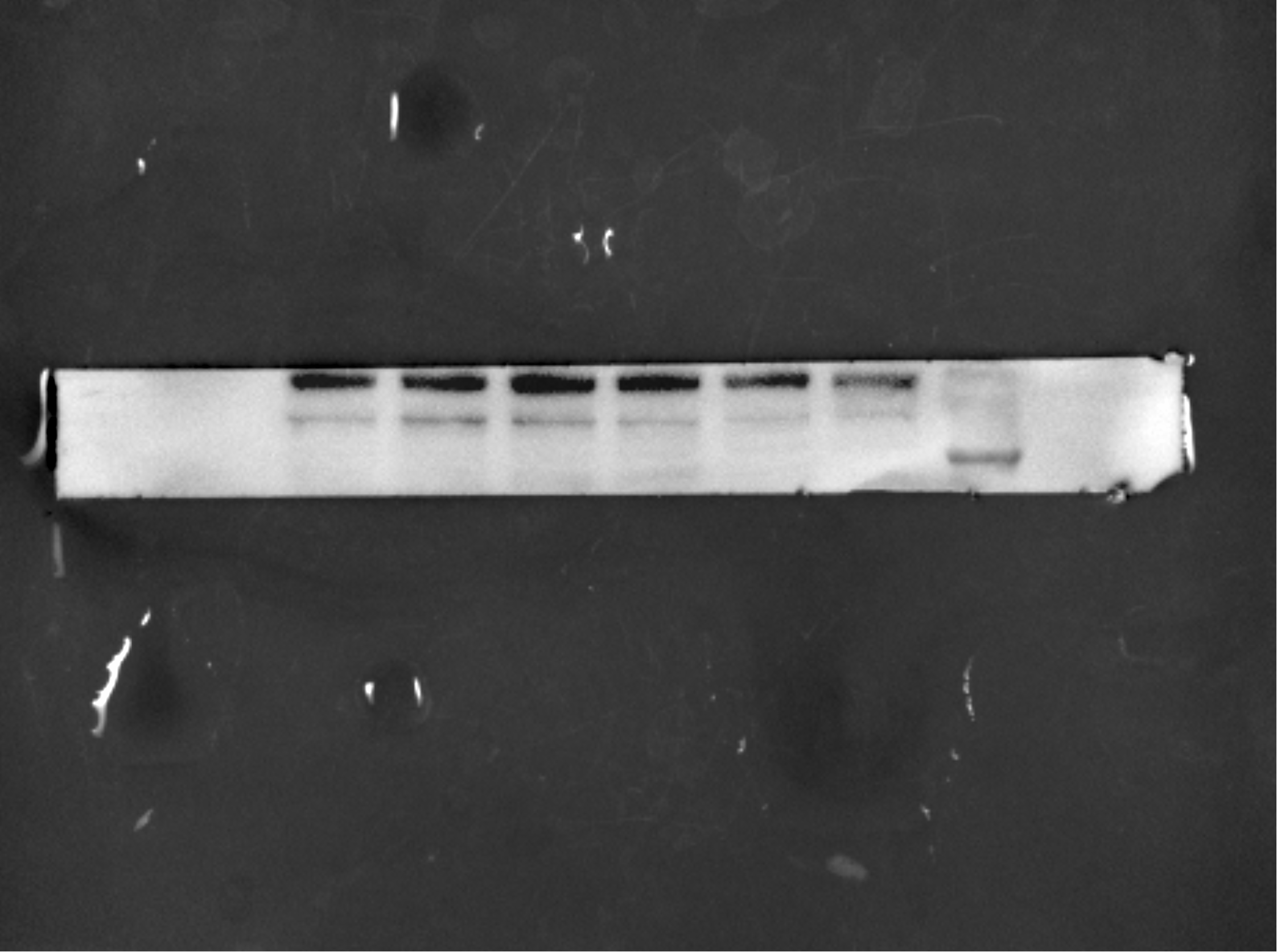

Supplement: Supplementary file 1 [file vetsci-12-00257-s001.zip › PABPC4 original blot images/Fig.1/C+D/pabpc4/merge.tif]

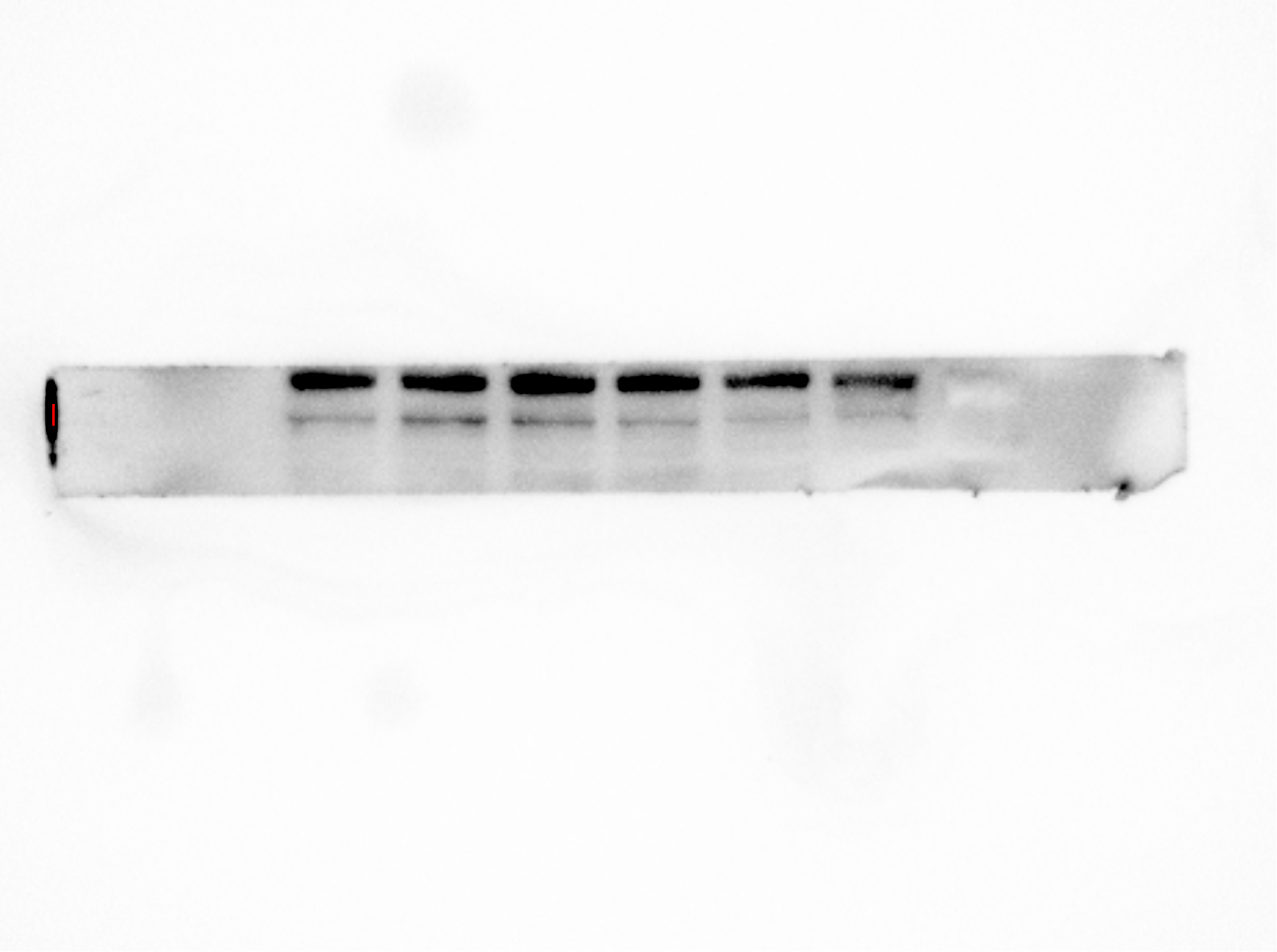

Supplement: Supplementary file 1 [file vetsci-12-00257-s001.zip › PABPC4 original blot images/Fig.1/C+D/pabpc4/s.tif]

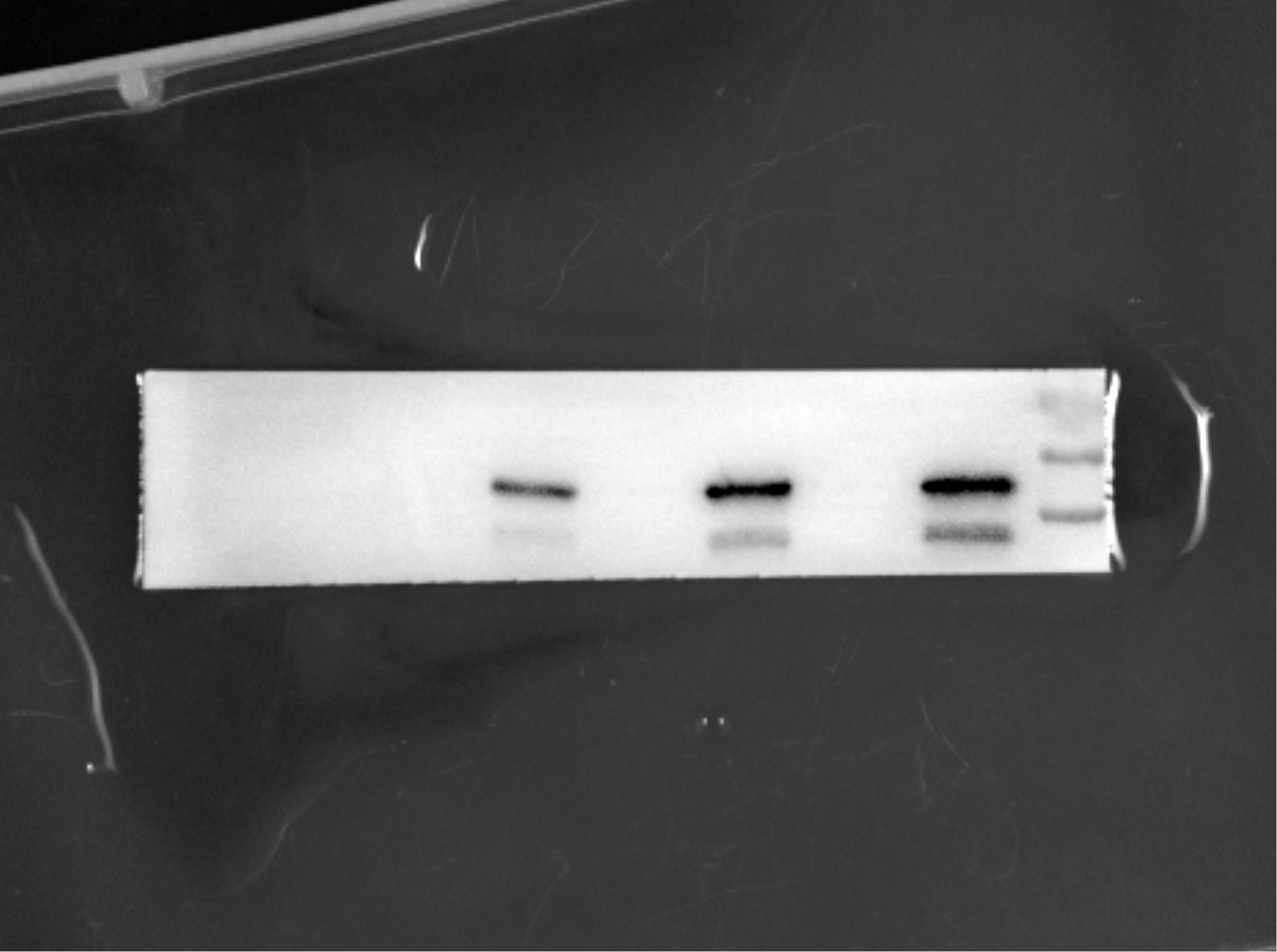

Supplement: Supplementary file 1 [file vetsci-12-00257-s001.zip › PABPC4 original blot images/Fig.1/C+D/SADS-CoV-n/h.tif]

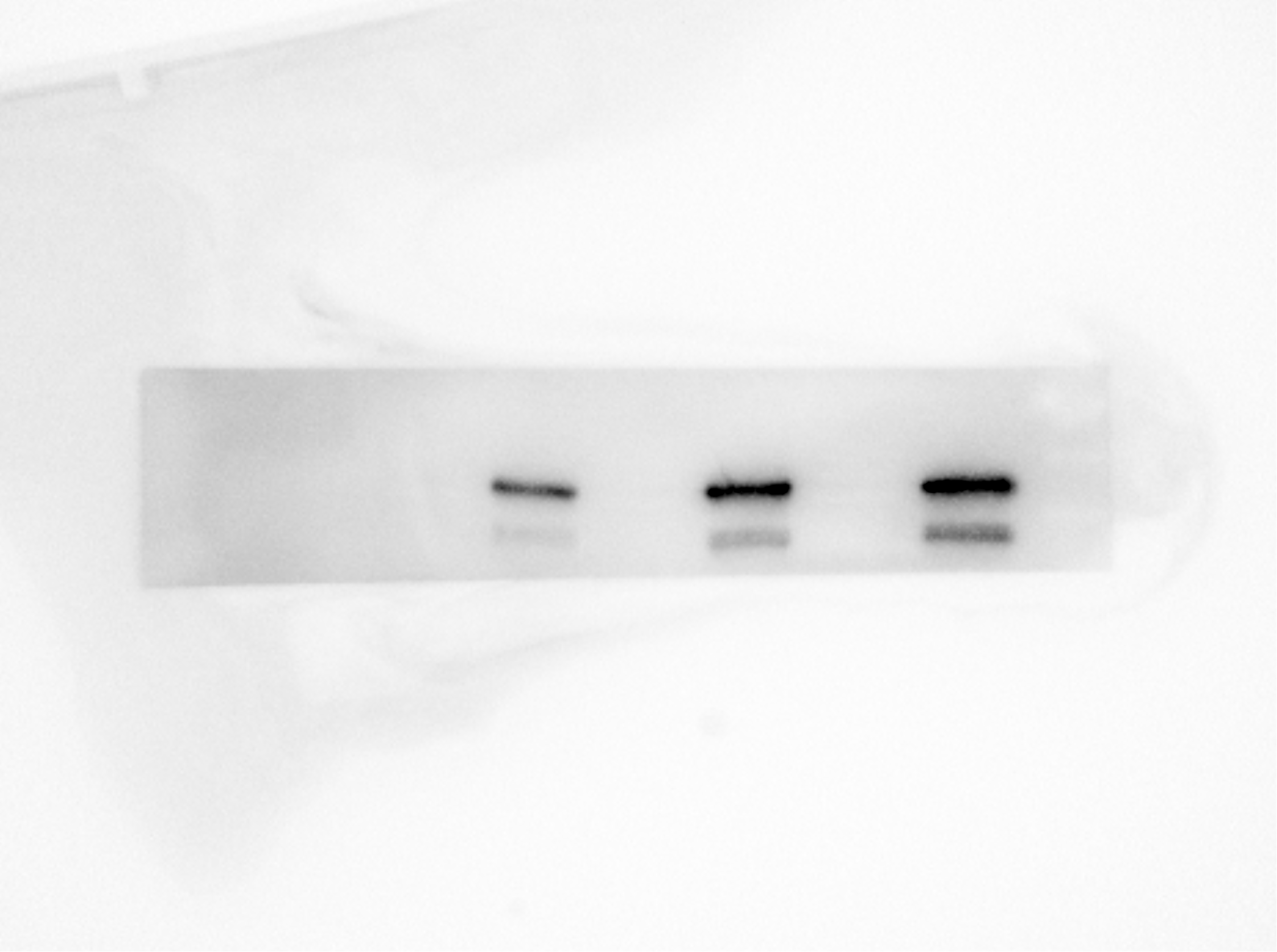

Supplement: Supplementary file 1 [file vetsci-12-00257-s001.zip › PABPC4 original blot images/Fig.1/C+D/SADS-CoV-n/s.tif]

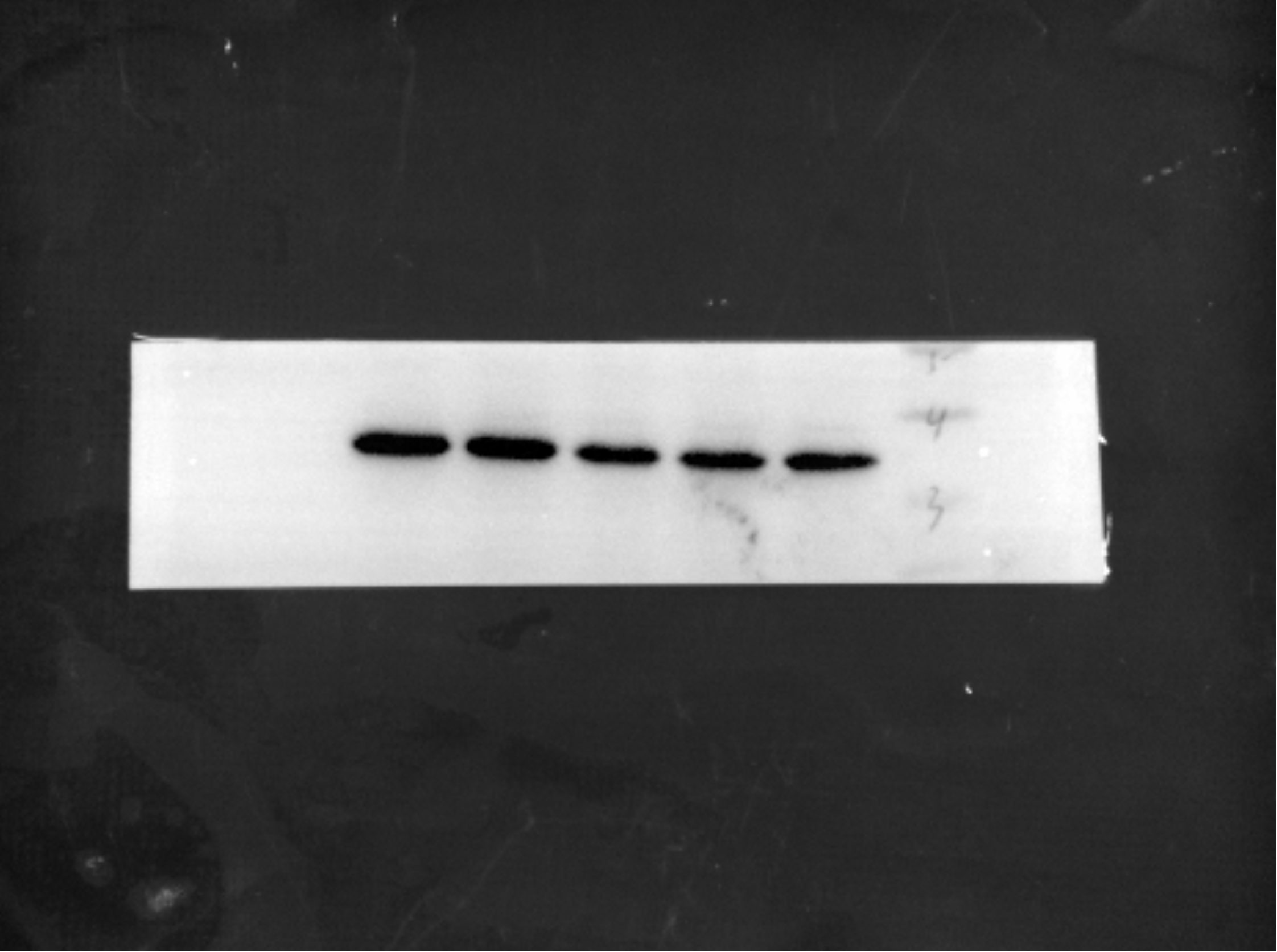

Supplement: Supplementary file 1 [file vetsci-12-00257-s001.zip › PABPC4 original blot images/Fig.2/A/gapdh/merge.tif]

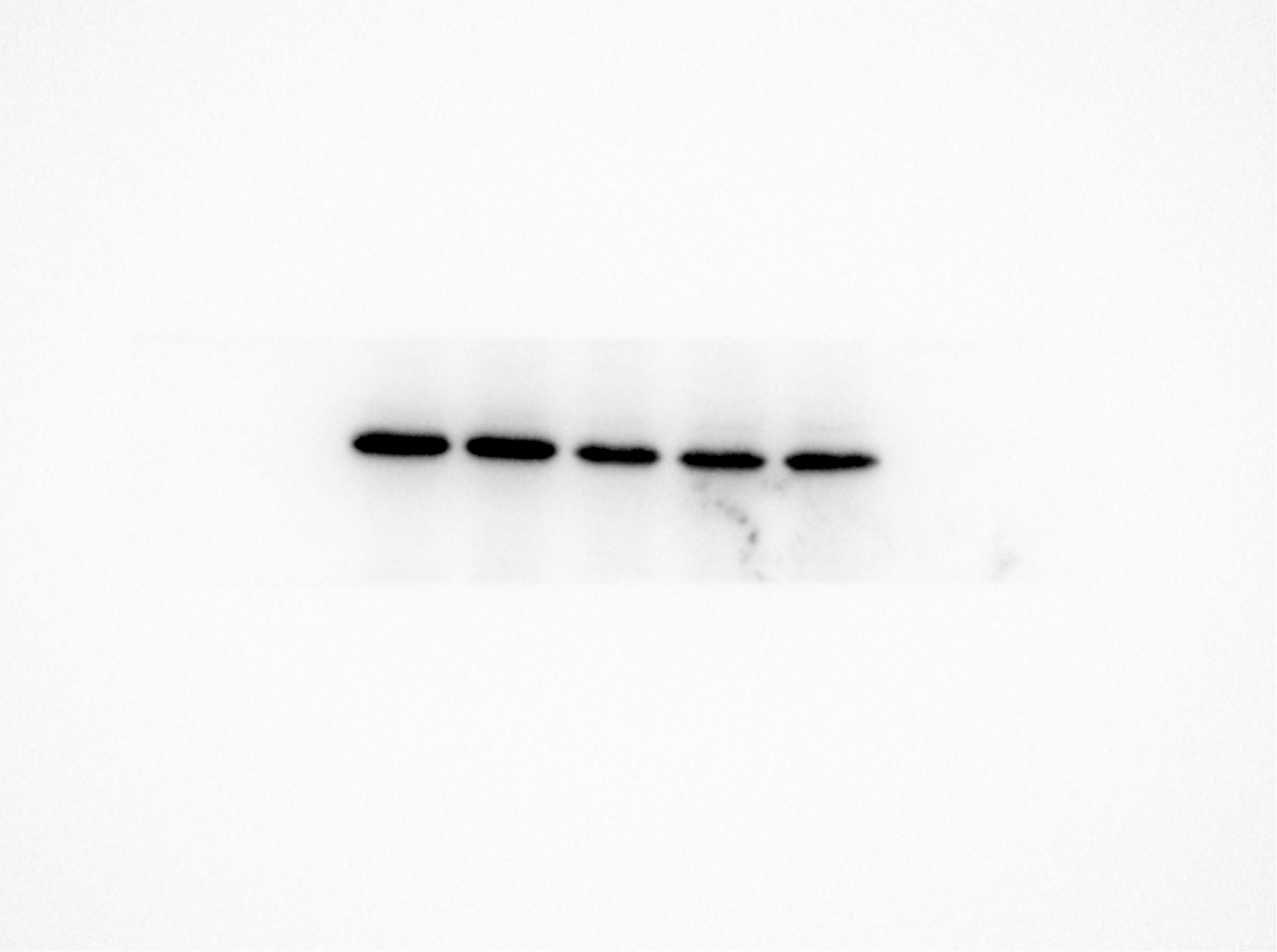

Supplement: Supplementary file 1 [file vetsci-12-00257-s001.zip › PABPC4 original blot images/Fig.2/A/gapdh/shiyantu.tif]

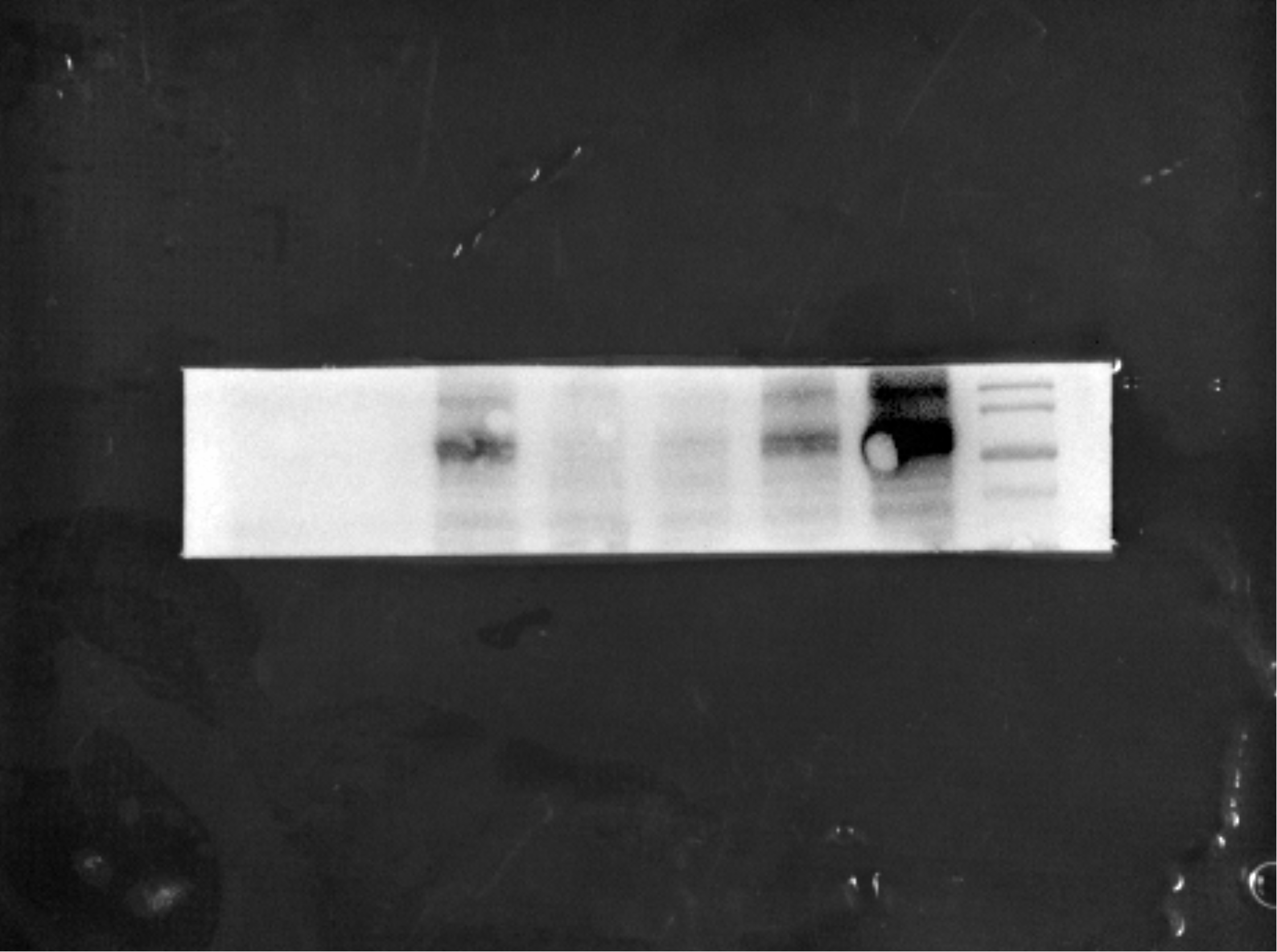

Supplement: Supplementary file 1 [file vetsci-12-00257-s001.zip › PABPC4 original blot images/Fig.2/A/ha/merge.tif]

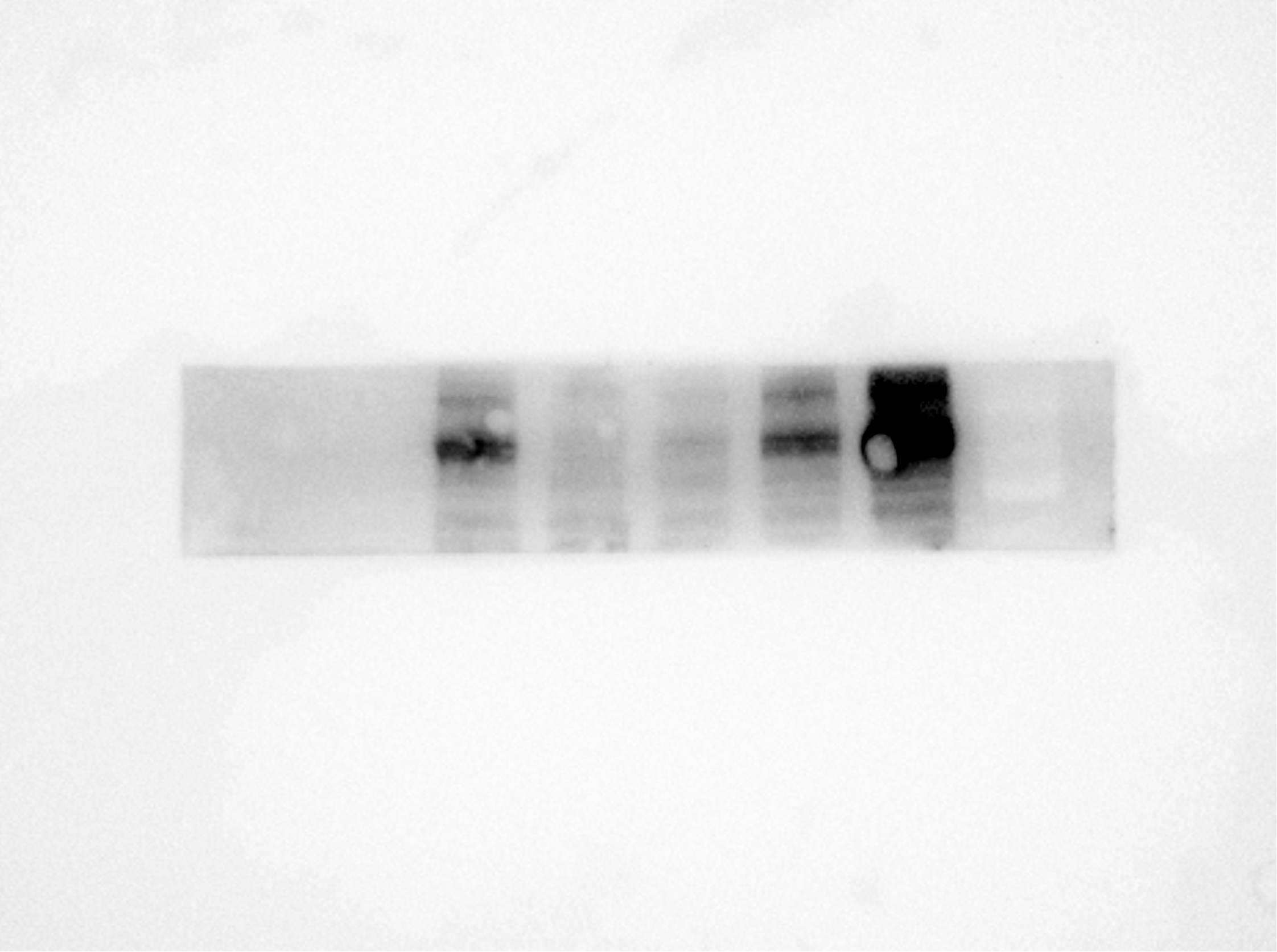

Supplement: Supplementary file 1 [file vetsci-12-00257-s001.zip › PABPC4 original blot images/Fig.2/A/ha/shiyantu.tif]

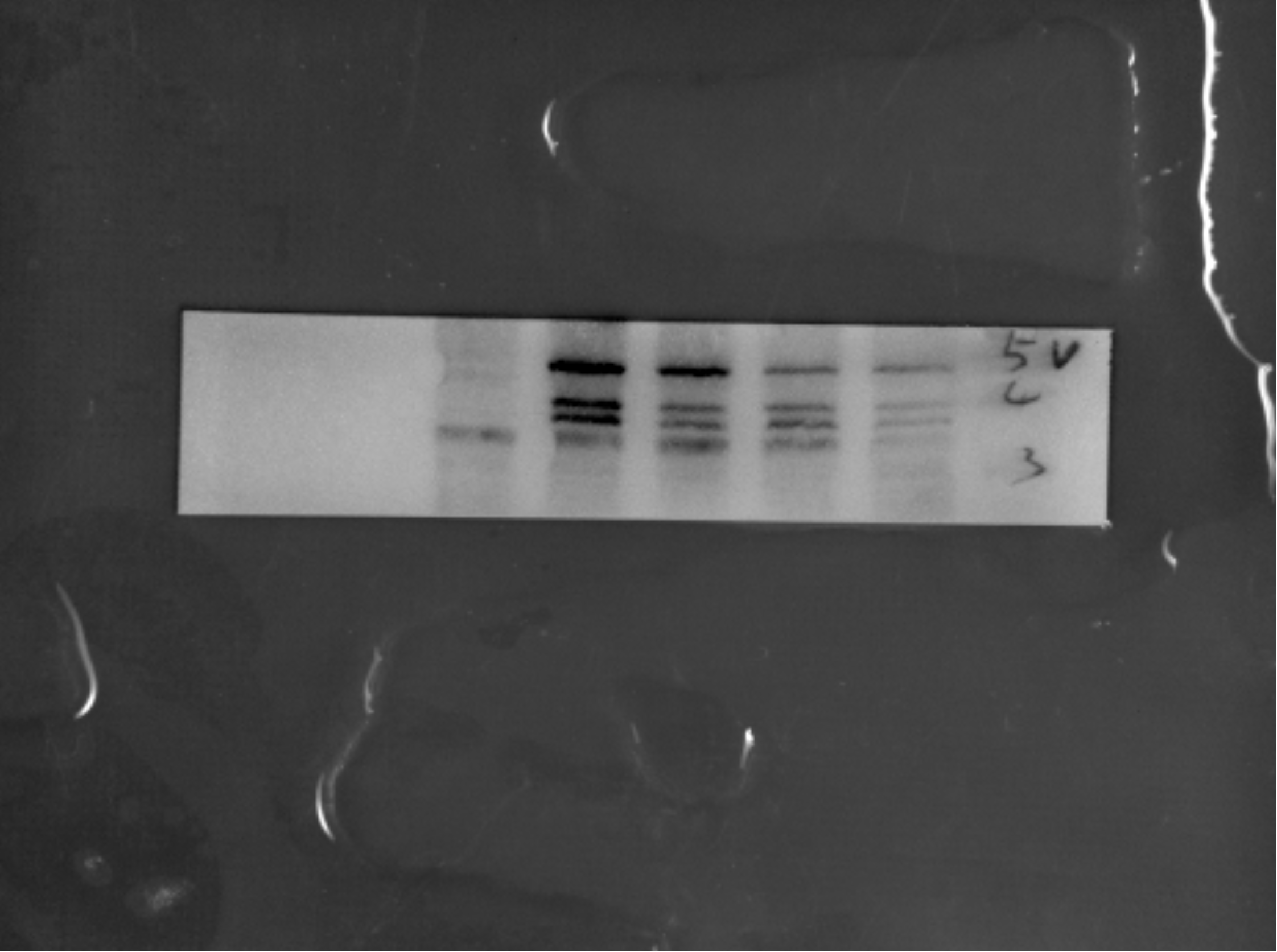

Supplement: Supplementary file 1 [file vetsci-12-00257-s001.zip › PABPC4 original blot images/Fig.2/A/SADS-CoV-n/merge.tif]

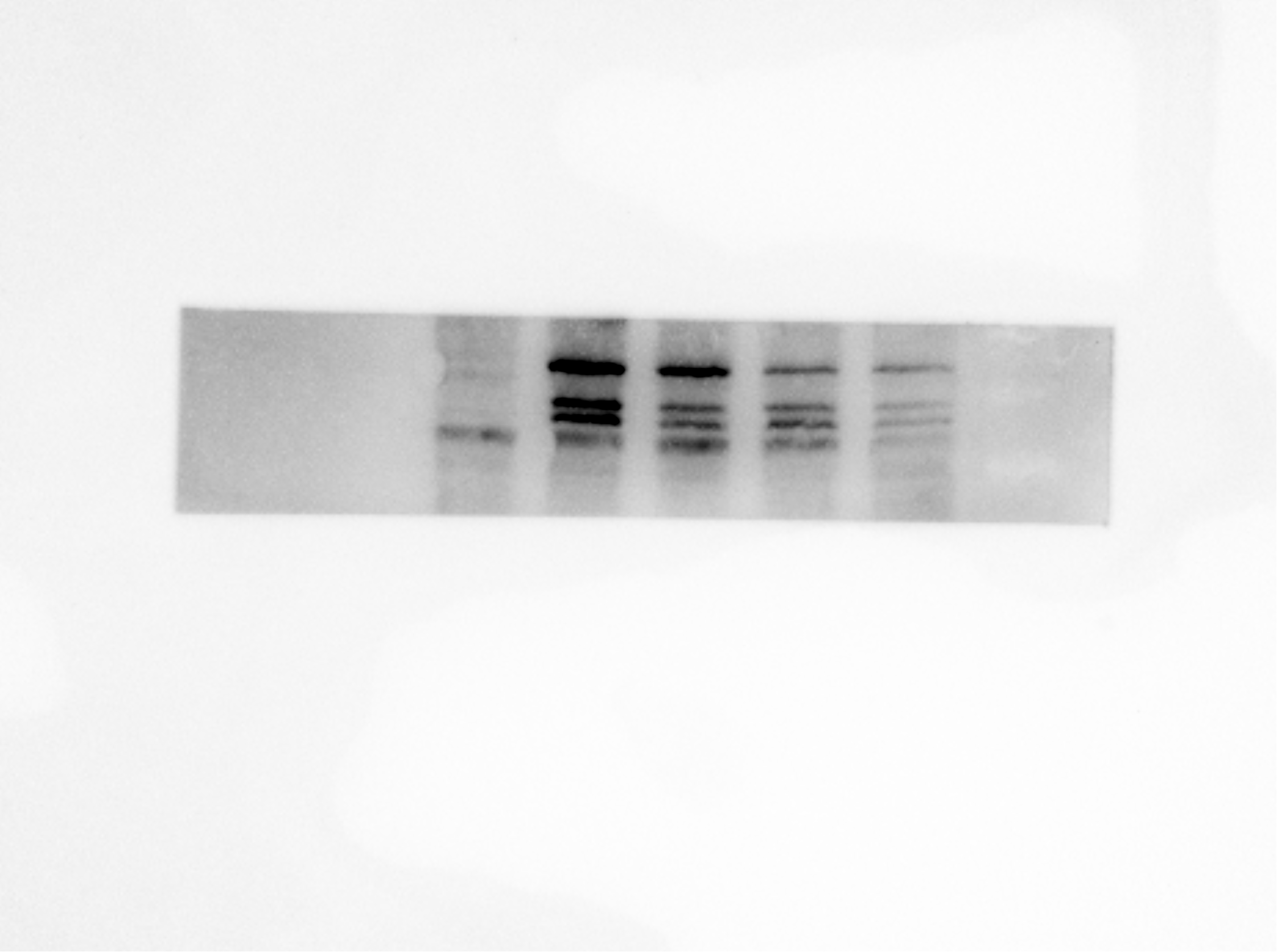

Supplement: Supplementary file 1 [file vetsci-12-00257-s001.zip › PABPC4 original blot images/Fig.2/A/SADS-CoV-n/shiyantu.tif]

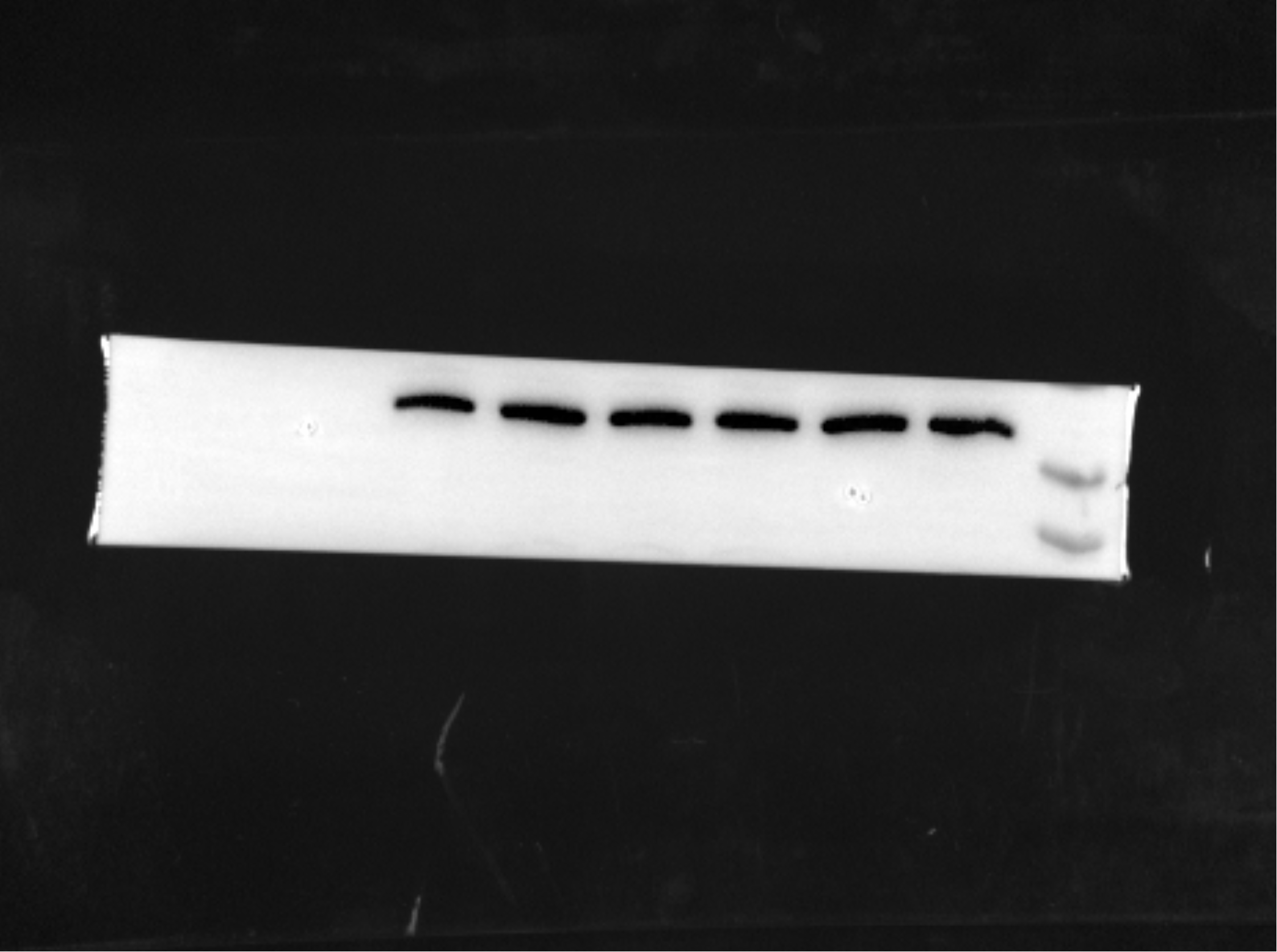

Supplement: Supplementary file 1 [file vetsci-12-00257-s001.zip › PABPC4 original blot images/Fig.2/B/gapdh/h.tif]

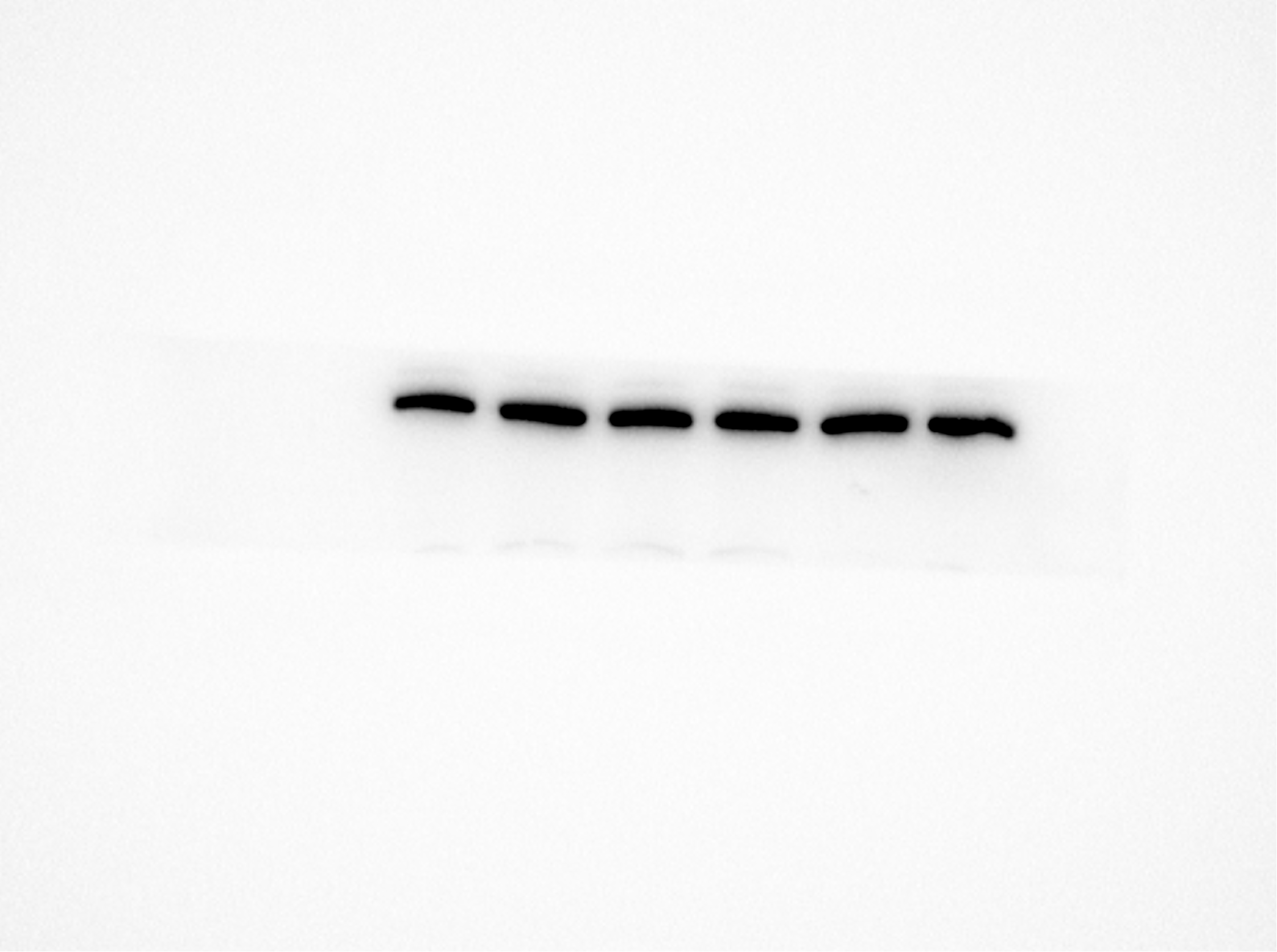

Supplement: Supplementary file 1 [file vetsci-12-00257-s001.zip › PABPC4 original blot images/Fig.2/B/gapdh/m.tif]

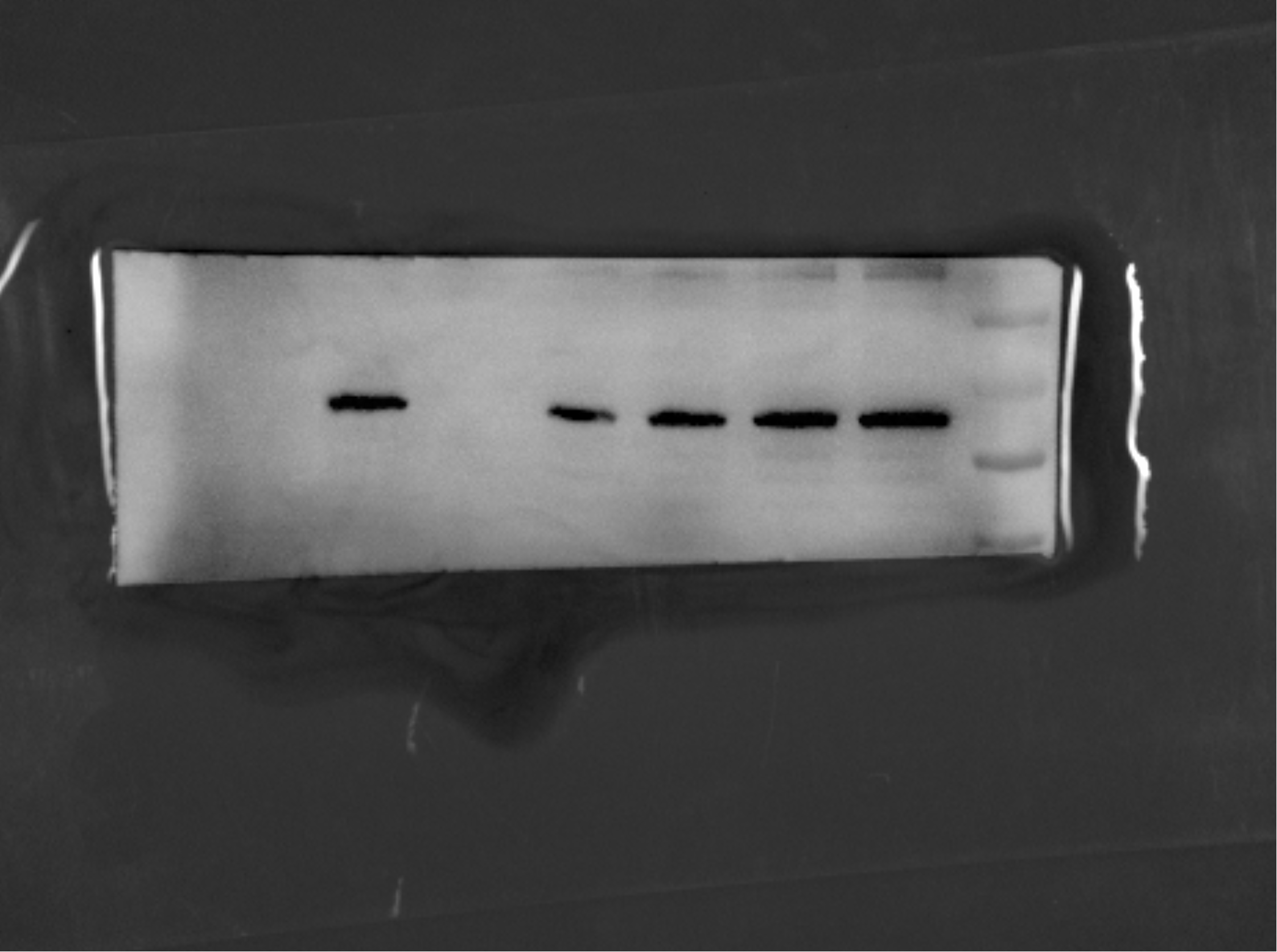

Supplement: Supplementary file 1 [file vetsci-12-00257-s001.zip › PABPC4 original blot images/Fig.2/B/ha/h.tif]

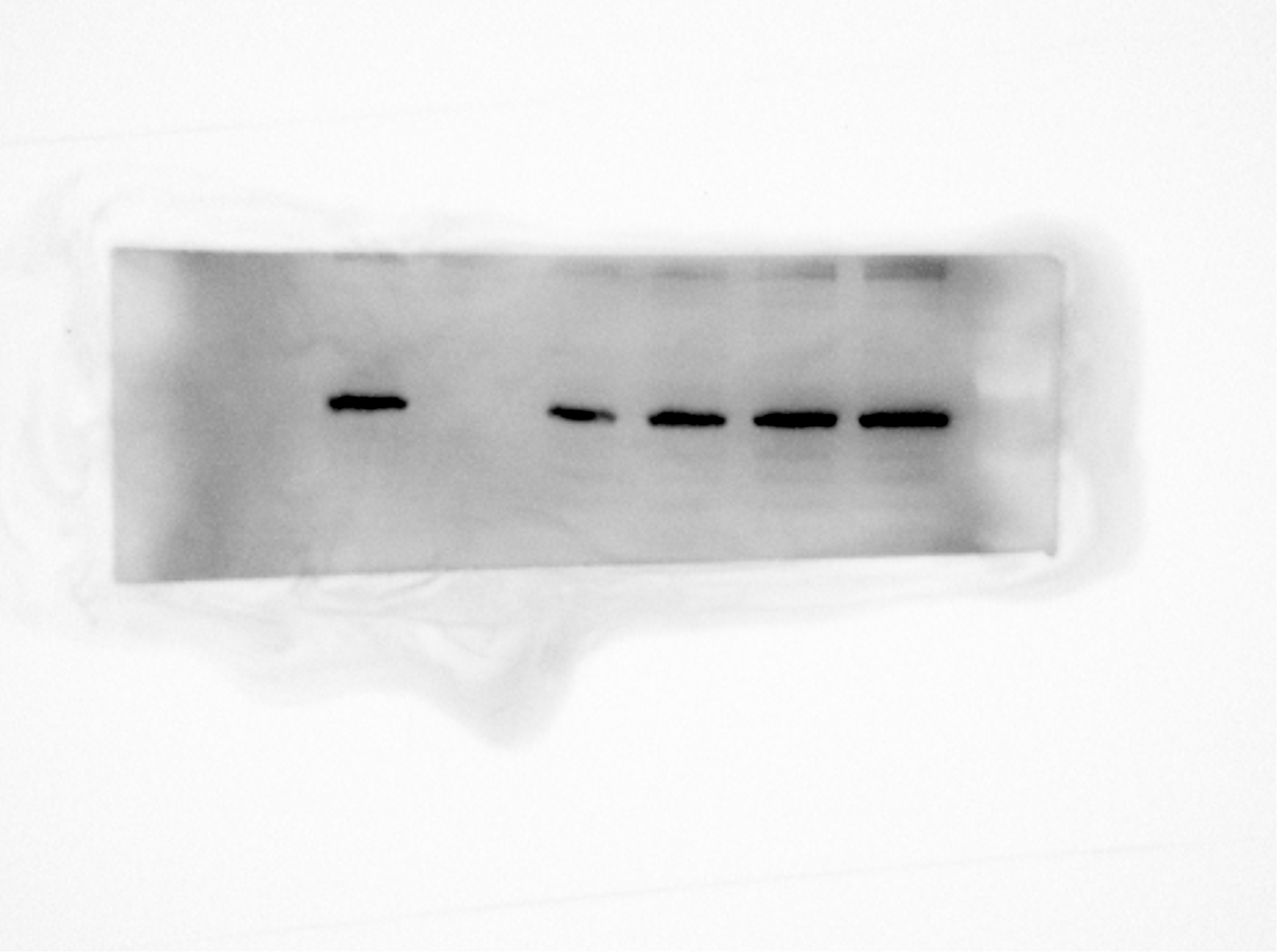

Supplement: Supplementary file 1 [file vetsci-12-00257-s001.zip › PABPC4 original blot images/Fig.2/B/ha/s.tif]

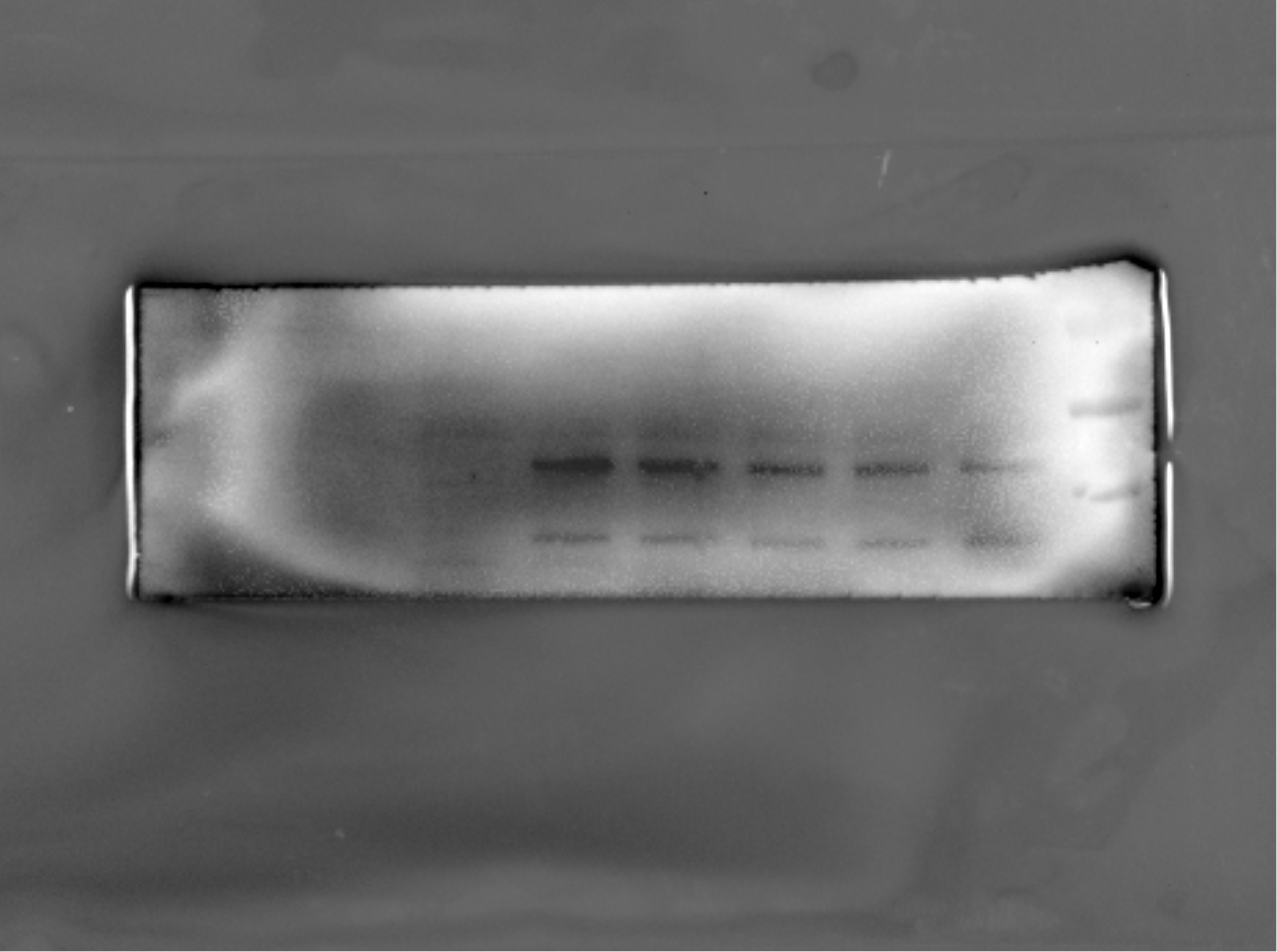

Supplement: Supplementary file 1 [file vetsci-12-00257-s001.zip › PABPC4 original blot images/Fig.2/B/SADS-CoV-n/merge.tif]

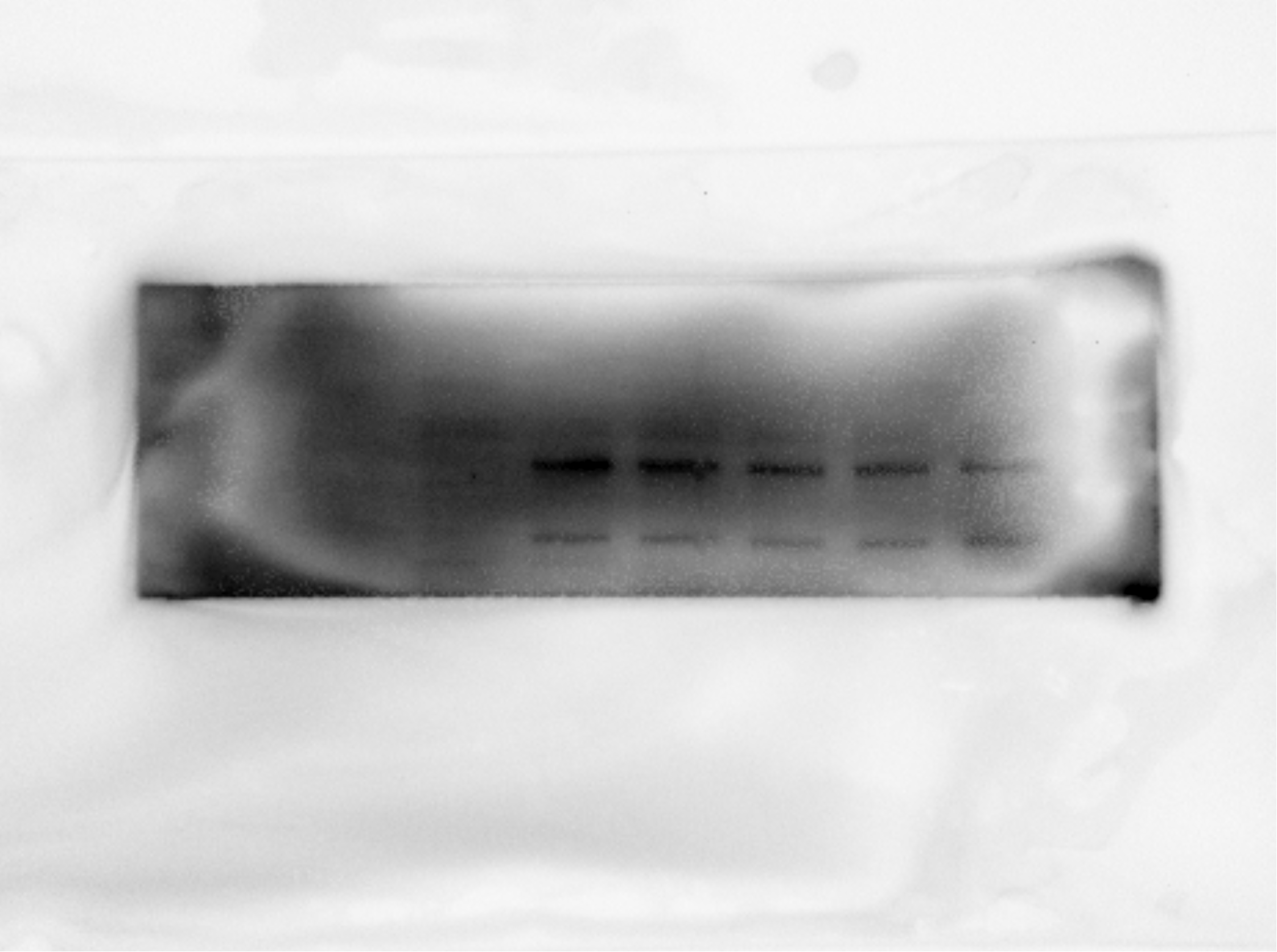

Supplement: Supplementary file 1 [file vetsci-12-00257-s001.zip › PABPC4 original blot images/Fig.2/B/SADS-CoV-n/s.tif]

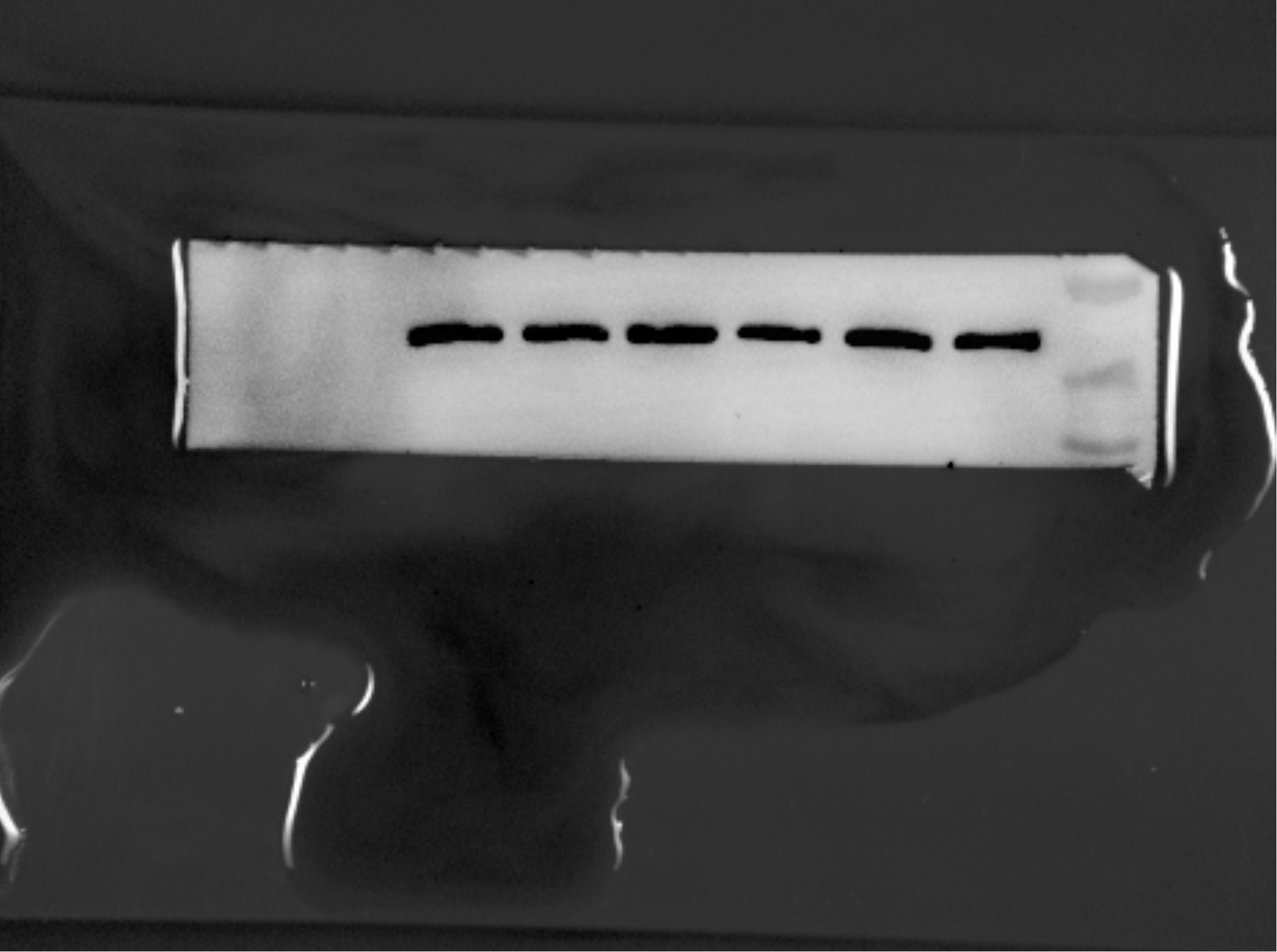

Supplement: Supplementary file 1 [file vetsci-12-00257-s001.zip › PABPC4 original blot images/Fig.2/E+F/GAPDH/H.tif]

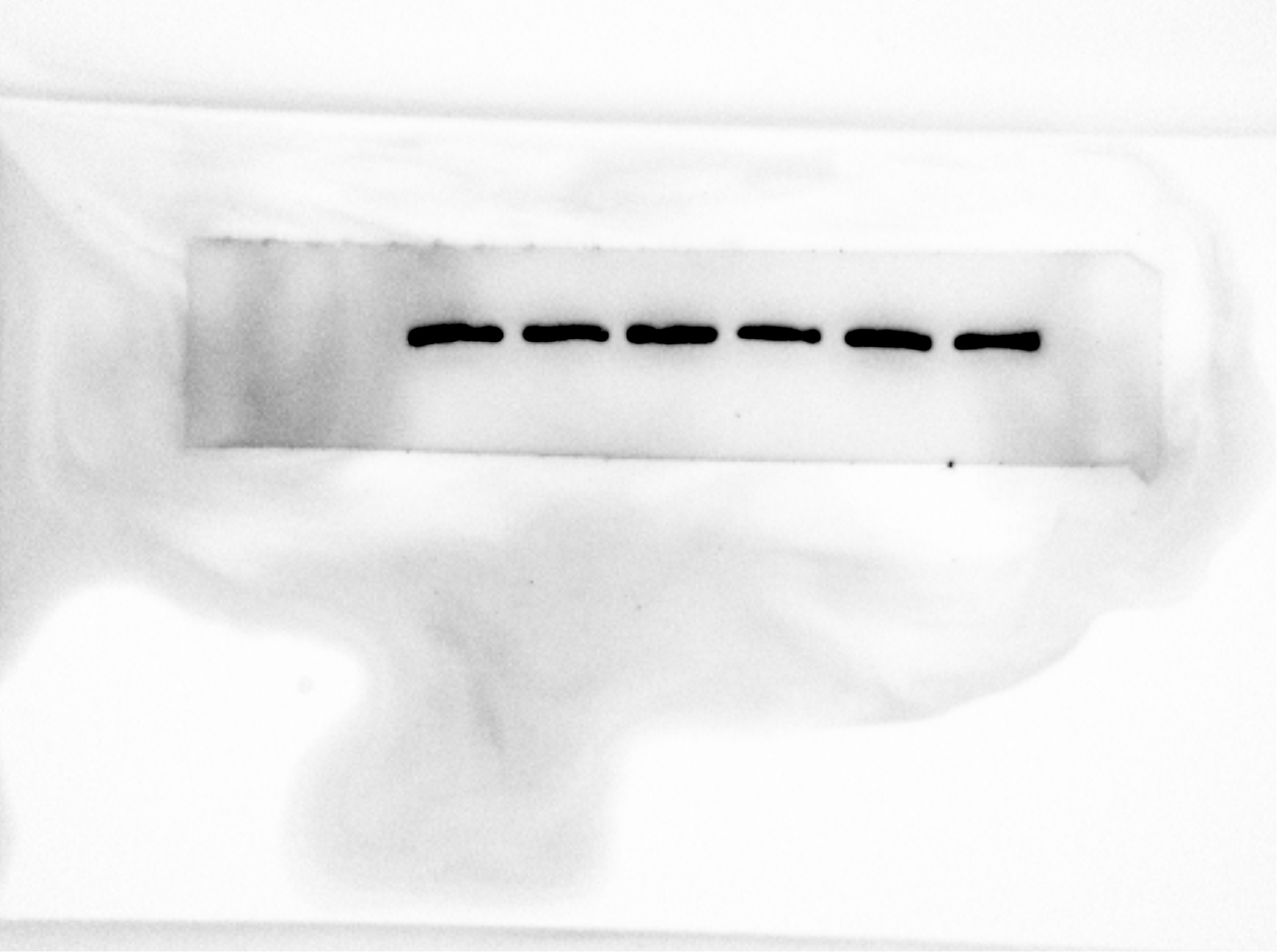

Supplement: Supplementary file 1 [file vetsci-12-00257-s001.zip › PABPC4 original blot images/Fig.2/E+F/GAPDH/S.tif]

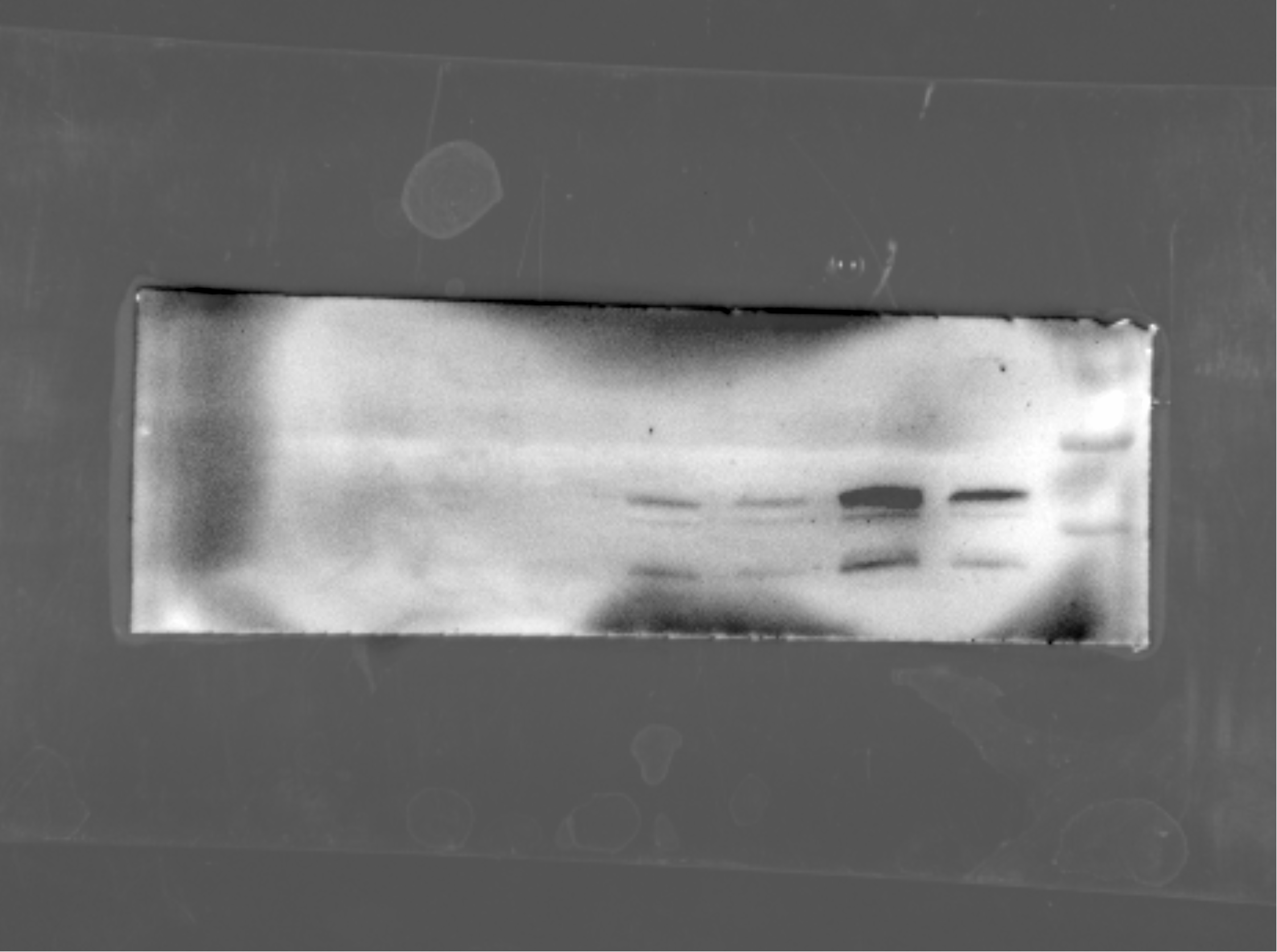

Supplement: Supplementary file 1 [file vetsci-12-00257-s001.zip › PABPC4 original blot images/Fig.2/E+F/N/h.tif]

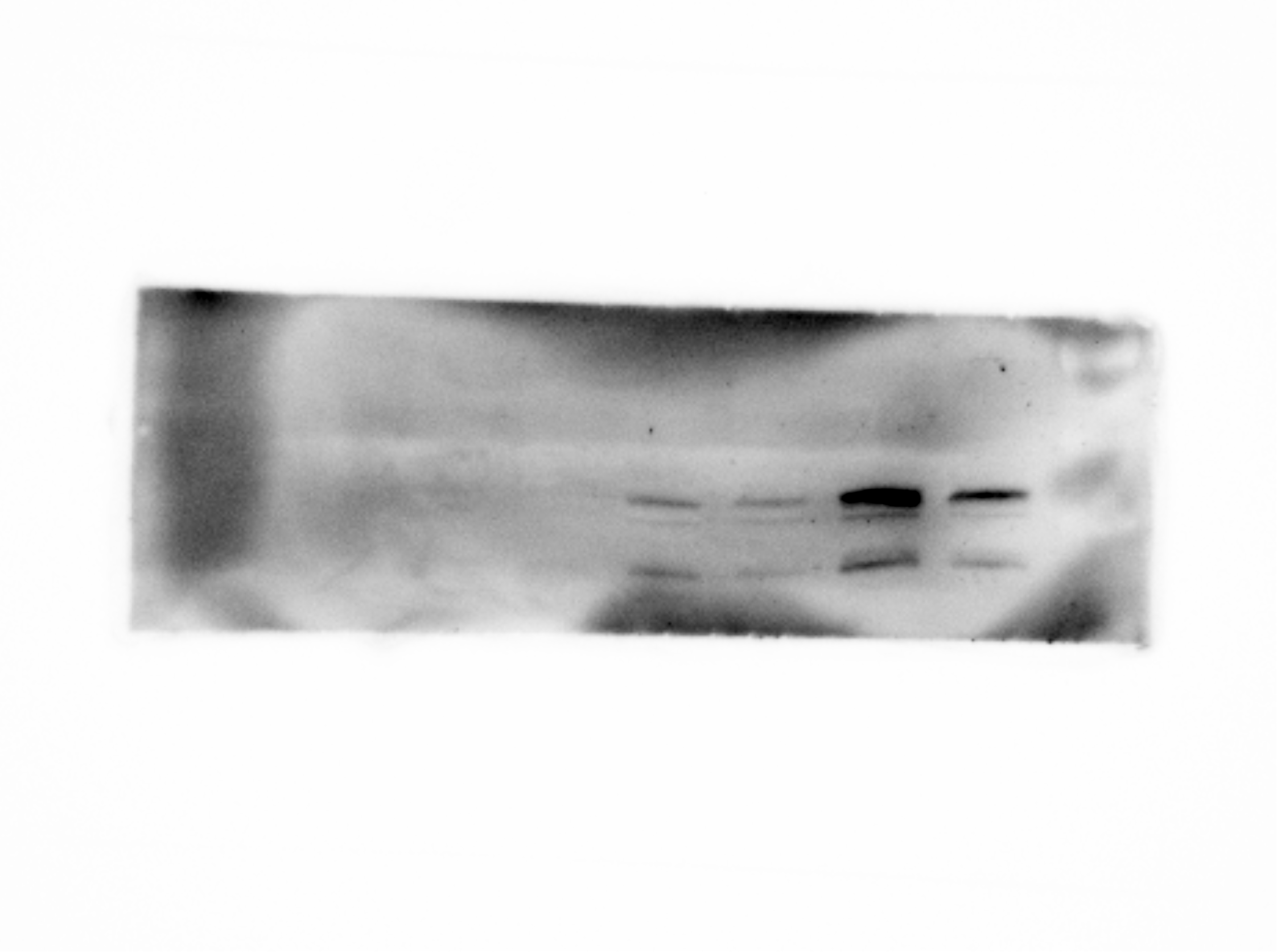

Supplement: Supplementary file 1 [file vetsci-12-00257-s001.zip › PABPC4 original blot images/Fig.2/E+F/N/m.tif]

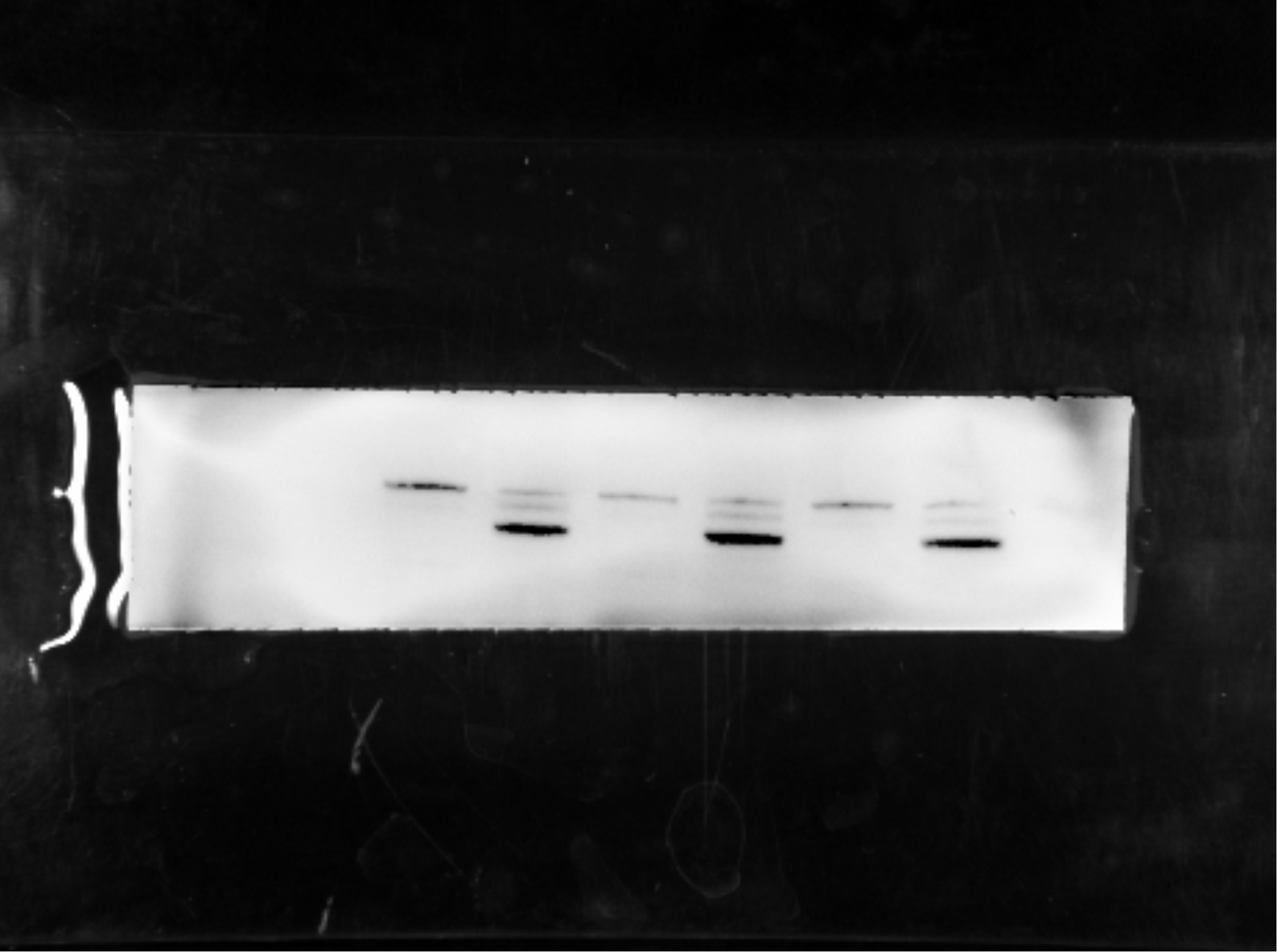

Supplement: Supplementary file 1 [file vetsci-12-00257-s001.zip › PABPC4 original blot images/Fig.2/E+F/PABPC4/h.tif]

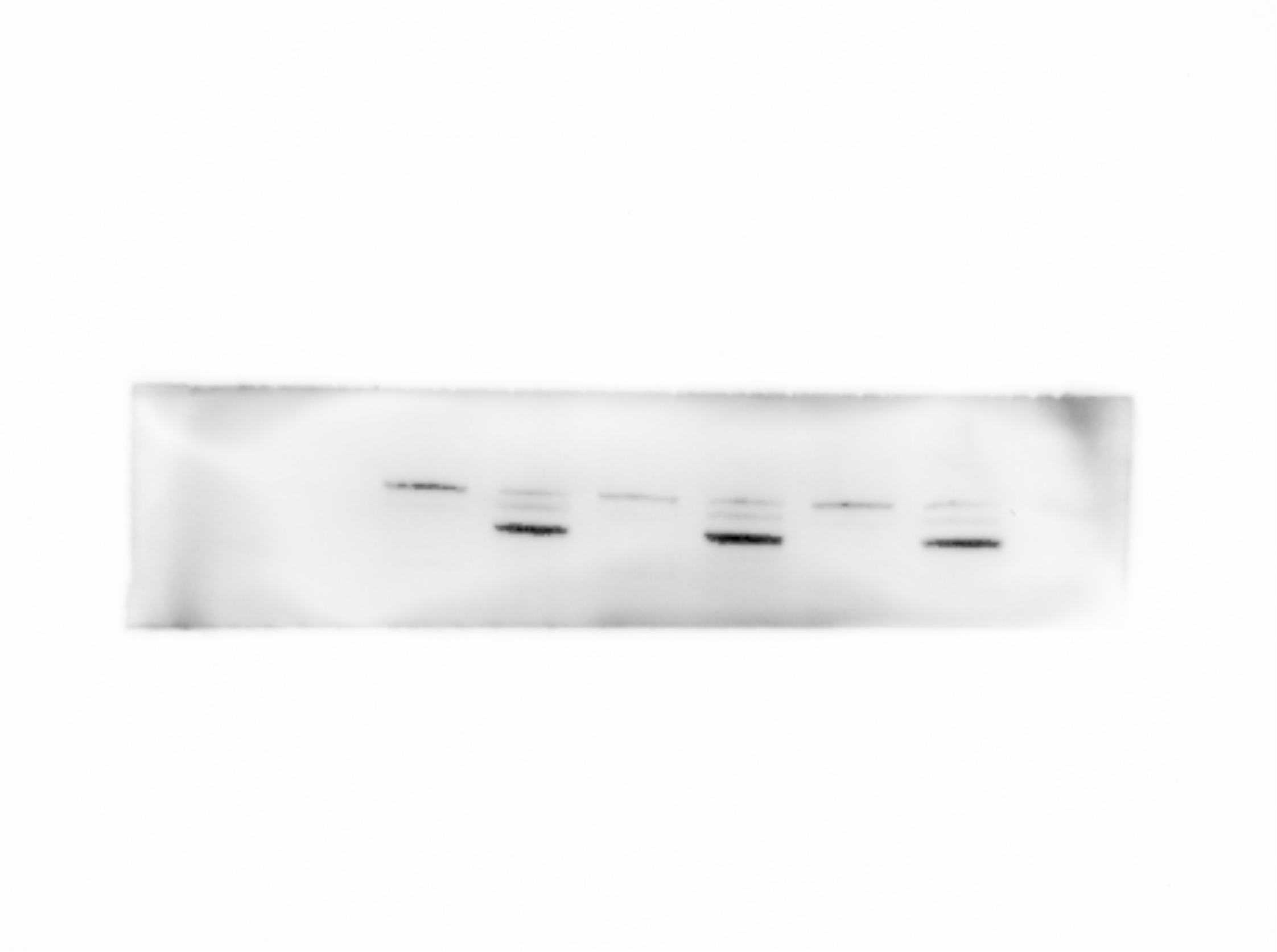

Supplement: Supplementary file 1 [file vetsci-12-00257-s001.zip › PABPC4 original blot images/Fig.2/E+F/PABPC4/s.tif]

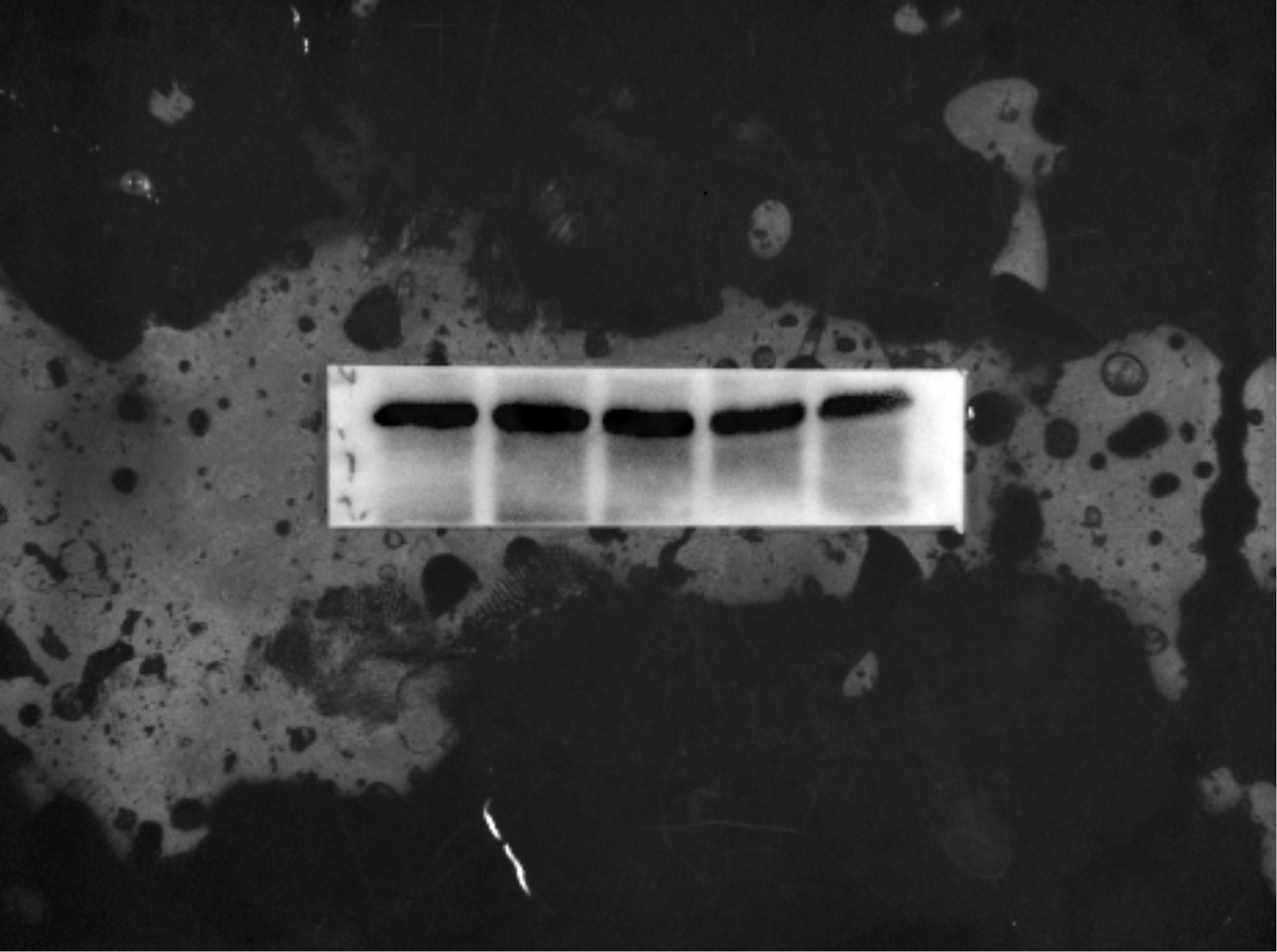

Supplement: Supplementary file 1 [file vetsci-12-00257-s001.zip › PABPC4 original blot images/Fig.2/G/gapdh/Administrator 2023-06-01_20h55m39s+Administrator 2023-06-01_20h54m42s.tif]

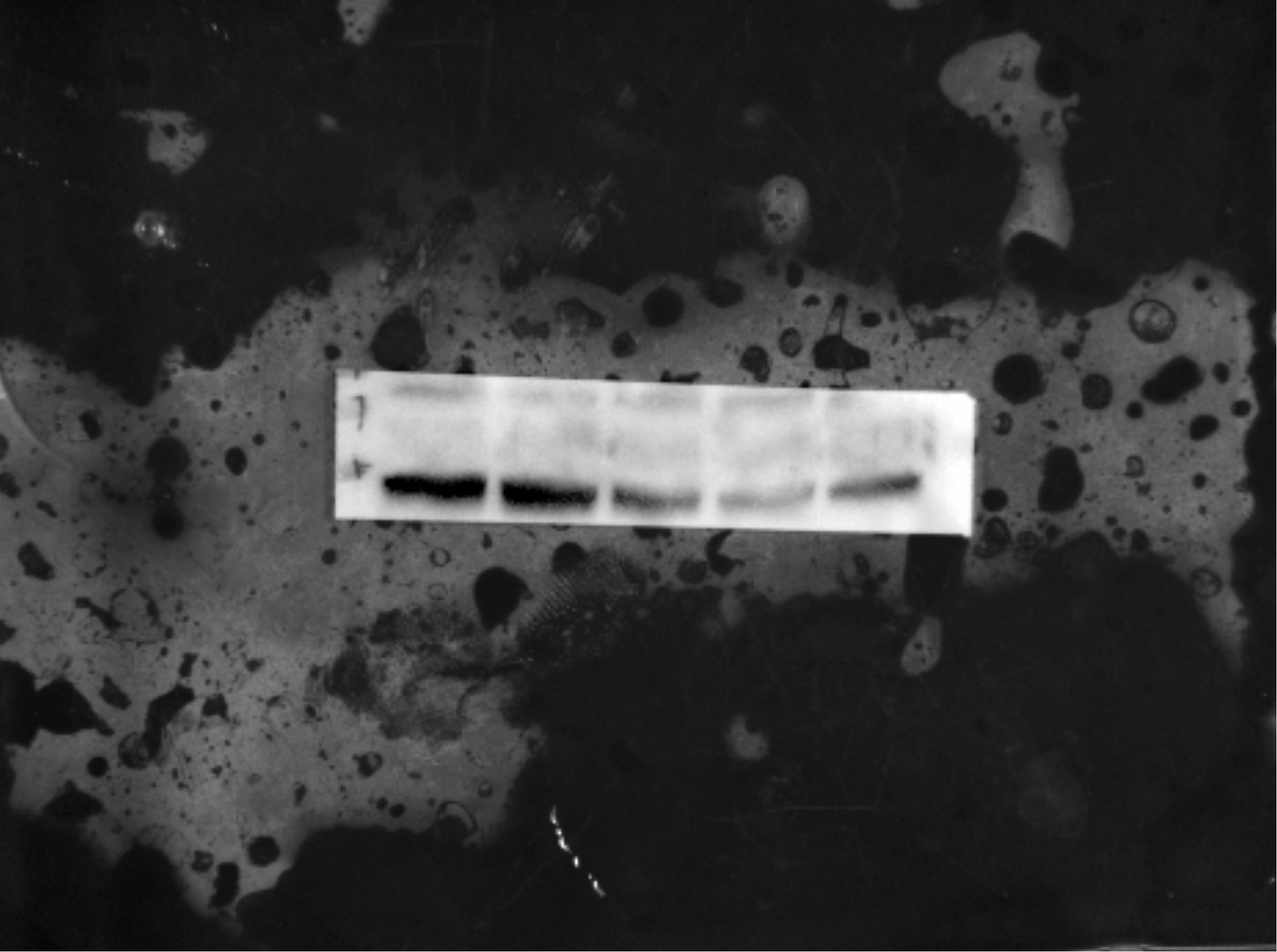

Supplement: Supplementary file 1 [file vetsci-12-00257-s001.zip › PABPC4 original blot images/Fig.2/G/PABPC4/Administrator 2023-06-01_20h53m14s+Administrator 2023-06-01_20h52m31s.tif]

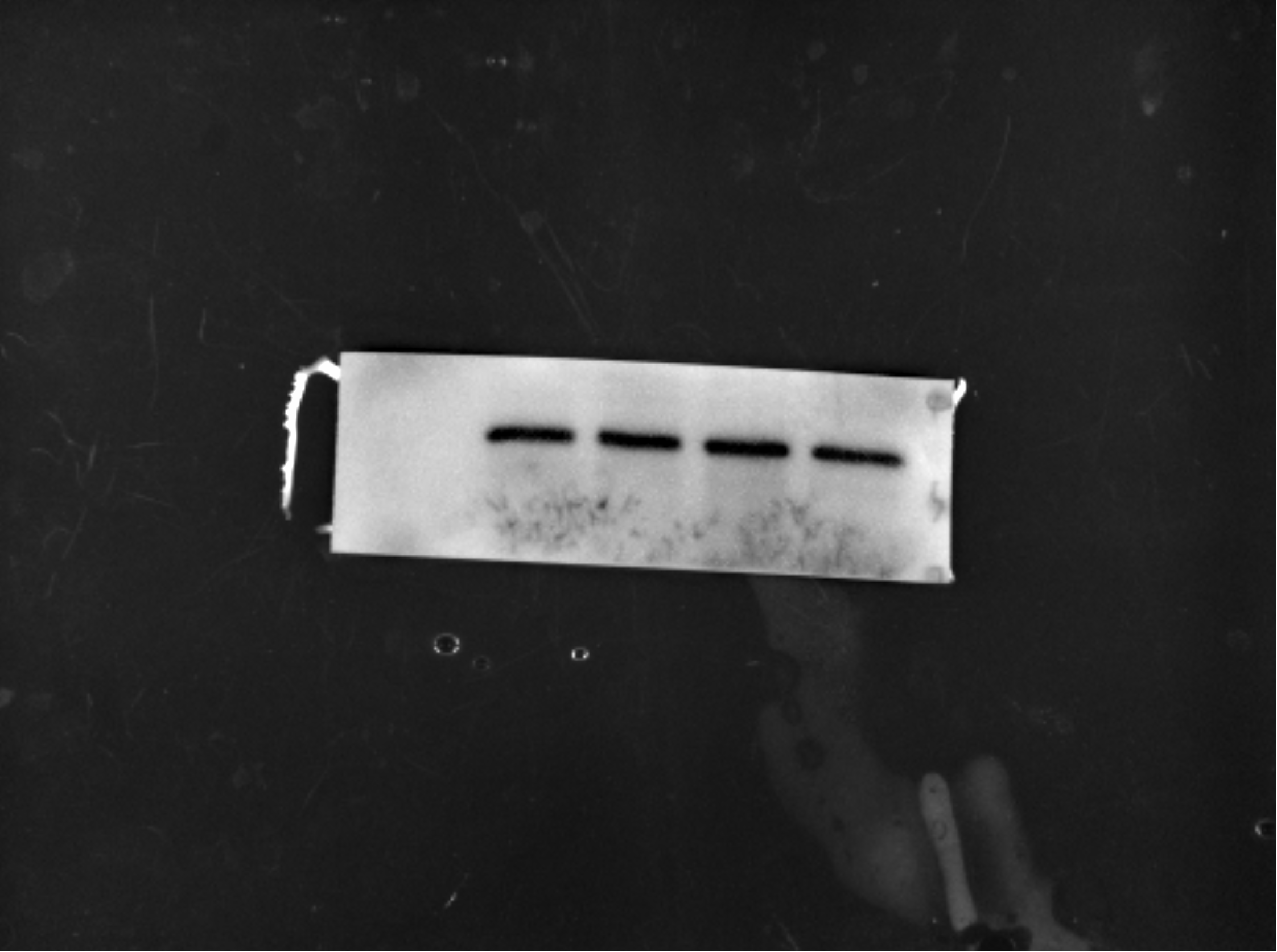

Supplement: Supplementary file 1 [file vetsci-12-00257-s001.zip › PABPC4 original blot images/Fig.2/H+I/gapdh/h.tif]

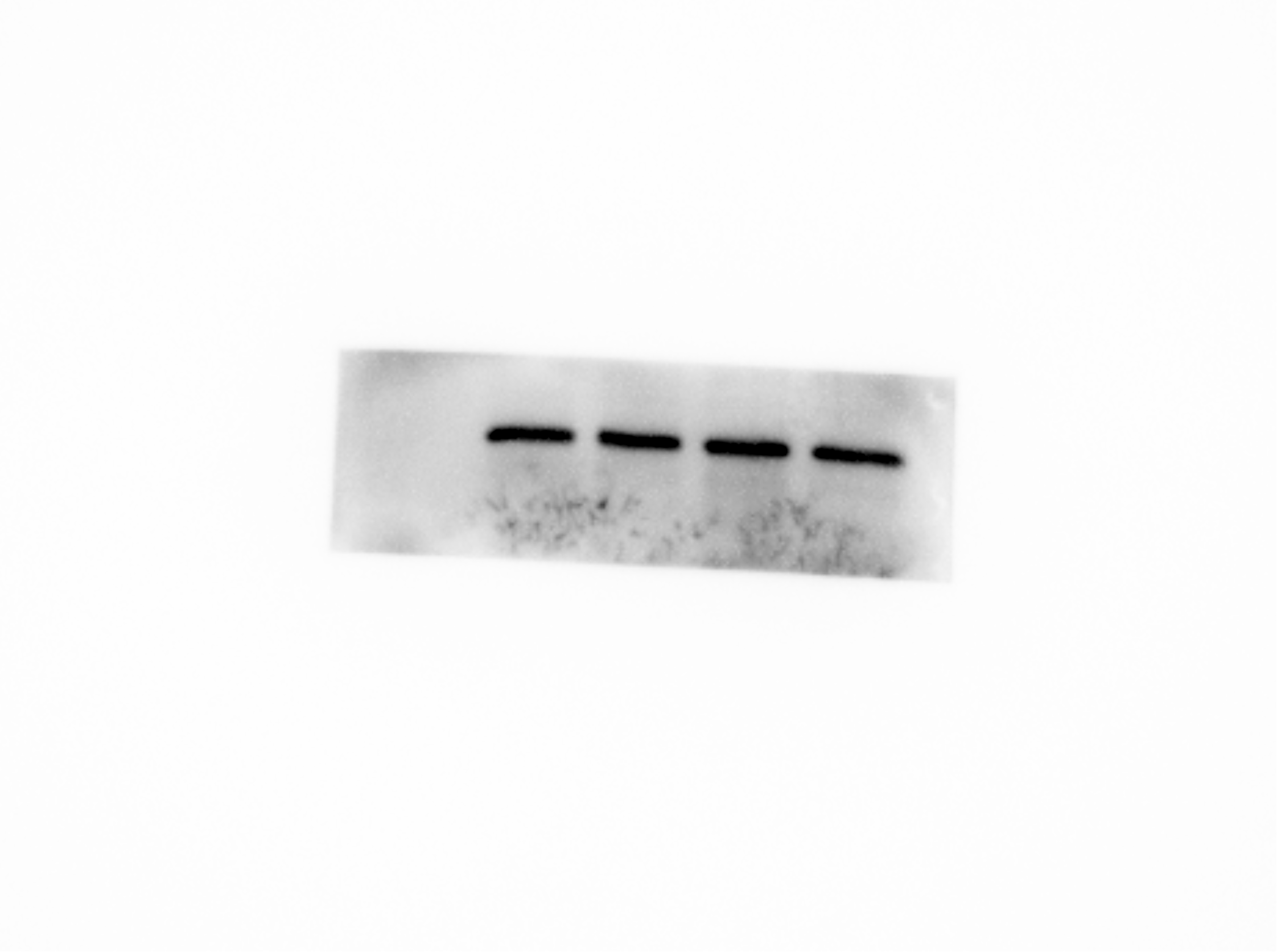

Supplement: Supplementary file 1 [file vetsci-12-00257-s001.zip › PABPC4 original blot images/Fig.2/H+I/gapdh/s.tif]

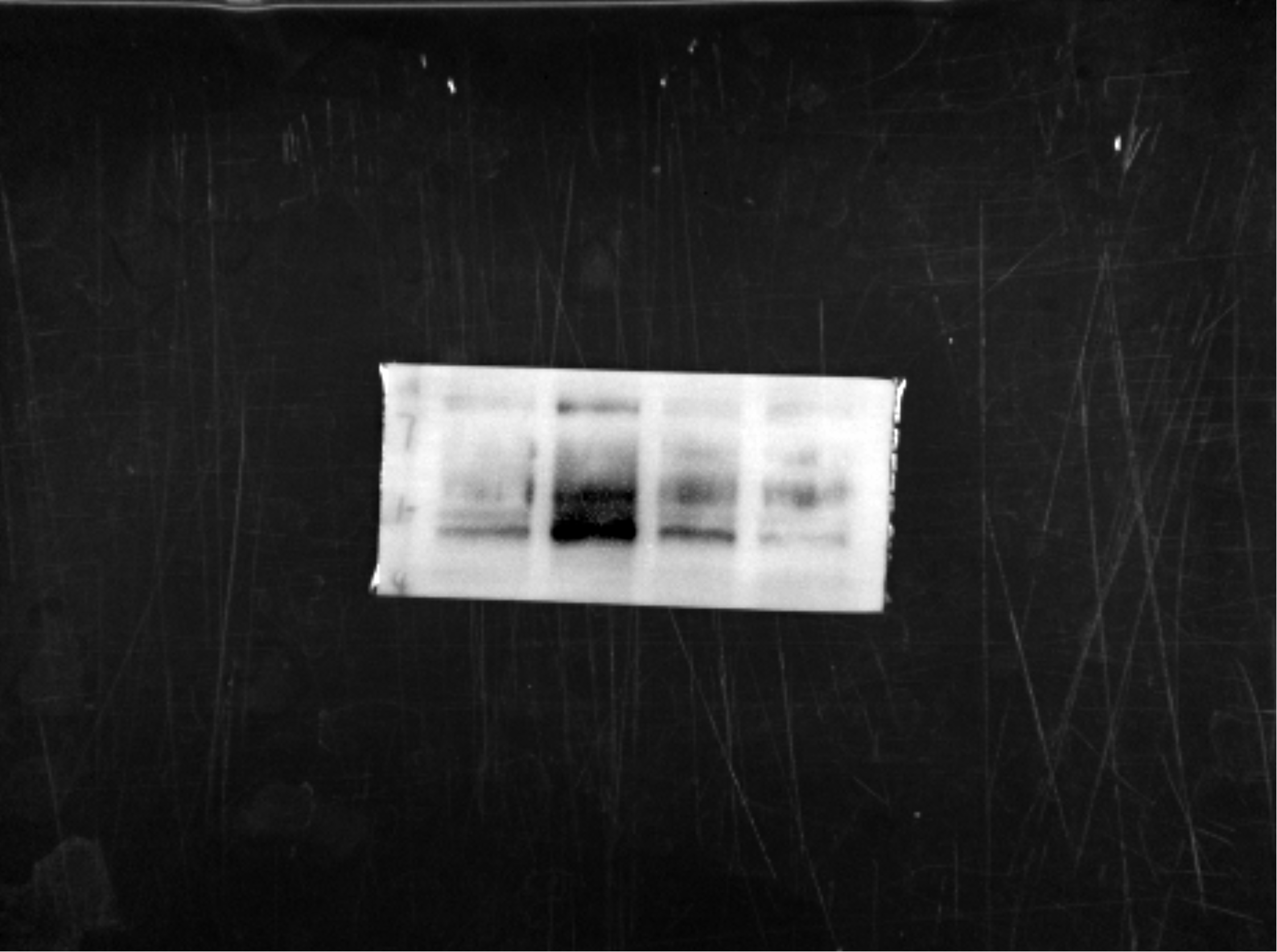

Supplement: Supplementary file 1 [file vetsci-12-00257-s001.zip › PABPC4 original blot images/Fig.2/H+I/p4/h.tif]

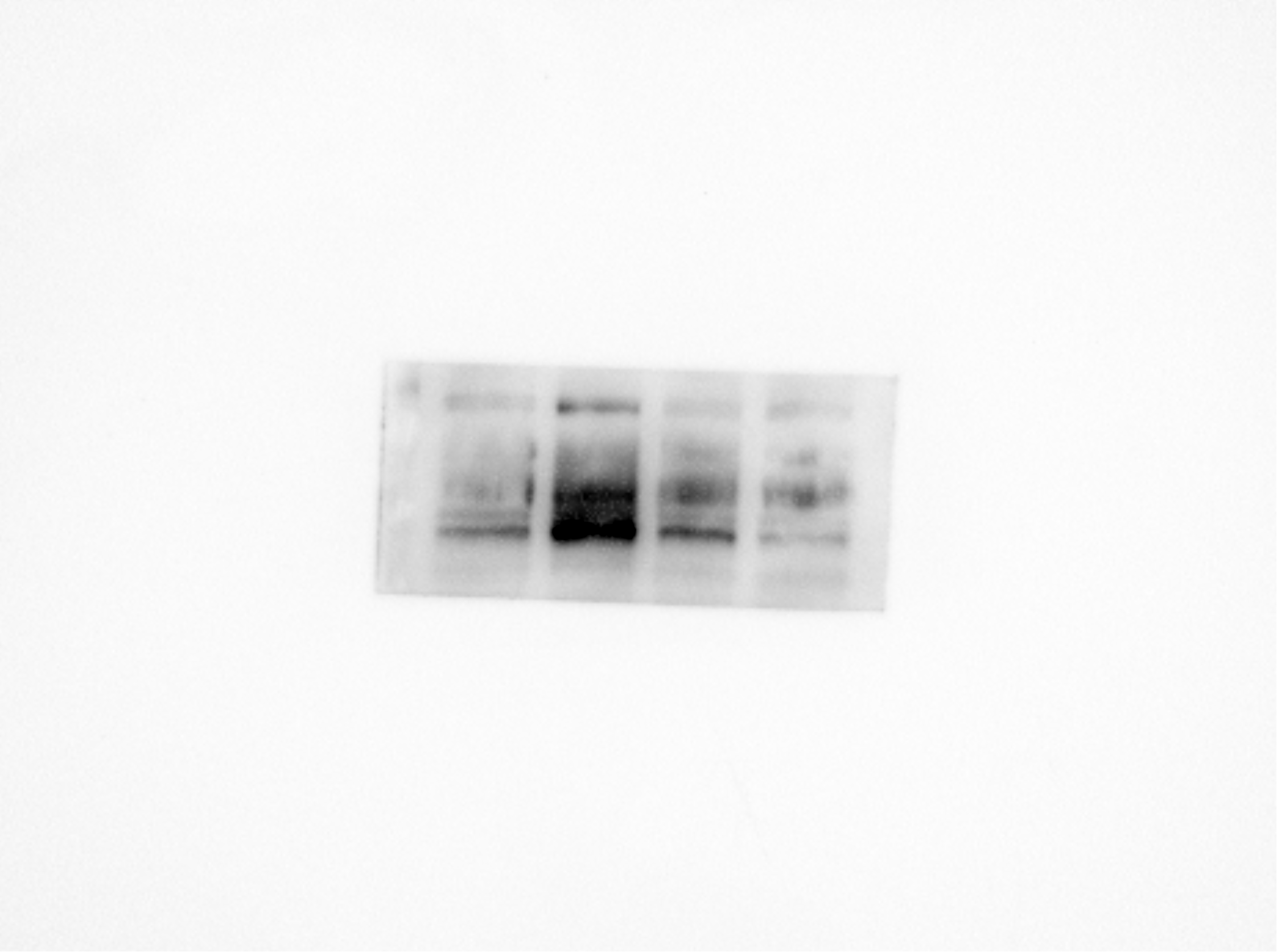

Supplement: Supplementary file 1 [file vetsci-12-00257-s001.zip › PABPC4 original blot images/Fig.2/H+I/p4/m.tif]

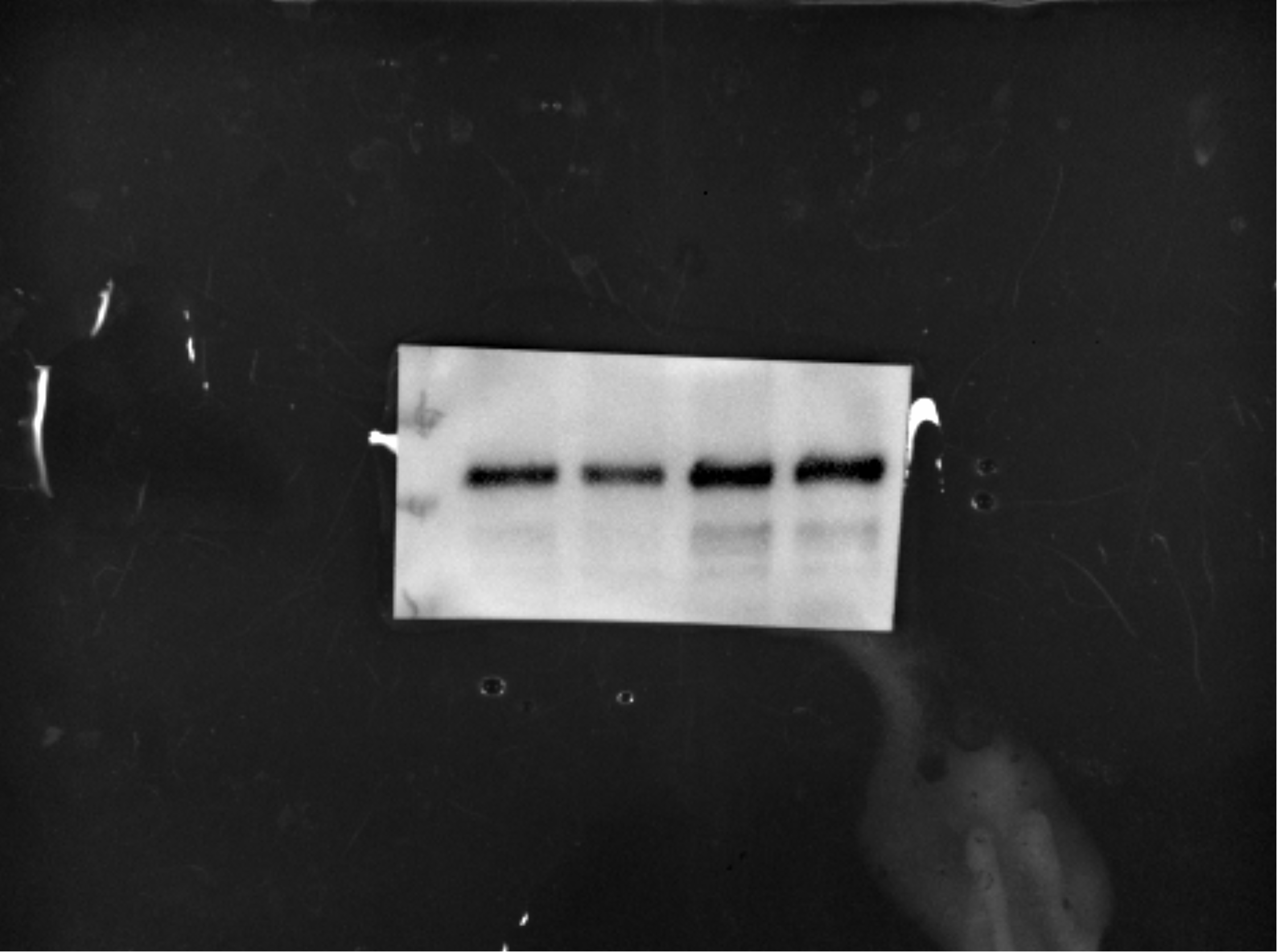

Supplement: Supplementary file 1 [file vetsci-12-00257-s001.zip › PABPC4 original blot images/Fig.2/H+I/SADS-CoV-n/h.tif]

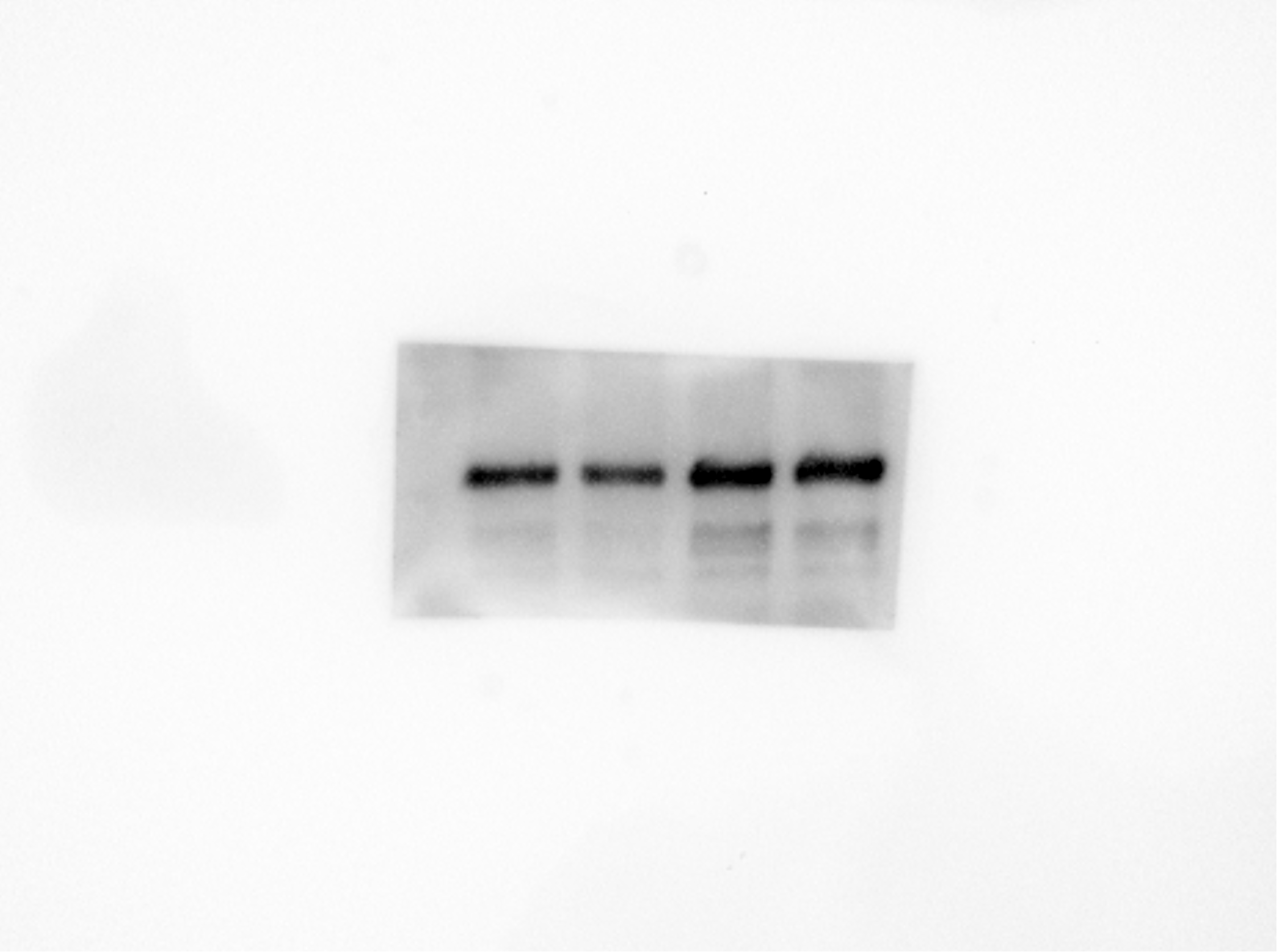

Supplement: Supplementary file 1 [file vetsci-12-00257-s001.zip › PABPC4 original blot images/Fig.2/H+I/SADS-CoV-n/s.tif]

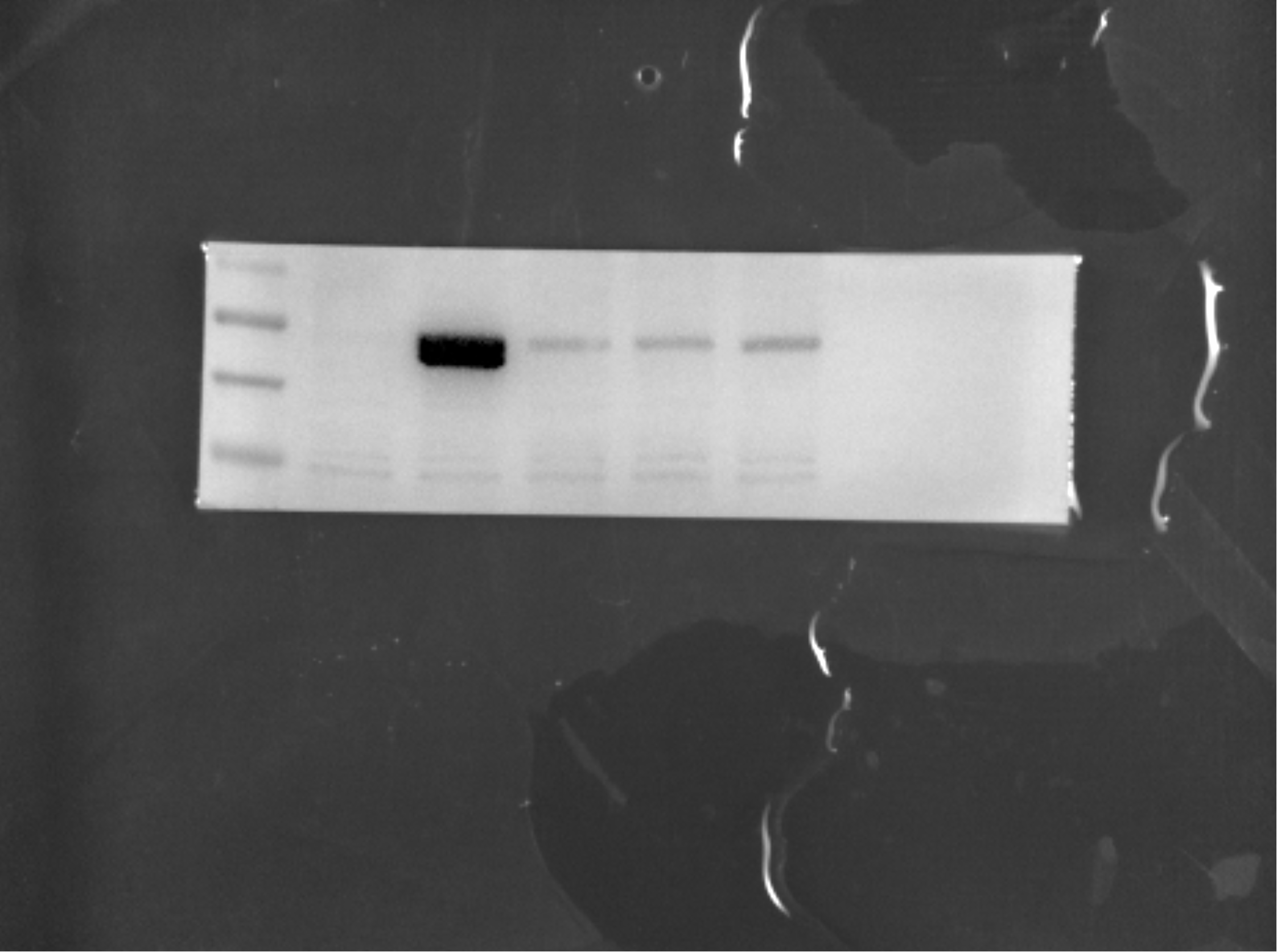

Supplement: Supplementary file 1 [file vetsci-12-00257-s001.zip › PABPC4 original blot images/Fig.3/A/flag/merge.tif]

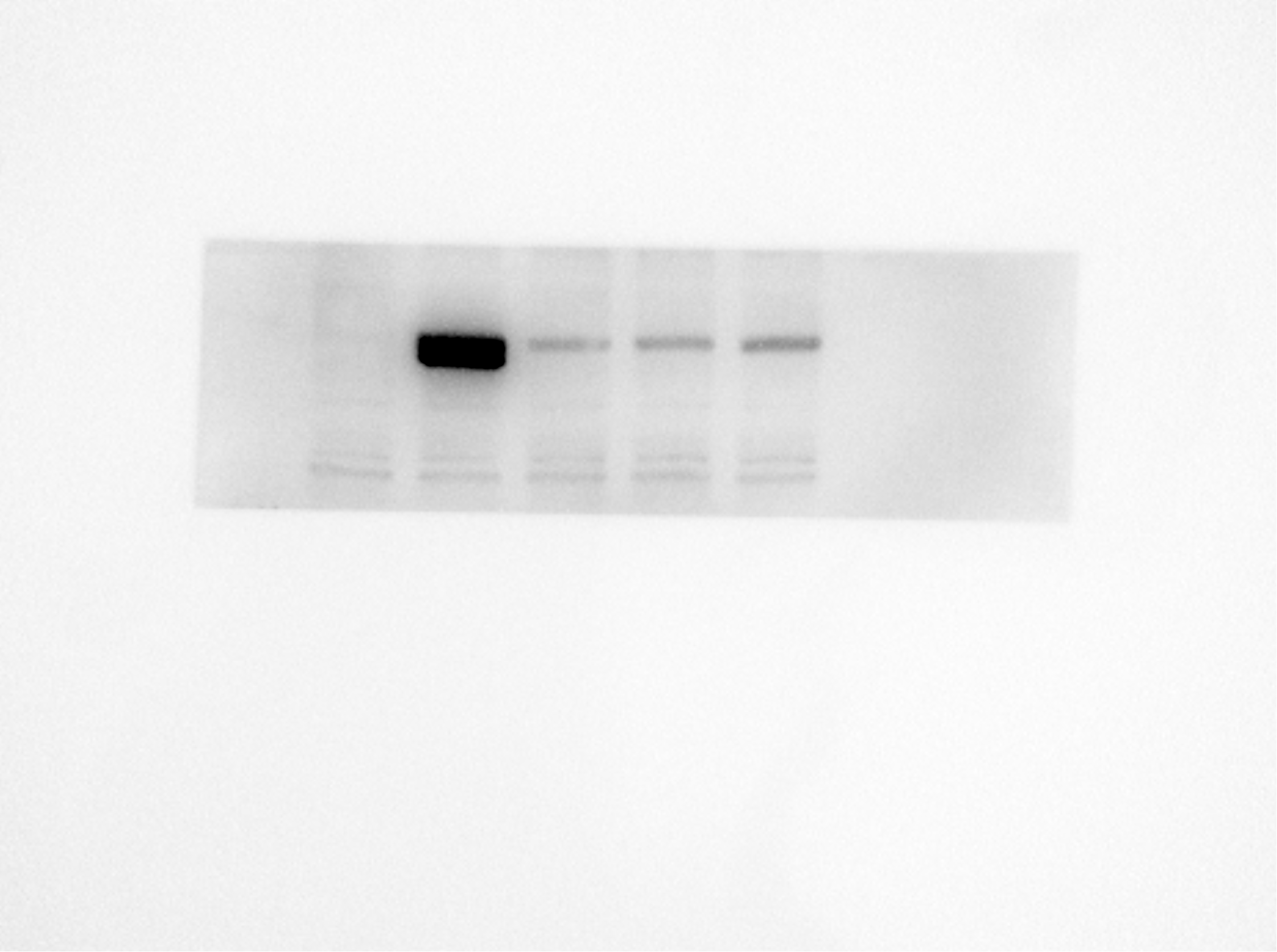

Supplement: Supplementary file 1 [file vetsci-12-00257-s001.zip › PABPC4 original blot images/Fig.3/A/flag/shiyantu.tif]

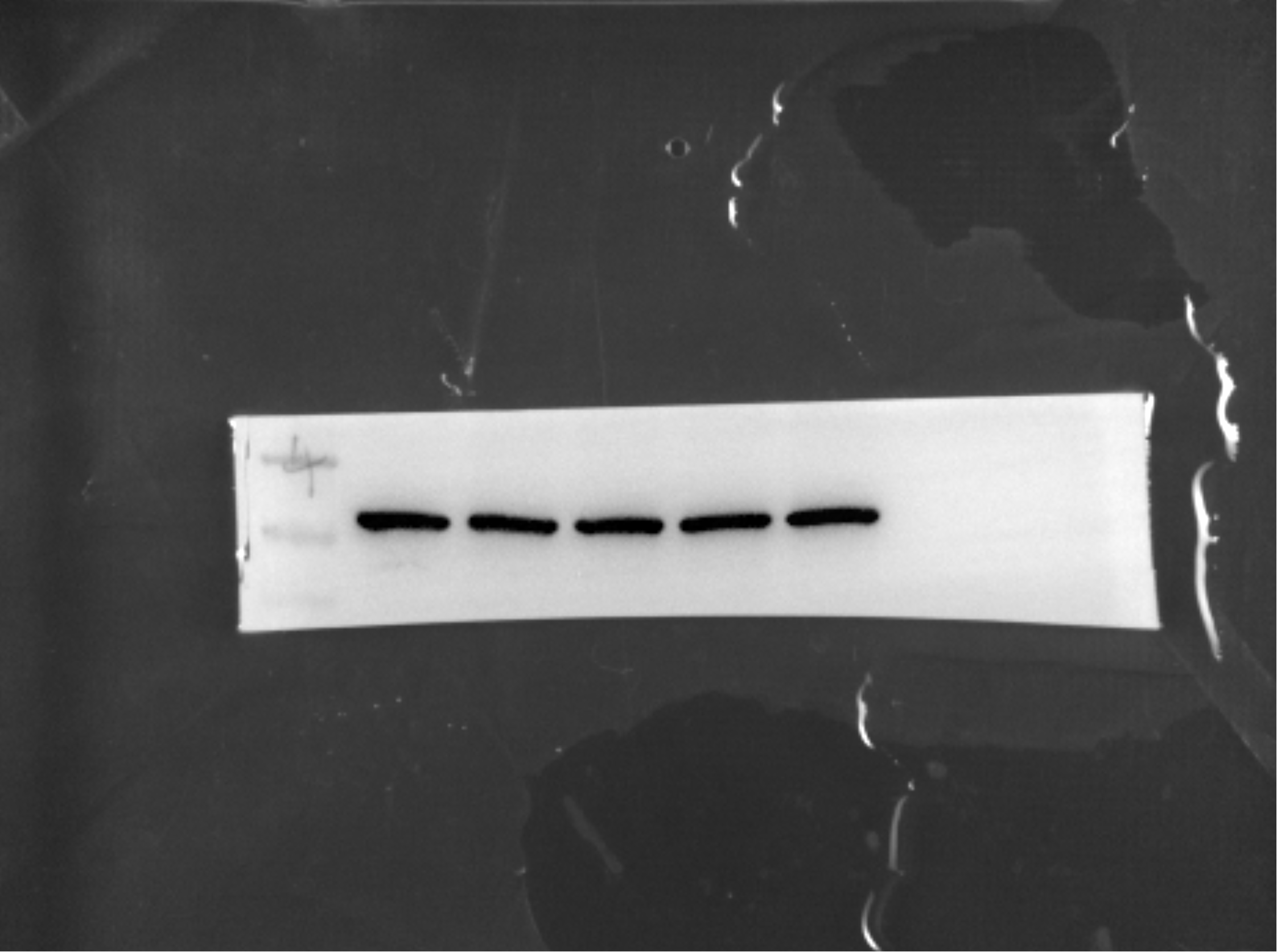

Supplement: Supplementary file 1 [file vetsci-12-00257-s001.zip › PABPC4 original blot images/Fig.3/A/gapdh/merge.tif]

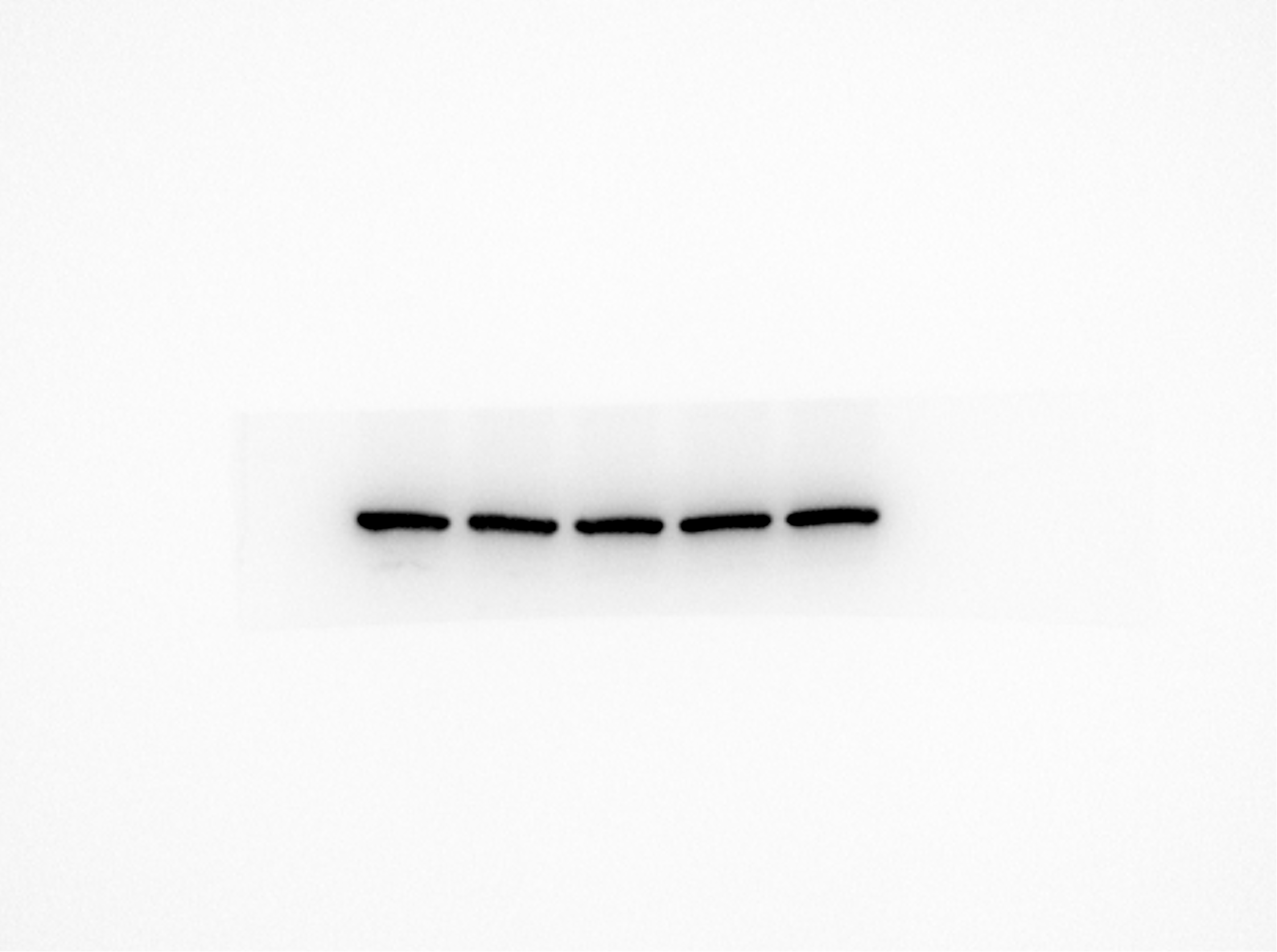

Supplement: Supplementary file 1 [file vetsci-12-00257-s001.zip › PABPC4 original blot images/Fig.3/A/gapdh/shiyantu.tif]

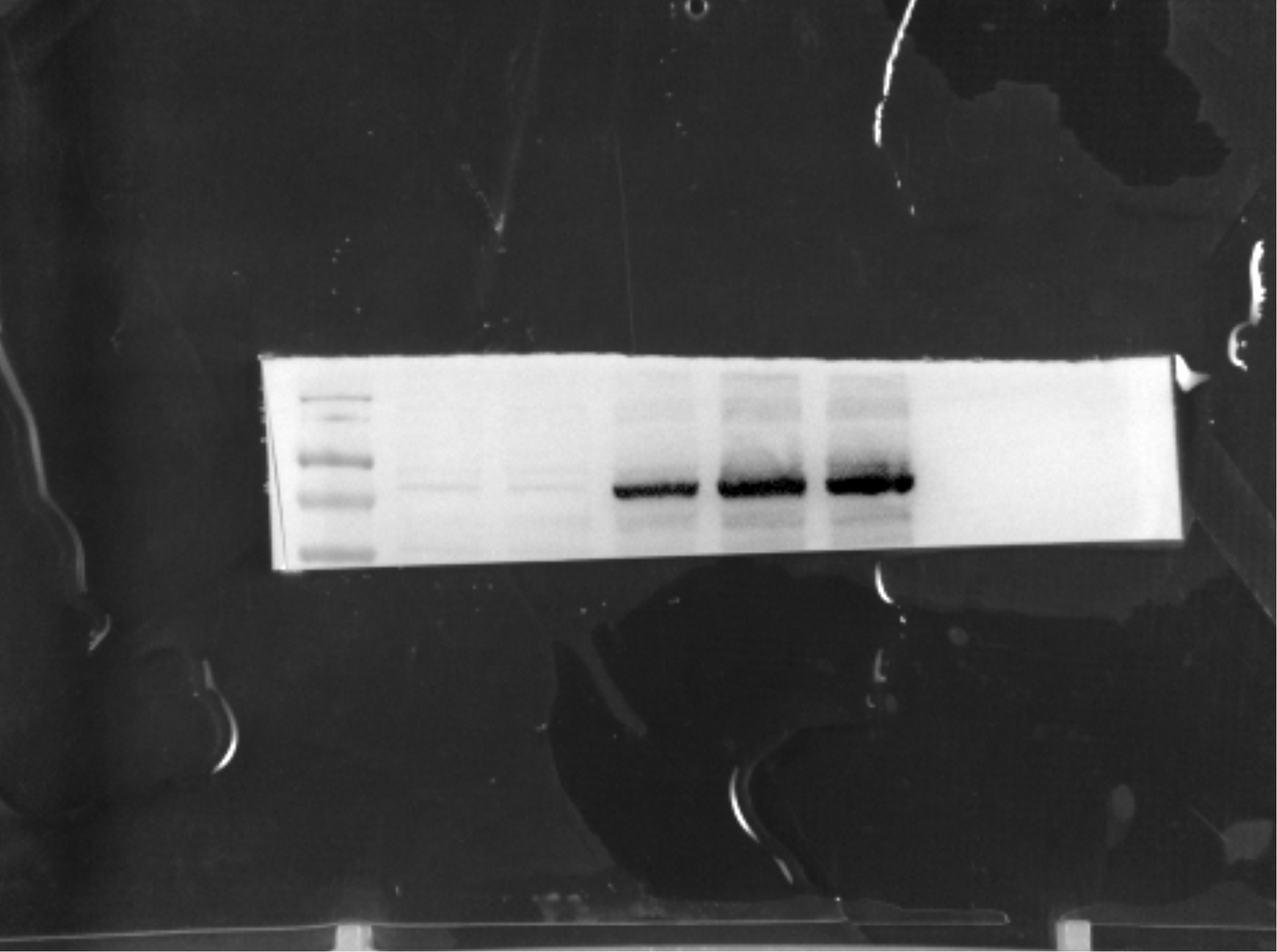

Supplement: Supplementary file 1 [file vetsci-12-00257-s001.zip › PABPC4 original blot images/Fig.3/A/ha/merge.tif]

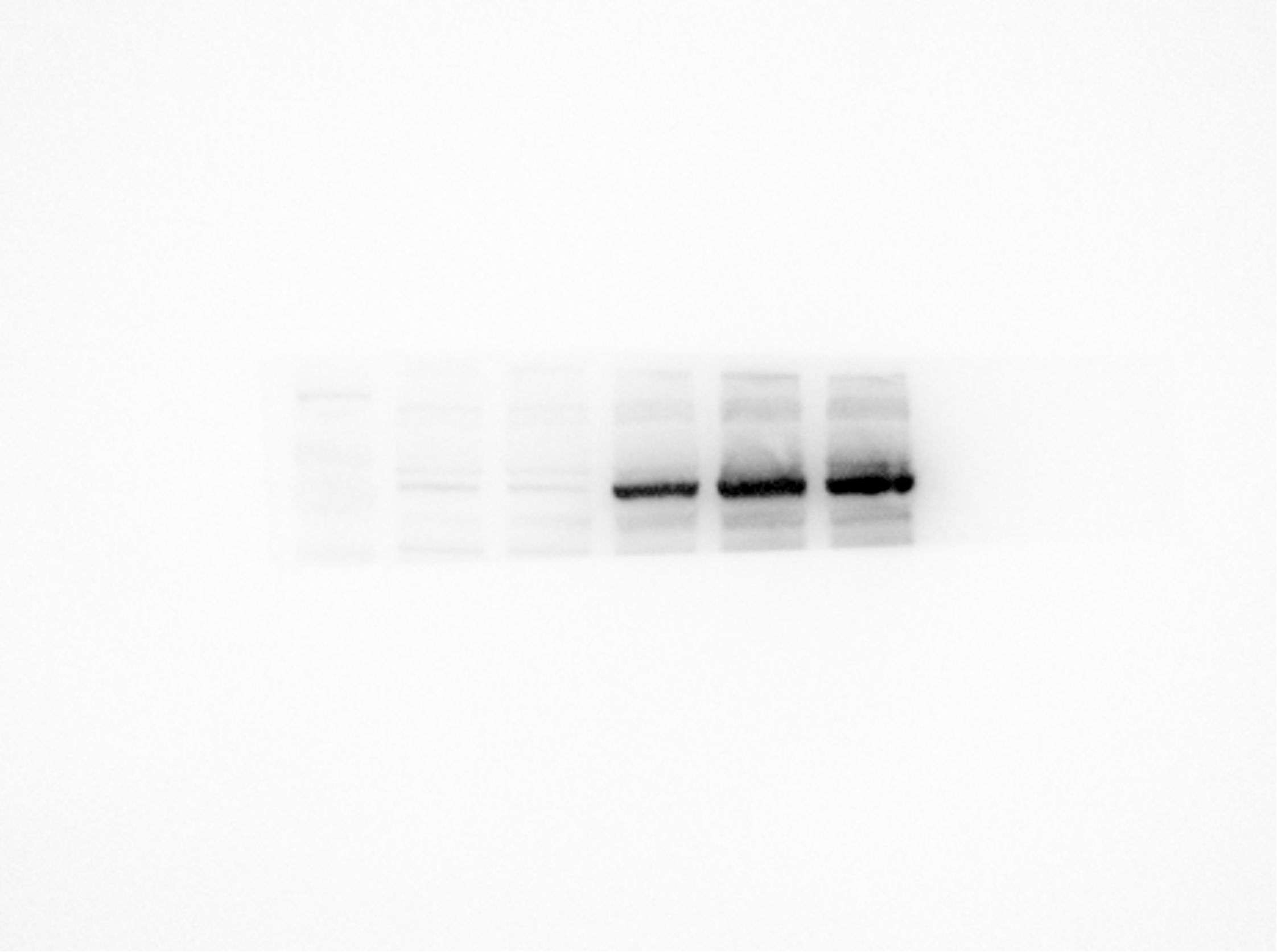

Supplement: Supplementary file 1 [file vetsci-12-00257-s001.zip › PABPC4 original blot images/Fig.3/A/ha/shiyantu.tif]

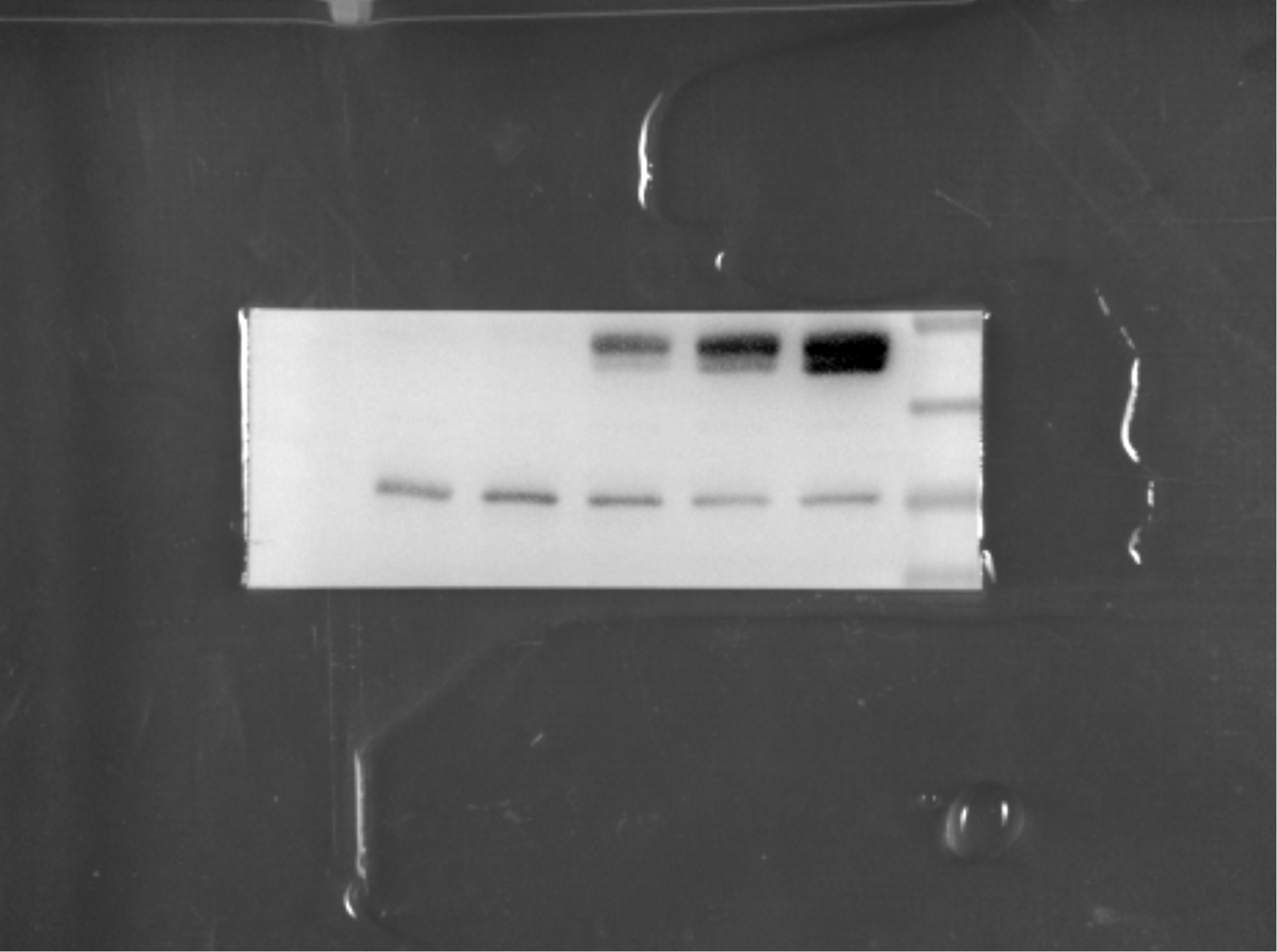

Supplement: Supplementary file 1 [file vetsci-12-00257-s001.zip › PABPC4 original blot images/Fig.3/B/flag/merge.tif]

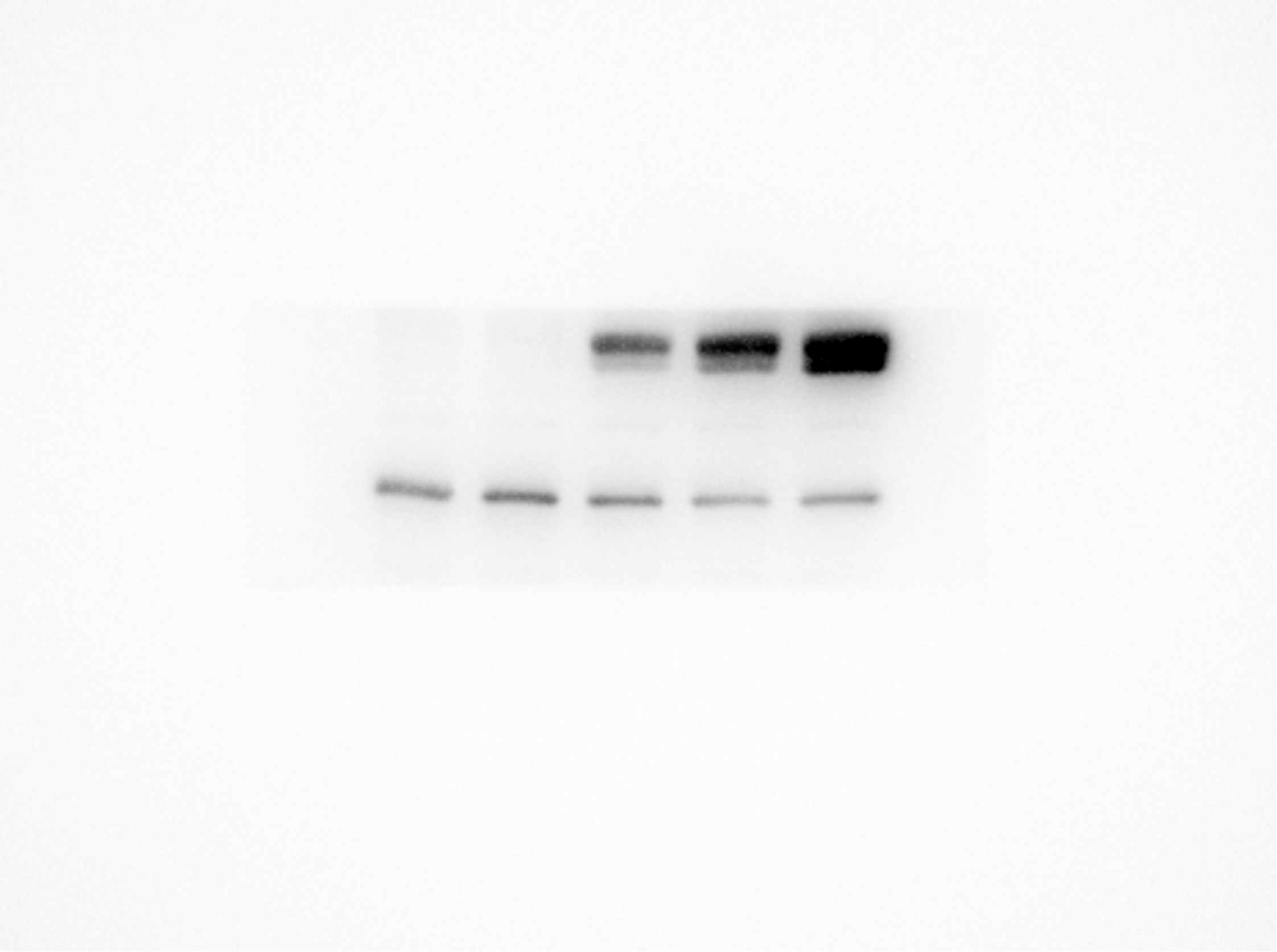

Supplement: Supplementary file 1 [file vetsci-12-00257-s001.zip › PABPC4 original blot images/Fig.3/B/flag/s.tif]

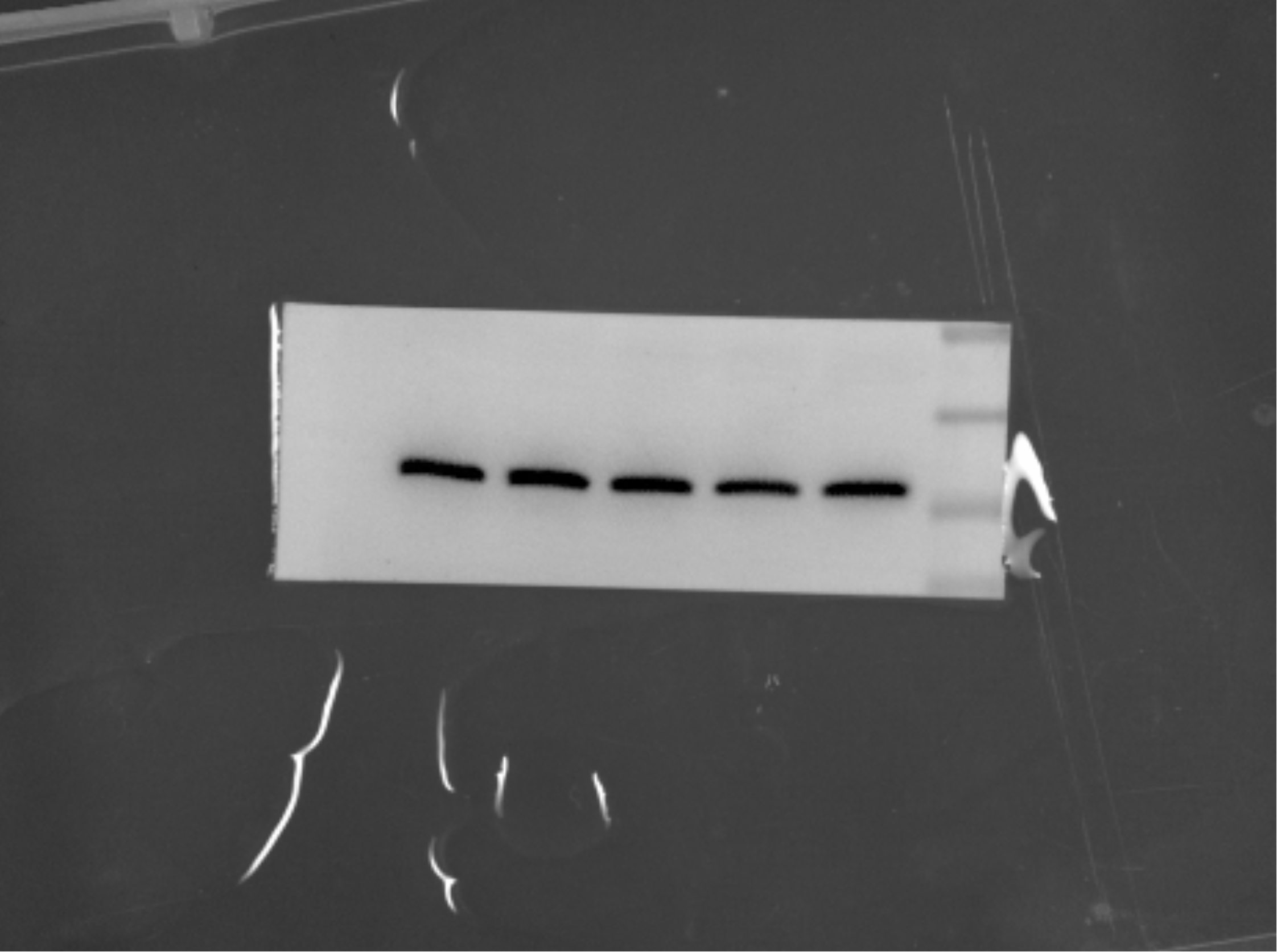

Supplement: Supplementary file 1 [file vetsci-12-00257-s001.zip › PABPC4 original blot images/Fig.3/B/GAPDH/merge.tif]

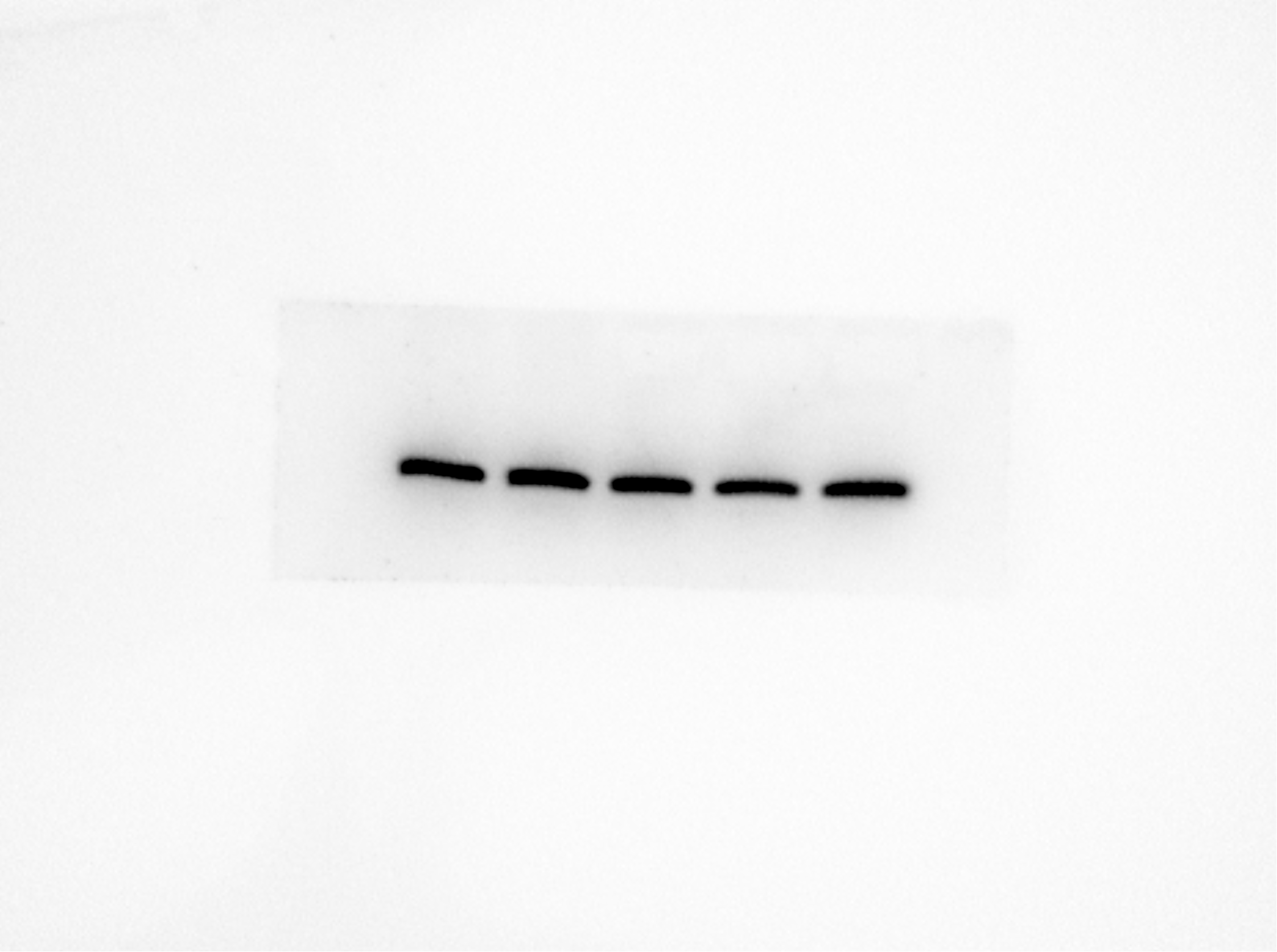

Supplement: Supplementary file 1 [file vetsci-12-00257-s001.zip › PABPC4 original blot images/Fig.3/B/GAPDH/shiyantu.tif]

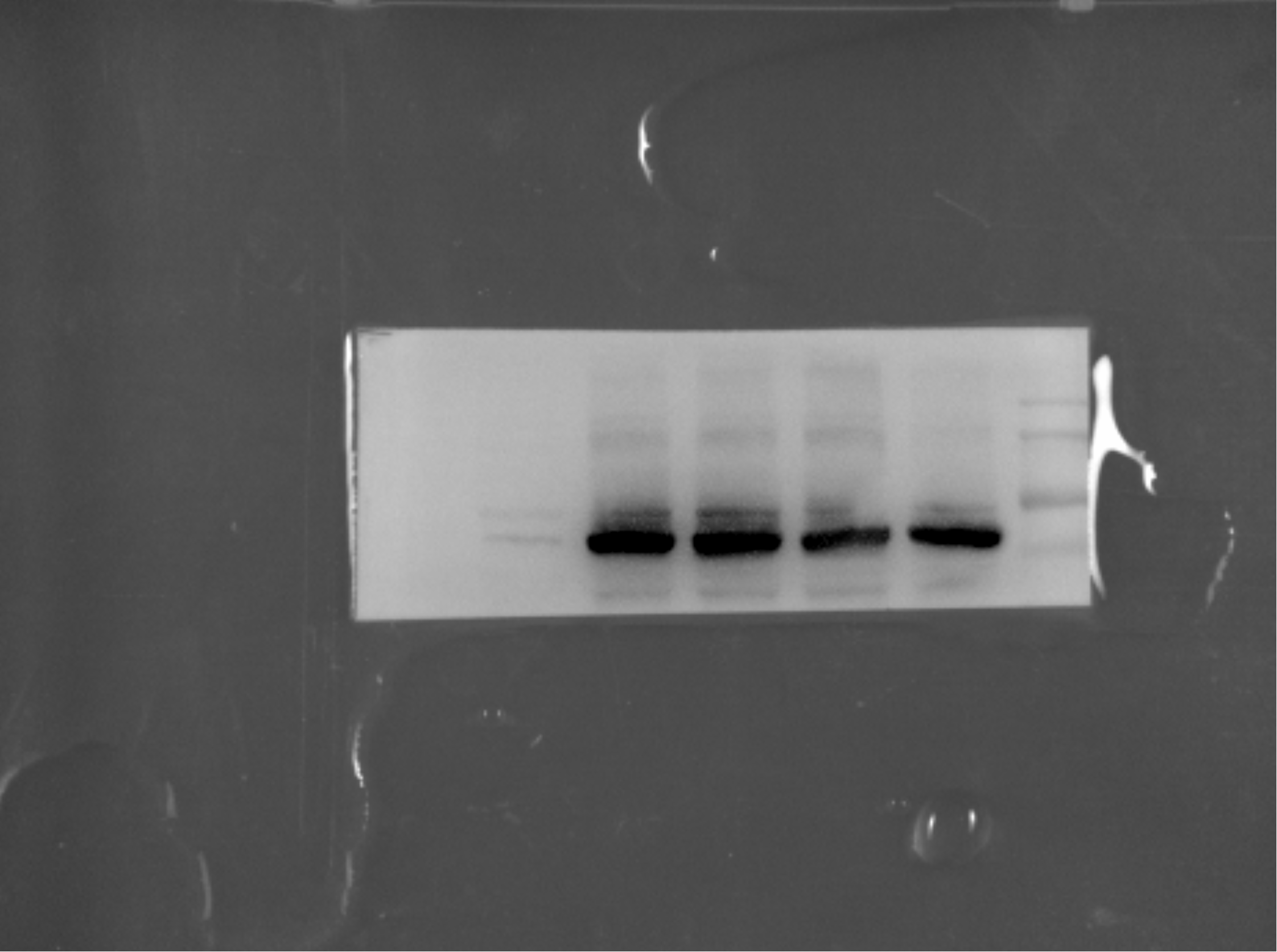

Supplement: Supplementary file 1 [file vetsci-12-00257-s001.zip › PABPC4 original blot images/Fig.3/B/ha/merge.tif]

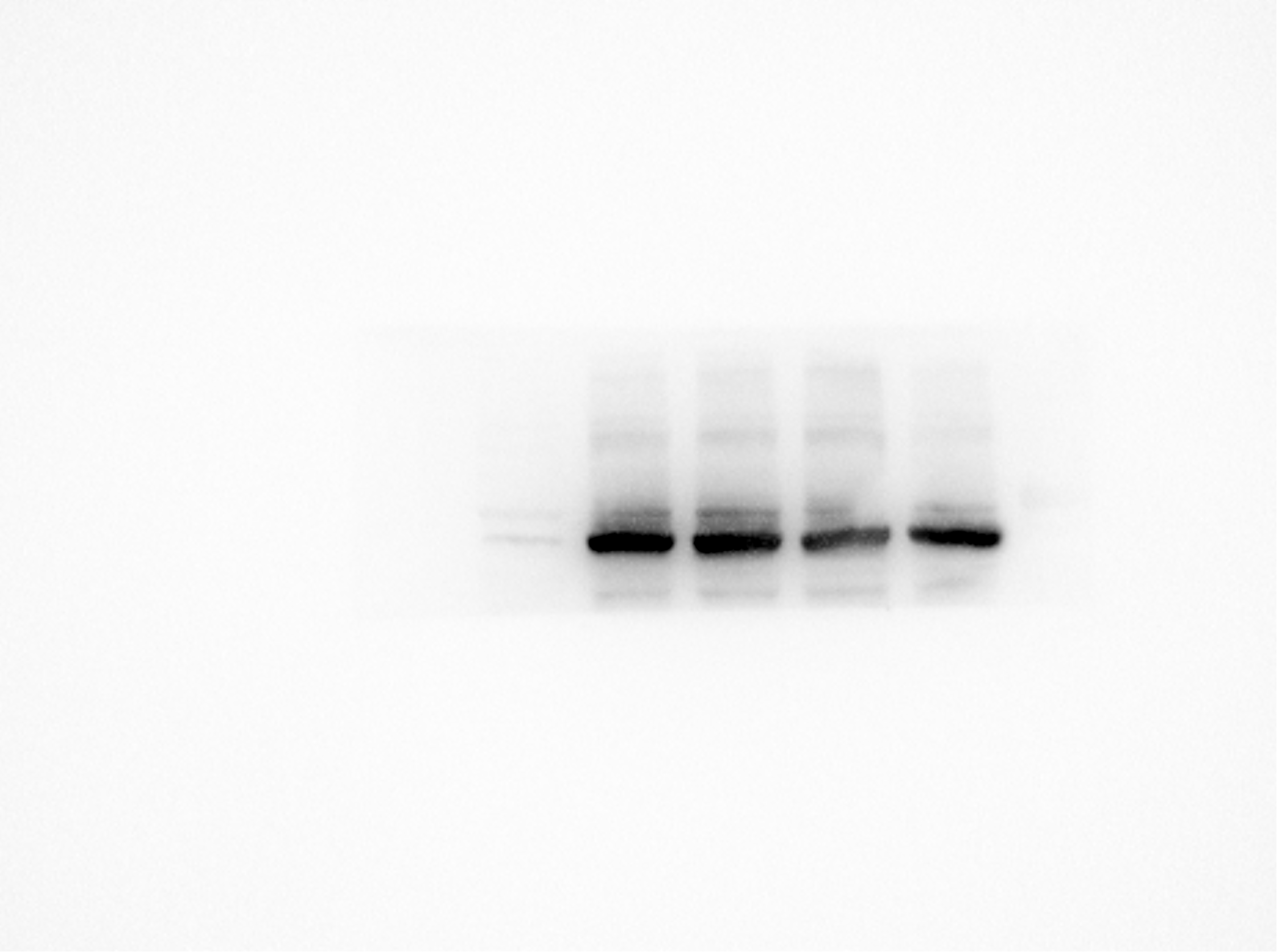

Supplement: Supplementary file 1 [file vetsci-12-00257-s001.zip › PABPC4 original blot images/Fig.3/B/ha/s.tif]

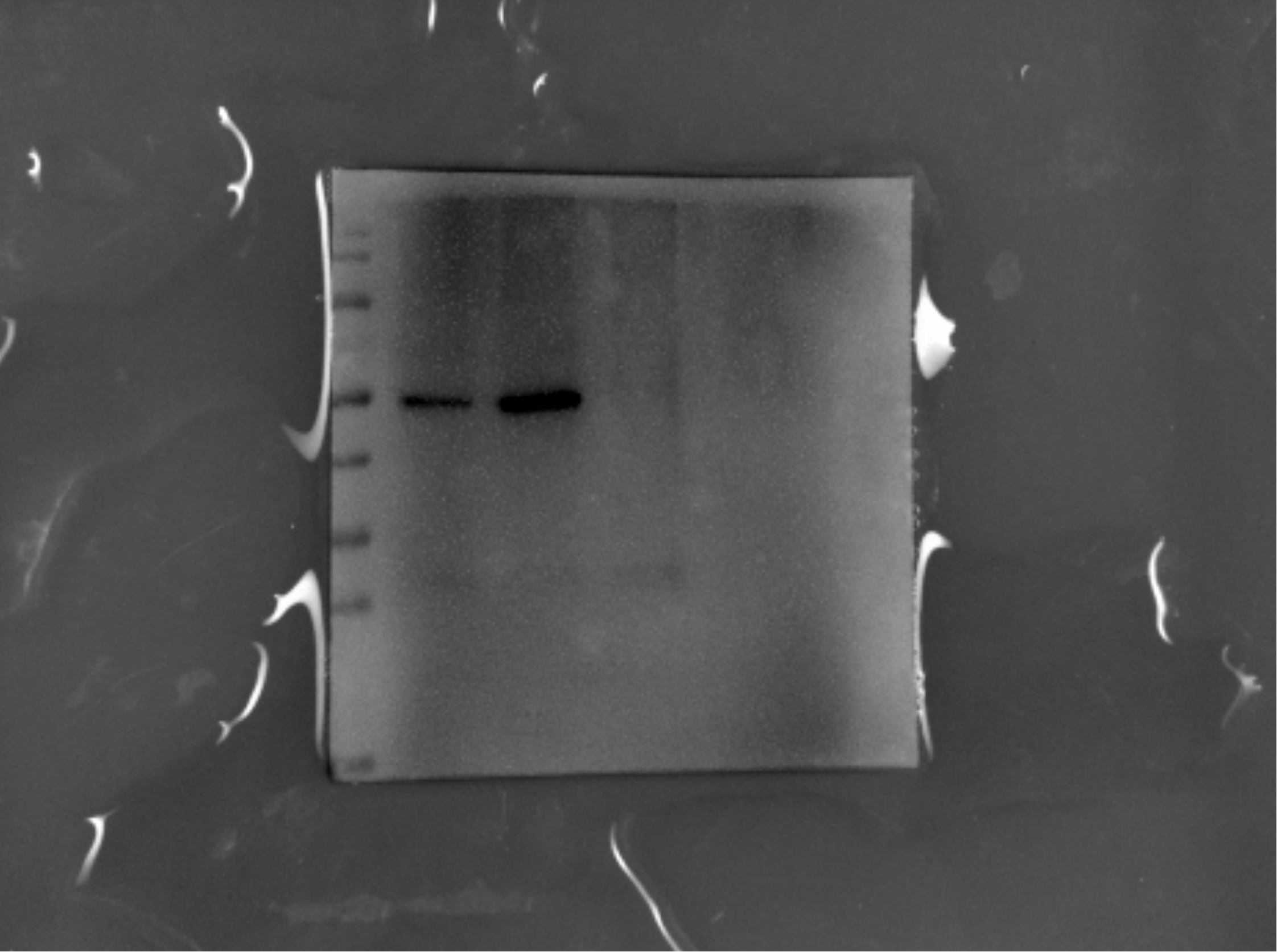

Supplement: Supplementary file 1 [file vetsci-12-00257-s001.zip › PABPC4 original blot images/Fig.3/C/IP FLAG/FLAG/merge.tif]

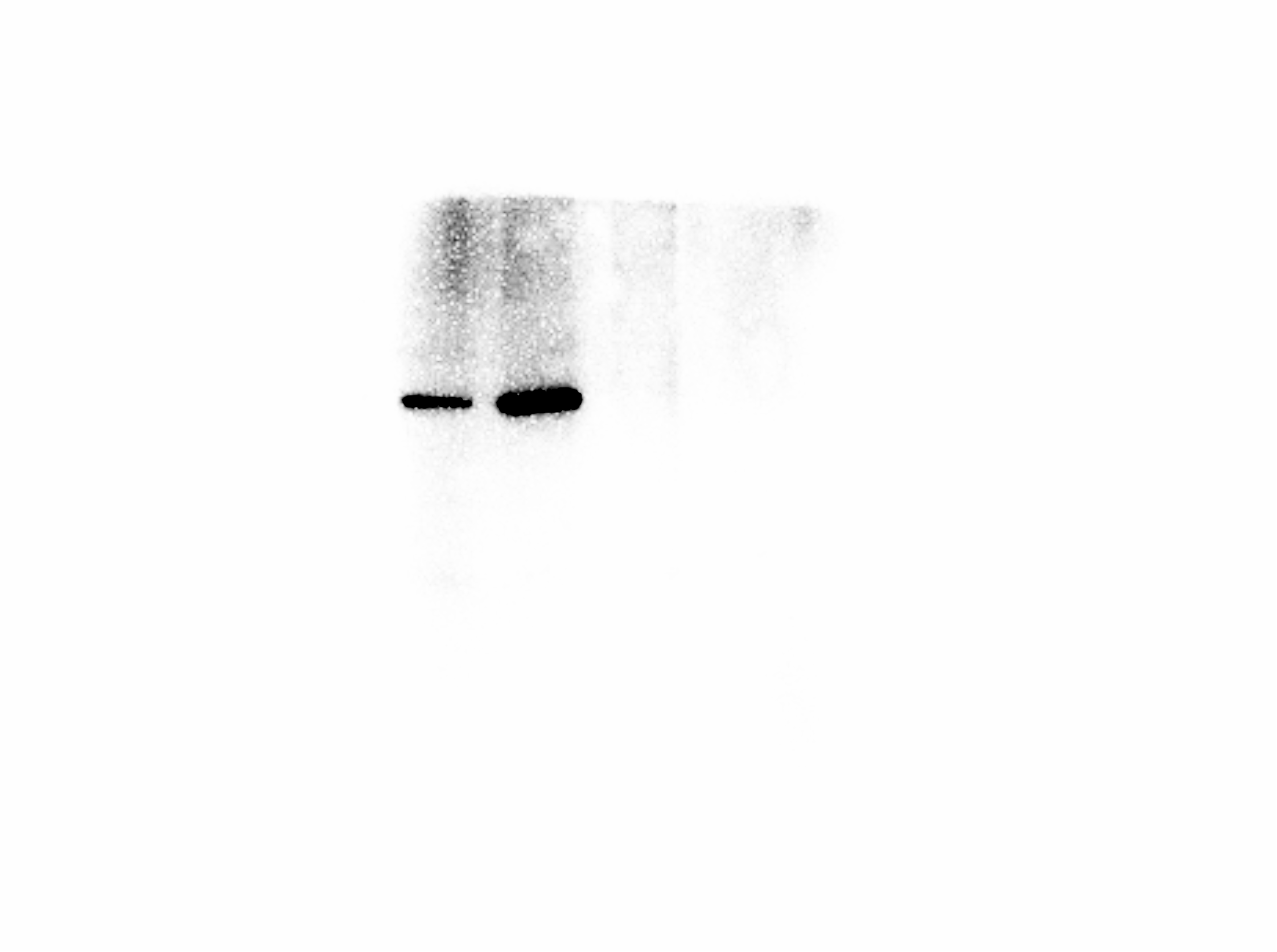

Supplement: Supplementary file 1 [file vetsci-12-00257-s001.zip › PABPC4 original blot images/Fig.3/C/IP FLAG/FLAG/shiyantu.tif]

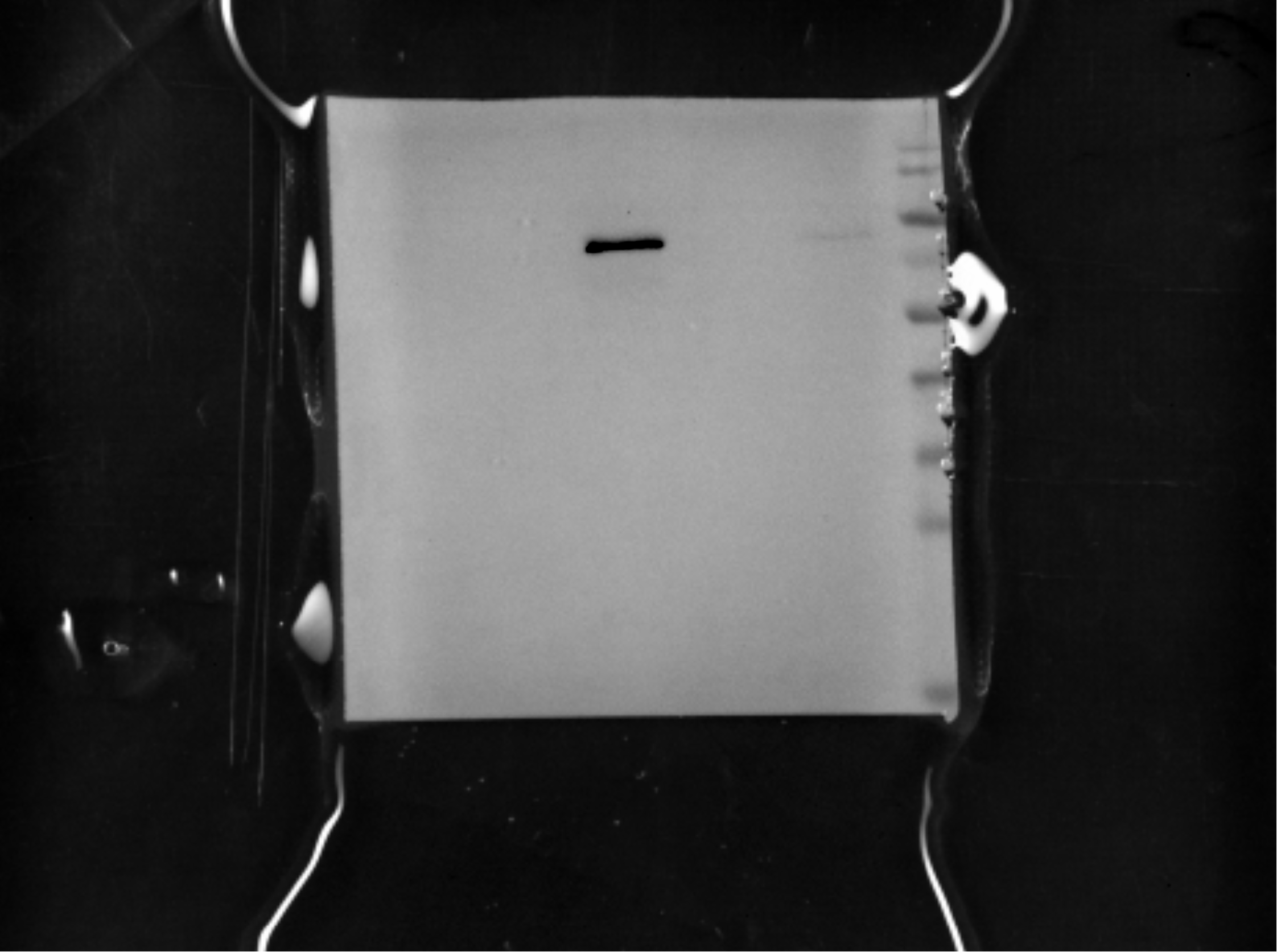

Supplement: Supplementary file 1 [file vetsci-12-00257-s001.zip › PABPC4 original blot images/Fig.3/C/IP FLAG/HA/MERGE.tif]

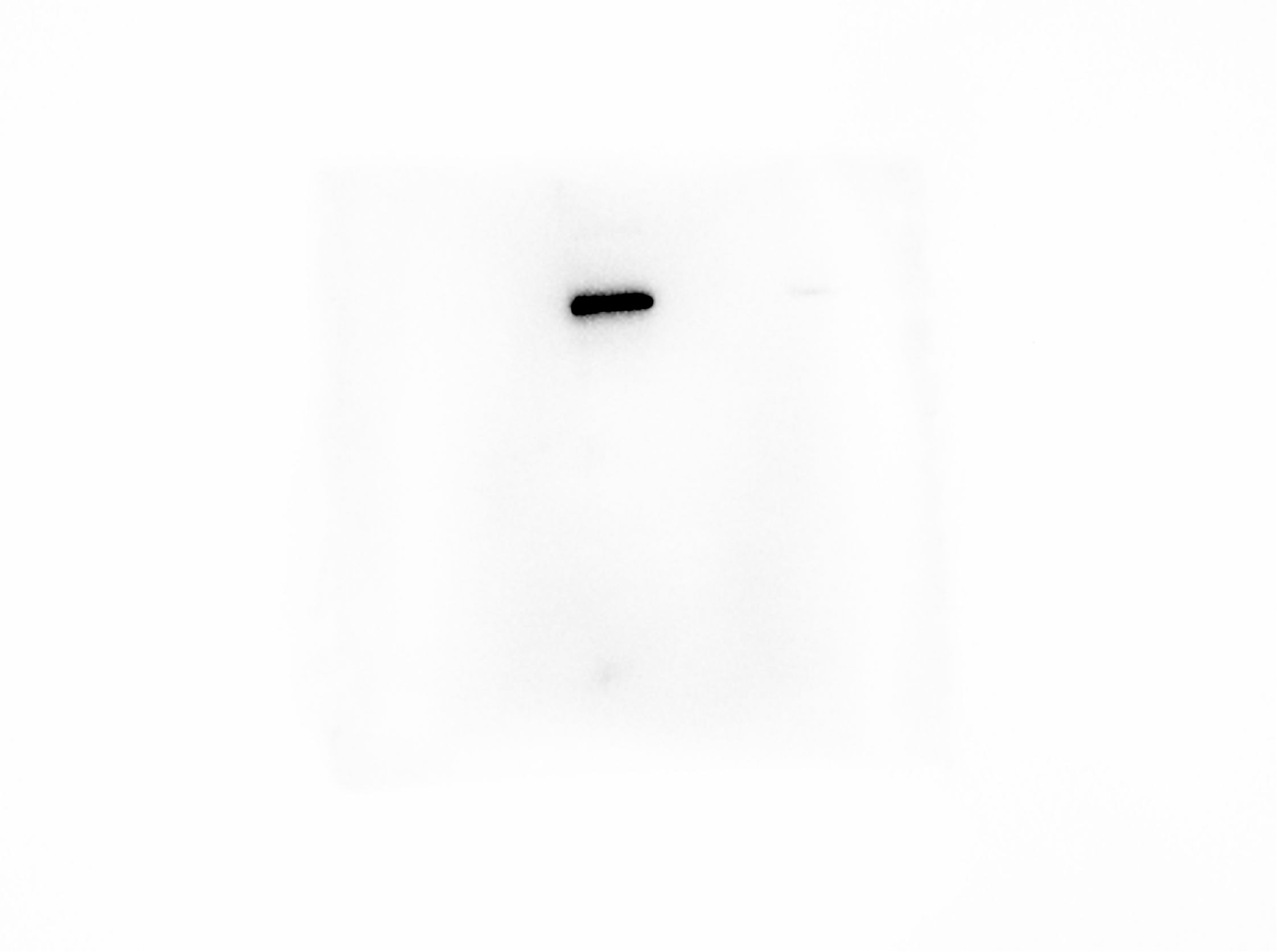

Supplement: Supplementary file 1 [file vetsci-12-00257-s001.zip › PABPC4 original blot images/Fig.3/C/IP FLAG/HA/SHIYANTU2.tif]

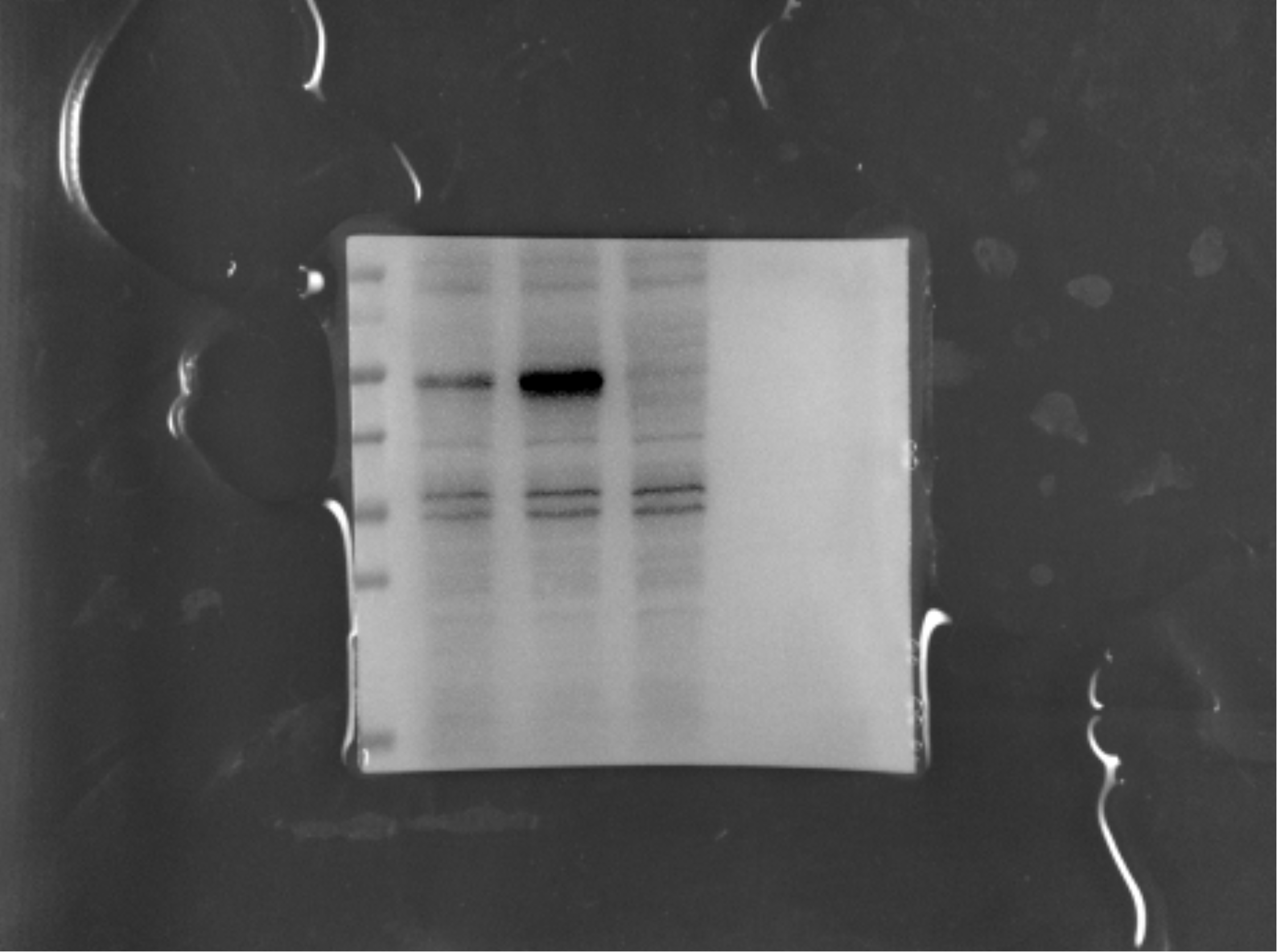

Supplement: Supplementary file 1 [file vetsci-12-00257-s001.zip › PABPC4 original blot images/Fig.3/C/IP FLAG/IB/flag/merge.tif]

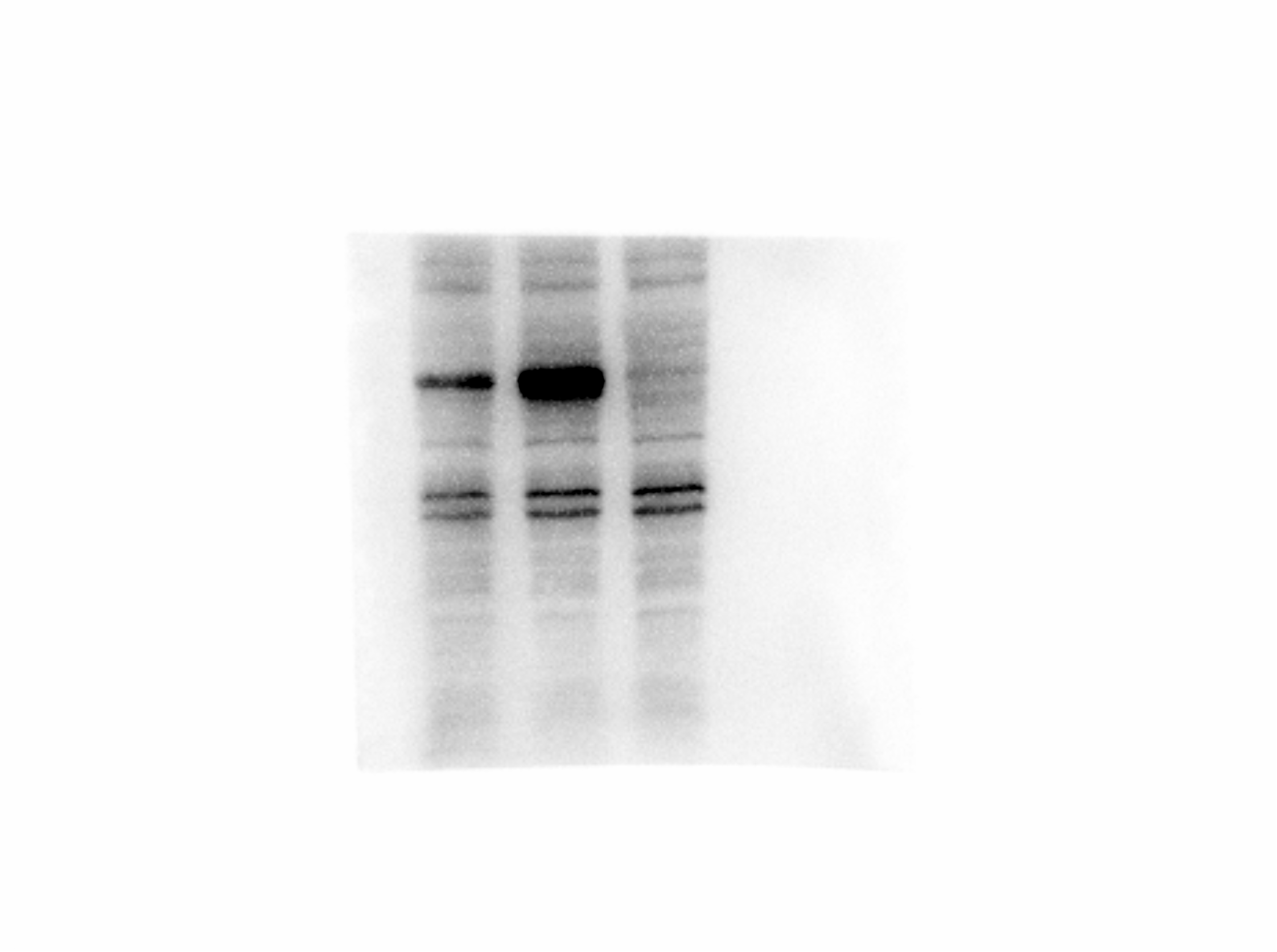

Supplement: Supplementary file 1 [file vetsci-12-00257-s001.zip › PABPC4 original blot images/Fig.3/C/IP FLAG/IB/flag/shiyantu.tif]

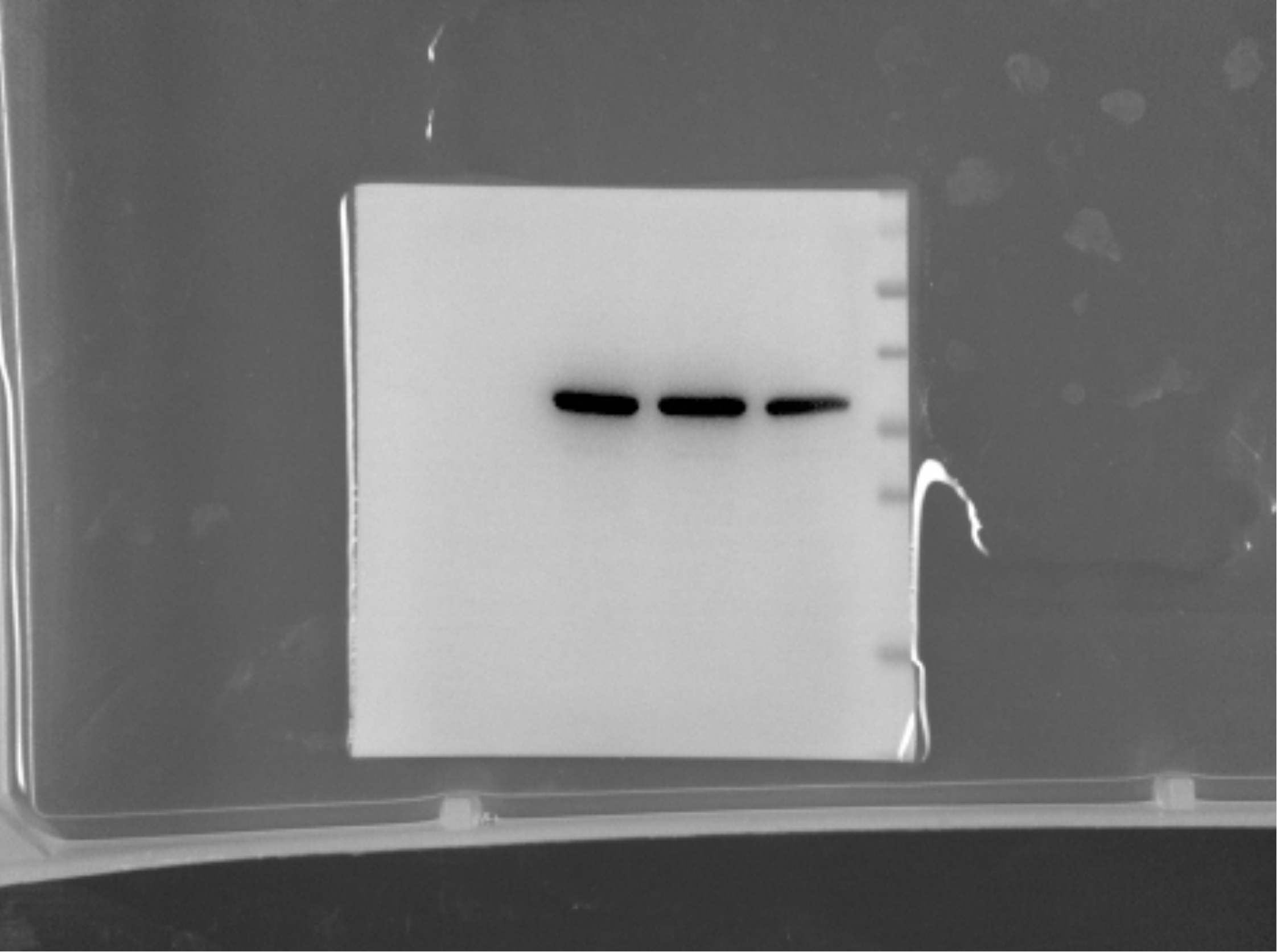

Supplement: Supplementary file 1 [file vetsci-12-00257-s001.zip › PABPC4 original blot images/Fig.3/C/IP FLAG/IB/gapdh/merge.tif]

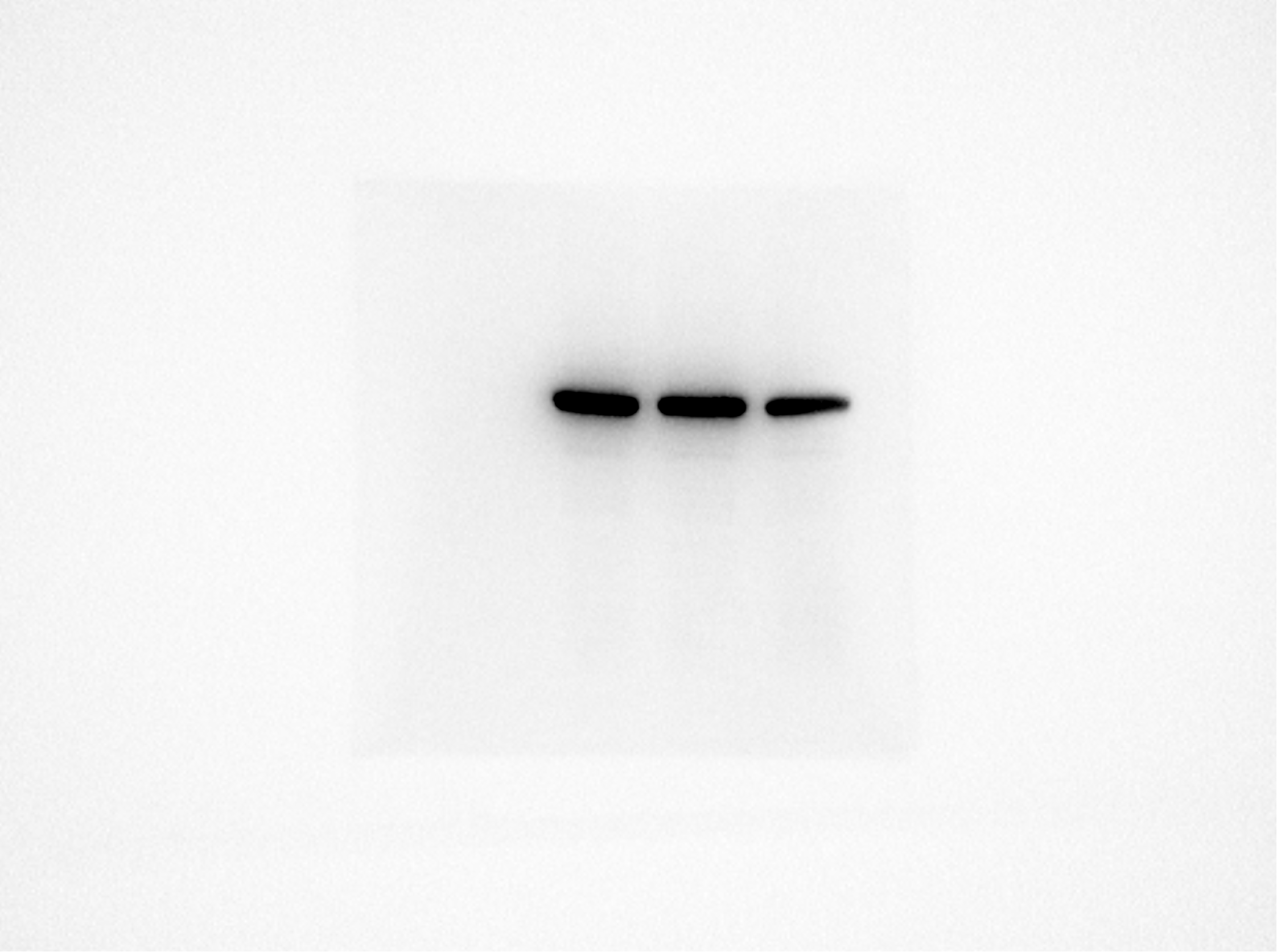

Supplement: Supplementary file 1 [file vetsci-12-00257-s001.zip › PABPC4 original blot images/Fig.3/C/IP FLAG/IB/gapdh/shiyantu.tif]

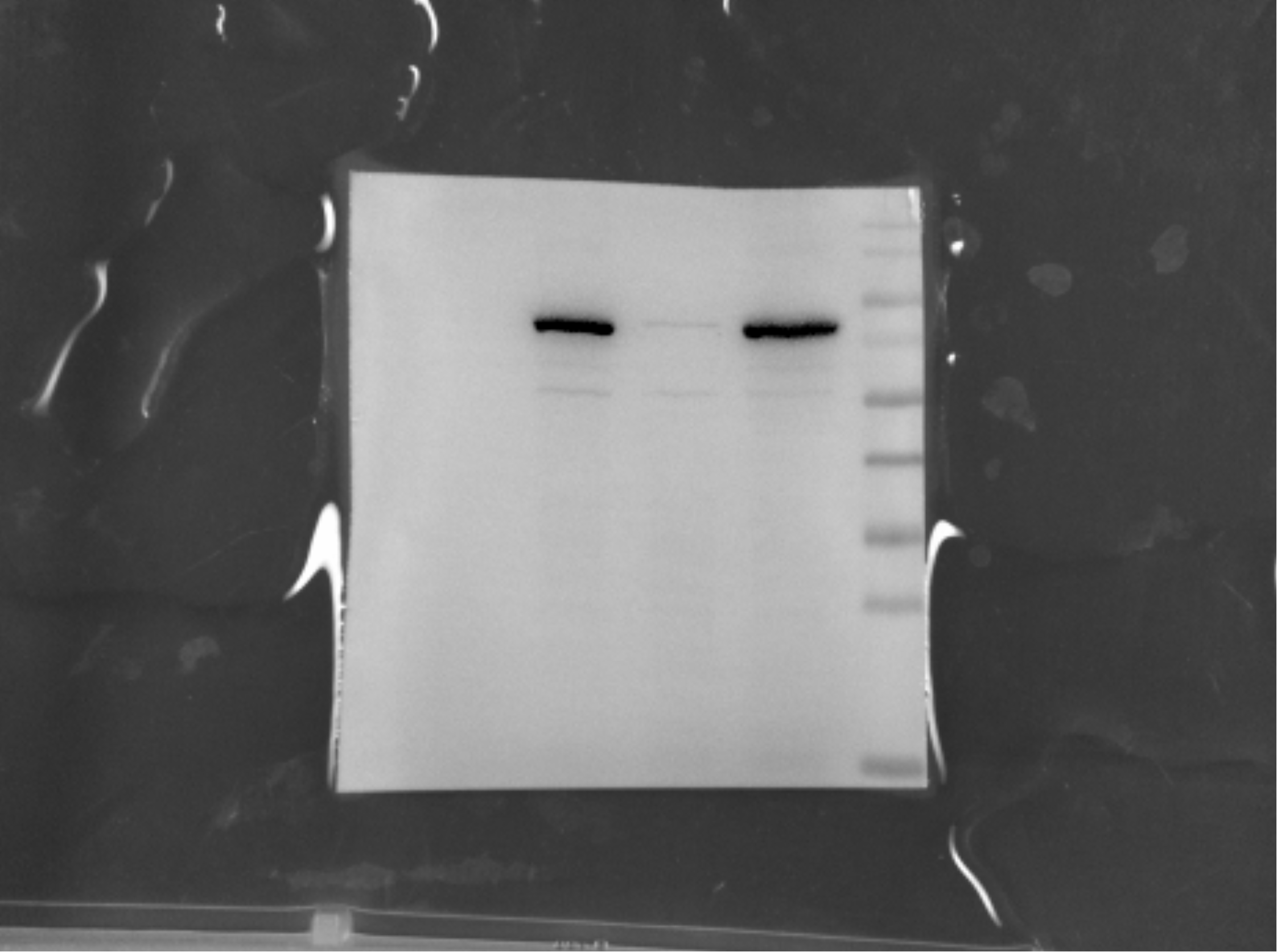

Supplement: Supplementary file 1 [file vetsci-12-00257-s001.zip › PABPC4 original blot images/Fig.3/C/IP FLAG/IB/ha/merge.tif]

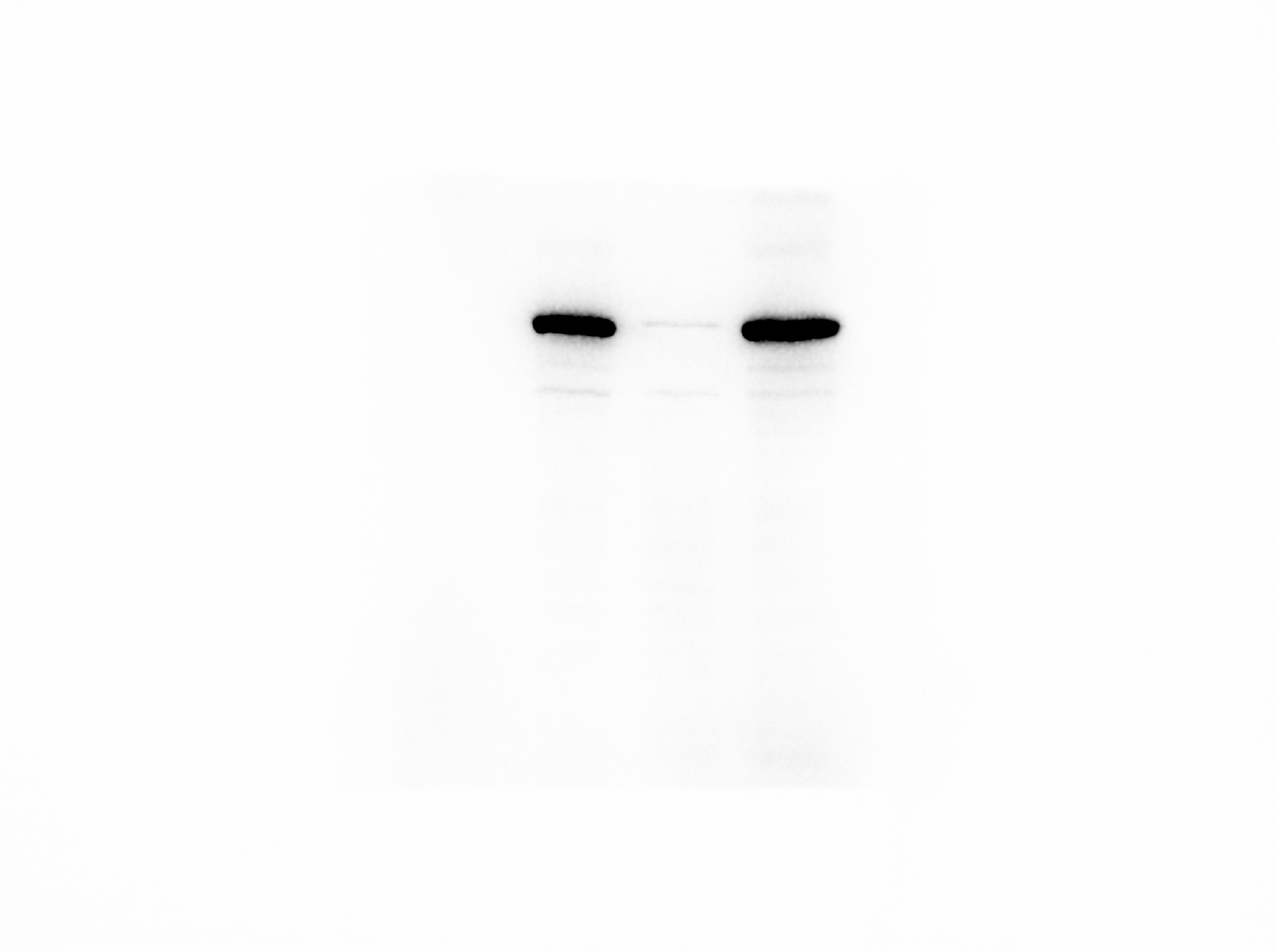

Supplement: Supplementary file 1 [file vetsci-12-00257-s001.zip › PABPC4 original blot images/Fig.3/C/IP FLAG/IB/ha/shiyantu.tif]

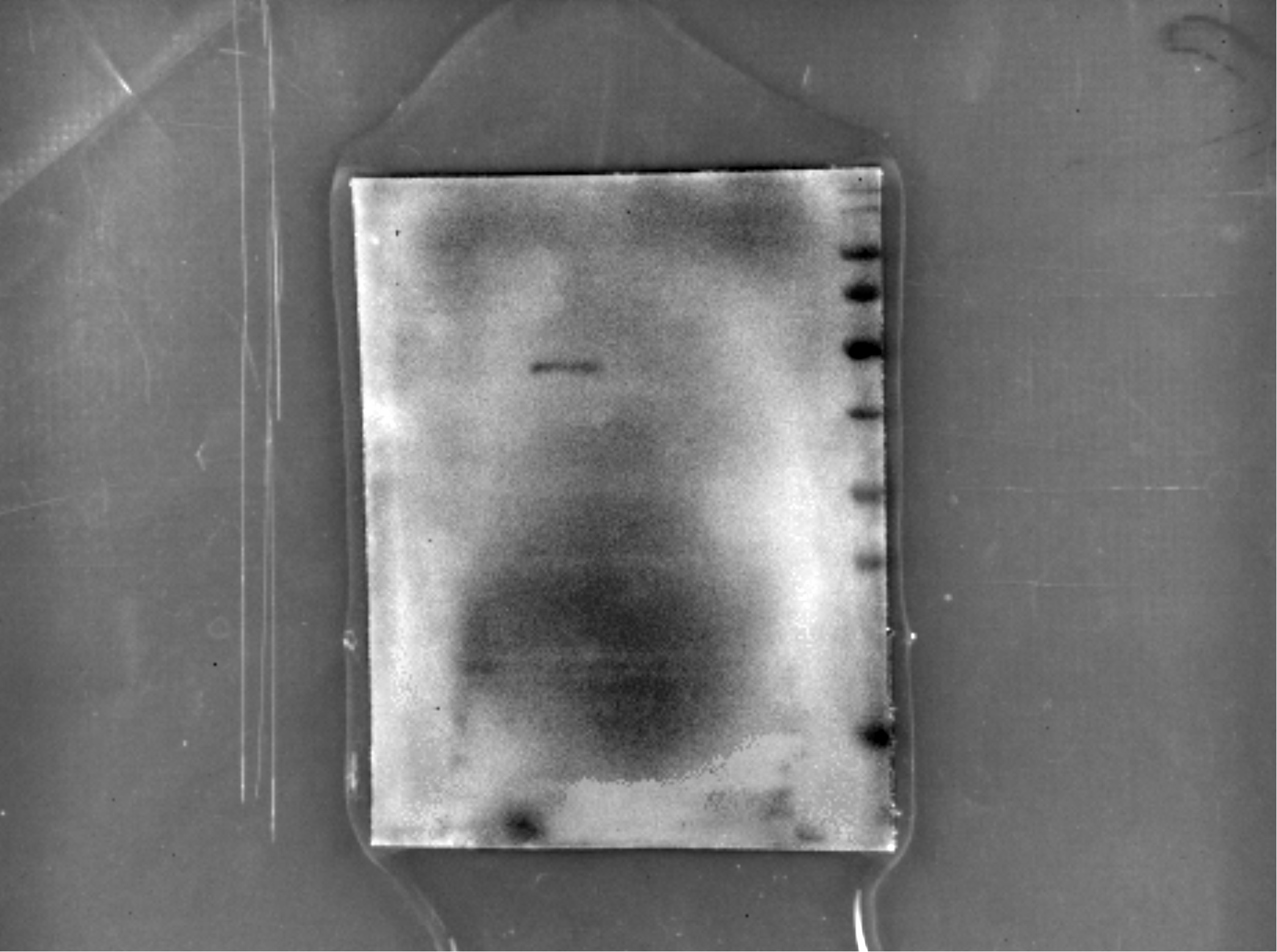

Supplement: Supplementary file 1 [file vetsci-12-00257-s001.zip › PABPC4 original blot images/Fig.3/C/IP HA/FLAG/MERGE.tif]

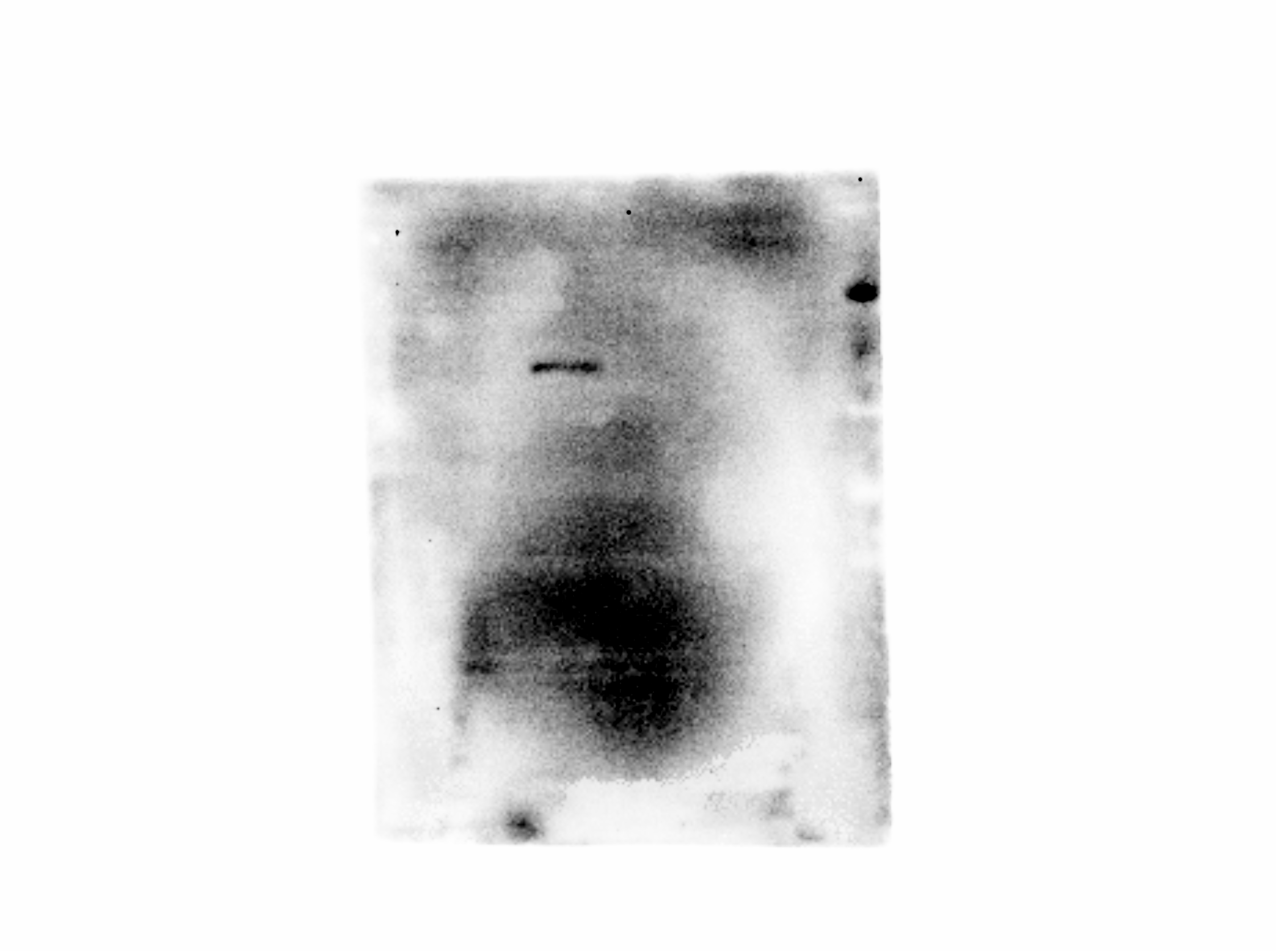

Supplement: Supplementary file 1 [file vetsci-12-00257-s001.zip › PABPC4 original blot images/Fig.3/C/IP HA/FLAG/SHIYANTU.tif]

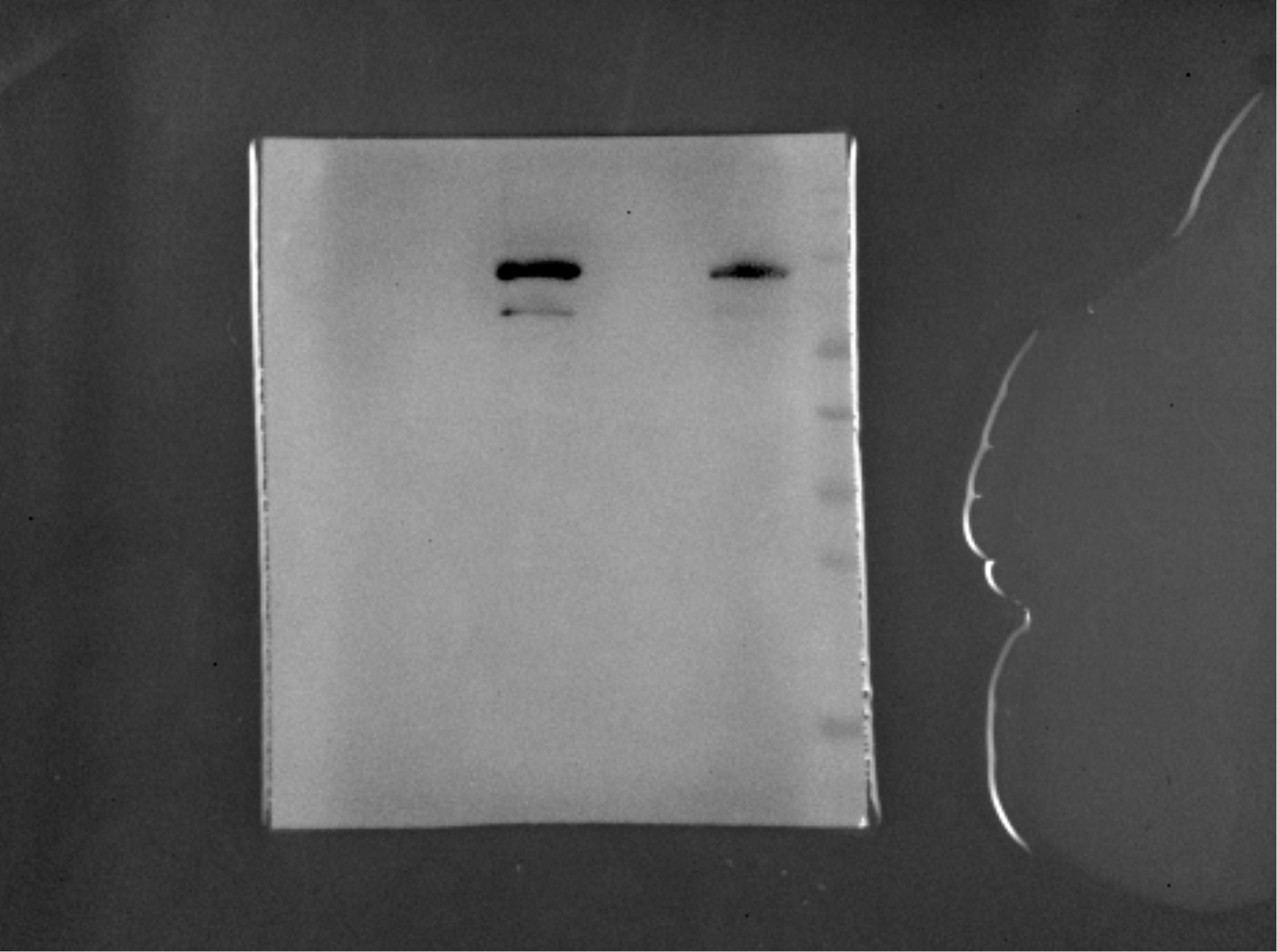

Supplement: Supplementary file 1 [file vetsci-12-00257-s001.zip › PABPC4 original blot images/Fig.3/C/IP HA/HA/MERGE.tif]

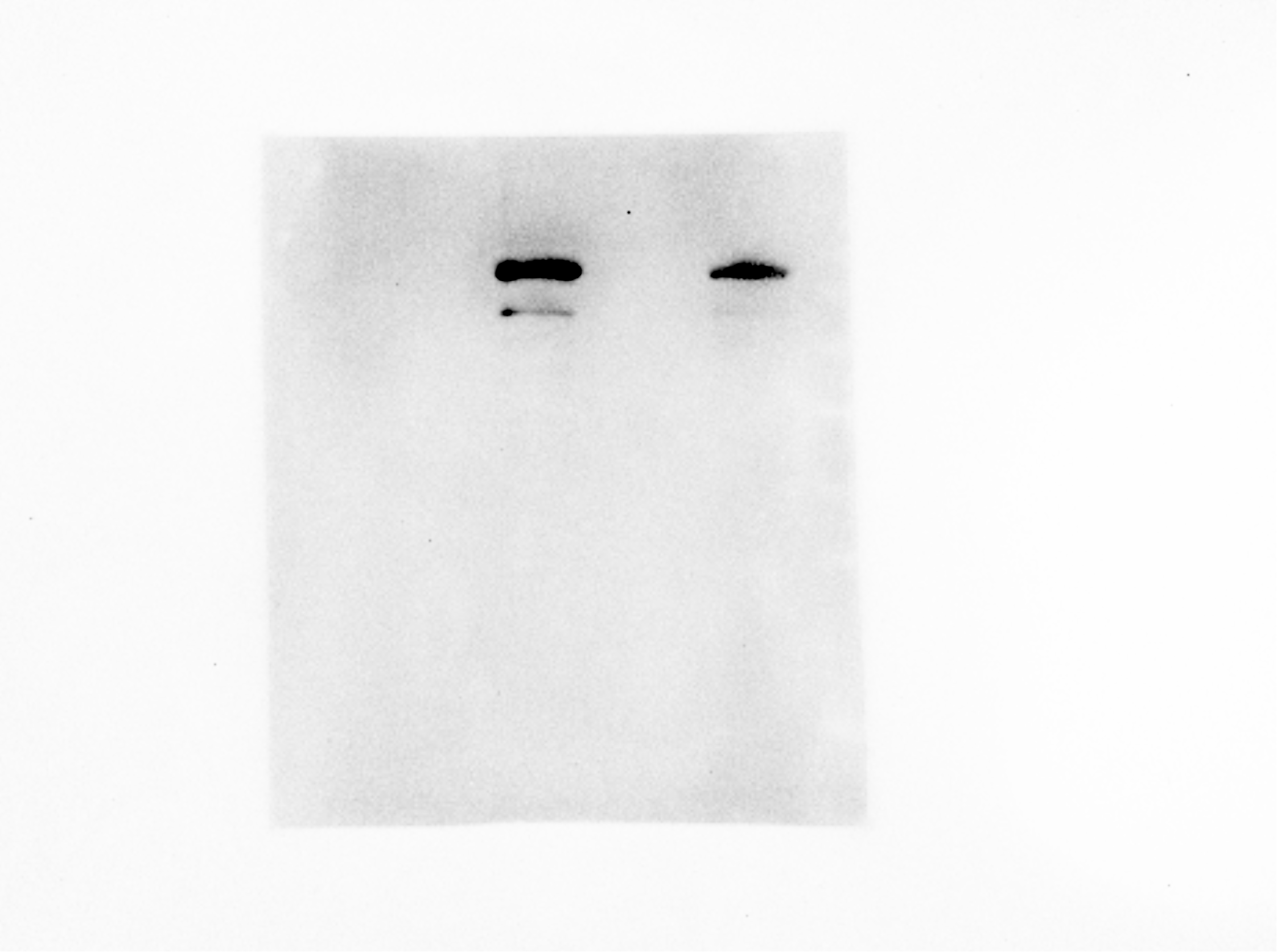

Supplement: Supplementary file 1 [file vetsci-12-00257-s001.zip › PABPC4 original blot images/Fig.3/C/IP HA/HA/SHIYANTU.tif]

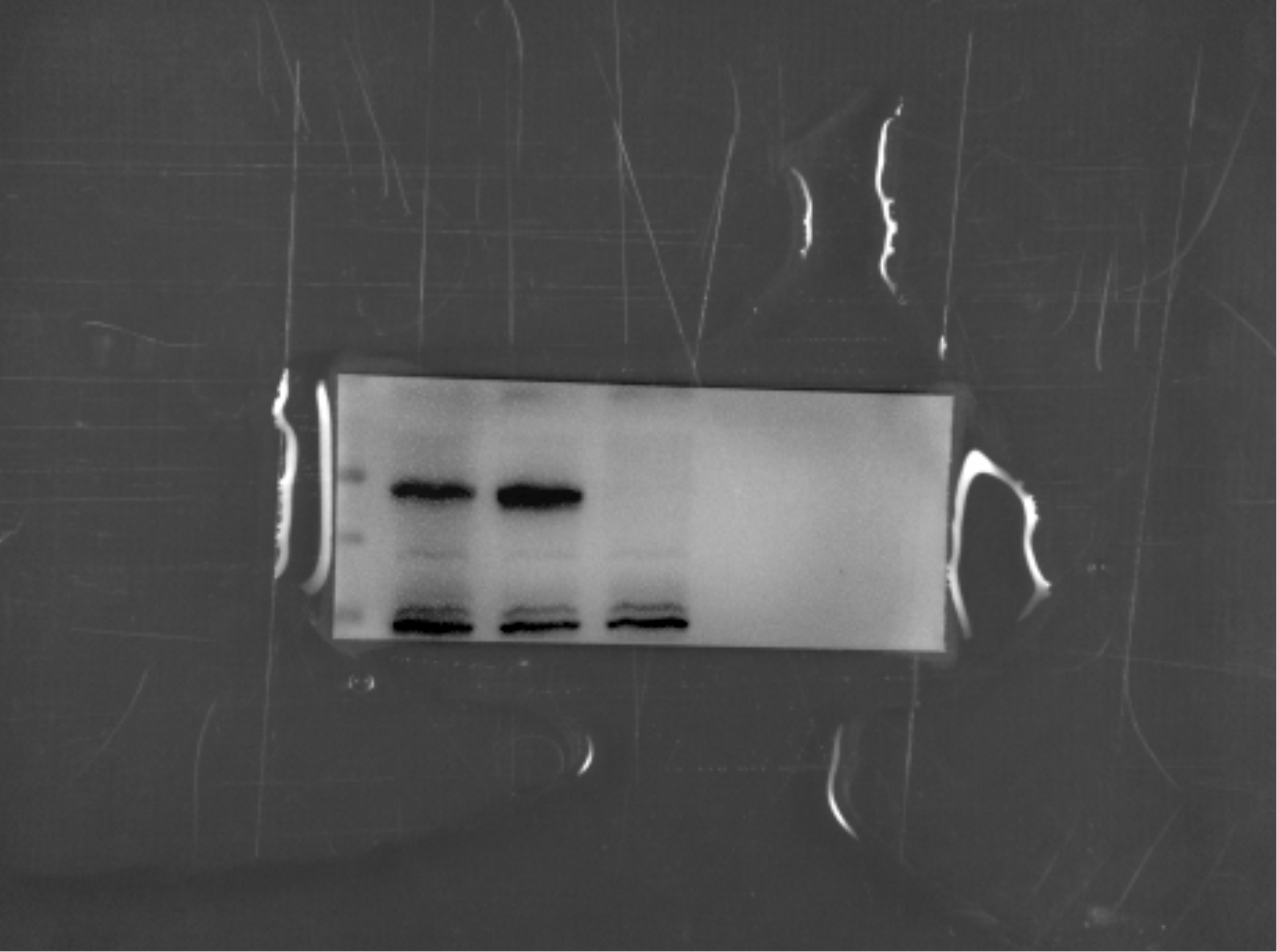

Supplement: Supplementary file 1 [file vetsci-12-00257-s001.zip › PABPC4 original blot images/Fig.3/C/IP HA/IB/flag/merge.tif]

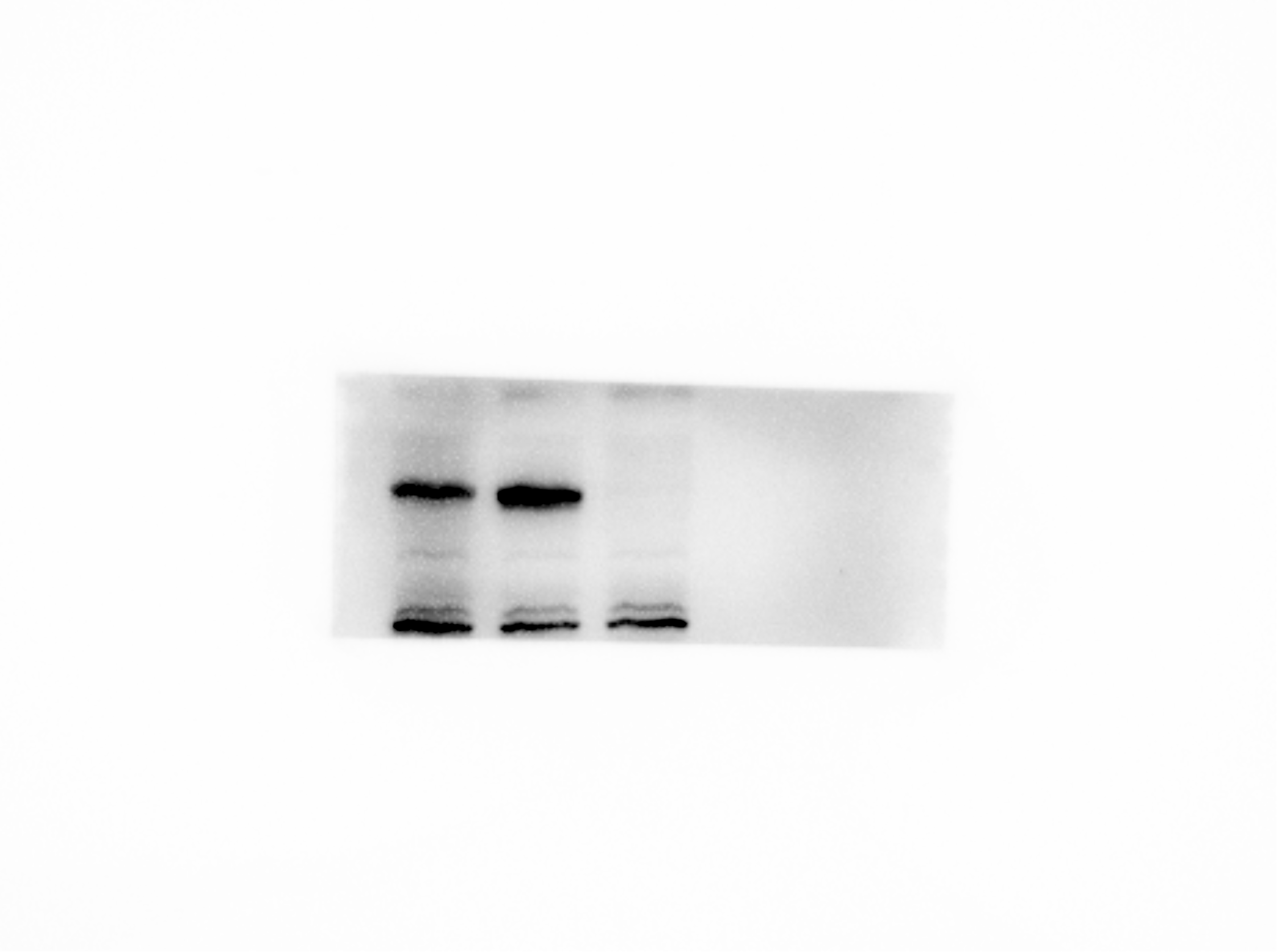

Supplement: Supplementary file 1 [file vetsci-12-00257-s001.zip › PABPC4 original blot images/Fig.3/C/IP HA/IB/flag/shiyantu.tif]

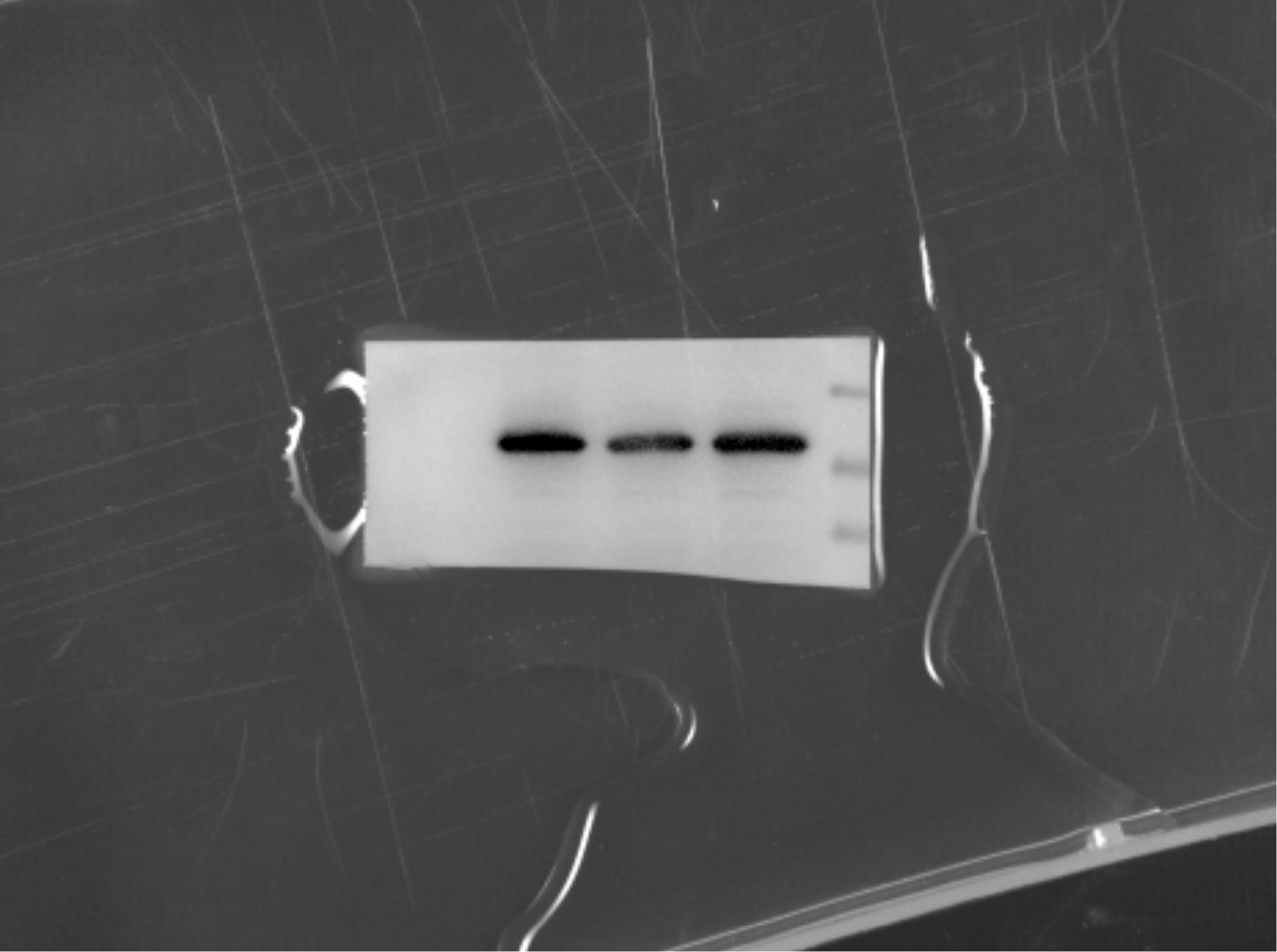

Supplement: Supplementary file 1 [file vetsci-12-00257-s001.zip › PABPC4 original blot images/Fig.3/C/IP HA/IB/gapdh/merge.tif]

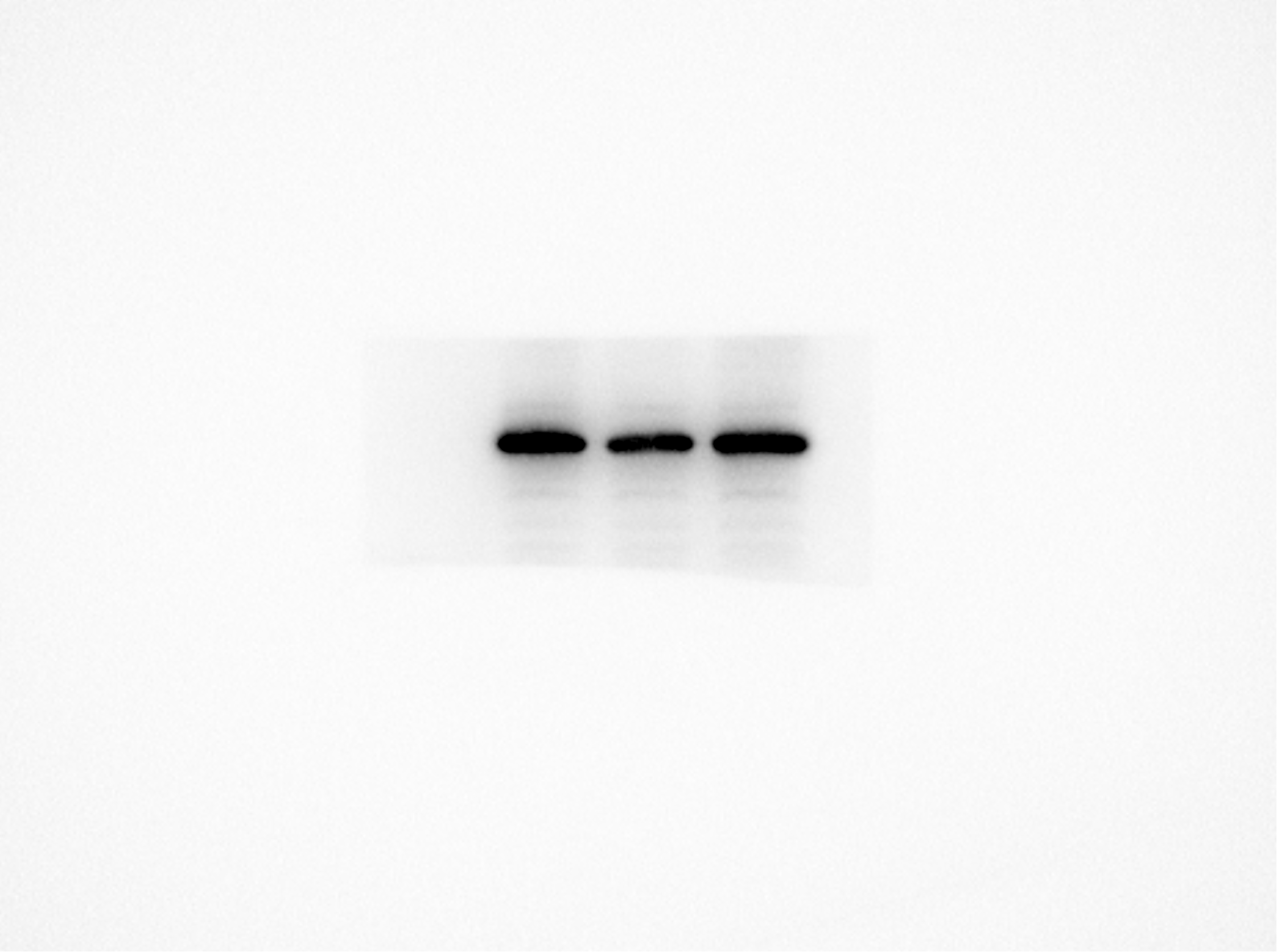

Supplement: Supplementary file 1 [file vetsci-12-00257-s001.zip › PABPC4 original blot images/Fig.3/C/IP HA/IB/gapdh/shiyantu.tif]

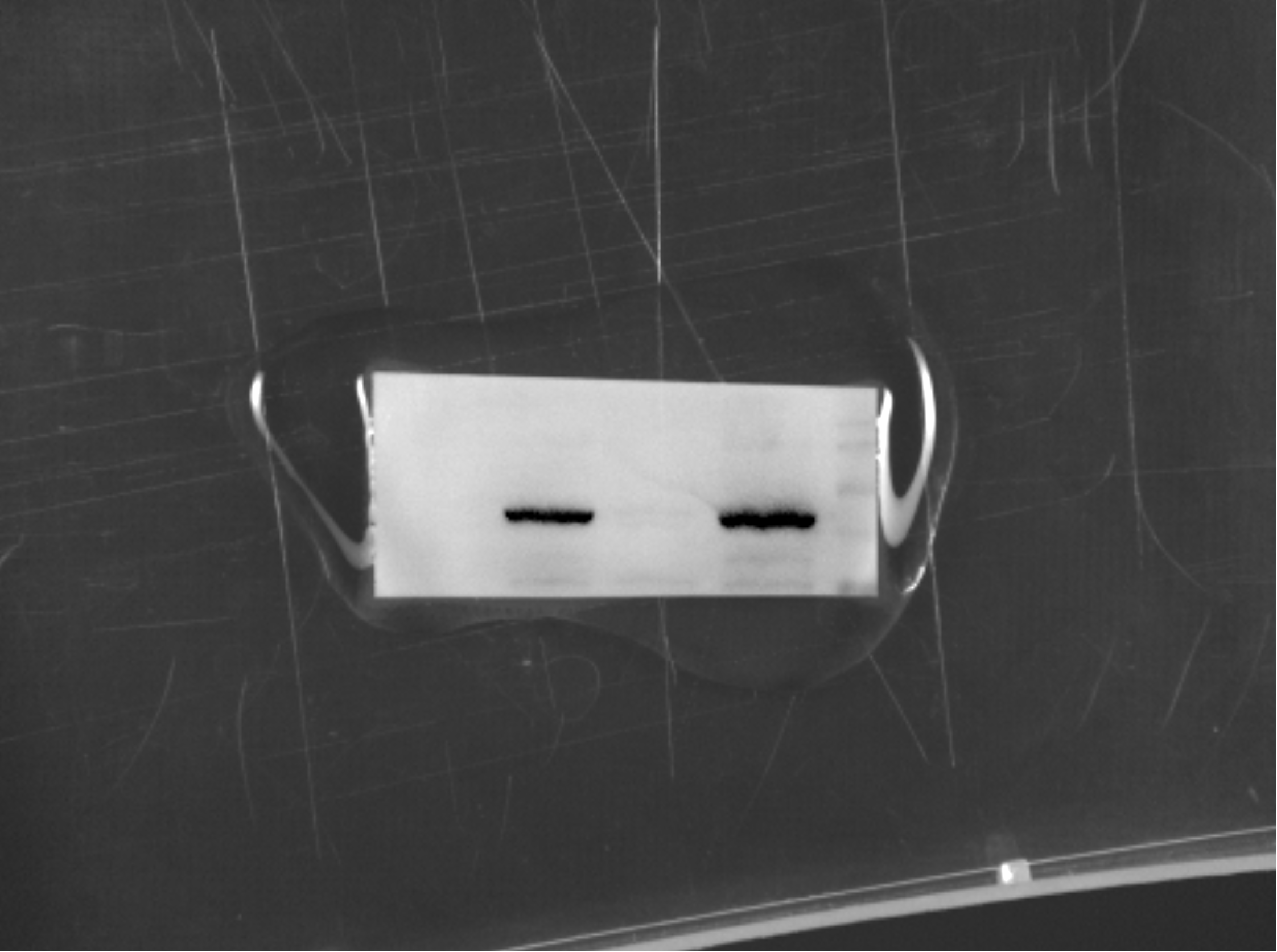

Supplement: Supplementary file 1 [file vetsci-12-00257-s001.zip › PABPC4 original blot images/Fig.3/C/IP HA/IB/ha/ha/merge.tif]

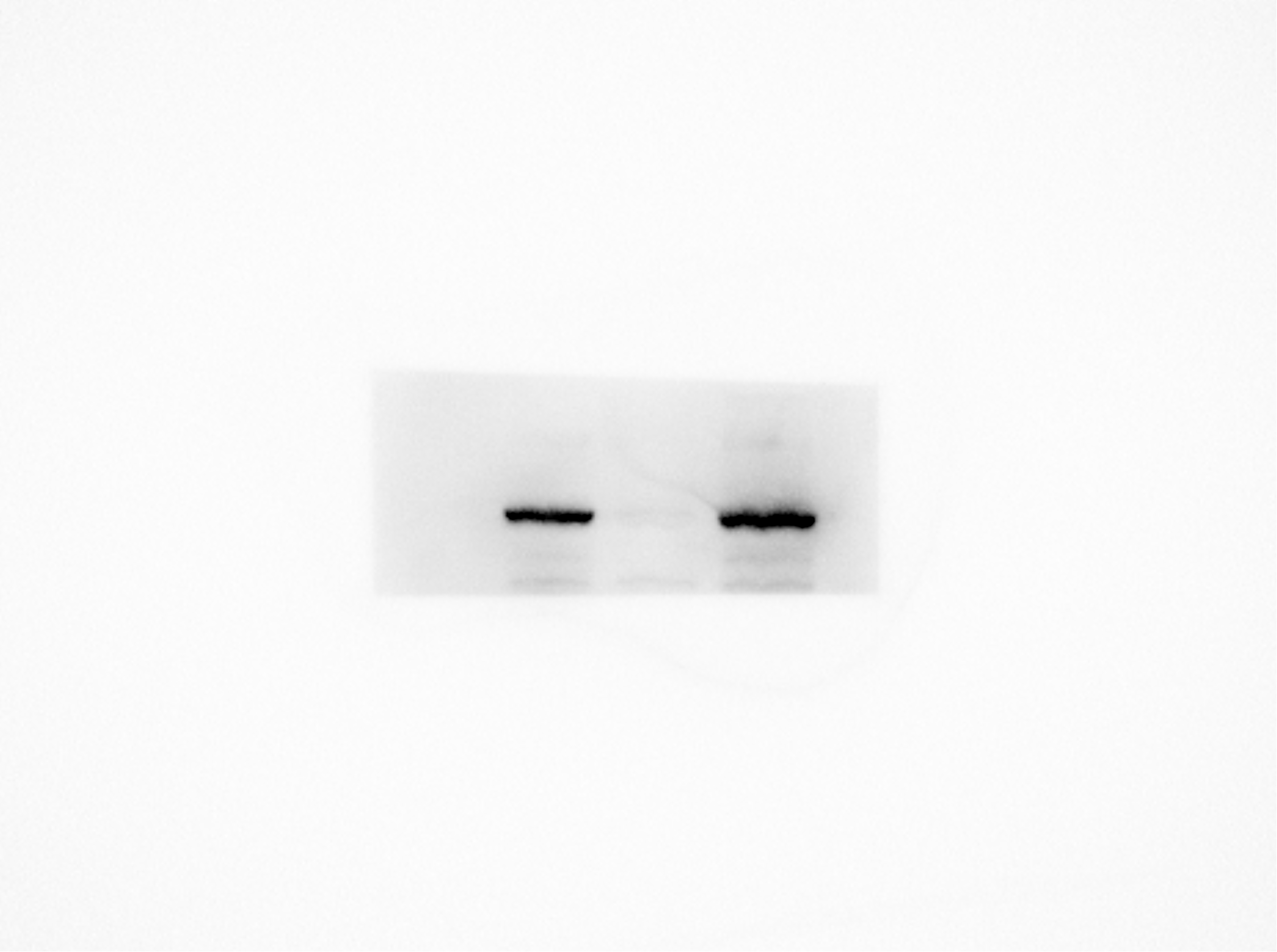

Supplement: Supplementary file 1 [file vetsci-12-00257-s001.zip › PABPC4 original blot images/Fig.3/C/IP HA/IB/ha/ha/shiyantu.tif]

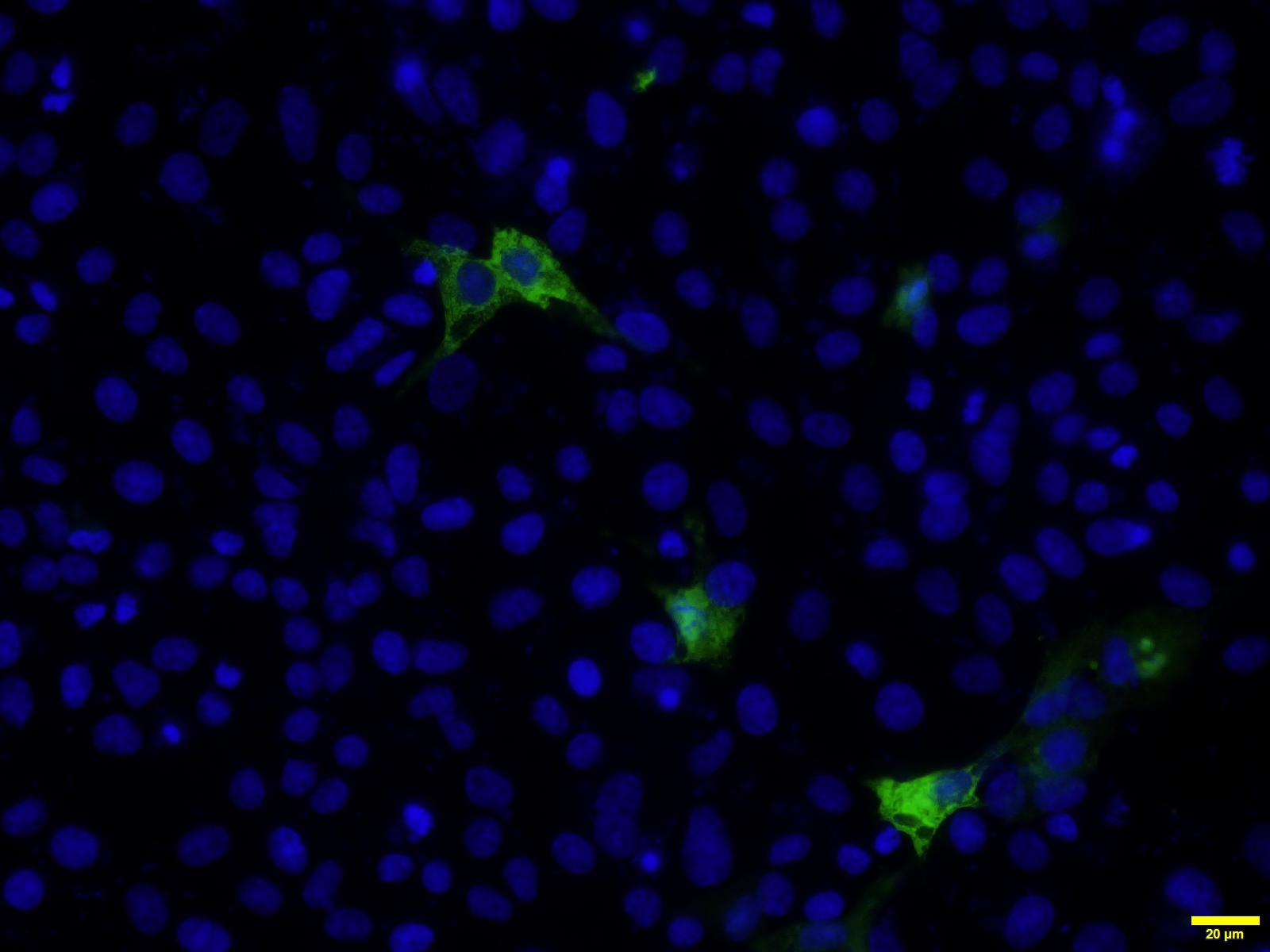

Supplement: Supplementary file 1 [file vetsci-12-00257-s001.zip › PABPC4 original blot images/Fig.3/D/N/图像_03.jpg]

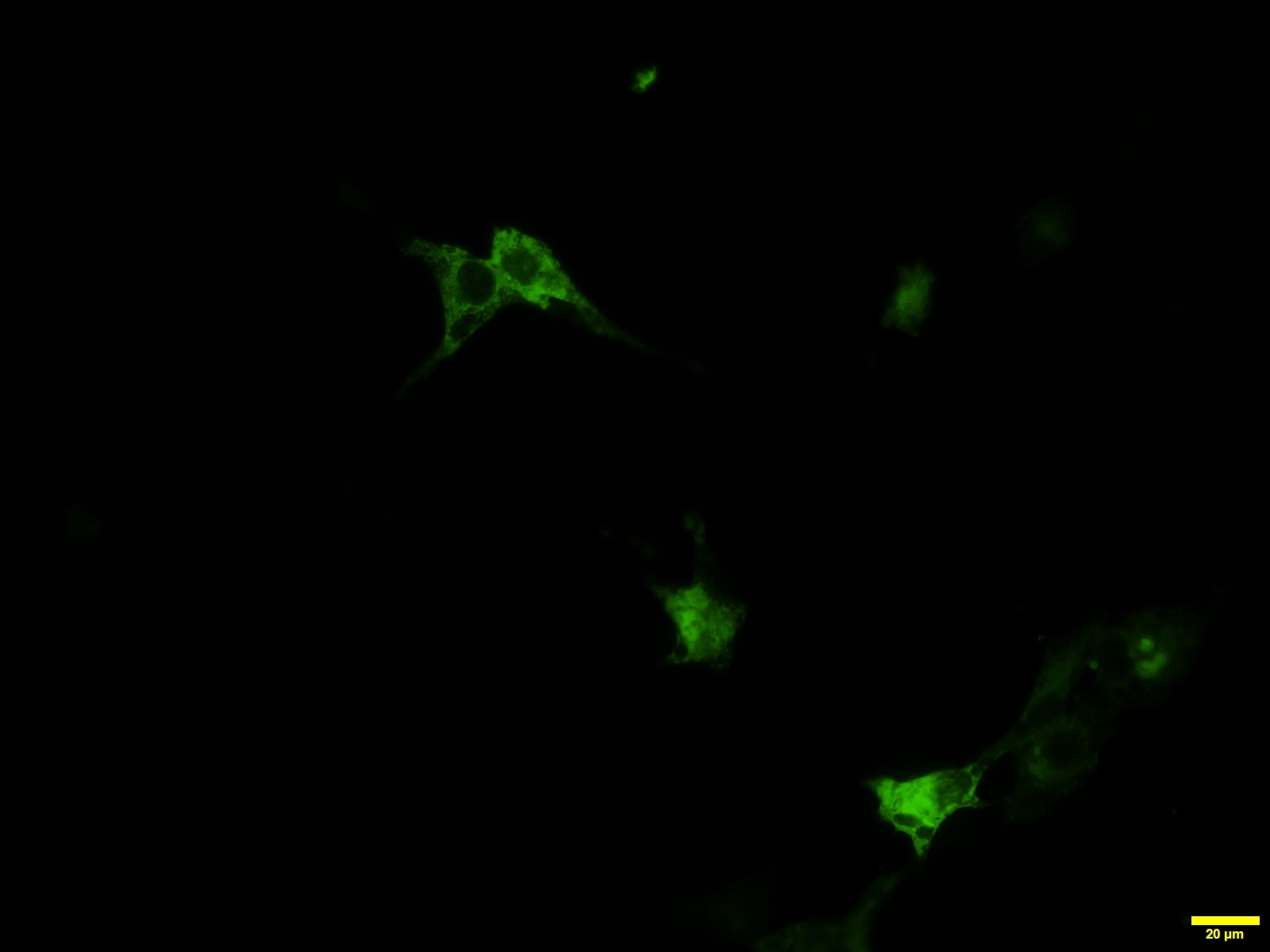

Supplement: Supplementary file 1 [file vetsci-12-00257-s001.zip › PABPC4 original blot images/Fig.3/D/N/图像_12607.jpg]

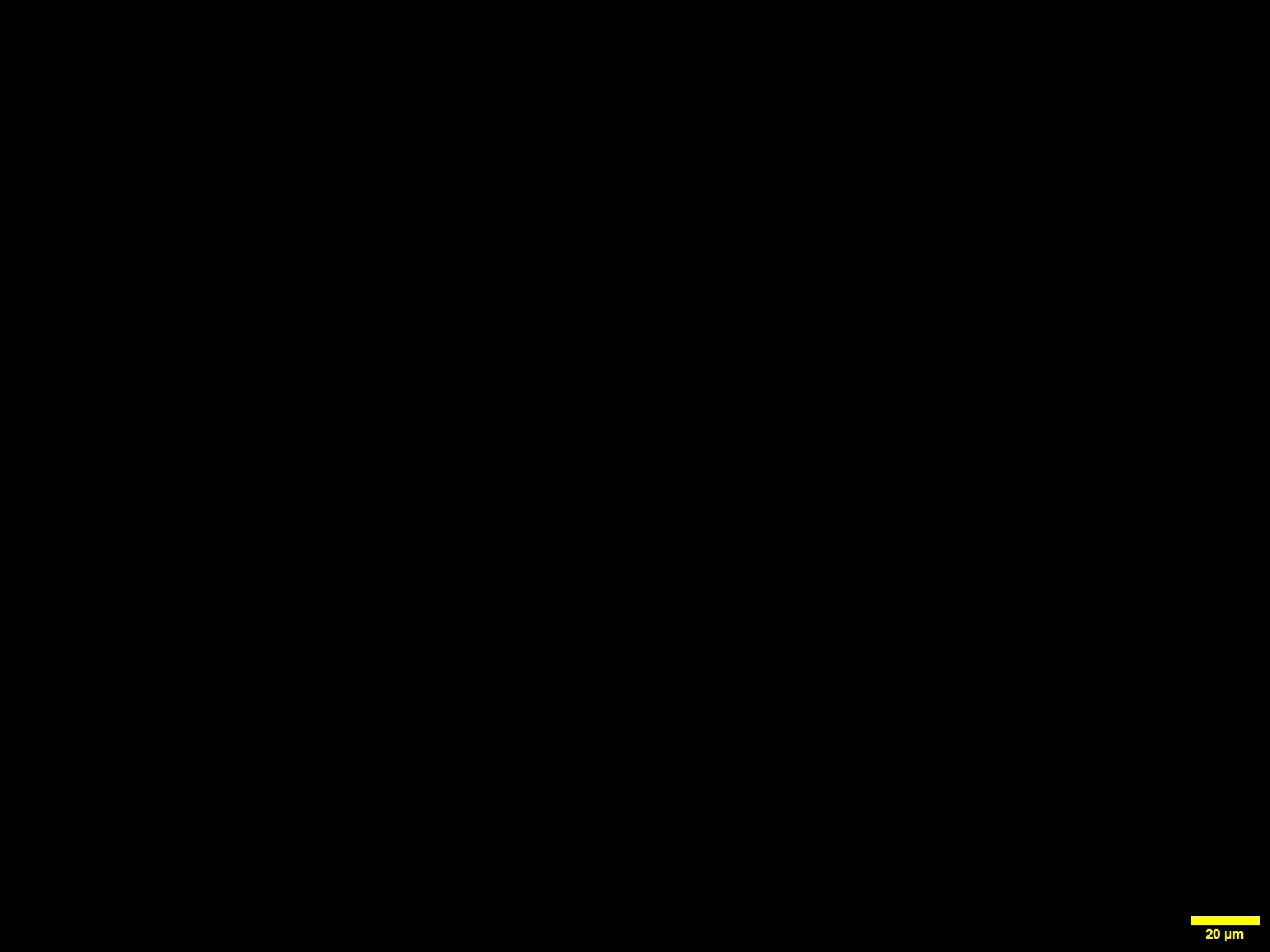

Supplement: Supplementary file 1 [file vetsci-12-00257-s001.zip › PABPC4 original blot images/Fig.3/D/N/图像_12608.jpg]

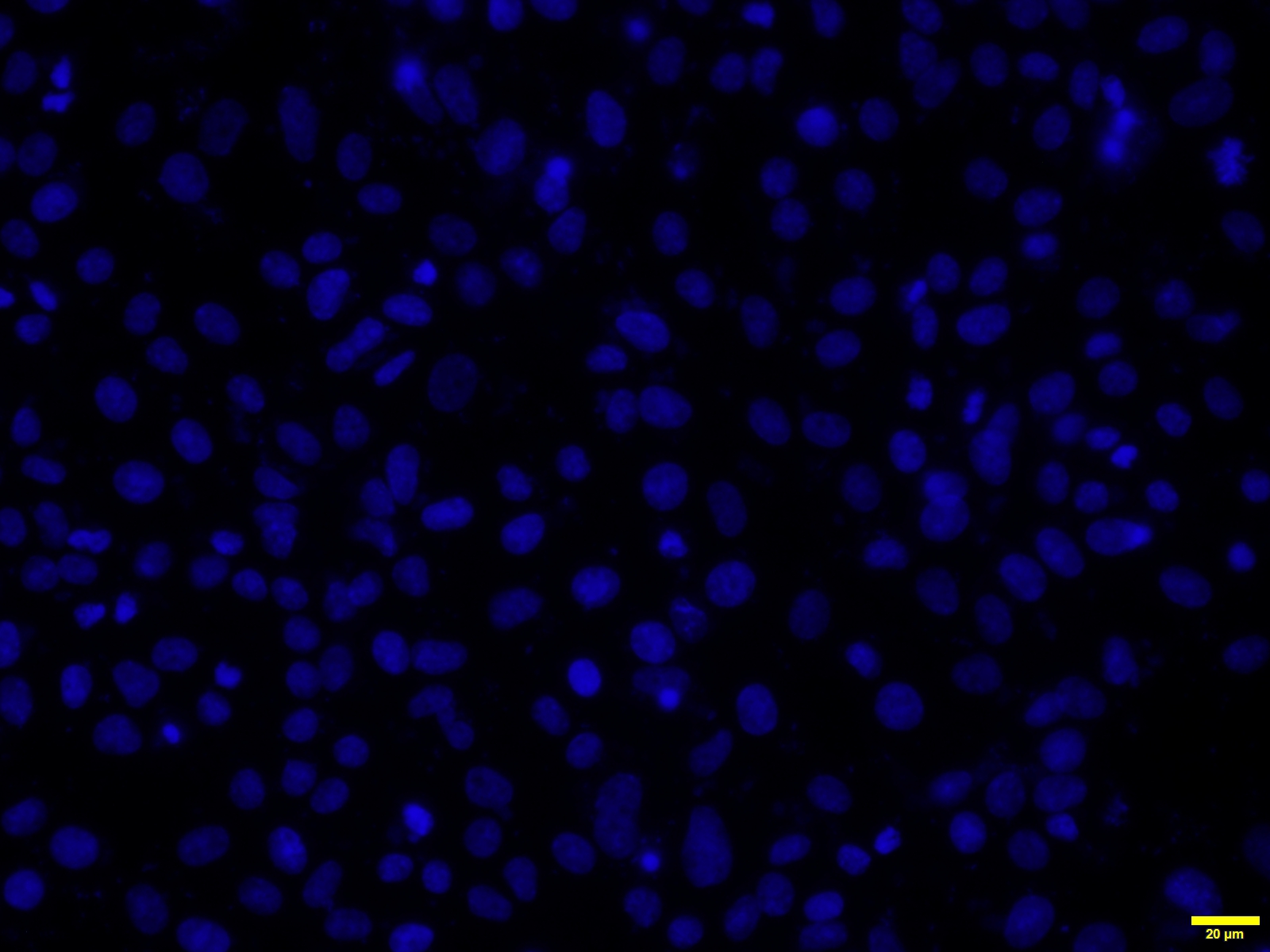

Supplement: Supplementary file 1 [file vetsci-12-00257-s001.zip › PABPC4 original blot images/Fig.3/D/N/图像_12609.jpg]

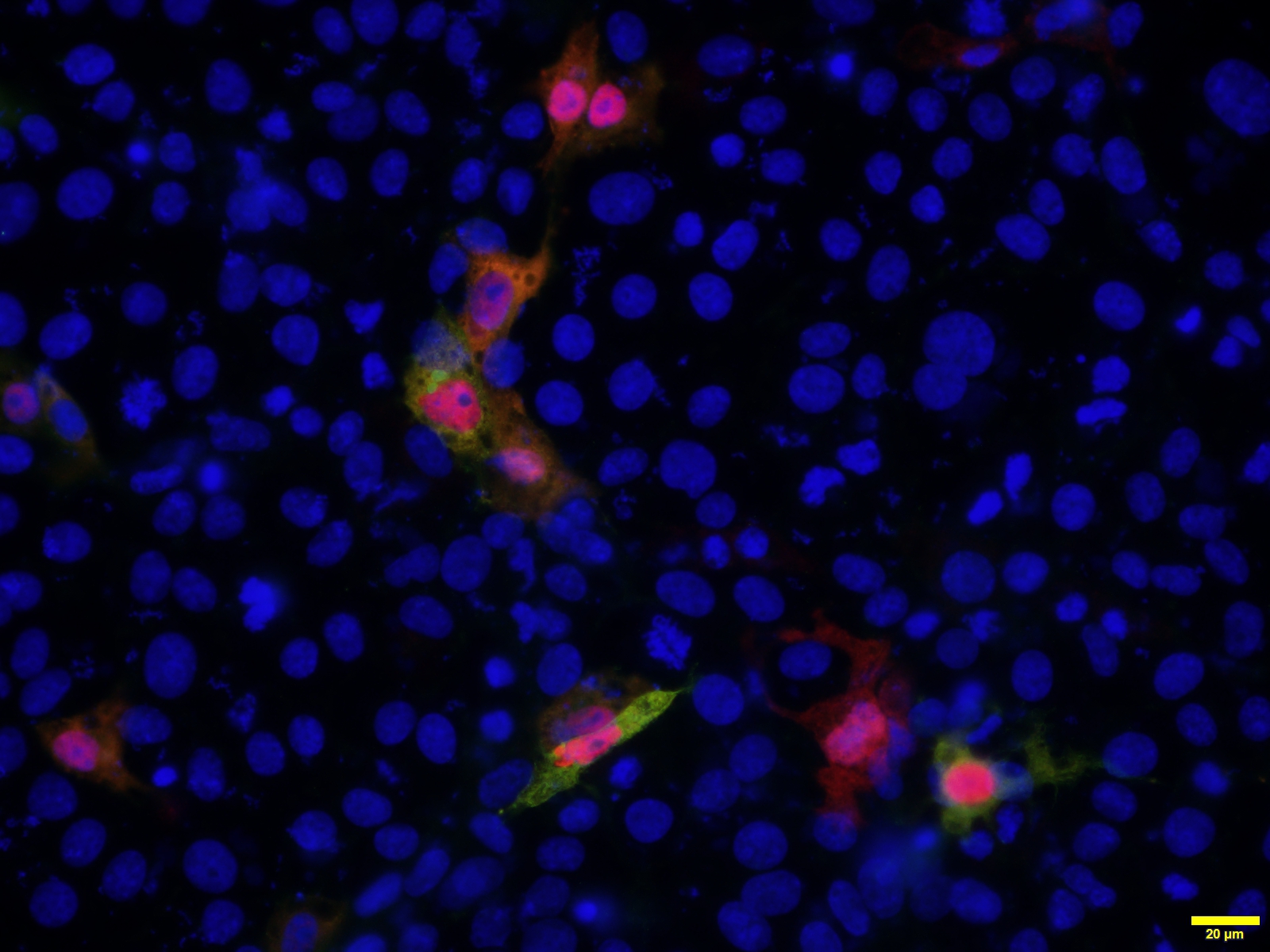

Supplement: Supplementary file 1 [file vetsci-12-00257-s001.zip › PABPC4 original blot images/Fig.3/D/N+P4/图像_03.jpg]

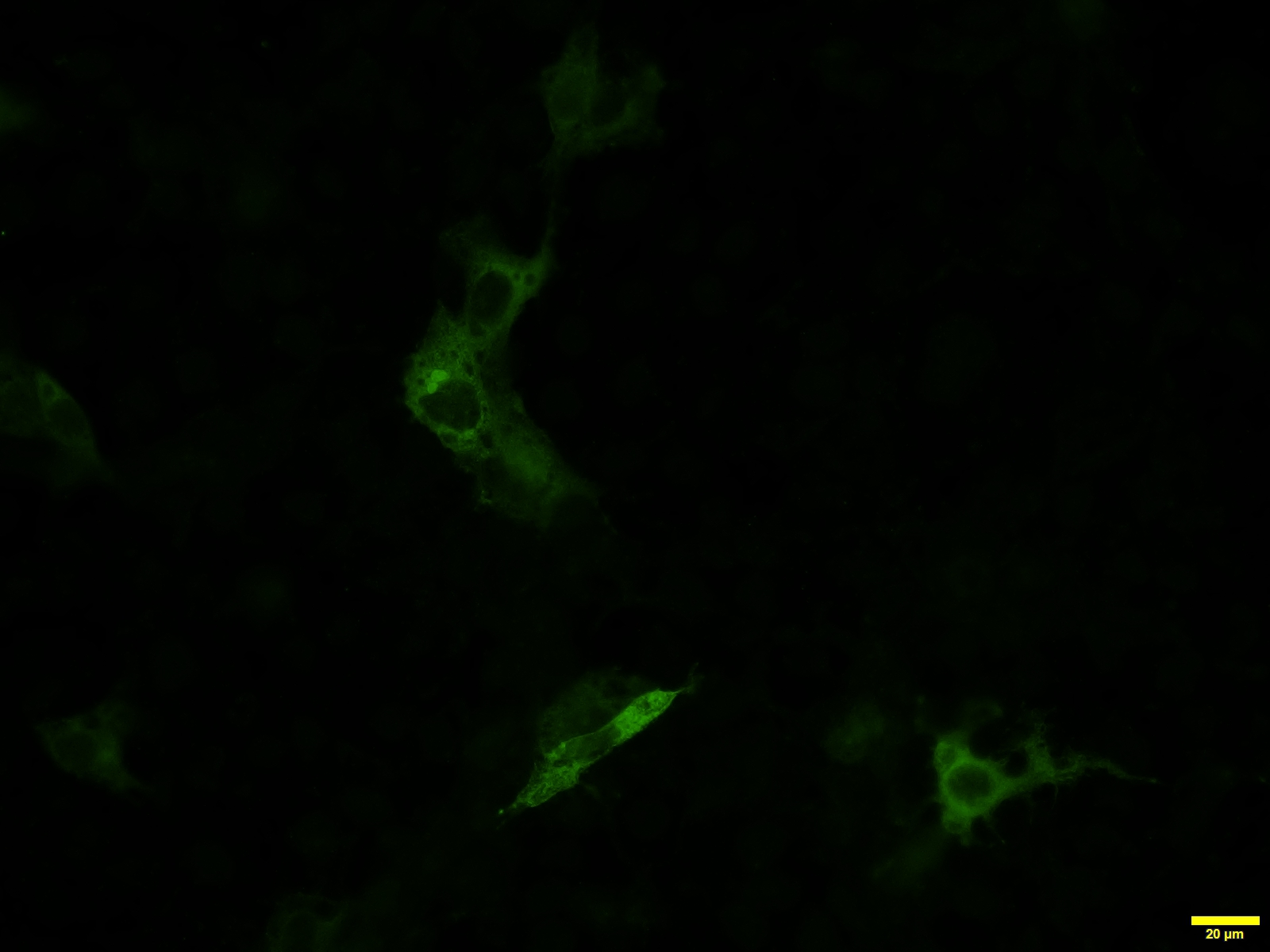

Supplement: Supplementary file 1 [file vetsci-12-00257-s001.zip › PABPC4 original blot images/Fig.3/D/N+P4/图像_12558.jpg]

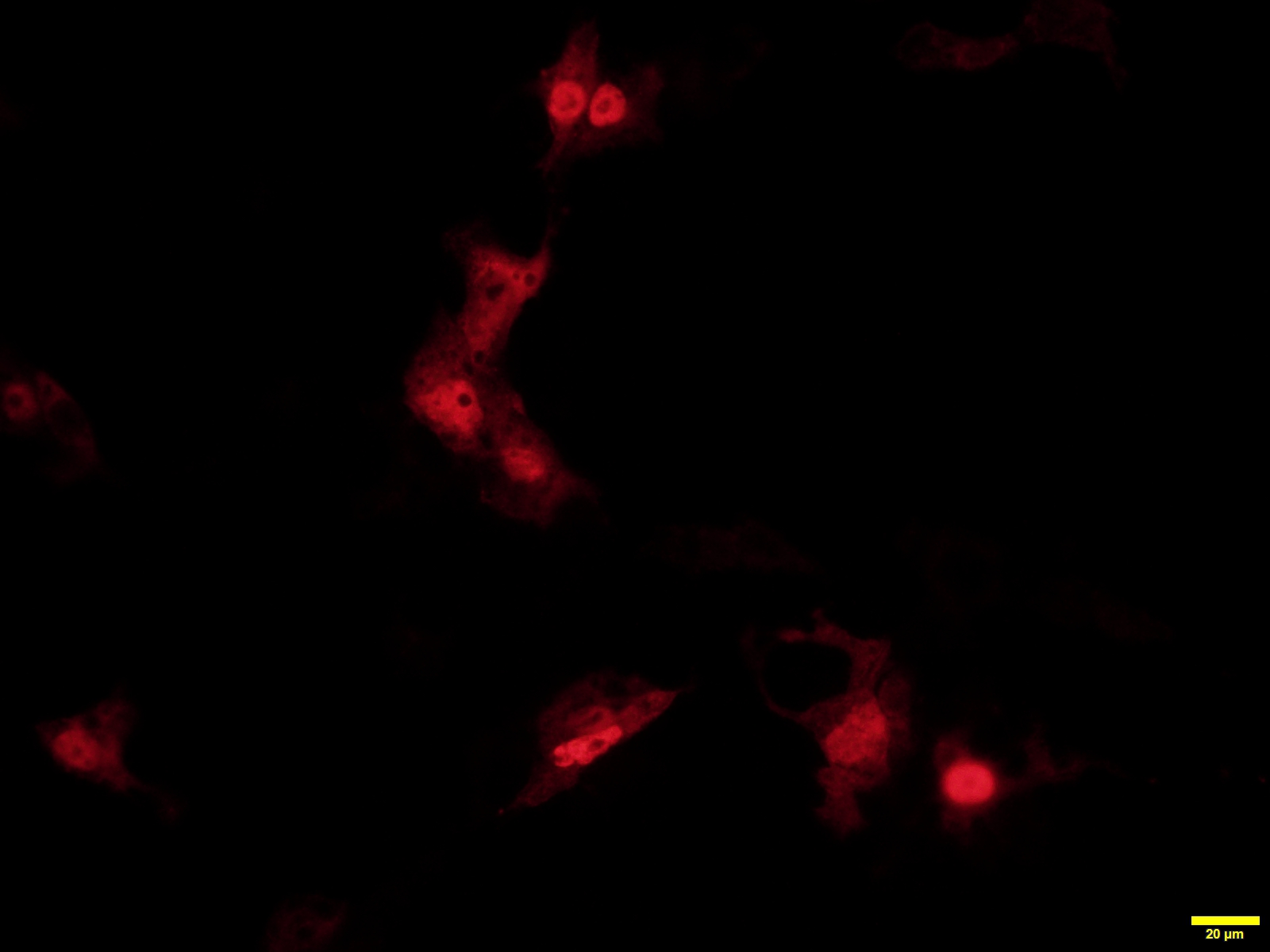

Supplement: Supplementary file 1 [file vetsci-12-00257-s001.zip › PABPC4 original blot images/Fig.3/D/N+P4/图像_12559.jpg]

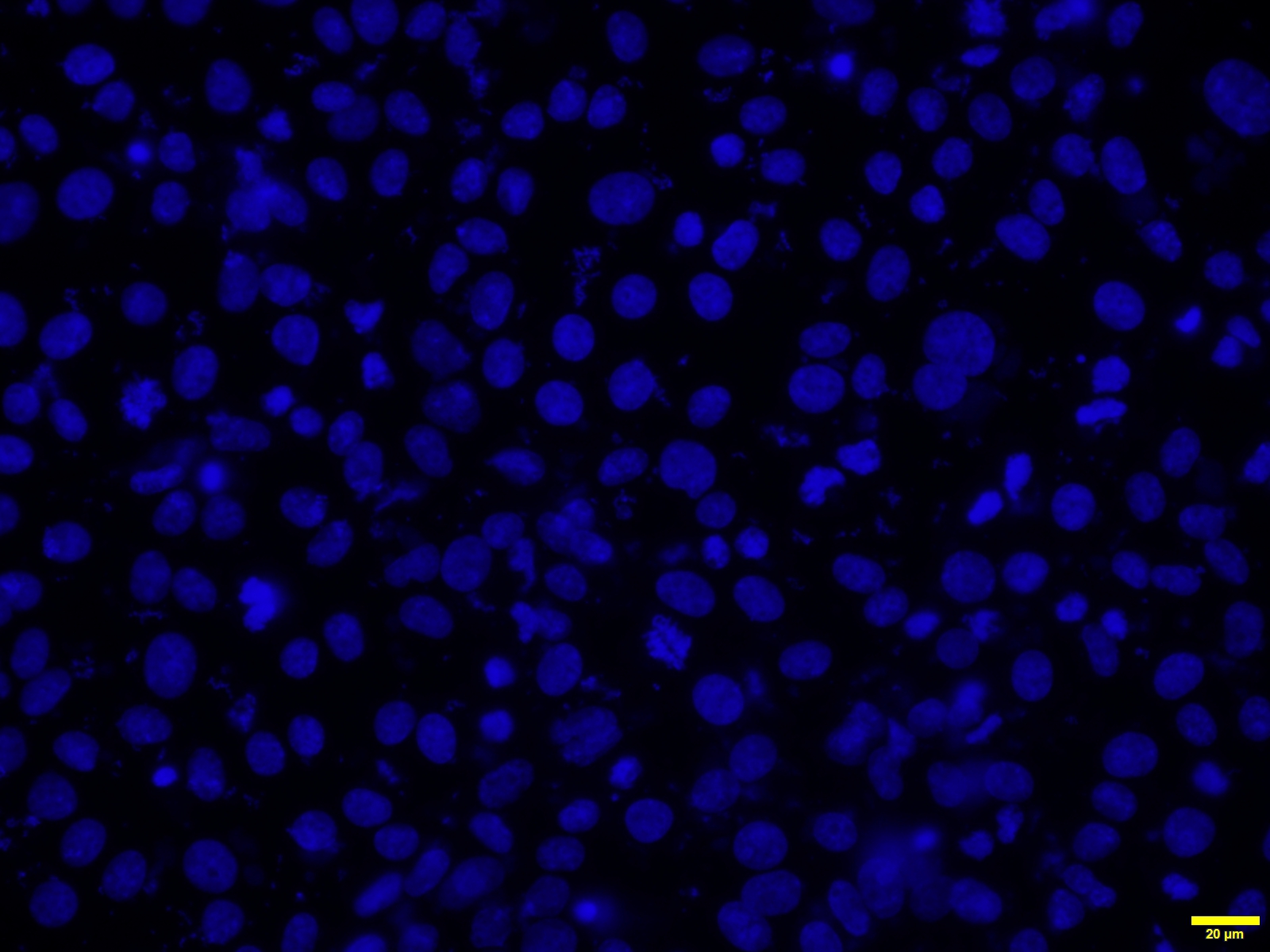

Supplement: Supplementary file 1 [file vetsci-12-00257-s001.zip › PABPC4 original blot images/Fig.3/D/N+P4/图像_12560.jpg]

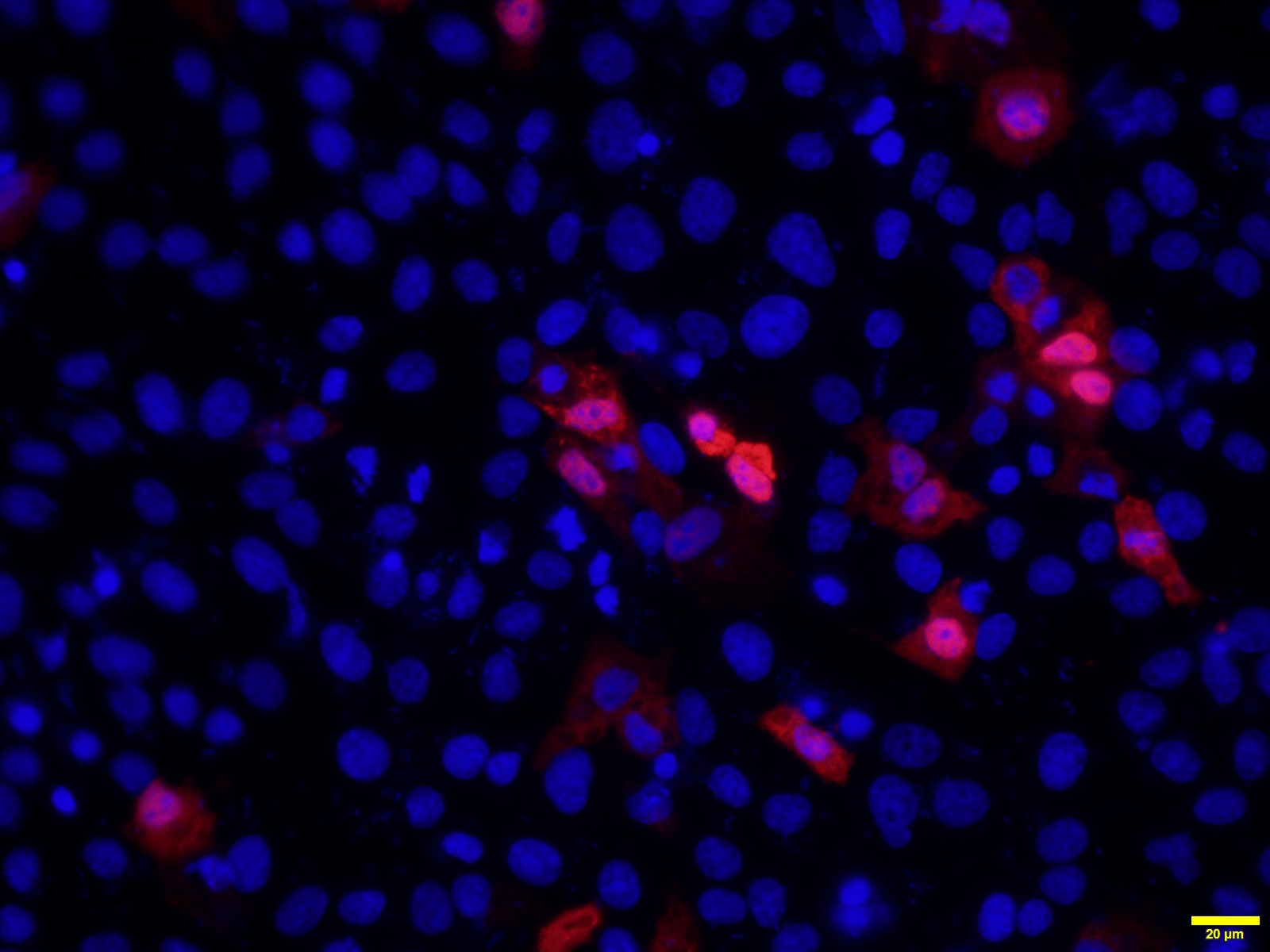

Supplement: Supplementary file 1 [file vetsci-12-00257-s001.zip › PABPC4 original blot images/Fig.3/D/P4/图像_01.jpg]

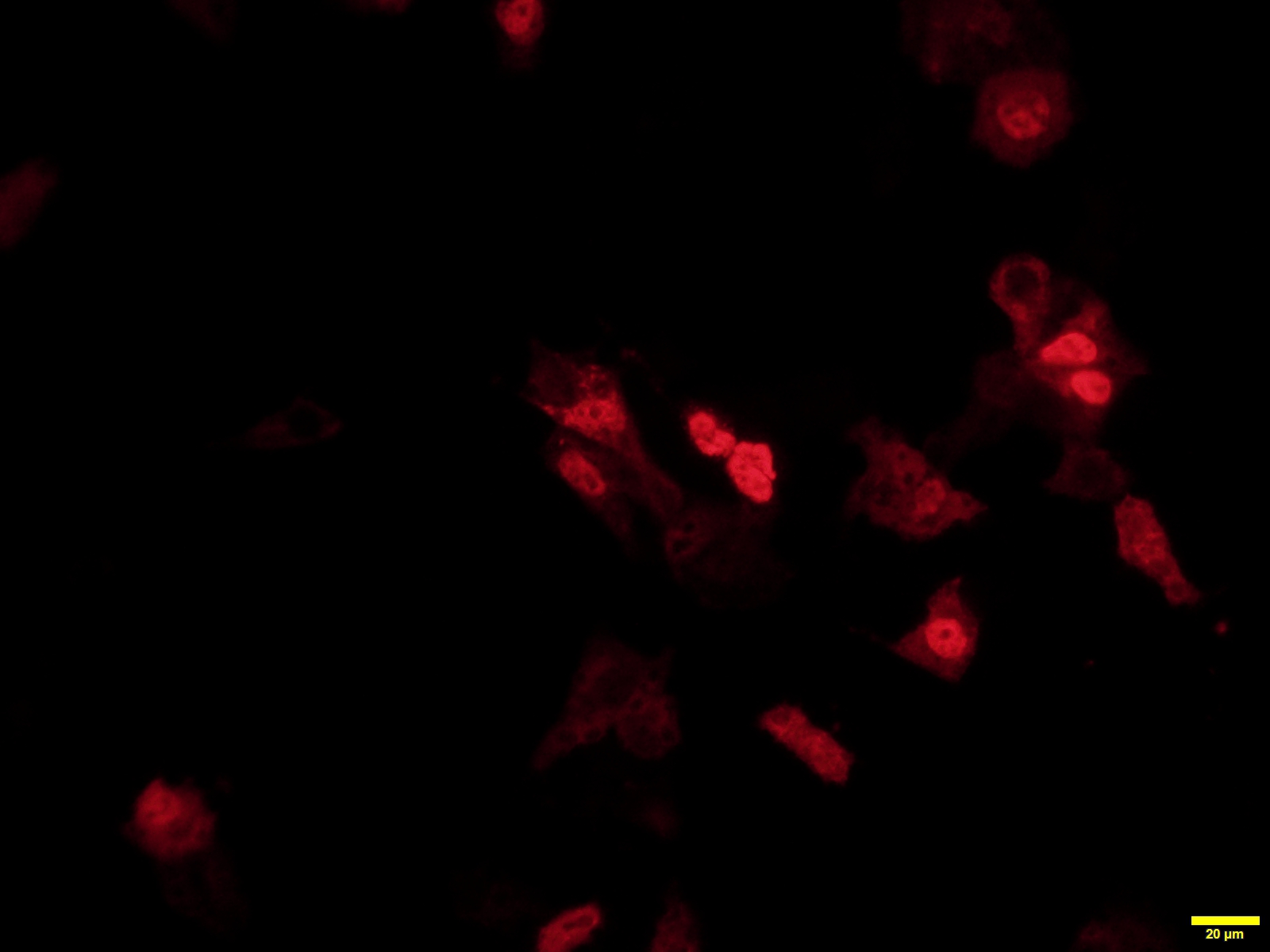

Supplement: Supplementary file 1 [file vetsci-12-00257-s001.zip › PABPC4 original blot images/Fig.3/D/P4/图像_12575.jpg]

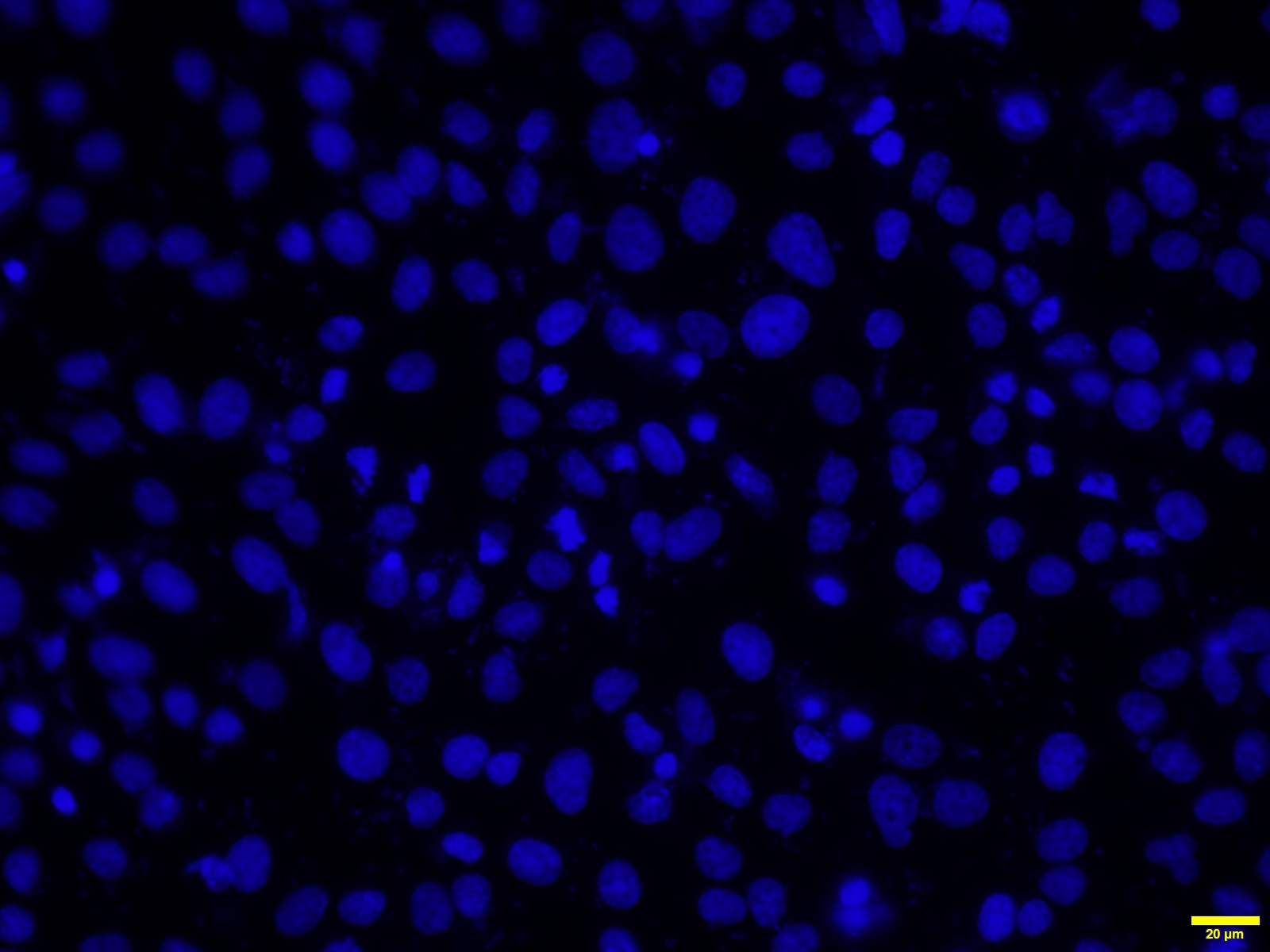

Supplement: Supplementary file 1 [file vetsci-12-00257-s001.zip › PABPC4 original blot images/Fig.3/D/P4/图像_12576.jpg]

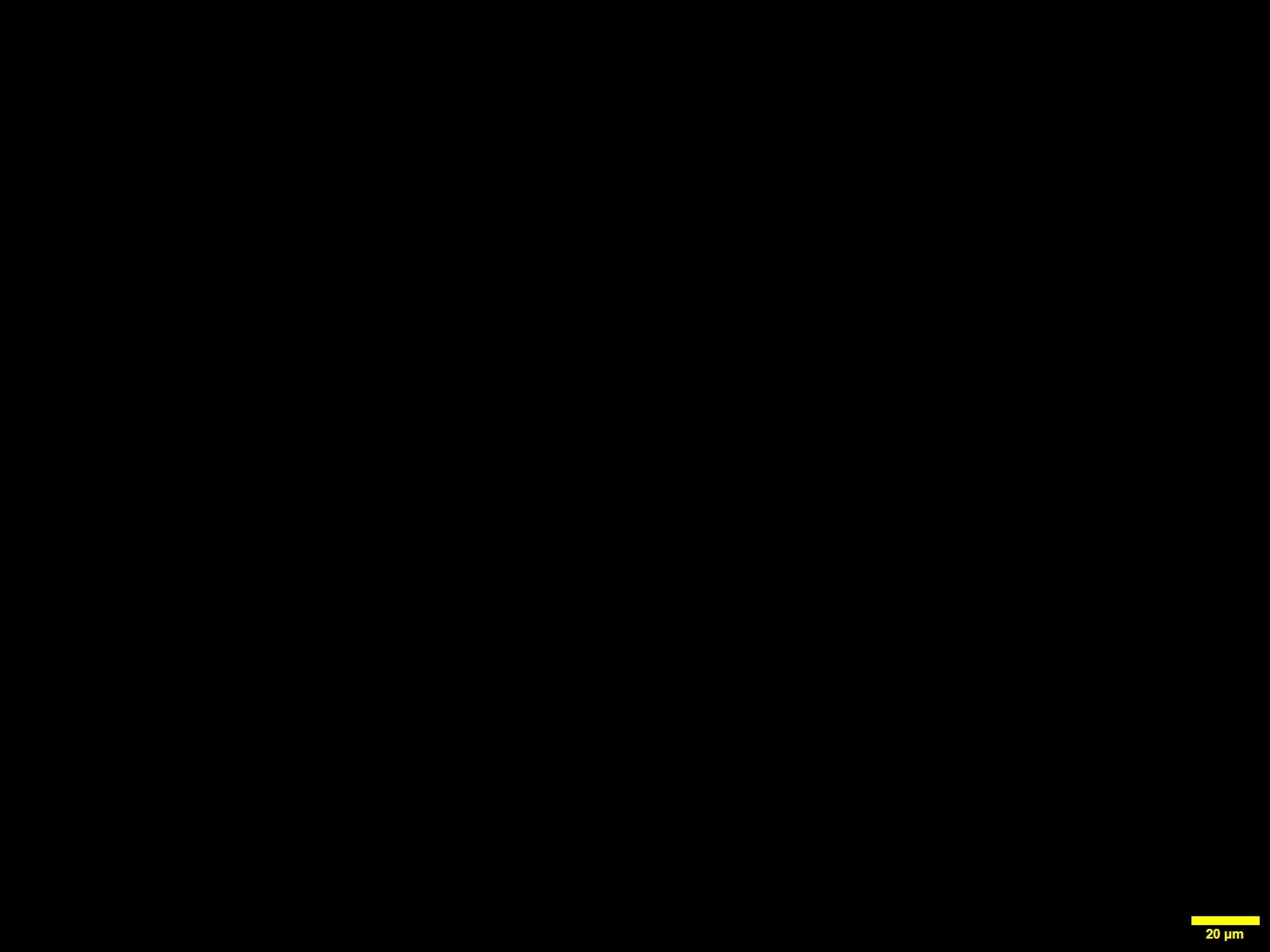

Supplement: Supplementary file 1 [file vetsci-12-00257-s001.zip › PABPC4 original blot images/Fig.3/D/P4/图像_12577.jpg]

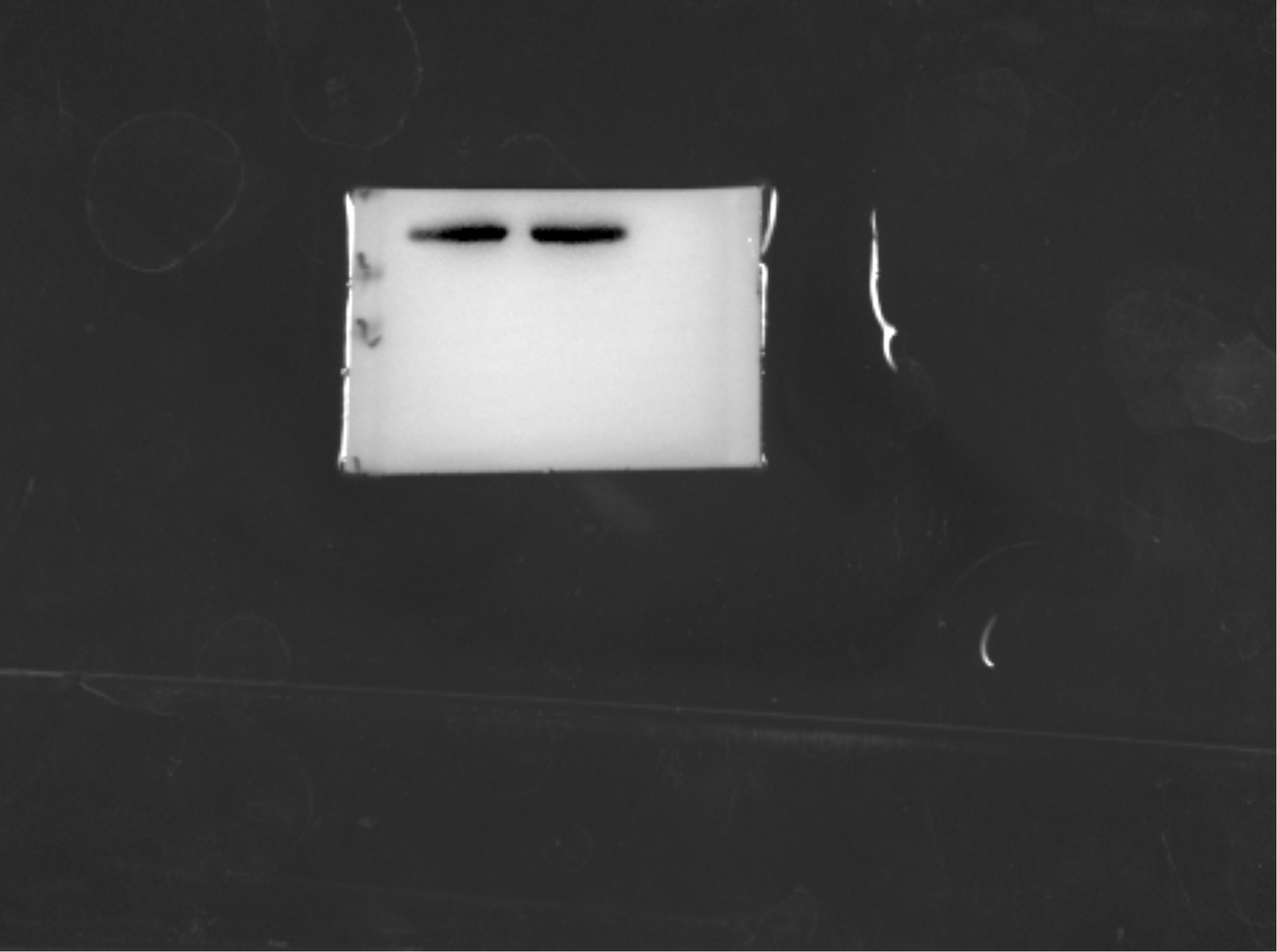

Supplement: Supplementary file 1 [file vetsci-12-00257-s001.zip › PABPC4 original blot images/Fig.3/E/ip 接毒/gapdh/h.tif]

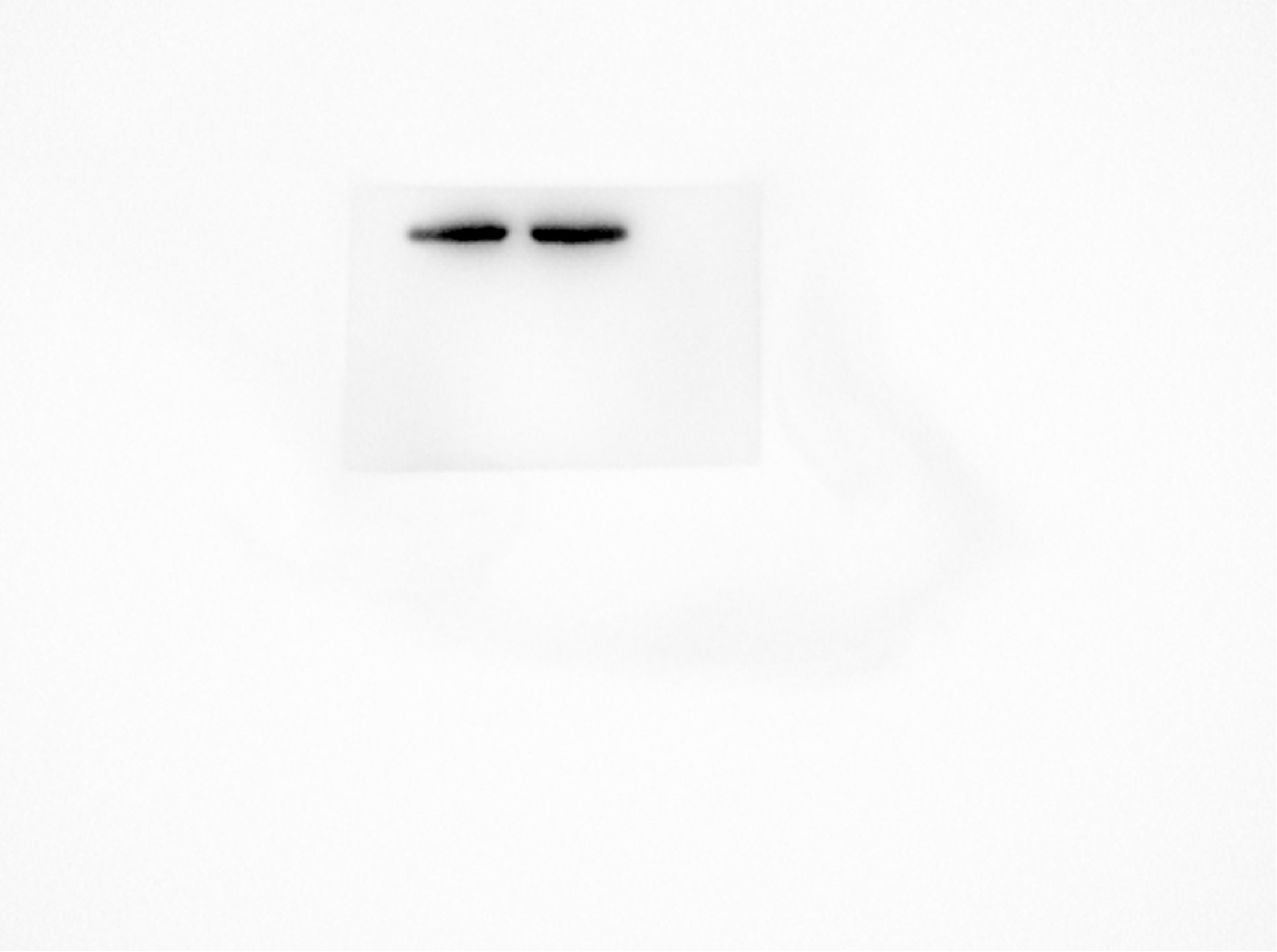

Supplement: Supplementary file 1 [file vetsci-12-00257-s001.zip › PABPC4 original blot images/Fig.3/E/ip 接毒/gapdh/s.tif]

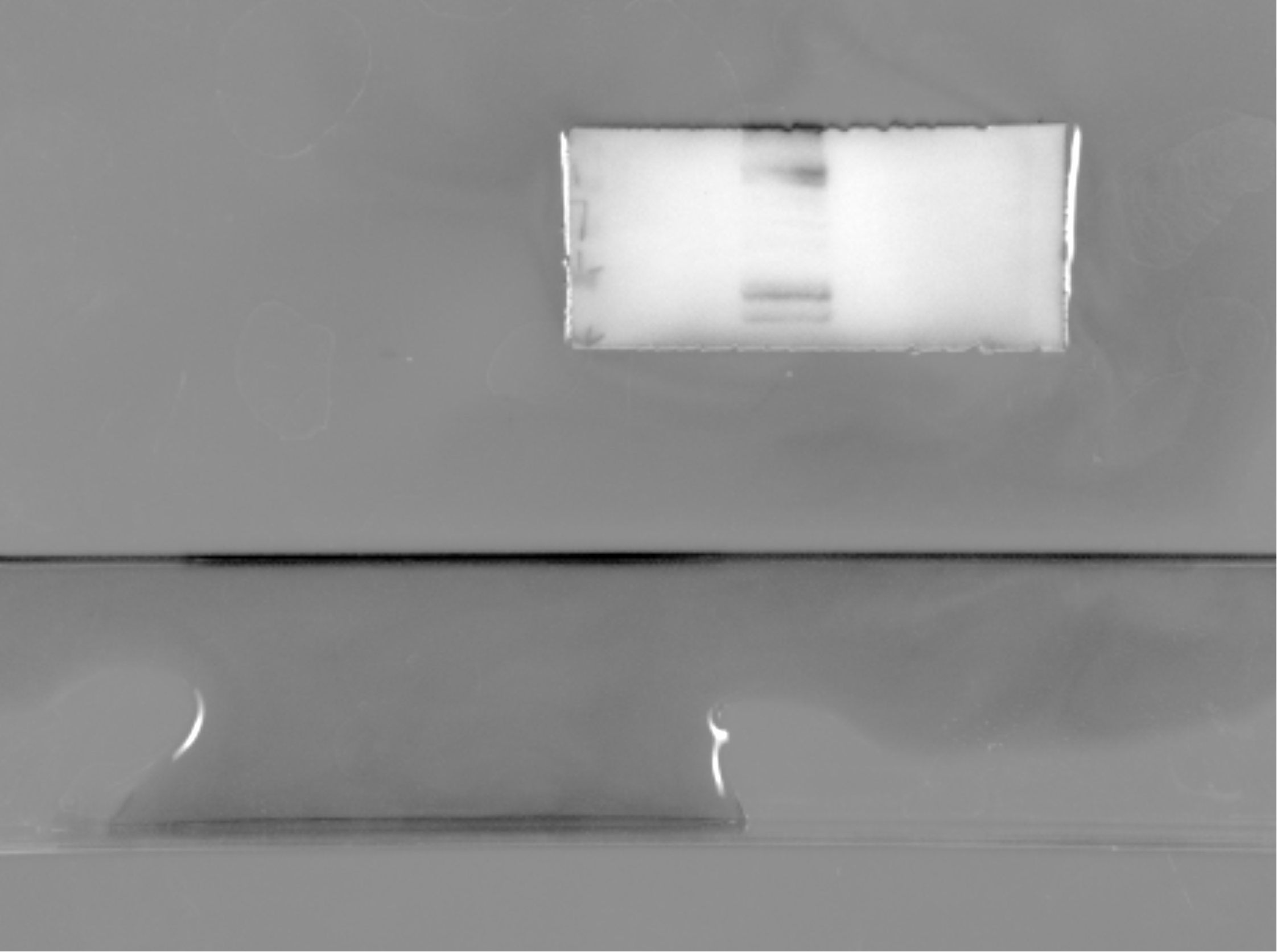

Supplement: Supplementary file 1 [file vetsci-12-00257-s001.zip › PABPC4 original blot images/Fig.3/E/ip 接毒/ib ha/h.tif]

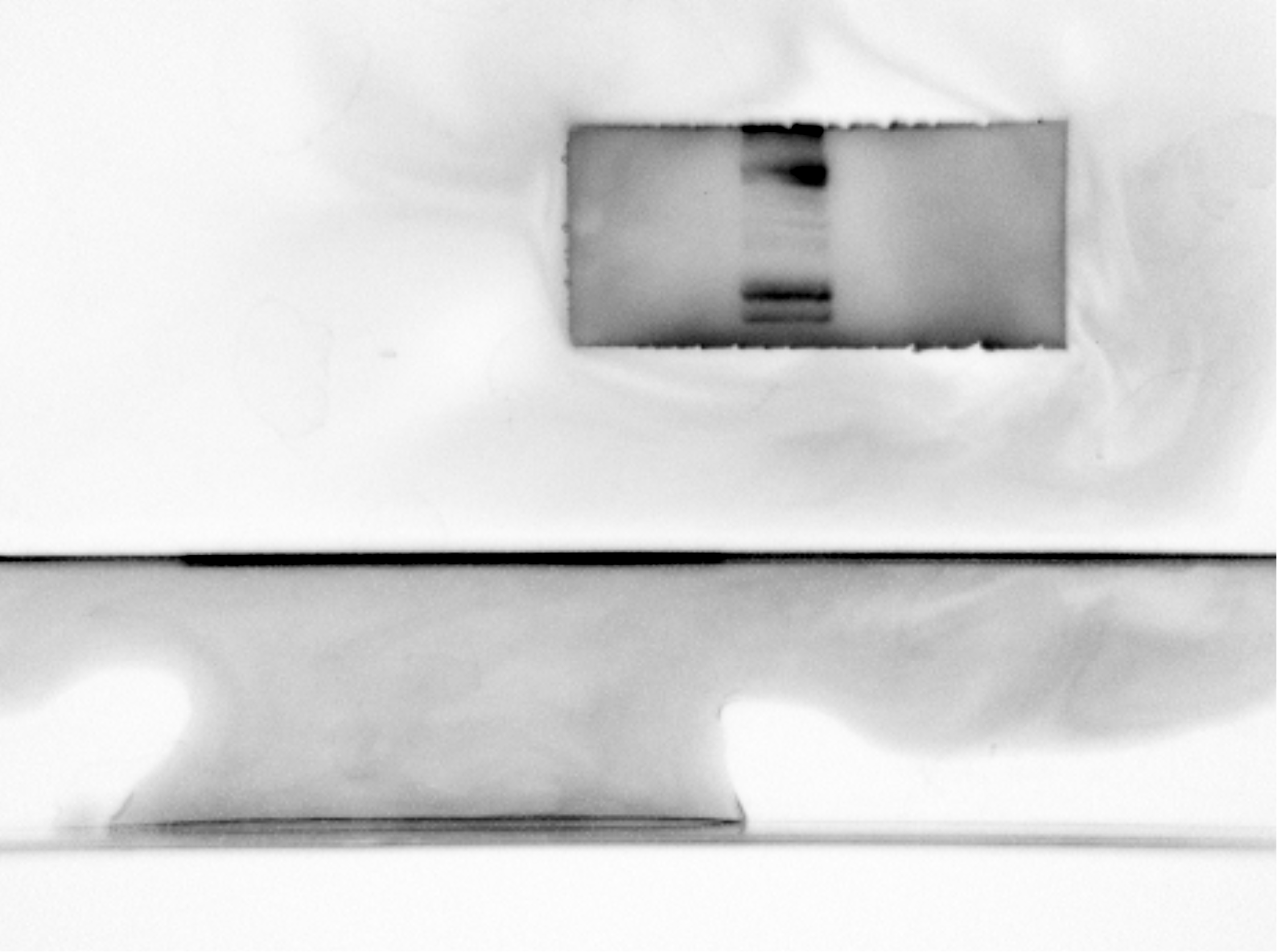

Supplement: Supplementary file 1 [file vetsci-12-00257-s001.zip › PABPC4 original blot images/Fig.3/E/ip 接毒/ib ha/s.tif]

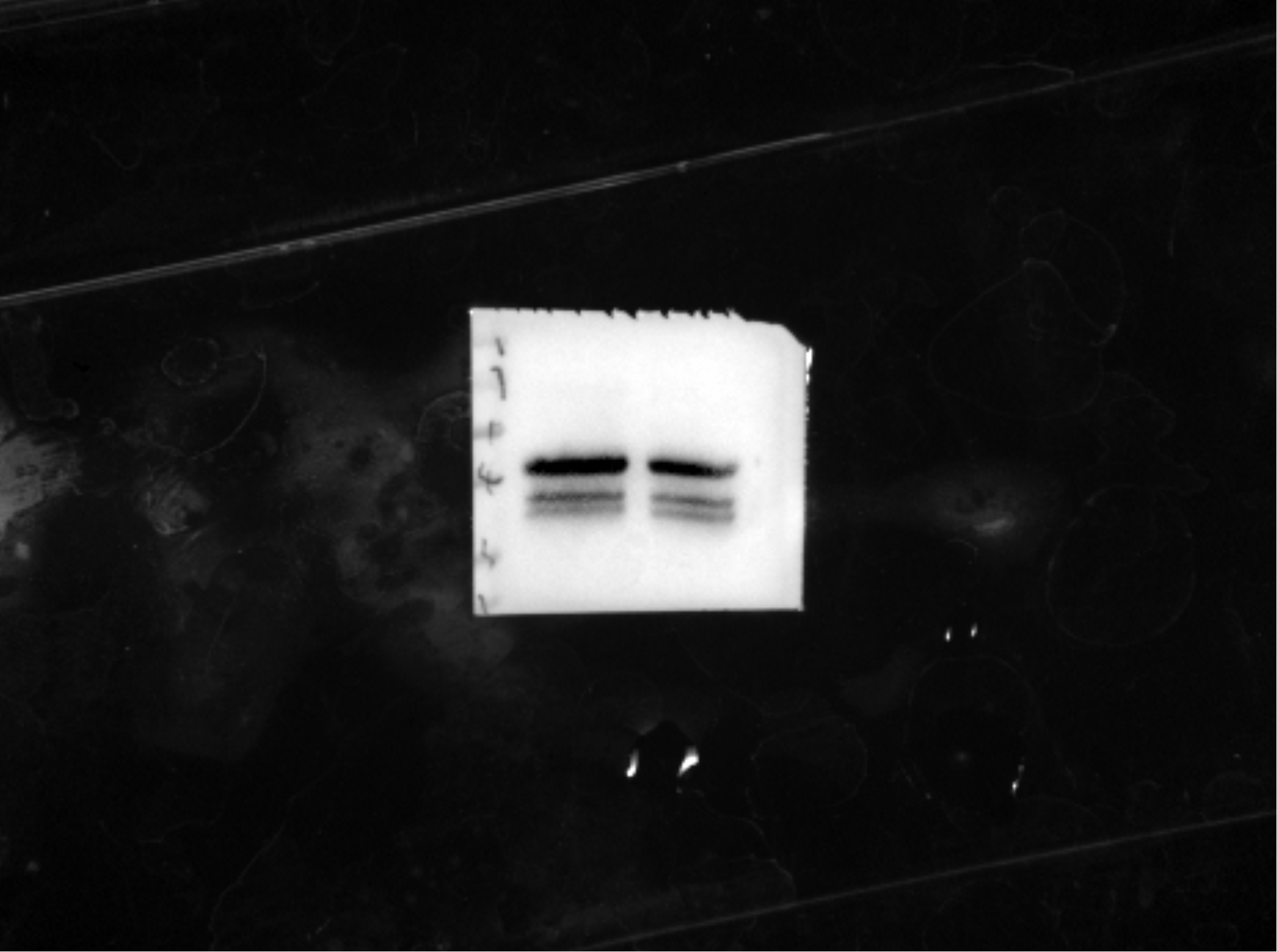

Supplement: Supplementary file 1 [file vetsci-12-00257-s001.zip › PABPC4 original blot images/Fig.3/E/ip 接毒/ib N-2/H.tif]

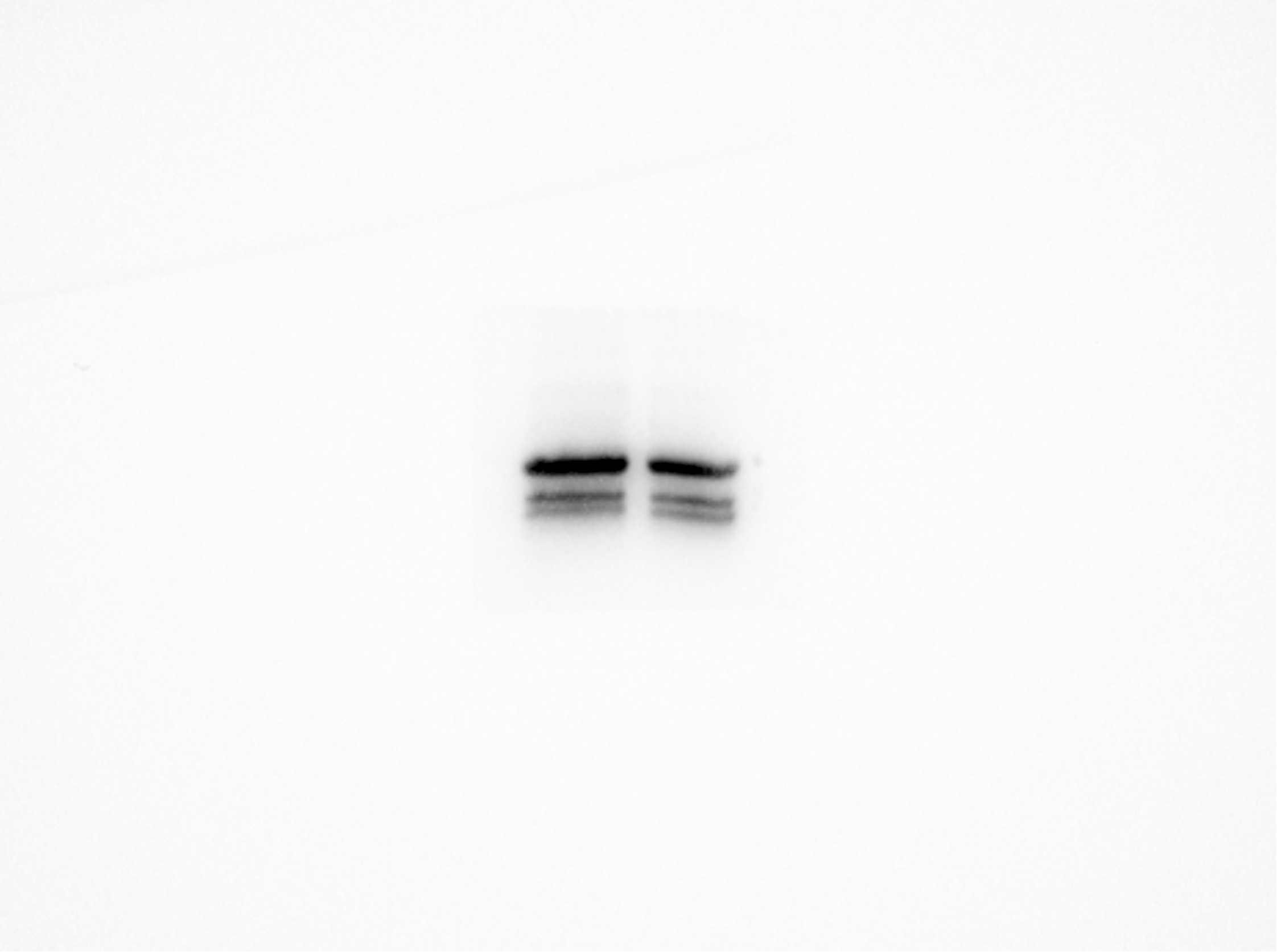

Supplement: Supplementary file 1 [file vetsci-12-00257-s001.zip › PABPC4 original blot images/Fig.3/E/ip 接毒/ib N-2/S.tif]

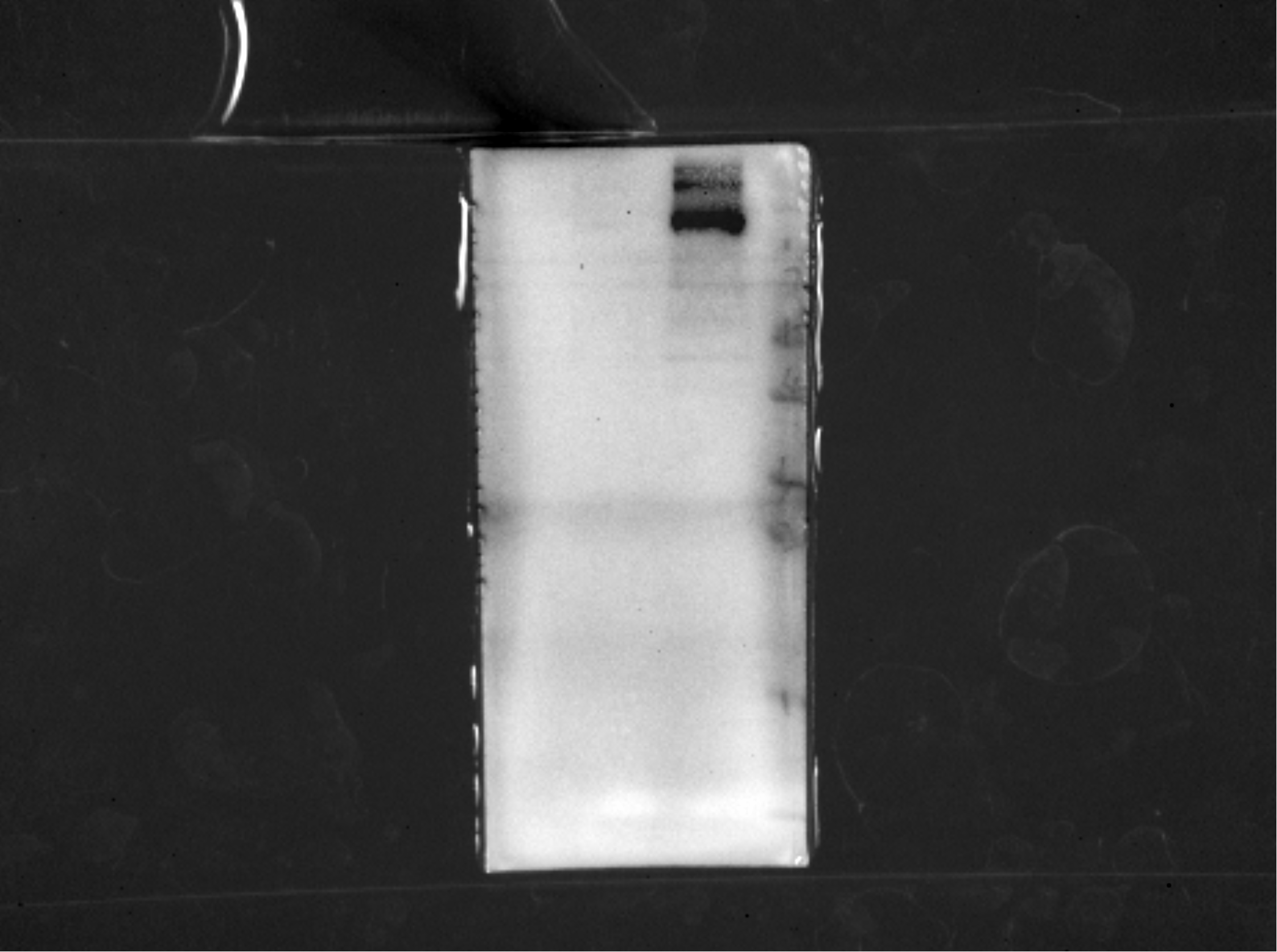

Supplement: Supplementary file 1 [file vetsci-12-00257-s001.zip › PABPC4 original blot images/Fig.3/E/ip 接毒/ip ha/merge.tif]

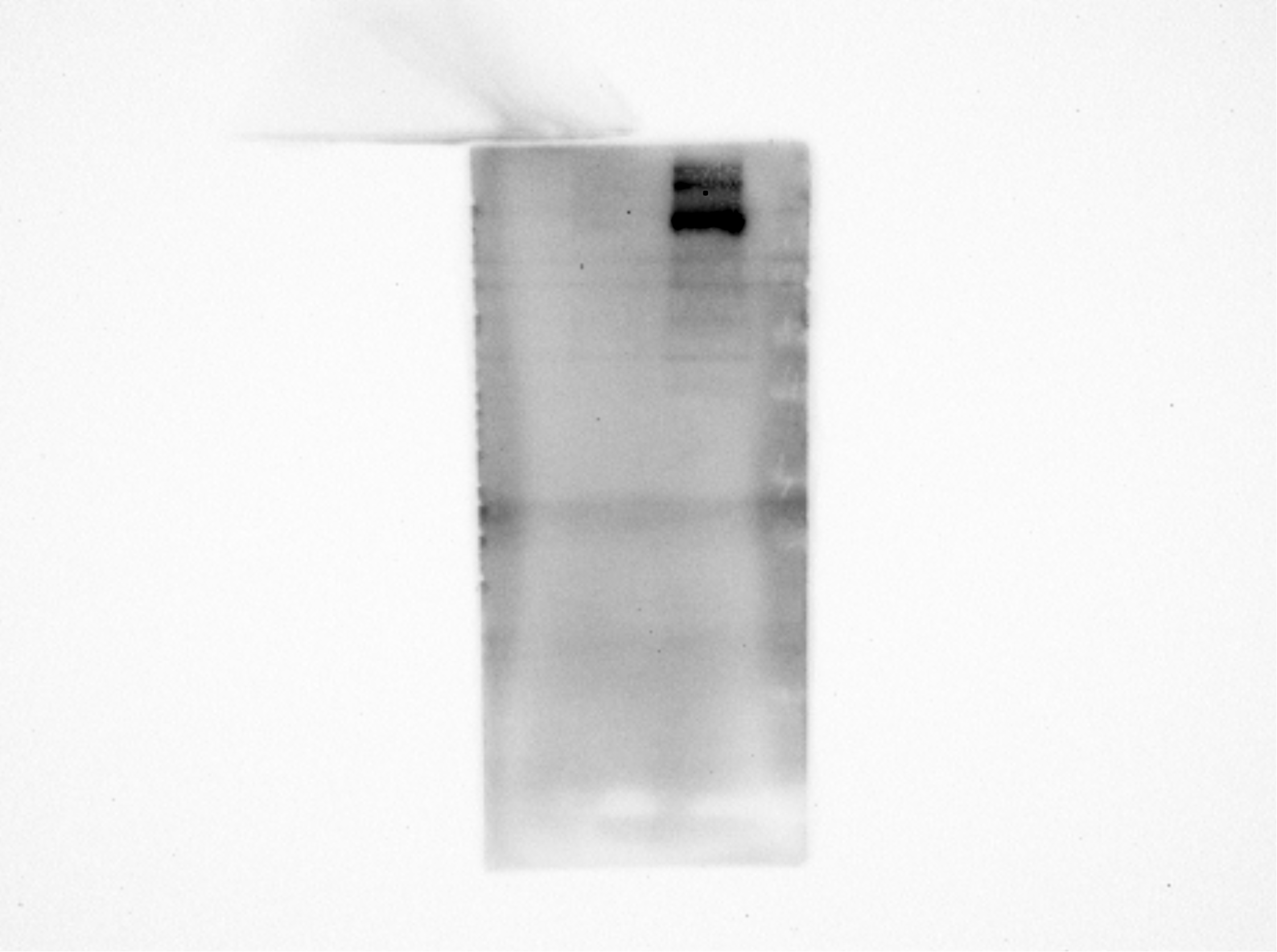

Supplement: Supplementary file 1 [file vetsci-12-00257-s001.zip › PABPC4 original blot images/Fig.3/E/ip 接毒/ip ha/s.tif]

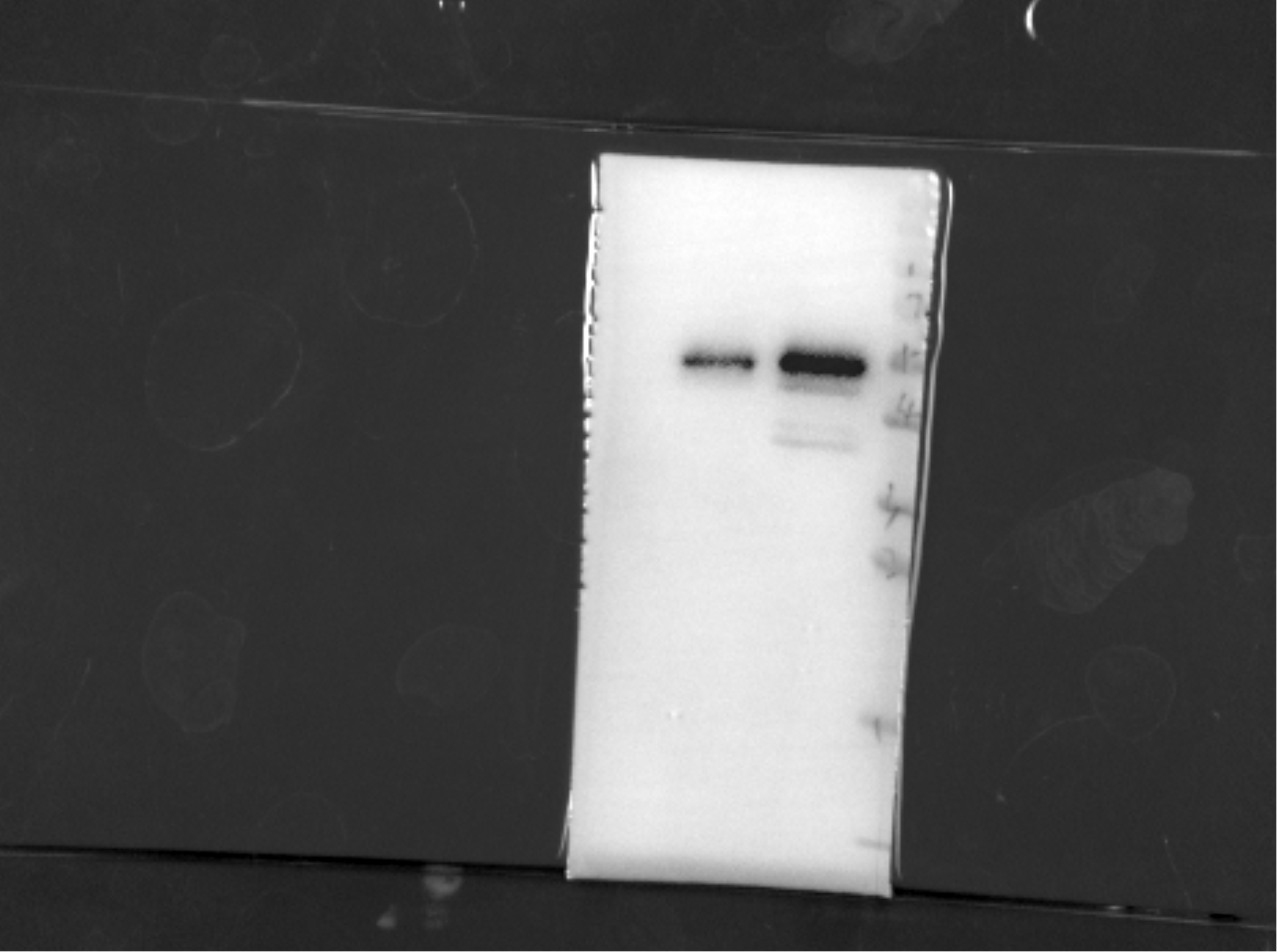

Supplement: Supplementary file 1 [file vetsci-12-00257-s001.zip › PABPC4 original blot images/Fig.3/E/ip 接毒/ip n/h.tif]

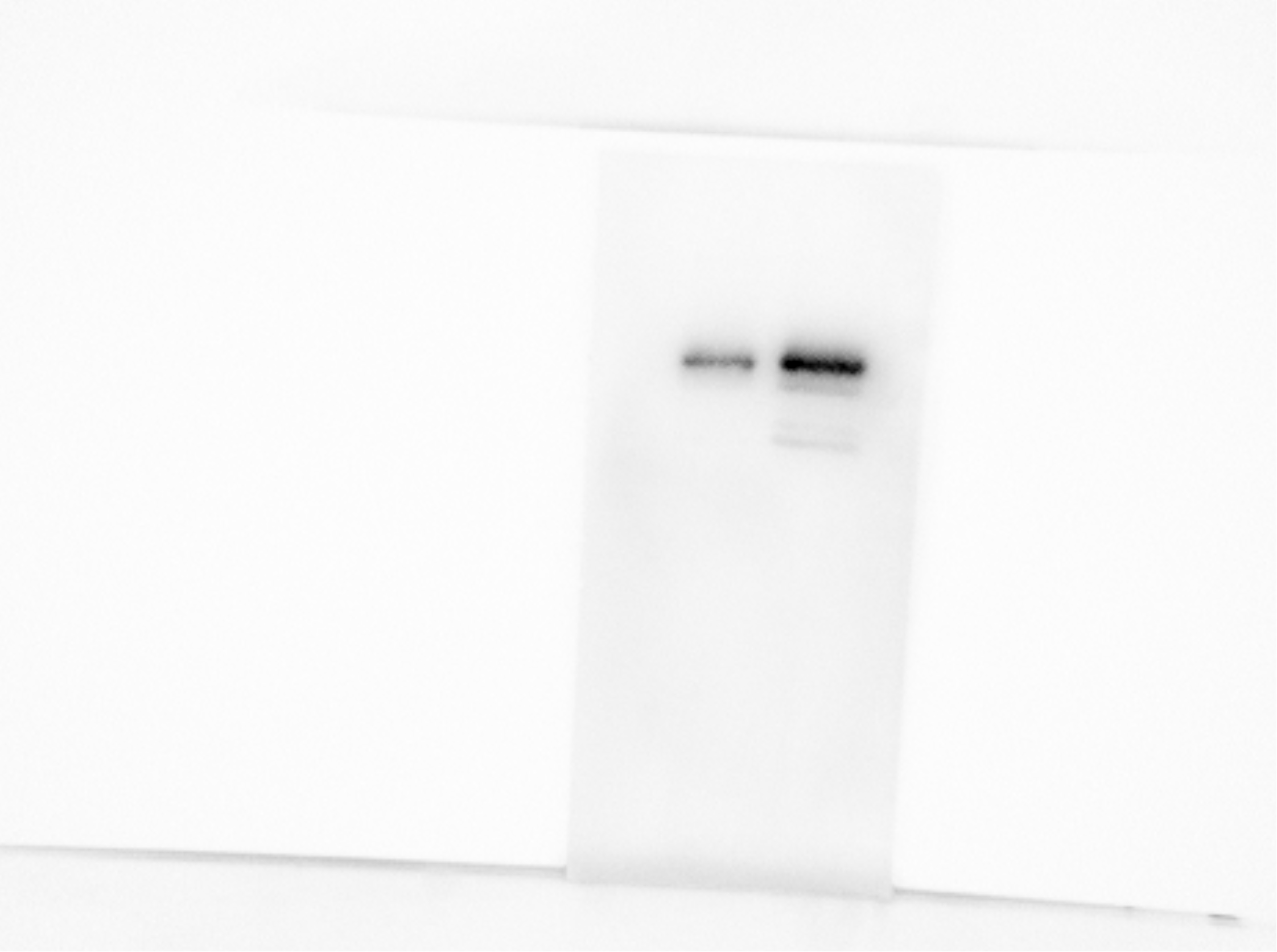

Supplement: Supplementary file 1 [file vetsci-12-00257-s001.zip › PABPC4 original blot images/Fig.3/E/ip 接毒/ip n/s.tif]

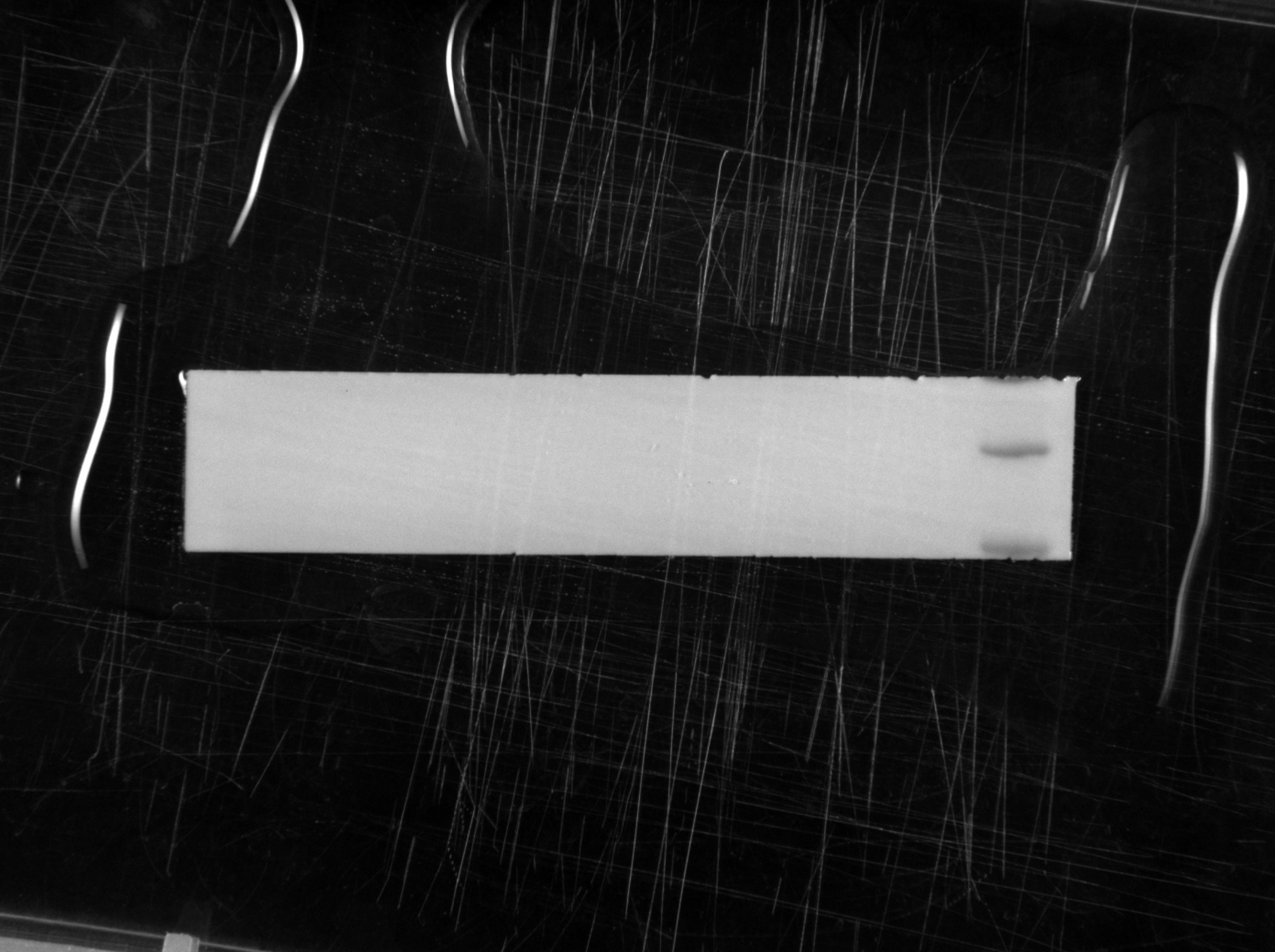

Supplement: Supplementary file 1 [file vetsci-12-00257-s001.zip › PABPC4 original blot images/Fig.3/F/flag-n/m.tif]

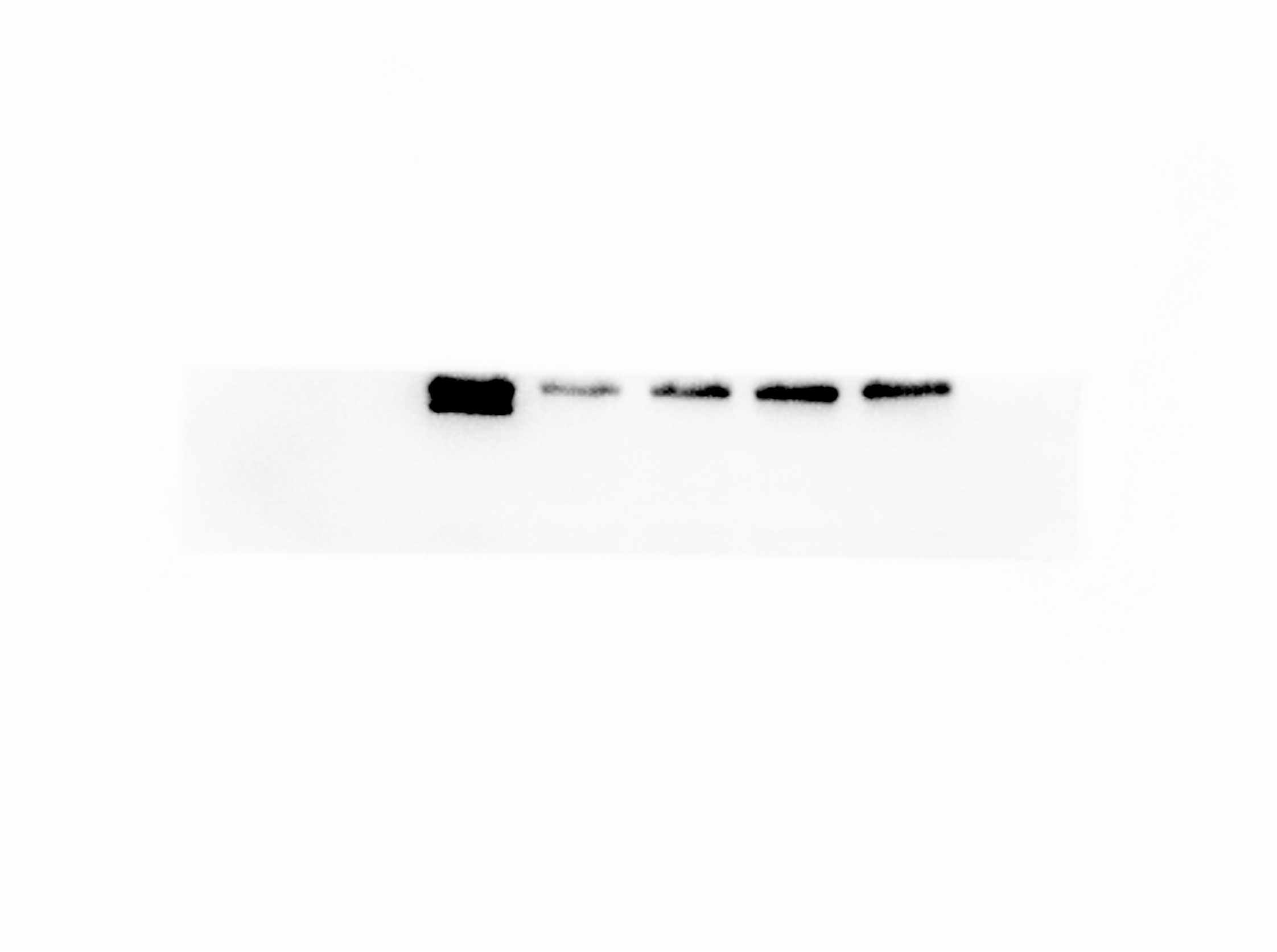

Supplement: Supplementary file 1 [file vetsci-12-00257-s001.zip › PABPC4 original blot images/Fig.3/F/flag-n/s.tif]

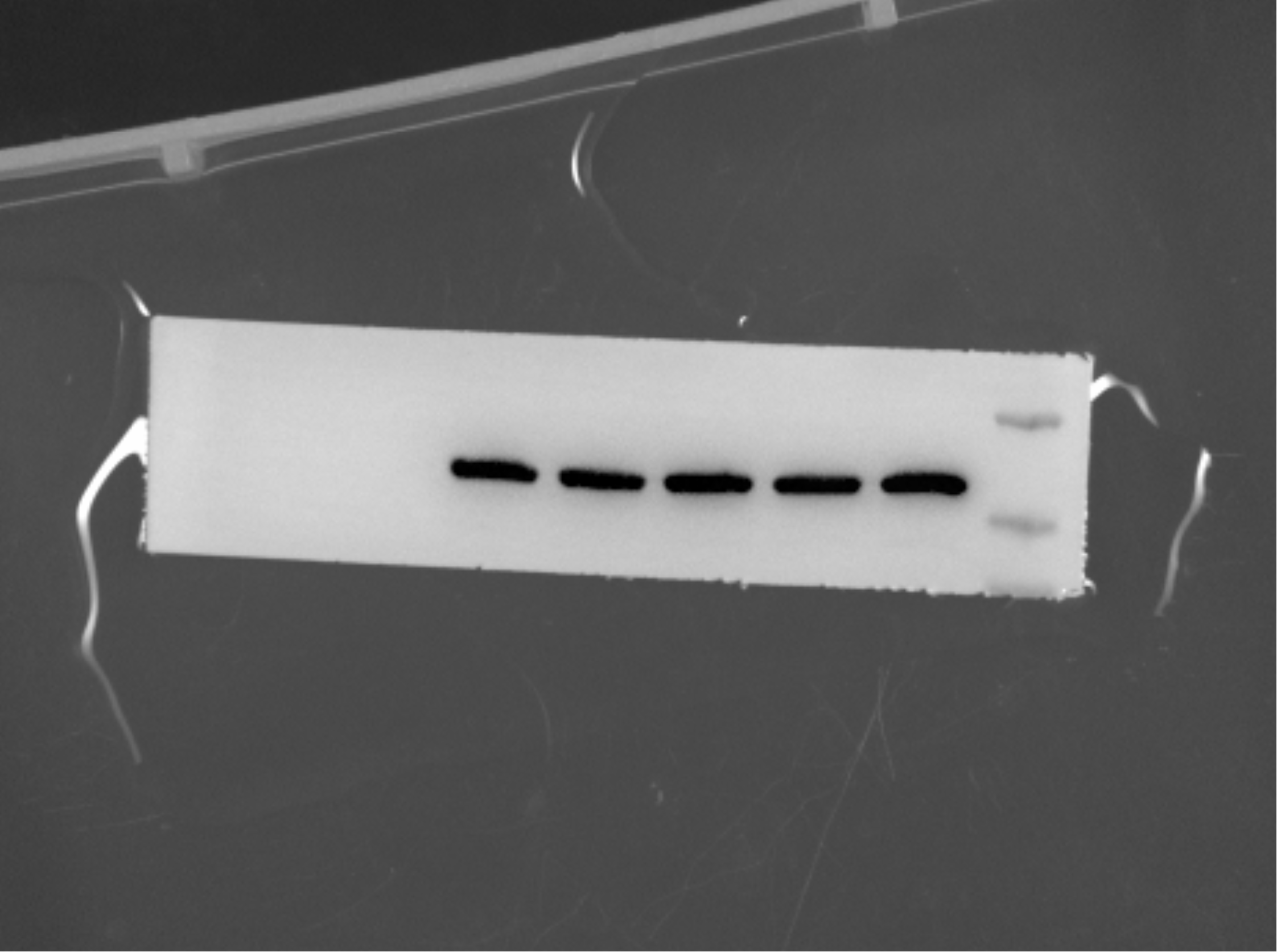

Supplement: Supplementary file 1 [file vetsci-12-00257-s001.zip › PABPC4 original blot images/Fig.3/F/gapdh/h.tif]

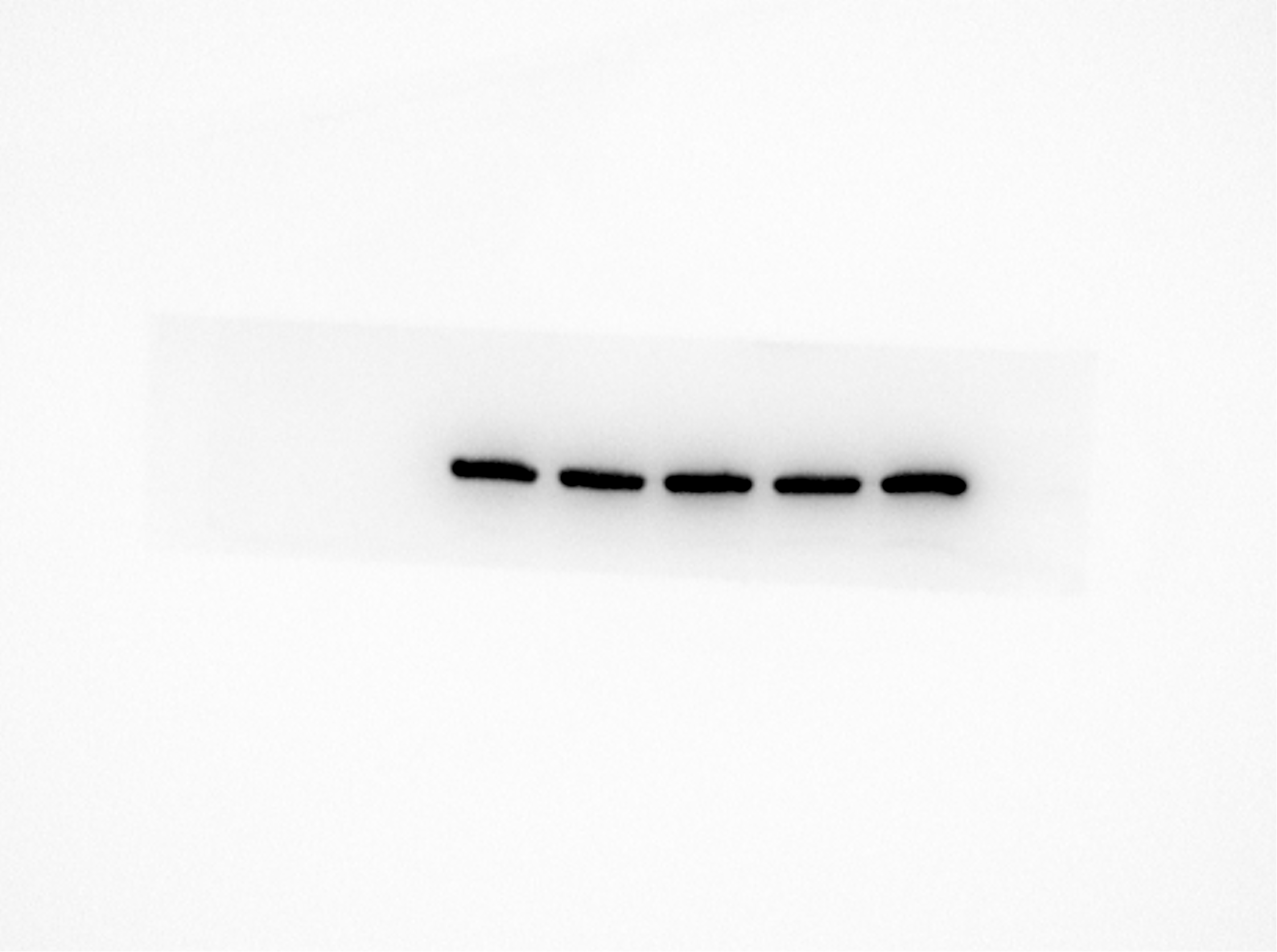

Supplement: Supplementary file 1 [file vetsci-12-00257-s001.zip › PABPC4 original blot images/Fig.3/F/gapdh/s.tif]

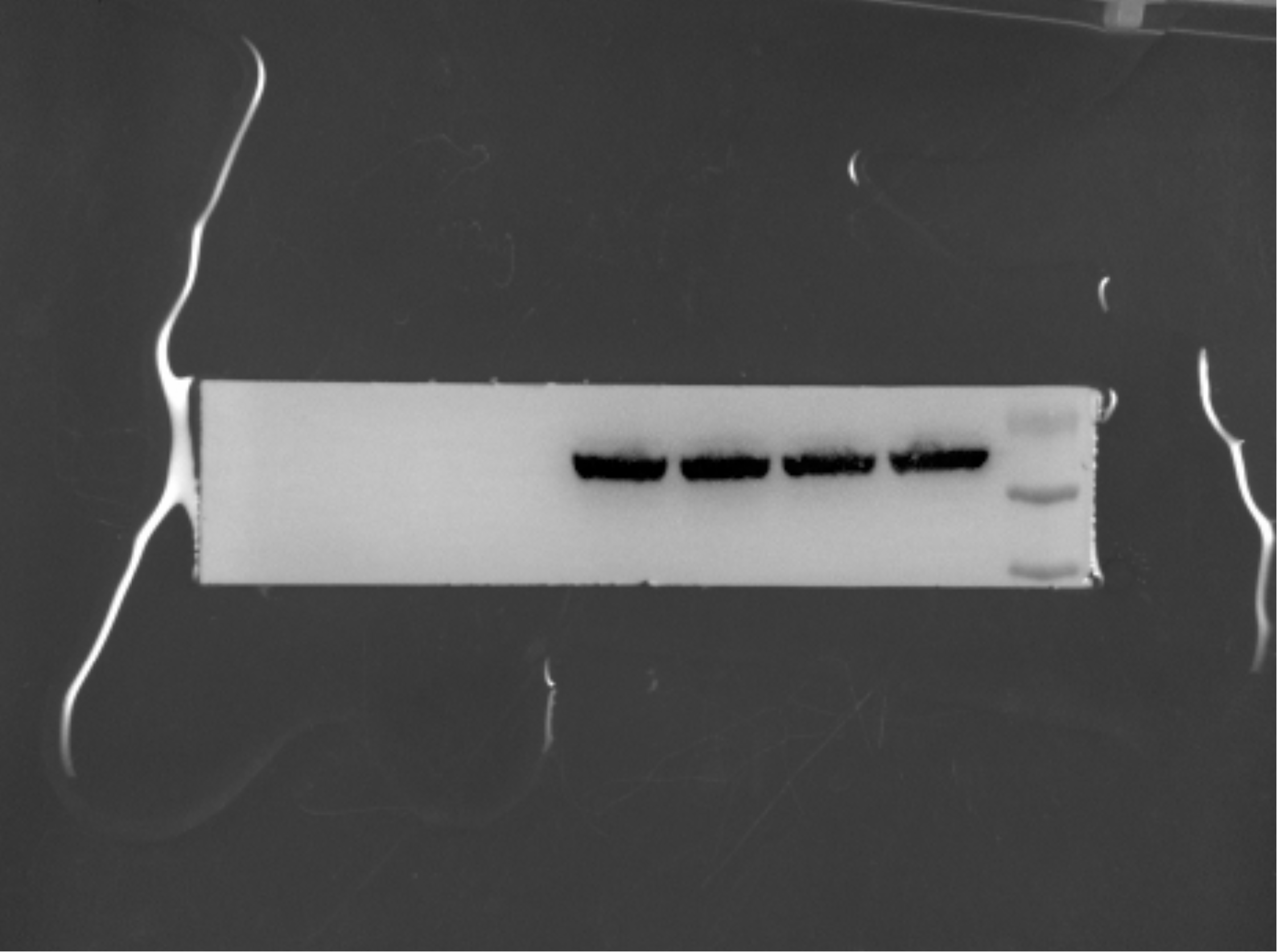

Supplement: Supplementary file 1 [file vetsci-12-00257-s001.zip › PABPC4 original blot images/Fig.3/F/p4/h.tif]

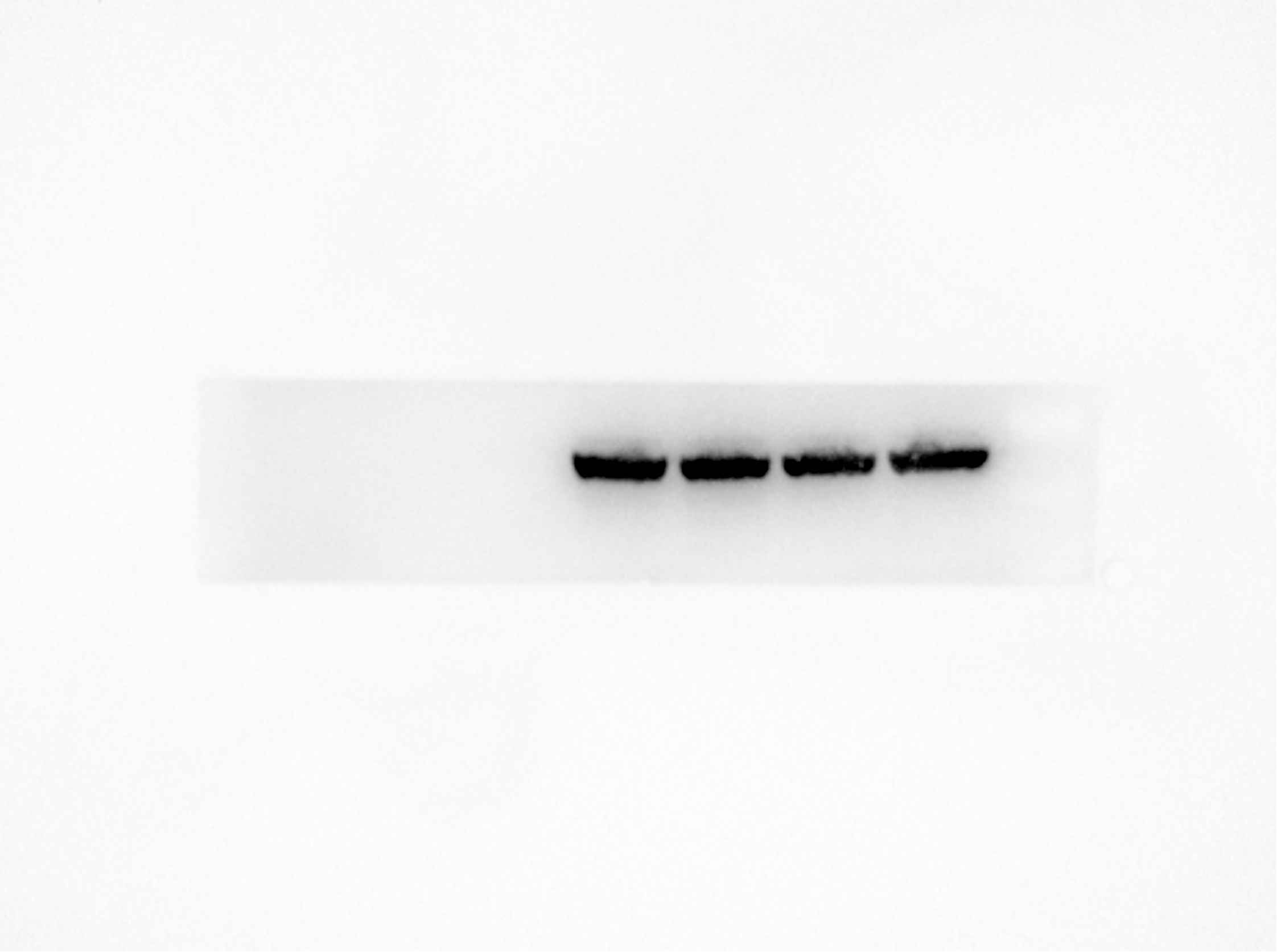

Supplement: Supplementary file 1 [file vetsci-12-00257-s001.zip › PABPC4 original blot images/Fig.3/F/p4/s.tif]

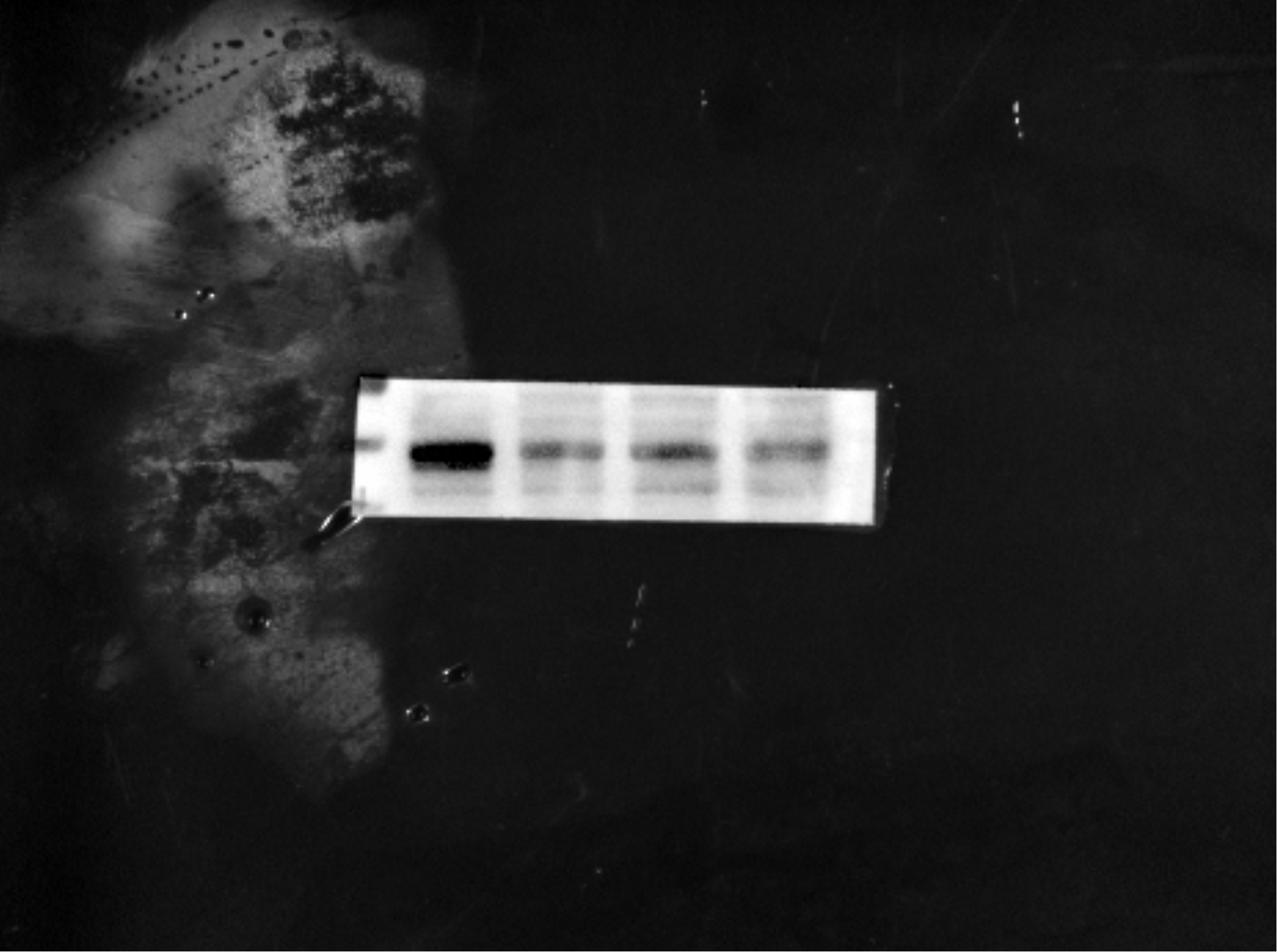

Supplement: Supplementary file 1 [file vetsci-12-00257-s001.zip › PABPC4 original blot images/Fig.4/A/flag/merge.tif]

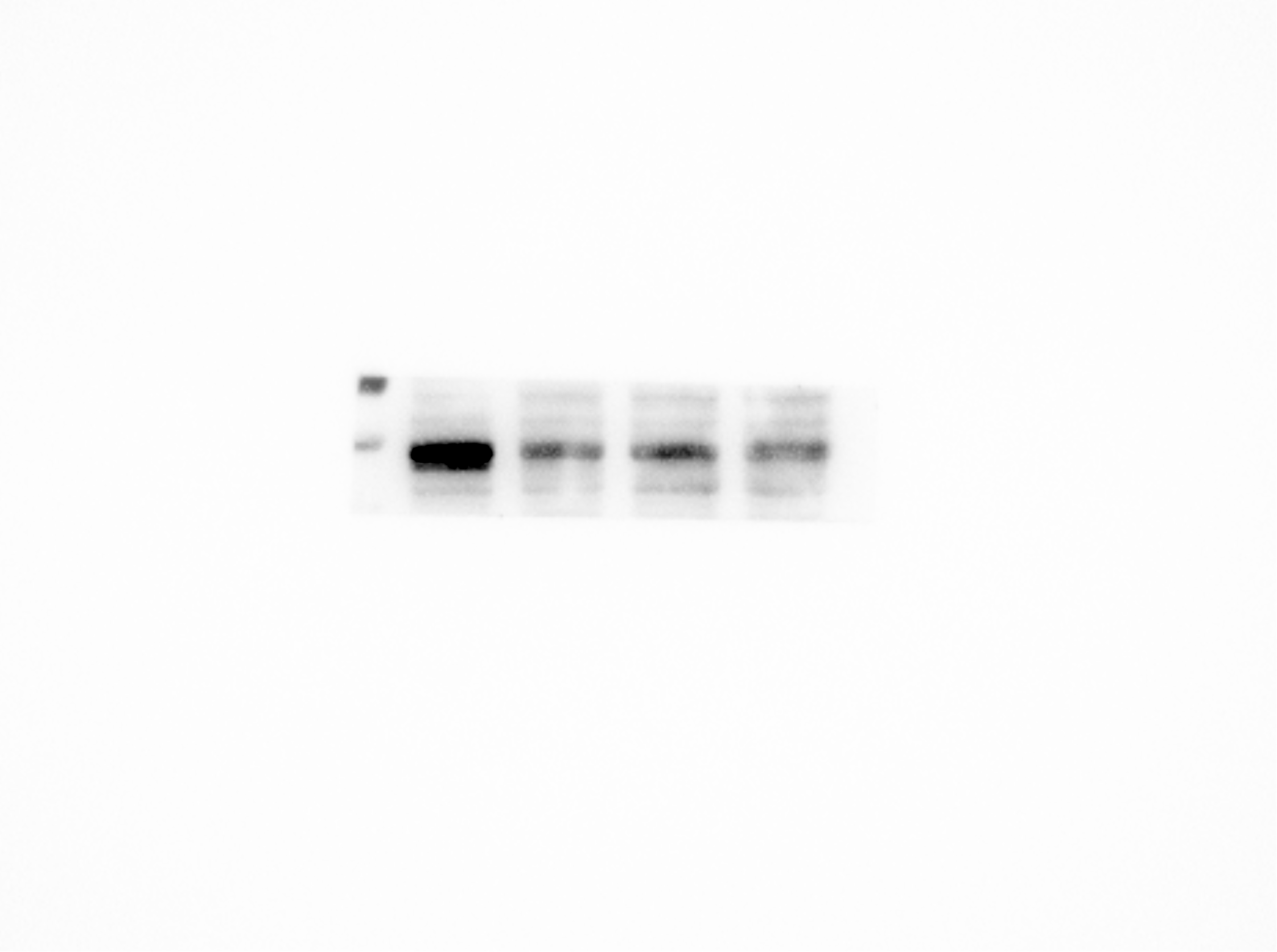

Supplement: Supplementary file 1 [file vetsci-12-00257-s001.zip › PABPC4 original blot images/Fig.4/A/flag/shiyantu.tif]
